# Proteomic analysis of *Plasmodium falciparum* response to isocryptolepine derivative

**DOI:** 10.1371/journal.pone.0220871

**Published:** 2019-08-08

**Authors:** Kitiya Rujimongkon, Mathirut Mungthin, Jumreang Tummatorn, Sumate Ampawong, Poom Adisakwattana, Usa Boonyuen, Onrapak Reamtong

**Affiliations:** 1 Department of Molecular Tropical Medicine and Genetics, Faculty of Tropical Medicine, Mahidol University, Bangkok, Thailand; 2 Department of Pharmacology, Phramongkutklao College of Medicine, Bangkok, Thailand; 3 Program on Chemical Biology, Chulabhorn Graduate Institute, Center of Excellence on Environmental Health and Toxicology, Ministry of Education, Bangkok, Thailand; 4 Laboratory of Medicinal Chemistry, Chulabhorn Research Institute, Bangkok, Thailand; 5 Department of Tropical Pathology, Faculty of Tropical Medicine, Mahidol University, Bangkok, Thailand; 6 Department of Helminthology, Faculty of Tropical Medicine, Mahidol University, Bangkok, Thailand; Instituto Rene Rachou, BRAZIL

## Abstract

Drug-resistant strains of malaria parasites have emerged for most of antimalarial medications. A new chemotherapeutic compound is needed for malarial therapy. Antimalarial activity against both drug-sensitive and drug-resistant *P*. *falciparum* has been reported for an isocryptolepine derivative, 8-bromo-2-fluoro-5-methyl-5H-indolo[3,2-c]quinoline (ICL-M), which also showed less toxicity to human cells. ICL-M has indoloquinoline as a core structure and its mode of action remains unclear. Here, we explored the mechanisms of ICL-M in *P*. *falciparum* by assessing the stage-specific activity, time-dependent effect, a proteomic analysis and morphology. Since human topo II activity inhibition has been reported as a function of isocryptolepine derivatives, malarial topo II activity inhibition of ICL-M was also examined in this study. The ICL-M exhibited antimalarial activity against both the ring and trophozoite stages of *P*. *falciparum*. Our proteomics analysis revealed that a total of 112 *P*. *falciparum* proteins were differentially expressed after ICL-M exposure; among these, 58 and 54 proteins were upregulated and downregulated, respectively. Proteins localized in the food vacuole, nucleus, and cytoplasm showed quantitative alterations after ICL-M treatment. A bioinformatic analysis revealed that pathways associated with ribosomes, proteasomes, metabolic pathways, amino acid biosynthesis, oxidative phosphorylation, and carbon metabolism were significantly different in *P*. *falciparum* treated with ICL-M. Moreover, a loss of ribosomes was clearly observed by transmission electron microscopy in the ICL-M-treated *P*. *falciparum*. This finding is in agreement with the proteomics data, which revealed downregulated levels of ribosomal proteins following ICL-M treatment. Our results provide important information about the mechanisms by which ICL-M affects the malaria parasite, which may facilitate the drug development of isocryptolepine derivatives.

## Introduction

**Fig 1:**
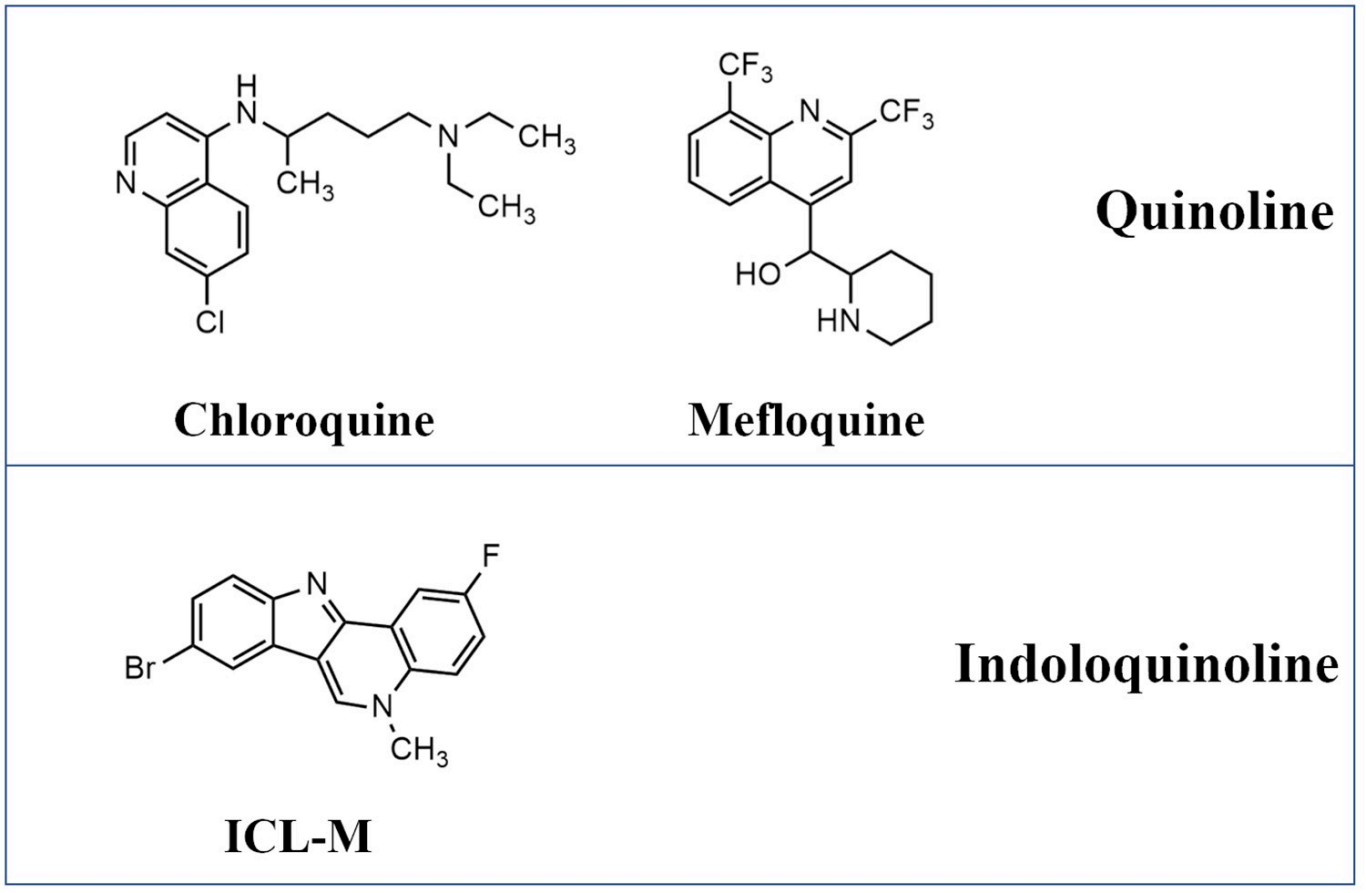
The structures of chloroquine, mefloquine and ICL-M.

Over the last decades, antimalarial drug resistance in *Plasmodium falciparum* has presented an obstacle for the malaria elimination program of the World Health Organization (WHO) [1]. Many studies have attempted to discover a new generation of antimalarial drugs. Herbal medicine has been the most common source of new candidate antimalarial compounds. *Cryptolepis sanguinolenta* is a Ghanaian traditional medicine used for malarial treatment. A report in 1989 showed that the aqueous root extract of this plant can successfully treat uncomplicated malaria in Ghana [2]. Isocryptolepine was first isolated from the roots of *C*. *sanguinolenta* in 1995, and its structure was determined to be 5-methyl-5H-indolo[3,2-c]quinoline. Isocryptolepine was later synthesized and tested for potential antimalarial properties. The results demonstrated that isocryptolepine possesses activity against both drug-sensitive and drug-resistance strains of *P*. *falciparum* [3–5]. Several strategies have been used to design and construct isocryptolepine derivatives with improved antimalarial activity [3–6]. In 2015, an isocryptolepine derivative containing fluorine and bromine, 8-bromo-2-fluoro-5-methyl-5H-indolo[3,2-c]quinoline (ICL-M), was reported to display an improved antimalarial activity against both drug-sensitive and drug-resistant strains and to induce less toxicity in human cells compared with the original isocryptolepine. The half maximal inhibitory concentration (IC_50_) of ICL-M in normal *Homo sapiens* lung cells was 121,944 nM, whereas the IC_50_ of chloroquine-resistant and mefloquine-resistant strains of *P*. *falciparum* were 132 and 211 nM, respectively [5]. Additionally, furfural, the agricultural byproduct, was found to possess the ability to be converted to isocryptolepine [7], suggesting the possibility of a low-cost synthesis process for this compound. Although ICL-M has indoloquinoline as its core structure, it could kill quinoline-resistant *P*. *falciparum* such as chloroquine-resistant and mefloquine-resistant strains [5]. Therefore, ICL-M may have different mechanisms for killing malaria parasite. The structure of chloroquine, mefloquine and ICL-M are shown in Fig 1. Isocryptolepine derivatives have been reported to target human topoisomerase II (topo II) in several different types of cancer cells [8]. However, the targets and modes of action of ICL-M in malaria parasites remain unclear. Understanding the molecular mechanism of ICL-M against malaria parasites may provide important insight into the mode of action for isocryptolepine derivatives as well as guidance for further structure development.

Proteomics is a high-throughput protein analysis that can be used to reveal the biological activity in parasites. Quantitative proteomics is a useful tool with which to study parasite gene expression during both the asexual and sexual stages [9]. This method has been applied to several studies of the host response to parasites [10, 11], the parasite response to its host [12–14], and drug target identification [15, 16]. In drug development, the differential protein expression between treated and non-treated parasites is commonly used as part of the pharmacodynamics information for a compound on parasite cells. Polyacrylamide gel electrophoresis (PAGE) coupled with mass spectrometry (MS) is a crucial method for identifying and quantifying proteins from large-scale antimalarial drug studies [17–19]. In malarial proteomics research, label-free quantification, such as the exponentially modified protein abundance index [20], has been applied for various types of samples, spanning the cultural blood stage [21], clinical blood stage [22], and mosquito stage [23]. Here, we aimed to apply a proteomics approach to investigating the mechanism of ICL-M against *P*. *falciparum*. The protein profile resulting from ICL-M exposure may provide crucial information for understanding the mechanism of isocryptolepine derivatives against malaria parasites and further facilitate antimalarial drug development.

## Methods

### 1. Cultivation of *P*. *falciparum* strain 3D7

*P*. *falciparum* 3D7 was cultured using O^+^ human erythrocytes in culture medium containing RPMI 1640 (Gibco, New York, USA) supplemented with 10% human serum, 2% sodium bicarbonate (Merck, New Jersey, USA), and 40 mg/L gentamycin sulphate (GPO, Bangkok, Thailand). Parasite culture was maintained at 37 °C under mixed gas (5% CO_2_, 5% O_2_, and 90% N_2_). The culture media was changed daily. Parasite growth was monitored by Giemsa-stained thin blood smears. Culture with mixed stages of *P*. *falciparum* was treated with 5% sorbitol (Amresco, Pennsylvania, USA) for 30 min at 37 °C for synchronization. After RPMI 1640 was used to wash the treated parasites, the parasite culture was continued [24]

### 2. Antimalarial activity assay

ICL-M was synthesized following the protocol developed by Aroonkit and colleagues in 2015 [5]. The compound was dissolved in dimethyl sulfoxide (DMSO) (Merck) to prepare a 10 mM stock concentration. The IC_50_ and ninety percent inhibitory concentration (IC_90_) were assessed to evaluate the antimalarial activity of ICL-M. Briefly, the compound was prepared at various concentrations in a 96-well plate. Synchronized parasites at 1% parasitemia (ring form) and 2% hematocrit were then loaded into the wells. All tests were performed in triplicate. The plate was further cultured for 24 h prior to [^3^H]hypoxanthine (PerkinElmer, Massachusetts, USA) labelling. After incubation for another 24 h, the surviving parasites were measured by radiolabeling. The inhibitory concentration was determined by an analysis of the dose-response curves calculated using GRAFIT software (Erithacus Software, Kent, UK).

### 3. Stage-specific antimalarial activity

Stage-specific antimalarial activity was analyzed. Ring (6–16 h after invasion) and trophozoite (30–34 h after invasion) stages were identified based on the criteria previously described by Silamut and colleagues [25]. Untreated and 450 nM ICL-M (three-fold the IC_50_)-treated parasites at 1% parasitemia in 2% hematocrit were incubated at 37 °C for 24 or 48 h. After incubation, the parasitemia percentages were estimated by Giemsa-stained thin blood smear visualized by microscopy.

### 4. Parasite topoisomerase II activity assay

Red blood cells (RBCs) infected with the trophozoite stage of *P*. *falciparum* were washed with 10 ml of RPMI 1640. Parasites were released from the RBCs by treatment with 0.15% saponin (Amresco). Free parasites were then washed three times with 10 ml of cold phosphate-buffered saline (PBS) (Merck). The resulting pellets were mixed with 2 ml of lysis buffer containing 20 mM HEPES [pH 7.8], 10 mM potassium chloride (KCl), 1 mM EDTA, 1 mM dithiothreitol (DTT), 1 mM phenylmethanesulfonyl fluoride (PMSF), and 1% Triton X-100 (all components from Merck). The mixture was incubated on ice for 5 min, after which the parasite nuclei were isolated using a modified protocol from Voss and colleagues [26]. Nuclear proteins were extracted using a Topoisomerase Type IIα Nuclear Extraction/Assay Kit (TopoGen Inc., Colorado, USA). The concentration of nuclear proteins was measured by a Bradford Assay (BioRAD Inc., California, USA) [27]. The solution used for the *P*. *falciparum* topoisomerase II (topo II) activity assay was prepared from 10 μg of nuclear proteins, 100 ng of kinetoplast DNA, 1× TopoII reaction buffer, and the test compounds (ICL-M or pyronaridine) at a 100 μM final concentration. The reaction was incubated for 30 min at 37 °C. After incubation, each sample was mixed with Tris-Borate-EDTA (TBE) buffer and GelRed solution (Biotium Inc., California, USA). The topo II reaction samples were loaded into a 1% agarose gel and subjected to electrophoresis, after which the gel was visualized by Gel Documentary (BioRAD Inc.).

### 5. Time-dependent effect of antimalarial activity

Synchronized trophozoite stage *P*. *falciparum* was prepared at 2% parasitemia and 2% hematocrit. The culture was separated into three groups: untreated, <0.01% DMSO-treated, and 250 nM of ICL-M (IC_90_)-treated parasites. The cultures were started using 5 ml, and 400-μl samples were taken at each timepoint. After 24 h, the infected RBCs were washed three times with RPMI 1640 and resuspended in 400 μl of culture medium. A 100-μl aliquot of each sample was taken and used for continued culture in 96-well plates, individually (in triplicate). Surviving parasites were quantified by [^3^H]hypoxanthine labelling. Parasitic growth was determined by measurement of ionizing radiation.

### 6. Protein preparation

The <0.01% DMSO- and 250 nM of ICL-M (IC_90_)-treated trophozoite stage parasites were cultured for 4 h (in three biological replicates). Total RBCs were collected, washed with RPMI 1640, and centrifuged at 500 ×g at room temperature for 5 min. Parasites were released from the RBCs by the addition of 0.15% saponin in PBS buffer followed by an incubation at 4 °C for 5 min. Free parasites were washed three times with ice-cold PBS, then centrifuged at 2,500 ×g at 4 °C for 10 min. The resulting parasite pellets were kept at −80 °C until use.

### 7. Sodium dodecyl sulfate polyacrylamide gel electrophoresis (SDS-PAGE)

Parasite proteins were extracted by adding lysis buffer (1% SDS, 1% Triton-X, and 0.5% NaCl [all components from Merck]) and rotated at 4 °C for 1 h. The lysate was centrifuged at 10,000 ×*g* at 4 °C for 10 min, after which the resulting supernatant was collected. The protein concentration of each sample was measured using a Bradford assay (BioRAD Inc.) [27]. Proteins (30 μg per sample) were separated by 12% SDS-PAGE. The gel was stained by Coomassie blue R (BioRAD Inc.) and de-stained in 45% methanol (Merck) and 10% acetic acid (Merck). Each lane was sliced into 16 pieces and subjected to tryptic digestion.

### 8. In-gel digestion

Gel pieces were de-stained with 50% acetonitrile in 50 mM ammonium bicarbonate (Merck). The disulfide bonds in the proteins were reduced by treatment with 4 mM DTT in 50 mM ammonium bicarbonate at 60 °C for 15 min. Subsequently, the proteins were alkylated by incubation with 250 mM iodoacetamide at room temperature in the dark for 30 min. The reaction was quenched by 4 mM DTT in 50 mM ammonium bicarbonate for 5 min at room temperature. All solutions were removed, and the gel pieces were dehydrated by acetonitrile. The gel pieces were then air dried and resuspended in 0.1 μg/μl of Trypsin Proteomics Grade (Sigma Aldrich, Missouri, USA) in 50 mM ammonium bicarbonate. The digestion was performed overnight at 37 °C. The digested peptides were extracted by acetonitrile and dried using a centrifugal evaporator.

### 9. Mass spectrometry

Peptide mixtures were resuspended in 0.1% formic acid. Each sample was injected into the UltiMate 3000 nano-liquid chromatography (nano-LC) system (Dionex, Surrey, UK). Peptide separation was performed using a C18 column at a flow rate of 300 nL/min. Mobile phase A was 0.1% formic acid in water. Mobile phase B was 80% acetonitrile in 0.1% formic acid. The elution occurred during the 30-min gradient from 4% mobile phase B to 50% mobile phase A and infused to a micrOTOF-Q (Bruker Daltonics, Bremen, Germany). The mass spectra from the mass spectrometry (MS) and tandem mass spectrometry (MS/MS) covered mass ranges of m/z 400–2000 and m/z 50–1500, respectively. A mascot generic file (.mgf) was generated using DataAnalysis 3.4 version software. Mascot daemon version 2.3.2 (Matrix Science, London, UK) was used to merge the .mgf files and identify the proteins. Identification and quantification of the proteins were performed against a NCBInr database (24 October 2018) specific to *P*. *falciparum* 3D7. The protein abundance was determined by a peptide count analysis using the emPAI value. Three biological replications were performed. Differential expression in at least in two of the biological replicates was reported as protein alteration during ICL-M treatment. The UniProt database (www.uniprot.org) was used for indicating gene ontology and protein localization. STRINGS software (https://string-db.org/) was used to analyze protein–protein interactions [28].

### 10. Transmission electron microscopy (TEM)

RBCs infected with the trophozoite stage of *P*. *falciparum* were treated with <0.01% DMSO (buffer control) and 250 nM of ICL-M (IC_90_) for 4 h. The infected RBCs were then centrifuged at 500 ×g for 5 min, and the resulting pellets were washed with RPMI 1640. The sample preparation protocol used for TEM analysis was modified from that described by Ampawong and colleagues [29]. Infected RBCs were fixed overnight by 2.5% glutaraldehyde in 0.1 M sucrose phosphate buffer (SPB) at 4 °C, then washed three times with 0.1 M SPB. The cells were incubated in 1% osmium tetroxide (Electron Microscopy Sciences [EMS], Pennsylvania, USA) in 0.1 M SPB for 1 h. Afterwards, the cells were washed three times with 0.1 M SPB, dehydrated with ethanol at room temperature, and infiltrated by a series of LR white resin (EMS). Finally, the cells were embedded with 100% LR white resin in a capsule overnight at 65 °C. Each sample was cut into thin sections and stained with 2% uranyl acetate (Sigma Aldrich, Missouri, USA) for 1 min followed by staining with lead citrate for 3 min, then washed with water. The samples were examined by a transmission electron microscope (HT7700–6610LV, Hitachi, Japan).

## Results

### 1. The activity of ICL-M against *P*. *falciparum* 3D7

**Fig 2:**
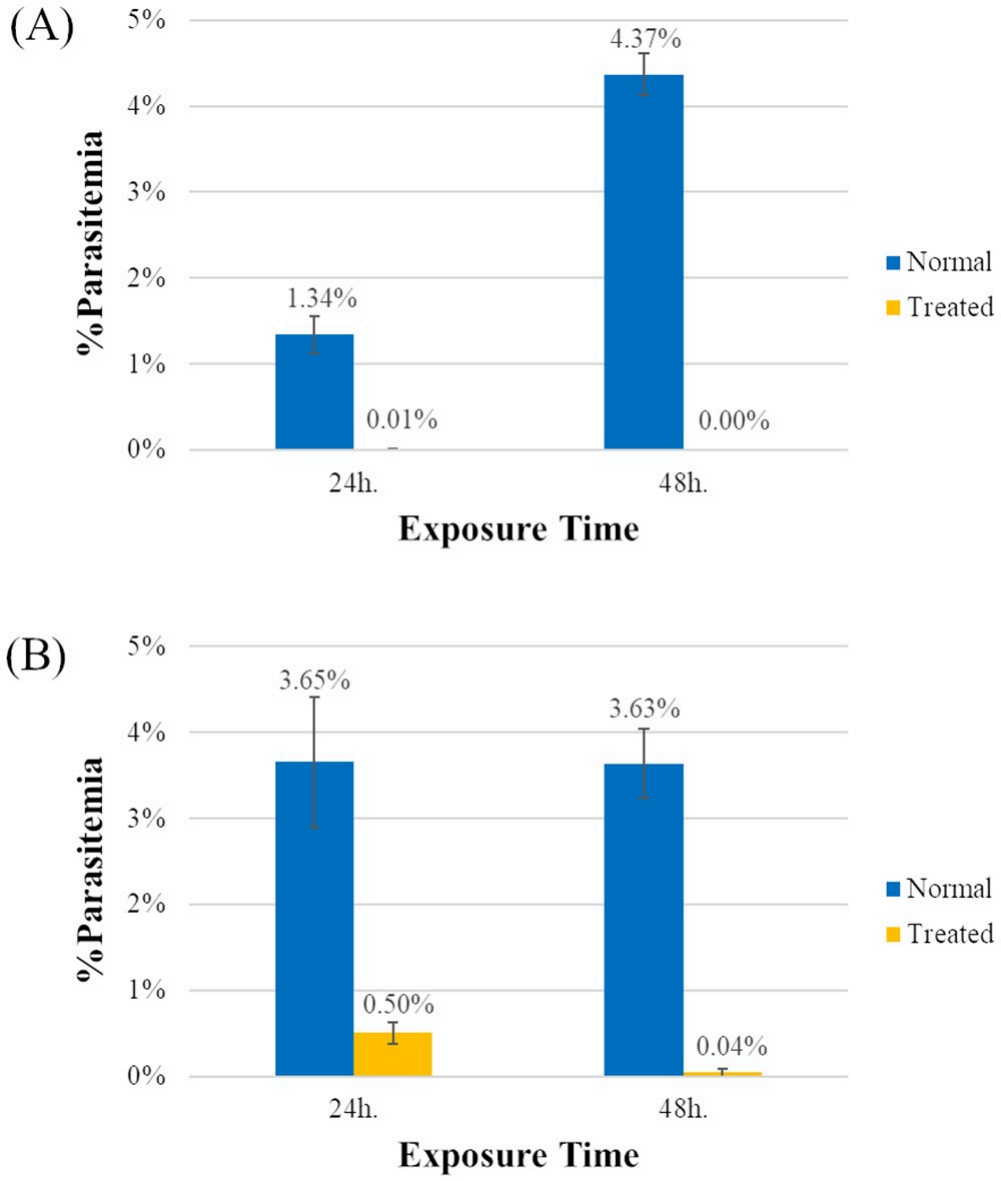
Stage-specific antimalarial activity of ICL-M. Synchronized *P*. *falciparum* in the ring or trophozoite stages were exposed to treatment with ICL-M at 3× IC_50_ for 24 or 48 h. (A–B) Antimalarial activity on the ring stage (A) and the trophozoite stage (B).

Antimalarial assays for ICL-M were performed using *P*. *falciparum* 3D7. The IC_50_ and IC_90_ of ICL-M were found to be 148.31 ± 22.87 and 243.39 ± 17.99 nM, respectively (Table 1). To assess the stage-specific antimalarial properties of ICL-M, the ring and trophozoite stages of *P*. *falciparum* 3D7 were each incubated with ICL-M. After 24 h of treatment, 99% of the ring-stage parasites were killed, and the total population was killed after 48 h of incubation (Fig 2). In contrast, ICL-M-treated trophozoite-stage parasites displayed 86% and 99% growth inhibition after 24 h and 48 h of incubation, respectively. Thus, ICL-M has the ability to kill both the ring and trophozoite stages of *P*. *falciparum*, but the trophozoite stage is slightly less sensitive to treatment with ICL-M compared with the early stage.

**Table 1:** Antimalarial activity of ICL-M against *P*. *falciparum* 3D7.

Compound	IC_50_	IC_90_
ICL-M	148.31 ± 22.87	243.39 ± 17.99

Mean (nM) ± SD

### 2. Inhibition of *P*. *falciparum* topoisomerase II activity

**Fig 3:**
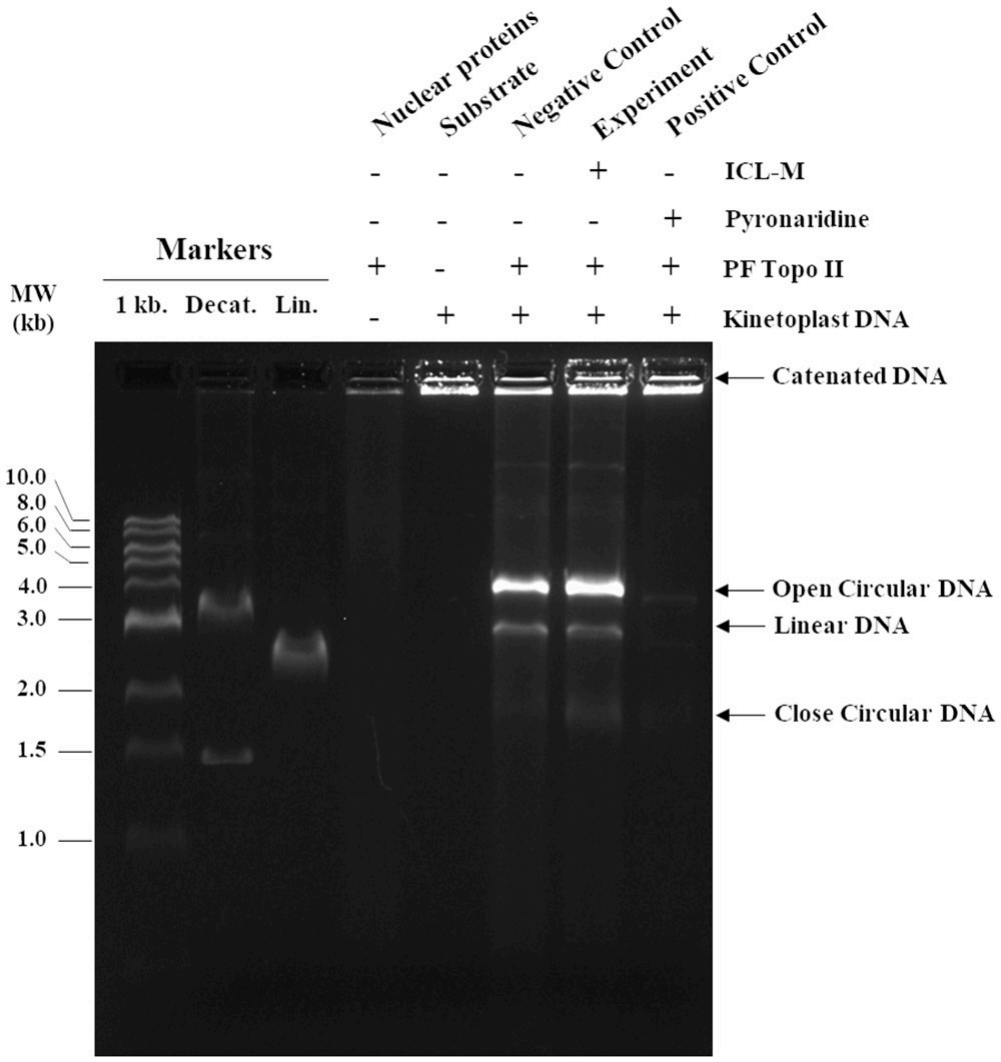
Effect of ICL-M on *P*. *falciparum* 3D7 topoisomerase II activity. Each reaction contained *P*. *falciparum* 3D7 topoisomerase II, kinetoplast DNA (substrate), and pyronaridine (positive control), DMSO (negative control), or ICL-M (experimental reaction).

Isocryptolepine derivatives have been reported to target topoisomerase II (topo II) in several different types of cancer cells [8]. Here, we tested the inhibitory activity of ICL-M against *P*. *falciparum* topo II. Pyronaridine is an antimalarial drug with documented parasite topo II inhibiting activity, so it was used as a positive control in this experiment (Fig 3). Topo II can break catenated DNA into decatenated DNA. In parasites treated with the negative control (DMSO, compound solvent), *P*. *falciparum* topo II activity was observed as the cutting of substrate kinetoplast DNA (catenated DNA) into circular DNA (decatenated DNA). As expected, an inhibition of *P*. *falciparum* topo II activity was observed in the presence of the positive control pyronaridine. Specifically, the resulting band of circular DNA was faint compared with that of the negative control-treated sample. When *P*. *falciparum* parasites were treated with ICL-M, a band of circular DNA with an intensity similar to that of the negative control was observed, suggesting that ICL-M treatment did not inhibit the activity of *P*. *falciparum* topo II in this assay.

### 3. Time course of *P*. *falciparum* growth rate inhibition by ICL-M

**Fig 4:**
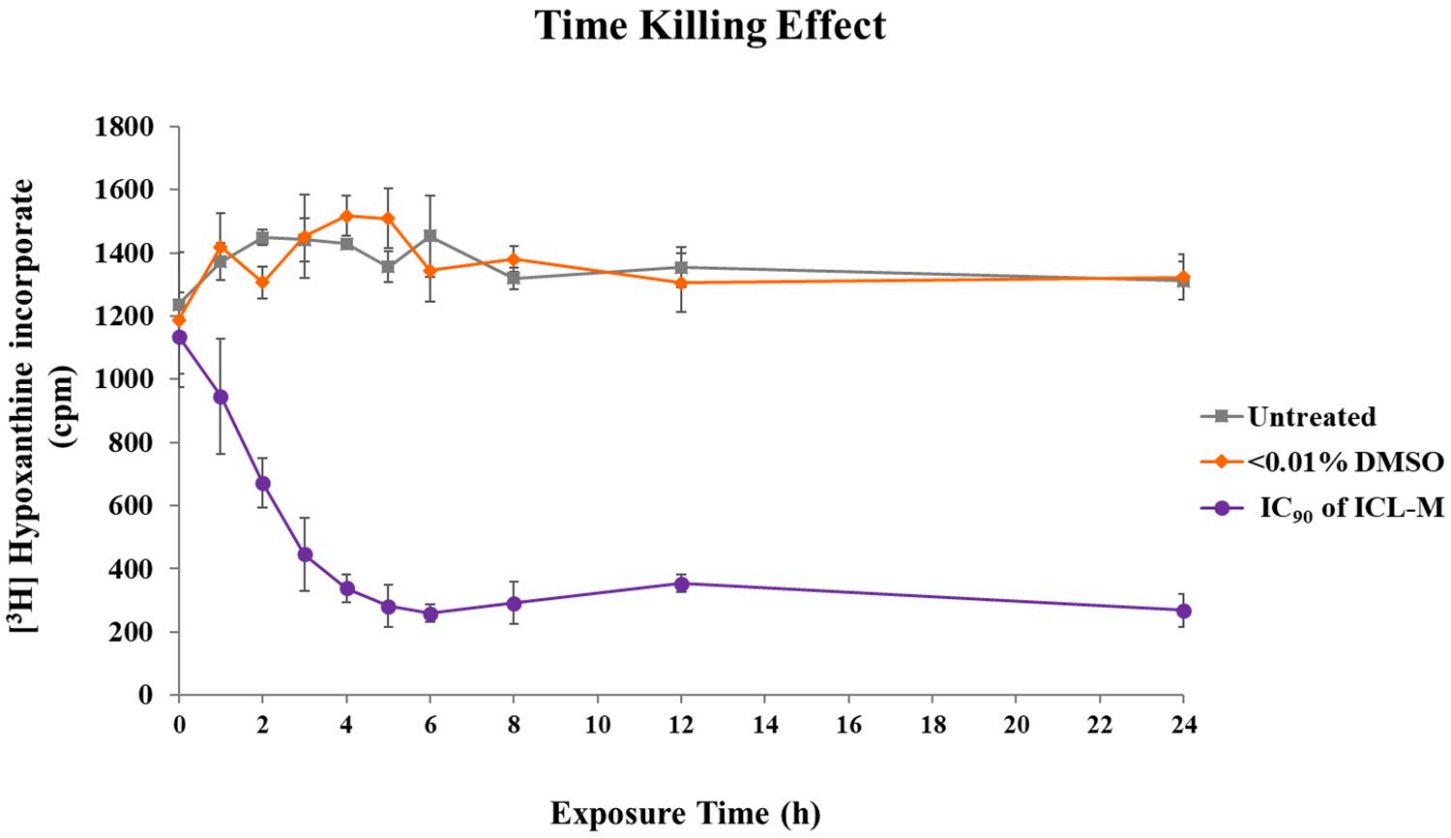
Time-dependent effects of treatment with ICL-M at IC_90_ on *P*. *falciparum* 3D7. The untreated, <0.01% DMSO-treated (solvent control), and 250 nM ICL-M (IC90)-treated groups were collected at different timepoints and assayed the growth of the parasite by [3H] hypoxanthine method.

To assess the growth rate inhibition against *P*. *falciparum* by ICL-M, three groups of trophozoite stage *P*. *falciparum* were prepared: the untreated, <0.01% DMSO-treated (solvent control), and 250 nM ICL-M (IC_90_)-treated groups. For the growth rate assay, a [^3^H] hypoxanthine supplement in the parasite culture was used to measure the growth of the parasite. The amount of parasite growth at each timepoint is shown in Fig 4. The growth rate of the *P*. *falciparum* in the <0.01% DMSO-treated group was similar to that of the untreated group, which suggests that the presence of the DMSO solvent did not affect the parasite growth rate. In contrast, the ICL-M treatment (IC_90_) initially caused detectable interference with parasite growth after 1 h of treatment and gradually reached the maximum inhibition rate after 4 h of exposure, maintaining this rate until at least 24 h of exposure.

### 4. Proteomic profile

**Fig 5:**
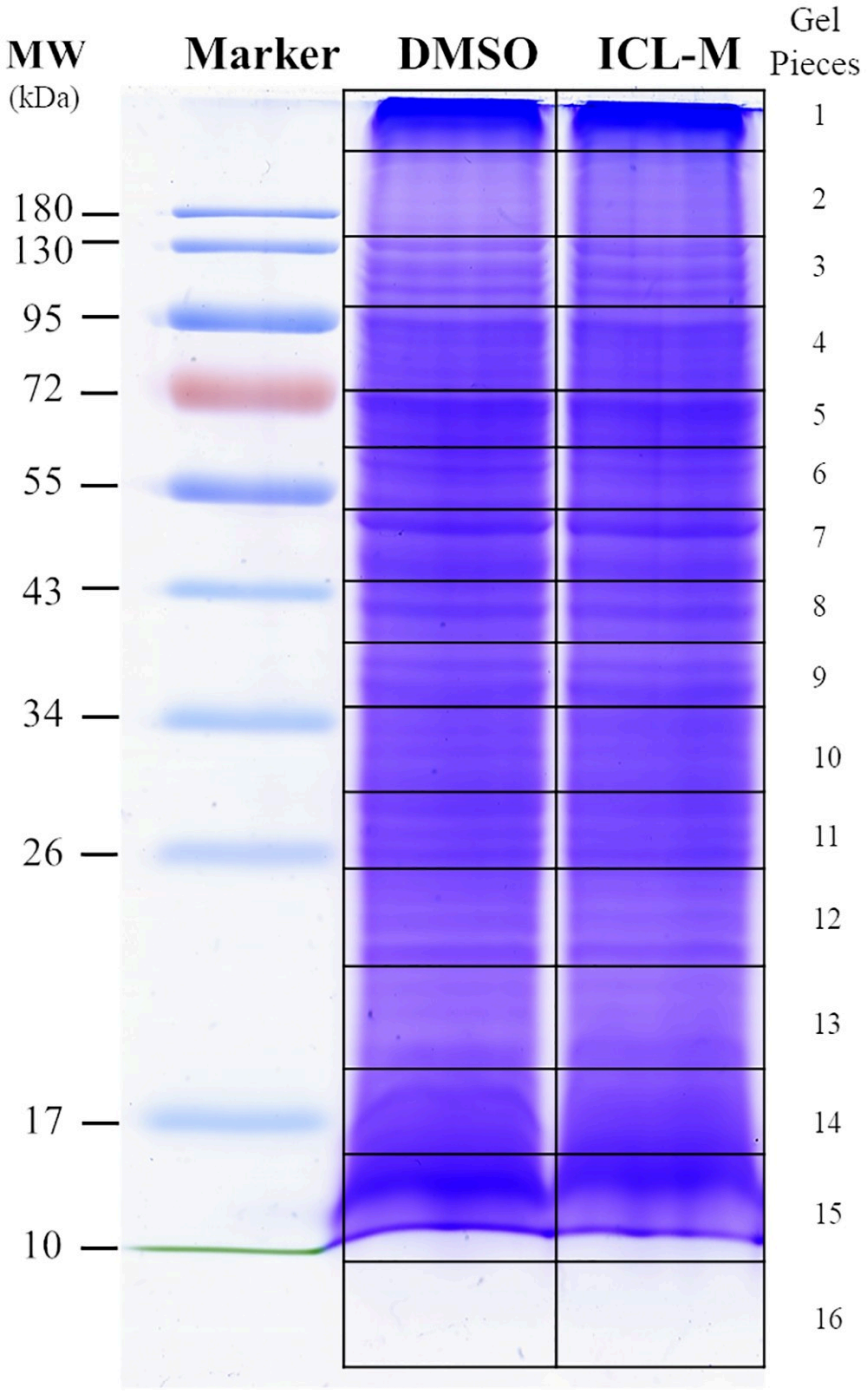
Coomassie blue-stained gel of DMSO- or ICL-M-treated *P*. *falciparum* 3D7 proteins. *P*. *falciparum* 3D7 parasites were exposed to DMSO (<0.01%) or ICL-M (IC_90_) for 4 h. Each gel lane was cut into 16 pieces for in-gel tryptic digestion.

**Fig 6:**
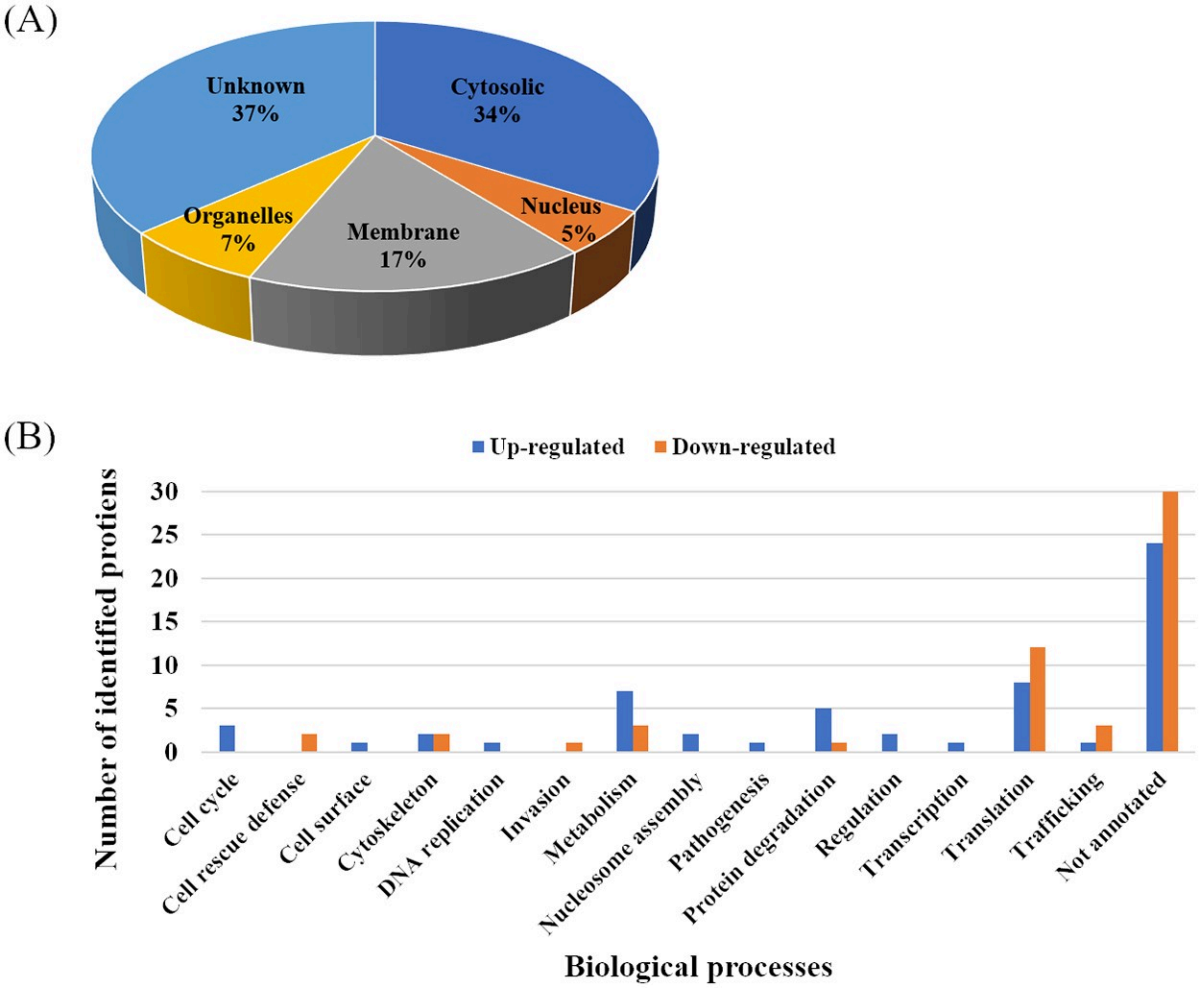
Gene ontology of differential *Plasmodium falciparum* proteins after ICL-M treatment. (A) Protein localization (B) Biological process classification. Blue bar: number of upregulated proteins, Orange bar: number of downregulated proteins.

**Fig 7:**
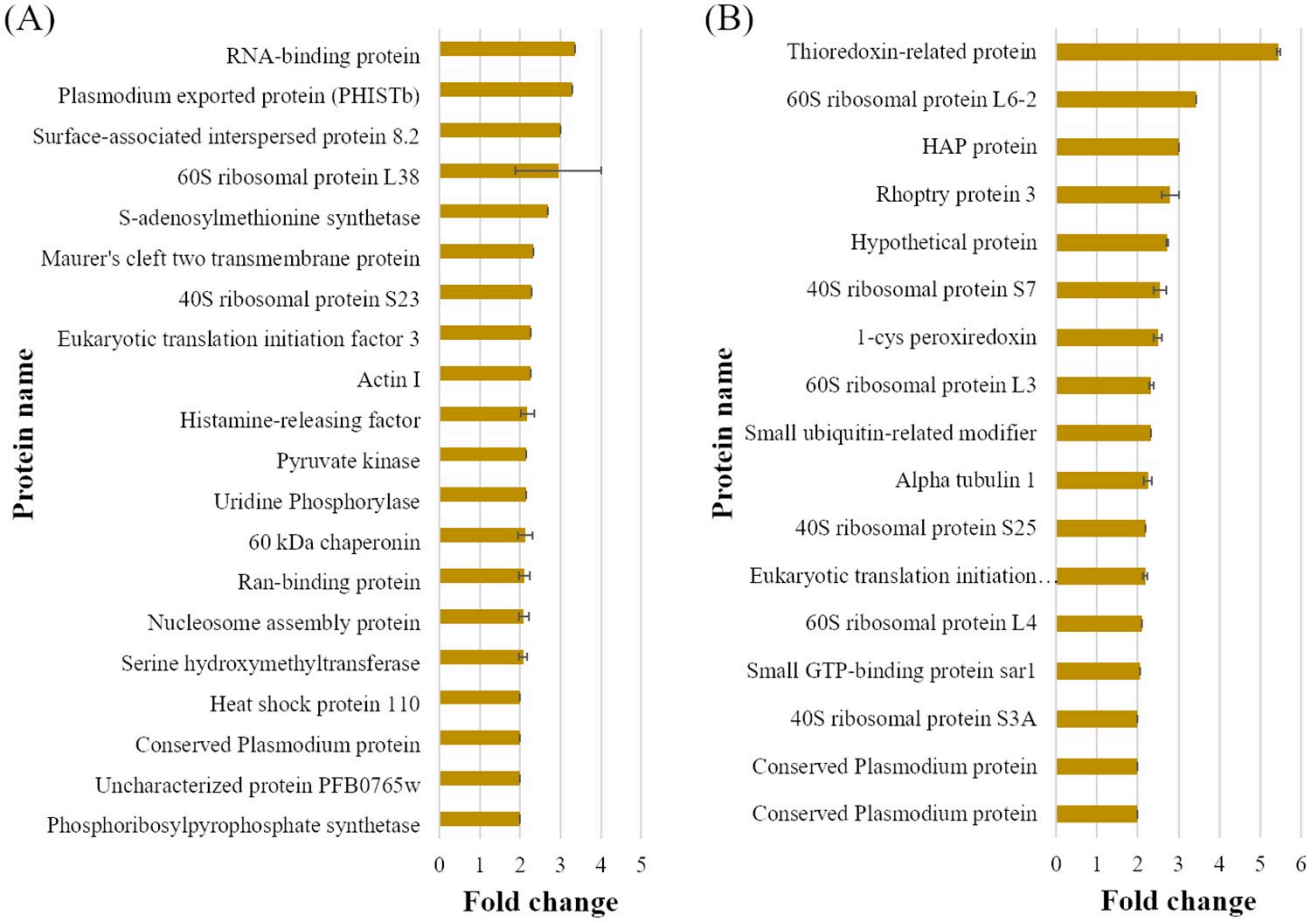
The most upregulated and downregulated proteins effect by ICL-M treatment ranked by emPAI values. (A) Upregulated proteins after ICL-M treatment. (B) Downregulated proteins after ICL-M treatment.

For studying differential protein expression, a proteomic approach was applied to *P*. *falciparum* exposed to ICL-M. Two groups of trophozoite stage *P*. *falciparum* parasites were prepared: <0.01% DMSO-treated (solvent control) and 250 nM ICL-M (IC_90_)-treated groups. Proteins were extracted from each experimental group after 4 h of treatment (the minimum exposure time needed to induce irreversible parasite death). The proteins were then separated by 1D-SDS-PAGE, and each lane was cut into 16 pieces (Fig 5) prior to in-gel trypsin digestion. The digested peptides were identified by LC-MS/MS, and each protein was quantified using a label-free (emPAI) spectral counting technique. A total of 668 unique proteins were identified (Table 2), with 511 and 514 proteins in the control and ICL-M-treated groups, respectively. Among these, 112 proteins were differentially expressed in the ICL-M-treated group when compared with the control group; there were 58 upregulated proteins (Table 3) and 54 downregulated proteins (Table 4) in the ICL-M-treated group. According to the Uniprot database, the differentially expressed proteins were localized to the cytosol (34%), membrane (17%), nucleus (5%), other organelles (7%), or had an unknown cellular location (37%) (Fig 6A). The upregulated proteins were specifically involved in several biological processes, including the cell cycle, cell surface, cytoskeleton, DNA replication, metabolism, nucleosome assembly, pathogenesis, protein degradation, regulation, transcription, translation, and trafficking. The downregulated proteins were linked to seven biological processes: cell rescue defense, cytoskeleton, invasion, metabolism, protein degradation, translation, and trafficking (Fig 6B). The most upregulated and downregulated proteins with their fold changes calculated by the semi-quantitative data were demonstrated in Fig 7.

**Table 2:** Protein detection of *P*. *falciparum* 3D7 after 4 h of treatment with IC_90_ ICL-M.

Compound	All Detection	All Change	Up	Down
DMSO	511	-	-	-
ICL-M	514	112	58	54

Total detected proteins = 668

**Table 3:** Upregulated proteins in ICL-M-treated *P*. *falciparum* 3D7.

Biological Process	NCBI Accession Number	Protein Description	Protein Mass	PI	Protein Score	Percent Coverage	Localization
**Cell Cycle**	gi|23504595|	Histamine-releasing factor	19967	4.58	210	23.4	Cytoplasm
	gi|46362290|	DNA repair protein RAD50, putative	267786	8.78	39	17.6	Nucleus
	gi|356624409|	Chain A, Translationally-controlled Tumor Protein Homolog	21594	4.9	26	19.7	Cytoplasm
**Cell Surface**	gi|23505252|	Cytoadherence linked asexual protein 9	160313	8.88	42	17.1	Membrane
**Cytoskeleton**	gi|74929507|	Actin I	41844	5.21	78	24.7	Cytoplasm
	gi|124806724|	GAS8-like protein, putative	54772	7.98	42	8.9	Golgi Apparatus
**DNA Replication**	gi|17148533|	Ran-binding protein	33176	4.92	57	16.4	Cytoplasm
**Metabolism**	gi|46361220|	Pyruvate kinase	55625	7.5	405	44.8	Cytoplasm
	gi|10129955|	S-adenosylmethionine synthetase	44816	6.28	250	31.1	Unknown
	gi|47169189|	Chain A, Uridine Phosphorylase, Putative	27745	6.32	188	44.3	Unknown
	gi|23615408|	Phosphoribosylpyrophosphate synthetase	49352	9.39	151	26.3	Unknown
	gi|322812543|	Chain A, Glucose-6-phosphate isomerase	69517	6.9	144	24.3	Unknown
	gi|124806534|	Serine hydroxymethyltransferase	49749	8.29	103	31	Cytoplasm
	gi|124801366|	ATP synthase F1, alpha subunit	61731	8.72	33	19.6	Mitochondria
**Nucleosome Assembly**	gi|23505090|	Nucleosome assembly protein	31807	4.17	131	24.2	Cytoplasm
	gi|124806302|	WD repeat-containing protein, putative	378674	9.02	45	12.8	Nucleus
**Pathogenesis**	gi|258597440|	Antigen 332, DBL-like protein	688870	3.86	45	5.2	Membrane
**Protein degradation**	gi|124810483|	Proteasome subunit alpha type-1, putative	28819	5.51	192	24	Cytoplasm
	gi|23615667|	Proteasome subunit alpha type-4, putative	27930	5.85	47	20.3	Cytoplasm
	gi|23615691|	Ubiquitin-conjugating enzyme, putative	22869	5.32	40	17.3	Cytoplasm
	gi|258597535|	60 kDa chaperonin	81434	4.97	30	22.8	Apicoplast
	gi|23505203|	Peptidyl-prolyl cis-trans isomerase	72507	9.11	25	15.8	Nucleus
**Regulation**	gi|23504638|	Phosphatidylinositol 3-kinase	255758	9.27	16	11.2	Food Vacuole
	gi|23498743|	tRNA m5C-methyltransferase, putative	141140	6.35	30	17	Unknown
**Transcription**	gi|124802200|	PRE-binding protein	131545	9.19	30	14.3	Nucleus
**Translation**	gi|124804373|	60S ribosomal protein L38	10307	10.71	100	58.6	Cytoplasm
	gi|7672213|	Eukaryotic translation initiation factor 3 subunit K, putative	28017	5.61	92	31.1	Cytoplasm
	gi|23615551|	60S ribosomal protein L18, putative	21733	10.62	88	45.1	Cytoplasm
	gi|23499061|	RNA-binding protein, putative	32421	9.11	81	15.4	Unknown
	gi|4493905|	40S ribosomal protein S23, putative	16120	10.83	66	33.8	Cytoplasm
	gi|46361134|	60S ribosomal protein L27a, putative	16712	10.54	59	31.8	Cytoplasm
	gi|124802168|	Eukaryotic translation initiation factor 2 subunit beta, putative	25306	9.23	42	43.2	Cytoplasm
	gi|124808549|	NOT family protein, putative	519499	6.88	27	11.8	Cytoplasm
**Trafficking**	gi|124805983|	Clathrin heavy chain, putative	232803	6	38	12.3	Cytoplasm
**Unknown**	gi|225632011|	Heat shock protein 110, putative	108119	5.5	95	16.8	Apicoplast
	gi|23498308|	Plasmodium exported protein (PHISTb), unknown function	35939	8.75	88	32	Unknown
	gi|8439487|	hypothetical protein, partial	23455	5.87	61	14.6	Membrane
	gi|23505032|	Elongation factor 1-beta	32007	4.94	50	25.4	Unknown
	gi|23504974|	ATP-dependent protease ATPase subunit ClpY	106396	8.42	48	17.4	Cytoplasm
	gi|3694805|	Cytoadherence linked asexual protein, partial	160994	8.98	42	20.1	Membrane
	gi|225631696|	Conserved Plasmodium protein, unknown function	1116481	9.37	39	11.1	Membrane
	gi|23498992|	Surface-associated interspersed protein 8.2 (SURFIN 8.2)	248324	5.35	38	17.7	Membrane
	gi|124808162|	Conserved Plasmodium protein, unknown function	240981	9.51	36	13	Unknown
	gi|23505262|	Plasmodium exported protein (PHISTc), unknown function	45472	9.71	33	16.4	Membrane
	gi|23505053|	Conserved Plasmodium protein, unknown function	85353	9.1	32	12.3	Unknown
	gi|258597726|	Conserved Plasmodium protein, unknown function	248348	9.01	30	10.2	Unknown
	gi|258549210|	Conserved Plasmodium protein, unknown function	30341	7.64	29	18.6	Unknown
	gi|124804341|	Parasitophorous vacuolar protein 1	51919	4.97	29	24.3	Cytoplasm
	gi|23499096|	Conserved Plasmodium protein, unknown function	170170	6.4	27	12.1	Unknown
	gi|46362309|	Conserved Plasmodium protein, unknown function	324818	6.62	25	10.5	Unknown
	gi|296005130|	Pfmc-2TM Maurer~s cleft two transmembrane protein	27380	9.4	25	31.2	Membrane
	gi|74862993|	RecName: Full = Uncharacterized protein PFB0765w	166903	6.19	25	17.1	Unknown
	gi|258597812|	Conserved Plasmodium protein, unknown function	70994	9.15	24	19	Unknown
	gi|46362277|	Conserved Plasmodium protein, unknown function	334204	8.49	22	18.4	Unknown
	gi|23498914|	Mitochondrial import inner membrane translocase subunit TIM14, putative	13044	10.09	21	22.6	Unknown
	gi|23615369|	Conserved Plasmodium membrane protein, unknown function	404731	8.76	21	14.6	Membrane
	gi|23498252|	Regulator of chromosome condensation, putative	236211	9.23	21	9.2	Unknown
	gi|23505265|	Plasmodium exported protein, unknown function	31267	9.69	20	8.9	Membrane

**Table 4:** Downregulated proteins in ICL-M-treated *P*. *falciparum* 3D7.

Biological Process	NCBI Accession Number	Protein Description	Protein Mass	PI	Protein Score	Percent Coverage	Localization
**Cell Rescue Defence**	gi|23499261|	1-cys peroxiredoxin	25148	6.31	155	47.3	Cytoplasm
	gi|23615654|	Thioredoxin-related protein, putative	23972	9.44	134	26.9	Membrane
**Cytoskeleton**	gi|23504938|	Alpha tubulin 1	50264	4.93	234	23.8	Cytoplasm
	gi|23504954|	Dynein heavy chain, putative	720134	6.18	32	10.6	Cytoplasm
**Invasion**	gi|23504955|	High molecular weight rhoptry protein 3	104789	6.25	150	21.4	Rhoptry
**Metabolism**	gi|237640532|	HAP protein	37376	4.97	141	32.2	Food Vacuole
	gi|46361188|	Hexokinase	55226	6.72	53	31.2	Cytoplasm
	gi|23504543|	Small ubiquitin-related modifier	11053	4.74	52	38	Nucleus
**Protein degradation**	gi|124804234|	Autophagy-related protein 7, putative	156530	6.05	29	18.2	Cytoplasm
**Translation**	gi|23504556|	60S ribosomal protein L4	46183	10.5	268	34.3	Cytoplasm
	gi|23615172|	40S ribosomal protein S7, putative	22467	9.81	266	41.8	Cytoplasm
	gi|23615526|	60S ribosomal protein L6-2, putative	25516	10.1	222	26.7	Cytoplasm
	gi|225631740|	60S ribosomal protein L19	21566	11.32	168	14.3	Cytoplasm
	gi|23499154|	60S ribosomal protein L13-2, putative	25425	10.78	123	30.2	Cytoplasm
	gi|124802670|	60S ribosomal protein L3	44193	10.21	89	29.3	Cytoplasm
	gi|4494003|	40S ribosomal protein S3A, putative	30028	9.8	55	33.2	Cytoplasm
	gi|23504862|	Eukaryotic translation initiation factor 3 subunit E, putative	61379	7.08	49	23.4	Cytoplasm
	gi|258597702|	40S ribosomal protein S25	11656	10.12	44	55.2	Cytoplasm
	gi|23498839|	Eukaryotic translation initiation factor 3 subunit I, putative	37261	6.43	36	19.6	Cytoplasm
	gi|124804772|	60S ribosomal protein L35ae, putative	16255	10.55	25	26.4	Cytoplasm
	gi|23498787|	60S ribosomal protein L34	17340	10.77	24	10.7	Cytoplasm
**Trafficking**	gi|23498233|	Small GTP-binding protein sar1	22006	6.75	72	31.3	Endoplasmic Reticulum
	gi|13375179|	Putative GTPase	22872	6.18	47	16	Membrane
	gi|23615179|	Sodium/hydrogen exchanger, Na+, H+ antiporter	225940	8.68	29	11.4	Membrane
**Unknown**	gi|8247298|	Hypothetical protein, partial	4755	9.23	159	60	Unknown
	gi|124801947|	RNA-binding protein, putative	30033	10.07	55	35.1	Unknown
	gi|23504857|	Hsc70-interacting protein	51092	4.67	45	14.6	Unknown
	gi|225632182|	Conserved Plasmodium protein, unknown function	126994	5.28	39	12	Unknown
	gi|23498915|	Conserved Plasmodium protein, unknown function	36570	8.79	39	22	Unknown
	gi|225632293|	Plasmodium exported protein, unknown function	36390	5.74	35	22.2	Unknown
	gi|14530178|	Krueppel-like protein	151550	7.89	33	11.5	Membrane
	gi|23498950|	Zinc finger, C3HC4 type, putative	253816	8.32	32	15.5	Unknown
	gi|23615182|	Conserved Plasmodium protein, unknown function	1111079	9.12	30	15.6	Membrane
	gi|23504877|	Conserved Plasmodium protein, unknown function	225390	6.01	30	16	Unknown
	gi|3649757|	Conserved Plasmodium protein, unknown function	202018	8.3	29	16.5	Cytoplasm
	gi|225631936|	MORN repeat protein, putative	519893	9.15	29	10.3	Membrane
	gi|124808756|	Conserved Plasmodium protein, unknown function	192484	9.67	29	16.8	Membrane
	gi|124806636|	Conserved Plasmodium protein, unknown function	212627	4.96	29	12.8	Unknown
	gi|23504575|	Conserved Plasmodium protein, unknown function	369953	5.29	29	9	Unknown
	gi|124809084|	Conserved Plasmodium protein, unknown function	152167	8.42	29	12.9	Unknown
	gi|124805631|	Conserved Plasmodium protein, unknown function	208541	8.3	29	15.2	Unknown
	gi|124802600|	Conserved Plasmodium protein, unknown function	190279	8.29	29	10.9	Unknown
	gi|124804432|	Conserved Plasmodium protein, unknown function	133324	5.18	29	17.4	Unknown
	gi|124804384|	Conserved Plasmodium protein, unknown function	118257	8.84	29	14.6	Unknown
	gi|225632254|	rRNA-processing protein FCF1, putative	23145	9.63	29	23.2	Nucleus
	gi|23505139|	RNA-binding protein, putative	22965	9.51	27	20.3	Unknown
	gi|124808373|	Conserved Plasmodium protein, unknown function	390446	9.23	25	11.7	Unknown
	gi|23615433|	Conserved Plasmodium protein, unknown function	319935	7.32	25	15.5	Unknown
	gi|23498752|	Conserved Plasmodium protein, unknown function	118270	8.1	23	9	Unknown
	gi|225631798|	Conserved protein, unknown function	296598	9.02	22	15.4	Membrane
	gi|124804419|	Conserved Plasmodium protein, unknown function	330099	6.16	21	12.1	Unknown
	gi|225631940|	Conserved Plasmodium protein, unknown function	737235	9.13	21	11.3	Membrane
	gi|124804142|	Leucine-rich repeat protein	93275	8.97	18	13.9	Unknown
	gi|225631849|	Conserved Plasmodium protein, unknown function	102515	8.83	18	11.7	Unknown

**Fig 8:**
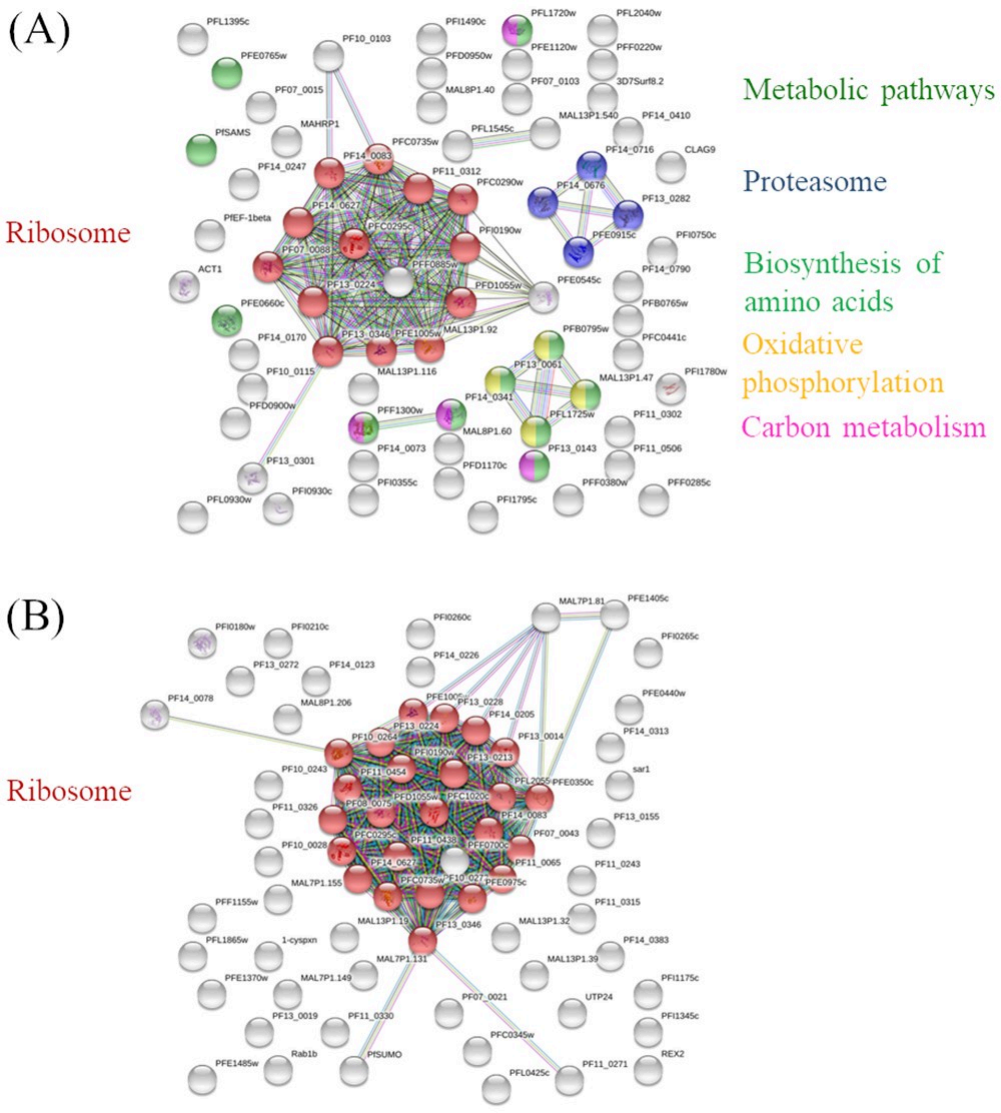
STRINGS protein–protein interaction analysis of differentially expressed proteins following ICL-M treatment. (A) Upregulated proteins after ICL-M treatment. (B) Downregulated proteins after ICL-M treatment. Red: Ribosome, Blue: Proteasome, Dark green: Metabolic pathways, Light green: Biosynthesis of amino acids, Yellow: Oxidative phosphorylation, and Pink: Carbon metabolism.

The predicted protein–protein interaction network identified the ribosome pathway as the major affected pathway in both the upregulated and downregulated proteins (Fig 8). In contrast, the proteasomal, amino acid biosynthesis, metabolic, oxidative phosphorylation, and carbon metabolism pathways were identified as minor pathways, and the proteins linked to them were detected in only the group of upregulated proteins.

### 5. Ultrastructural effect of ICL-M

**Fig 9:**
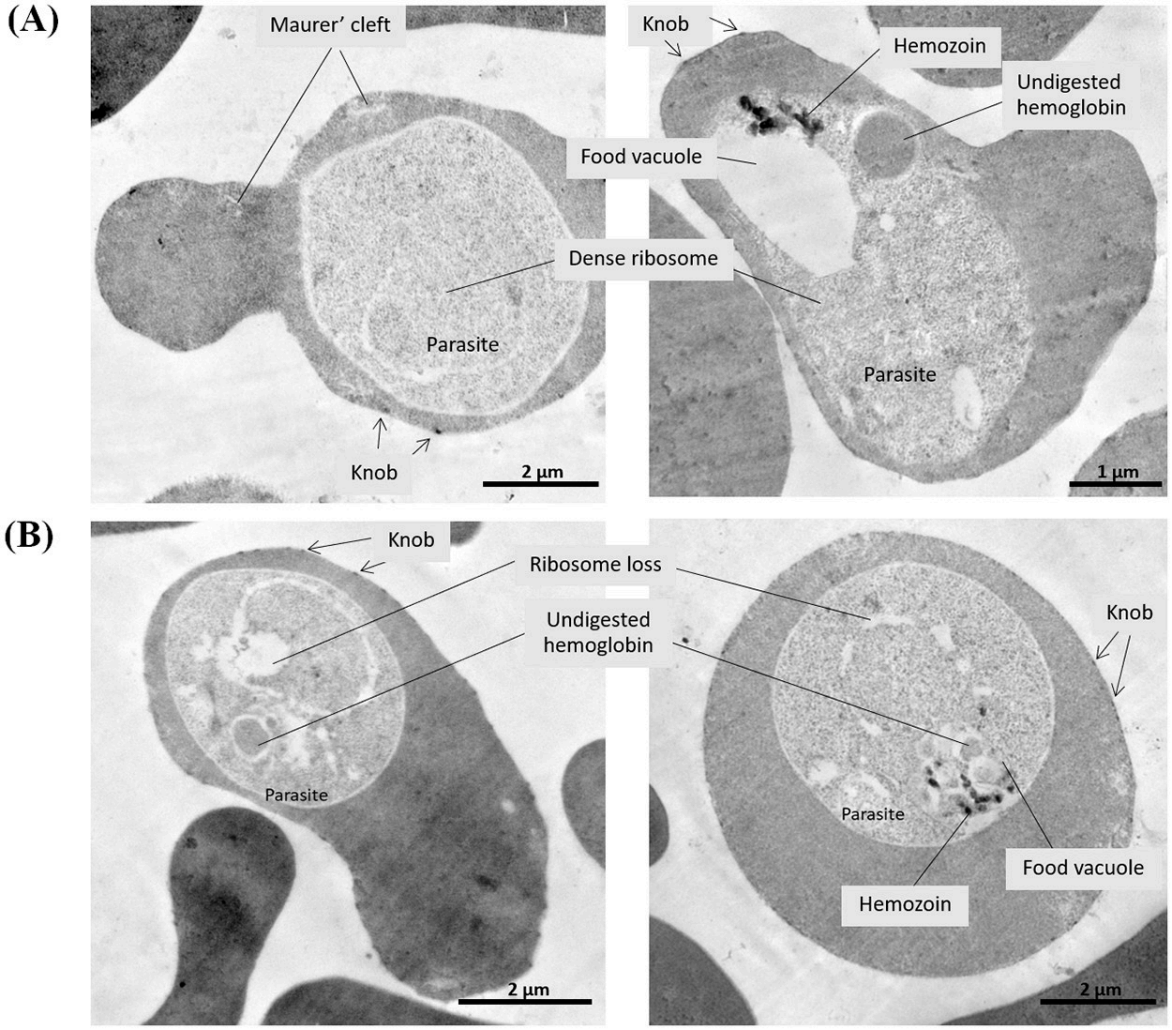
Transmitted electron micrograph of trophozoite stage *P*. *falciparum* 3D7 parasites after a 4-h compound exposure. (A) The control group treated with DMSO. (B) The experimental group treated with ICL-M.

To examine the effect of ICL-M treatment on parasite ultrastructure, the morphology of ICL-M-treated *P*. *falciparum* was investigated. Differences were observed between the trophozoite stage parasites within RBCs in the control culture (Fig 9A) and the ICL-M treatment (Fig 9B) groups. Numerous Maurer’s clefts and knobs were seen in the infected RBCs of both groups. Internally, both the control and ICL-M-treated parasites displayed normal food vacuoles with hemozoin, undigested hemoglobin, and dense ribosomes. Interestingly, the loss of ribosome area could be observed in the ICL-M-treated group.

## Discussion

The present study aimed to explore the molecular mechanisms of ICL-M. We studied the effects of ICL-M on *P*. *falciparum* by assessing its stage-specific activity, topo II activity inhibition, and time-dependent effect as well as by performing a proteomic analysis and a TEM morphology analysis. The stage-specific activity results indicate that ICL-M has antimalarial activity against both the ring and trophozoite stages of *P*. *falciparum*. This result corresponds to those of quinine-, artemisinin-, artesunate-, and chloroquine-treated parasites [30, 31]. After drug exposure, the ICL-M demonstrated a stronger inhibitory effect on the ring stage of *P*. *falciparum* than on the trophozoite stage, which is similar to the results of reports on chloroquine and artemisinin [30, 31]. In contrast, quinine and artesunate have stronger inhibitory effects on trophozoite stage parasites than they do on ring stage parasites. We also found that the trophozoite stage of *P*. *falciparum* was irreversibly damaged by ICL-M (IC_90_) treatment by 4 h after exposure. Thus, ICL-M causes irreversible damage faster than do either artemether (IC_90_) or lumefantrine (IC_90_), which display irreversible killing parasite at 5 h or 8 h after exposure, respectively [17].

In previous studies, several isocryptolepine derivatives have been tested for use in anti-cancer chemotherapy, where they demonstrated high efficiency supporting their further development as novel drugs. Indoloquinoline, which forms the core of ICL-M, has demonstrated activity against the human cancer topo II enzyme in melanoma, leukemia, and non-melanoma skin cancer [32–34]. In addition, the inhibition activity of another isocryptolepine derivative, 3-methoxy-11H-pyrido[3',4':4,5]pyrrolo[3,2-c]quinoline-1,4-dione, has been studied on mouse topo I and topo II [8]. Moreover, etoposide, a well-known anticancer targeting human topo IIα, induced the same inhibitory activity on *P*. *falciparum* 3D7 topo II [35]. These previous findings raised a concern that ICL-M might inhibit *P*. *falciparum* 3D7 topo II. However, our results indicate that ICL-M has no inhibitory activity against *P*. *falciparum* topo II, although indoloquinoline was reported to accumulate in the nucleus of *P*. *falciparum* [36]. In addition, many studies have provided evidence of isocryptolepine analogues interrupting the macromolecules that interfere with the replication and transcription processes in both eukaryotic and prokaryotic cells [37–39]. However, *P*. *falciparum* topo II might not be the direct target of this compound. The compound accumulation in nucleus may result from DNA intercalation observed in other indoloquinoline derivatives [37]. On the other hands, ICL-M may interact with other topoisomerases or other target proteins. To investigate whether ICL-M affects protein targets associated with antimalarial mechanisms, we applied a large-scale proteomics approach using mass spectrometry. The control and ICL-M-treated groups were identified 511 and 514 proteins, respectively. An proteomic study performed on *Plasmodium* using in-solution digestion by endoprotease Lys-C and trypsin could yield 714 protein identification in asexual blood stage [9]. The different number of protein identification may cause by the completeness of protease digestion to prepare peptide mixture for mass spectrometric analysis.

Fifty-eight *P*. *falciparum* nuclear proteins were identified in our study. Among these, six proteins showed differential expression; DNA repair protein RAD50 (RAD50), peptidyl-prolyl cis-trans isomerase (PPIase), PRE-binding protein (PREBP), and WD repeat-containing protein (WDR) were upregulated after ICL-M exposure, whereas rRNA-processing protein FCF1 (FCF1) and small ubiquitin-related modifier (SUMO) were downregulated. Interestingly, PPIase, PREBP, and SUMO have been reported as drug or vaccine targets in *P*. *falciparum* [40], [41], [42]. These mechanisms may be involved in the inhibition of *P*. *falciparum* by ICL-M. PPIase accelerates protein folding by catalyzing the cis–trans isomerization of peptide bonds that are N-terminal to proline residues in polypeptide chains. This enzyme has been reported as a target of several antimalarial drugs, such as cyclosporine A, FK506, and rapamycin [40]. PREBP is involved in DNA-binding transcription factor activity. This protein is expressed in the *Plasmodium* merozoite stage and has been reported as a target of monoclonal antibodies that inhibit parasite invasion [41]. In addition, PREBP has also been reported as a potential candidate for *P*. *falciparum* vaccine development [42]. SUMO plays an important role in the oxidative stress response during the *P*. *falciparum* intra-erythrocyte developmental cycle. It is a promising target for the development of *P*. *falciparum* sumoylation inhibitors and parasite replication [43]. The other three differentially expressed proteins detected in our study, RAD50, WDR, and FCF1, have not been reported to have an association with existing antimalarial drugs. Based on our results, it is possible that ICL-M might have some effects on parasite nuclear proteins as described above.

In addition to affecting nuclear proteins, one isocryptolepine derivative was reported to affect activity in the parasite food vacuole [44]. A number of studies suggested that quinoline antimalarial drugs, such as chloroquine, quinine, and mefloquine, could inhibit hemozoin formation in the food vacuole of parasites [45]. Since isocryptolepine also contains part of quinoline in its core structure, this drug has been investigated for its ability to inhibit heme detoxification. The result revealed that β-hematin detoxification was inhibited by 3 fold less isocryptolepine than chloroquine [4]. Therefore, isocryptolepine may contribute to other actions in the parasite food vacuole. Our proteomics data identified nine *P*. *falciparum* food vacuole proteins in our samples. Among these, histo-aspartic protease (HAP) protein was 3-fold downregulated and phosphatidylinositol 3-kinase (PI3K) was only identified in the treated parasite. HAP is a type of *P*. *falciparum* aspartic protease and plays a role in hemoglobin degradation. [46]. An alternative name for HAP is plasmepsin (PM) III. In *P*. *falciparum*, four PMs, specifically PM I, II, III, and IV, are key mediators for hemoglobin degradation [47]. All four of these PMs have received considerable attention as potential antimalarial drug targets. The absence of PM III led to the most significant hyper-susceptibility in response to antimalarial compounds such as chloroquine [48] and piperaquine [49]. In our study, PM III was downregulated under ICL-M treatment. The low level of HAP may play a role in increasing the susceptibility of parasites to ICL-M, thus improving the antimalarial efficiency of ICL-M. PI3K was up-regulated in *P*. *falciparum* parasites exposed to ICL-M. PI3K is involved in trafficking of host hemoglobin to the parasite food vacuole by endocytosis [50]. While, the downregulation of PM III may reduce the hemoglobin degradation. An upregulation of PI3K may be a compensatory effect to the increased hemoglobin uptake that occurs when hemoglobin digestion is altered.

The protein–protein interactions of all the differentially expressed proteins detected by our proteomics analysis were assessed using the STRING server. Following ICL-M treatment, the significantly enriched pathways included ribosomal, proteasomal, metabolic, amino acid biosynthesis, oxidative phosphorylation, and carbon metabolism pathways. Ribosomes are the major machinery in the translational process; thus, a mutation or alteration of ribosomal proteins leads to abnormal function in cell proliferation [51], [52], [53]. Several ribosomal proteins have been reported as drug targets. Tetracycline, an antibiotic, is able to bind to 70S ribosome and inhibit protein synthesis in bacteria [54]. For *P*. *falciparum*, cryogenic electron microscopy revealed that the ribosome GTPase-associated center reacts with mefloquine. Correspondingly, the inhibition of translational processes is one of modes of action for mefloquine [55]. Our proteomics data identified a total of 58 ribosomal proteins; four and ten proteins were upregulated and downregulated, respectively. Interestingly, 60S ribosomal protein L18 and 60S ribosomal protein L27a were both upregulated after ICL-M treatment. This result corresponds to a similar finding following artemisinin treatment of parasites [12]. ICL-M and artemisinin might share some modes of action relating to interference with protein synthesis.

The proteasomal pathway was significantly enriched following ICL-M exposure of *P*. *falciparum*. The proteasome is the major protein degradation regulatory network. When homeostasis between protein synthesis and degradation is unbalanced, cell survival is not possible [56]. In *Plasmodium*, proteasomal proteins have been suggested as potential antimalarial drug targets that could act to disrupt parasite protein homeostasis [57]. Our data identified a total of 11 proteasomal proteins, among which there were two upregulated after ICL-M treatment: proteasome subunit alpha type-1 and proteasome subunit alpha type-4. The differential expression of either of these two proteins has not been reported in studies of other antimalarial drugs. However, other proteins in the proteasome pathway, such as 20S proteasome beta subunit, proteasome regulatory subunit, and proteasome subunit beta type 1, were found to be upregulated after artemisinin treatment of parasites [12]. Additionally, the downregulation of 26S proteasome regulatory subunit was observed in doxycycline-treated parasites [13]. Based on our findings, creating an imbalance in *P*. *falciparum* protein homeostasis might be one mode of action for ICL-M.

The glycolytic metabolic pathway was also affected in *P*. *falciparum* after ICL-M exposure. Glycolysis is an important metabolic pathway that produces energy for the parasite. Several studies have explored the effect of antimalarial drugs in the expression of proteins involved in this pathway [17, 58] [13, 18]. Our study detected three proteins in this pathway that were differentially expressed in *P*. *falciparum* after ICL-M exposure: hexokinase, glucose-6-phosphate isomerase, and pyruvate kinase. Hexokinase and pyruvate kinase have also been reported to have altered expression levels in parasites following doxycycline treatment [13]. Given that ICL-M altered the expression level of proteins in the glycolysis pathway, ICL-M may influence parasite energy production.

In our electron microscopy analysis, we observed a clear loss of ribosomes in the ICL-M-treated *P*. *falciparum*. This result supports the finding from our proteomics analysis that ribosomal proteins were highly downregulated in ICL-M-treated *P*. *falciparum*. Similarly abnormal parasite morphology was observed by previous work testing other antimalarial drugs, such as artesunate, quinine, and piperaquine [59]. Ribosome depletion leads to a lack of necessary protein functions for parasite biological processes. Moreover, the expression of ribosomal proteins is crucial for the development of several organisms. For example, the ribosomal protein L19 expression differs throughout the growth phases of *Leishmania* spp. during their lifecycle [60]. Additionally, a study in female *Schistosoma japonicum* reported that the ribosomal protein expression profiles were changed during sexual maturation [61]. Thus, the downregulation of ribosomal proteins after ICL-M treatment may affect the development of *P*. *falciparum*.

## Conclusion

The effect of ICL-M, a derivative of isocryptolepine, on malarial parasites was studied *in vitro* using *P*. *falciparum* 3D7 as a model. This compound had an antimalarial effect on both the ring and trophozoite stages of *P*. *falciparum*. Our proteomics analysis revealed that ICL-M disrupts several *P*. *falciparum* biological processes; ribosomal, proteasomal, and metabolism pathways were the main pathways found to be affected by ICL-M treatment.

## Supporting information

S1 File Protein identification from differential proteomics experiment replication 1. Total proteins were identified and quantified by Mascot daemon version 2.3.2 software. The protein database was collected from an NCBInr database (24 October 2018) specific to *P*. *falciparum* 3D7.
(PDF)

S2 File Protein identification from differential proteomics experiment replication 2. Total proteins were identified and quantified by Mascot daemon version 2.3.2 software. The protein database was collected from an NCBInr database (24 October 2018) specific to *P*. *falciparum* 3D7.
(PDF)

S3 File Protein identification from differential proteomics experiment replication 3. Total proteins were identified and quantified by Mascot daemon version 2.3.2 software. The protein database was collected from an NCBInr database (24 October 2018) specific to *P*. *falciparum* 3D7.
(PDF)

S4 File Selection of differential proteins from three biological replicates. The experiment was performed in three biological replicates. Proteins with altered expression levels were selected based on different expression levels in at least two biological replicates.
(PDF)
