# Supplementary material for: Proteomic analysis of Plasmodium falciparum response to isocryptolepine derivative
Source: PLoS One. 2019 Aug 8;14(8):e0220871. doi: 10.1371/journal.pone.0220871 (PMC6687117; doi:10.1371/journal.pone.0220871)
Supplement: S1 File — Total proteins were identified and quantified by Mascot daemon version 2.3.2 software. The protein database was collected from an NCBInr database (24 October 2018) specific to P. falciparum 3D7. (PDF) [file pone.0220871.s001.pdf]

Supplement 1 file. Protein identification from differential proteomics experiment replication 1

Total proteins were identified and quantified by Mascot daemon version 2.3.2 software.

Protein database was collected from NCBInr database (24 October 2018) specific to *P. falciparum* 3D7

Rep.1		Rep.1 DMSO									Rep.1 ICL-M									ICL-M	ICL-M
NCBI acc_no	All prot_desc	prot_score	prot_mass	prot_matc	prot_matc	prot_sequ	prot_sequ	prot_cove	prot_pi	emPAI	prot_score	prot_mass	prot_matc	prot_matc	prot_sequ	prot_sequ	prot_cove	prot_pi	emPAI	Fold change	Up/Down
gi|23499105	heat shock protein 70	6079	73868	247	196	35	27	47.4	5.51	4.29	7008	73868	293	227	32	27	43.7	5.51	4.07	0.95	
gi|23505079	heat shock protein 70	4107	72343	202	126	23	19	41.6	5.18	2.82	4808	72343	212	139	24	18	43.7	5.18	1.97	0.70	
gi|124810131	glyceraldehyde-3-phosphate dehydrogenase	3515	36612	100	80	23	19	75.7	7.59	8.66	3412	36612	93	76	21	17	71.2	7.59	8.14	0.94	
gi|371941768	Hsp70-x	2473	75006	106	81	14	6	23.4	5.59	0.41	2586	75006	122	94	12	6	20.3	5.59	0.44	1.07	
gi|50400239	RecName: Full=Enolase; AltName: Full=2-phospho-D-glycerate hydro-lyase; AltName: Full=2-phosphoglycerate dehydratase	2187	48647	82	54	25	16	59	6.21	3.58	2155	48647	82	51	25	17	60.5	6.21	4.74	1.32	
gi|23615698	elongation factor 1-alpha	2164	48928	164	95	19	14	51.5	9.12	2.97	2443	48928	173	103	20	14	54	9.12	3.94	1.33	
gi|23498766	heat shock protein 90	1853	86112	108	59	32	16	38.1	4.94	1.56	1832	86112	103	60	31	14	35	4.94	1.31	0.84	
gi|46361043	ornithine aminotransferase	1329	46025	60	37	16	10	31.9	6.47	1.65	1442	46025	66	39	17	9	37.2	6.47	1.61	0.98	
gi|124803934	GTP-binding nuclear protein RAN/TC4	1293	24860	46	35	11	8	57	7.72	2.14	1358	24860	58	39	13	8	61.7	7.72	2.82	1.32	
gi|23615406	L-lactate dehydrogenase	1195	34086	56	40	13	11	40.8	7.12	4.39	1413	34086	51	42	14	11	42.7	7.12	4.93	1.12	
gi|225632282	protein disulfide isomerase	1013	55479	58	33	13	9	29.2	5.56	1.53	1266	55479	65	43	13	10	29	5.56	1.67	1.09	
gi|344189571	Chain A, Phosphoglycerate Kinase	987	46264	57	32	31	13	70.5	7.82	2.25	1170	46264	68	37	37	17	65.3	7.82	4.82	2.14	Up
gi|129926	RecName: Full=Phosphoglycerate kinase	987	45398	43	32	20	13	59.9	7.63	2.33	1170	45398	54	37	24	15	62.7	7.63	4.58	1.97	
gi|74920225	RecName: Full=Fructose-bisphosphate aldolase	962	40080	49	33	18	13	52.8	8.33	2.89	1013	40080	47	29	19	12	57.2	8.33	3.2	1.11	
gi|124803500	histone H2B	804	13117	76	39	11	9	59.8	10.27	65.99	663	13117	89	47	11	9	59.8	10.27	30.23	0.46	Down
gi|31505529	histone H4, partial	755	11448	31	22	6	4	48.5	11.23	7.33	747	11448	35	22	7	5	66	11.23	11.28	1.54	
gi|124809712	elongation factor 2	744	93462	31	16	13	4	22.5	6.36	0.19	894	93462	41	20	18	6	28.2	6.36	0.25	1.32	
gi|23498286	40S ribosomal protein S19	693	19710	34	19	11	8	60.6	10.17	5.69	854	19710	37	27	7	6	47.6	10.17	2.8	0.49	Down
gi|23505200	merozoite surface protein 1	644	195605	40	21	22	11	17.6	6.11	0.22	630	195605	40	18	21	9	17.5	6.11	0.19	0.86	
gi|225632253	14-3-3 protein	622	30174	31	18	10	6	50	4.86	1.58	466	30174	36	17	10	6	42.7	4.86	2.04	1.29	
gi|124806075	endoplasmin, putative	592	94959	56	17	22	9	37.1	5.28	0.41	848	94959	61	27	22	10	34.8	5.28	0.44	1.07	
gi|46361058	histone H3	468	15437	63	25	12	6	49.3	11.14	5.07	573	15437	68	30	12	6	47.1	11.14	3.38	0.67	
gi|124804377	60S ribosomal protein P0	450	34945	19	11	10	4	42.1	6.28	0.89	514	34945	25	15	12	6	46.8	6.28	1.38	1.55	
gi|124802054	DNA/RNA-binding protein Alba 3	415	11969	24	12	9	4	86.9	9.3	3.58	282	11969	16	10	5	3	66.4	9.3	2.8	0.78	
gi|23498886	40S ribosomal protein S5, putative	411	21849	17	11	8	4	45.1	9.67	1.74	310	21849	17	9	8	4	41.5	9.67	1.89	1.09	
gi|303324901	Chain A, Heat Shock Protein 86	396	25195	25	16	7	3	32.7	4.85	0.46	-	-	-	-	-	-	-	-	-	Down-Detect	Down
gi|23615568	phosphoethanolamine N-methyltransferase	360	31024	16	14	6	5	24.8	5.43	0.67	563	31024	22	16	8	5	38.3	5.43	0.92	1.37	
gi|23499152	DNA/RNA-binding protein Alba 1	357	27242	43	18	12	5	40.7	10.58	1.54	385	27242	44	19	11	5	38.7	10.58	1.68	1.09	
gi|4493980	peptidyl-prolyl cis-trans isomerase	356	18940	18	14	6	6	31	8.29	3.4	423	18940	20	17	7	6	36.8	8.29	3.77	1.11	
gi|225631678	60S ribosomal protein L12, putative	326	18101	17	10	9	4	58.8	9.54	0.99	326	18101	16	8	9	4	57	9.54	1.48	1.49	
gi|23504519	actin-depolymerizing factor 1	325	13732	7	7	1	1	18	7.66	0.25	467	13732	15	10	4	2	49.2	7.66	0.6	2.40	Up
gi|4494010	histone H2A variant, putative	320	16443	25	13	4	3	30.4	10.63	0.76	353	16443	29	19	4	3	30.4	10.63	0.82	1.08	
gi|225631753	cell division cycle protein 48 homologue,putative	290	92329	25	8	14	3	23.3	4.95	0.15	315	92329	28	10	13	3	24.9	4.95	0.16	1.07	
gi|124803860	peptidyl-prolyl cis-trans isomerase	288	21717	16	11	7	6	39	7.1	1.75	221	21717	24	9	10	4	51.3	7.1	1.14	0.65	
gi|124802119	adenylate kinase	285	27594	18	10	10	7	46.3	8.97	2.15	336	27594	26	14	12	8	43	8.97	2.79	1.30	
gi|46361129	histone H2A	273	14114	27	11	5	2	43.9	10.29	0.92	362	14114	28	18	4	3	38.6	10.29	1.5	1.63	
gi|23499115	high mobility group protein B2	264	11527	10	9	4	3	39.4	9.97	1.86	249	11527	9	7	4	3	42.4	9.97	2.02	1.09	
gi|75009812	RecName: Full=Triosephosphate isomerase; Short=TIM; AltName: Full=Triose-phosphate isomerase	260	27917	11	8	6	4	19.8	6.01	0.76	376	27917	17	11	7	3	29.4	6.01	1.05	1.38	
gi|23505159	conserved Plasmodium protein, unknown function	247	24683	17	12	6	3	30.4	5.49	0.67	238	24683	17	10	5	3	27.2	5.49	0.72	1.07	
gi|258597456	nucleosome assembly protein	245	40463	17	9	4	1	13.8	4.76	0.08	367	40463	19	9	4	1	13.5	4.76	0.09	1.13	
gi|23504687	40S ribosomal protein S9, putative	237	22109	18	10	8	6	45.5	10.45	1.35	109	22109	12	5	5	2	23.8	10.45	0.35	0.26	Down
gi|23504938	alpha tubulin 1	234	50264	8	7	3	2	11.9	4.93	0.21	160	50264	10	4	6	1	21	4.93	0.07	0.33	Down
gi|23504556	60S ribosomal protein L4	229	46183	17	5	11	4	27.5	10.5	0.42	189	46183	10	4	8	3	23.1	10.5	0.25	0.60	
gi|124810348	exported protein 2	229	33391	15	9	7	3	19.5	5.1	0.46	176	33391	14	9	6	4	18.8	5.1	0.66	1.43	
gi|124808771	60S ribosomal protein L5, putative	223	33977	9	6	7	4	21.1	9.78	0.46	186	33977	7	5	5	4	21.1	9.78	0.49	1.07	
gi|74876423	RecName: Full=Tubulin beta chain; AltName: Full=Beta-tubulin	221	49719	17	6	8	4	28.3	4.73	0.3	299	49719	11	7	6	3	20	4.73	0.31	1.03	
gi|11127605	heat shock protein hsp70 homologue Pfhsp70-3	212	71546	27	8	13	6	28.7	5.9	0.37	-	-	-	-	-	-	-	-	-	Down-Detect	Down

gi|124805478	eukaryotic translation initiation factor 5A	212	17620	11	6	7	3	55.9	5.42	1.02	222	17620	11	7	8	4	66.5	5.42	1.53	1.50	
gi|225631960	40S ribosomal protein S19	208	16753	7	6	2	2	22.8	10.27	0.74	234	16753	8	4	3	1	31.7	10.27	0.8	1.08	
gi|124808810	60S ribosomal protein L21	201	18783	21	9	6	1	31.1	10.04	0.65	243	18783	31	13	6	2	35.4	10.04	1.02	1.57	
gi|23504648	40S ribosomal protein S11	194	16067	15	6	6	3	41.7	10.49	1.16	236	16067	22	8	8	3	51	10.49	1.25	1.08	
gi|23615723	40S ribosomal protein S15	191	17240	19	5	8	2	57.6	10.38	2.54	107	17240	15	3	8	2	49	10.38	2.79	1.10	
gi|124804238	40S ribosomal protein S18, putative	181	17880	14	9	6	3	47.4	10.46	1.01	255	17880	21	11	7	3	49.4	10.46	2.01	1.99	
gi|258596854	stevor	180	34204	13	9	3	1	12.8	8.76	0.1	159	34204	11	7	3	1	8.2	8.76	0.1		
gi|23615606	DNA/RNA-binding protein Alba 4	174	42133	10	4	6	1	16.9	7.14	0.08	151	42133	14	3	9	1	25.8	7.14	0.08		
gi|23504494	mature parasite-infected erythrocyte surface antigen	171	168186	24	6	14	3	14	4.76	0.12	275	168186	25	9	16	5	13	4.76	0.13	1.08	
gi|23504618	purine nucleoside phosphorylase	171	26841	17	6	6	4	36.7	6.07	0.6	-	-	-	-	-	-	-	-	-	Down-Detect	Down
gi|282403624	Chain A, Uridine Phosphorylase, Putative	171	30453	17	6	6	4	32.6	5.83	0.52	134	30453	16	4	8	3	30.1	5.83	0.39	0.75	
gi|225631740	60S ribosomal protein L19	168	21566	11	9	4	4	13.7	11.32	0.79	117	21566	11	5	4	4	13.7	11.32	0.85	1.08	
gi|46361130	histone H3 variant, putative	167	15432	52	15	9	3	39	11.15	0.82	213	15432	55	19	10	4	44.9	11.15	1.33	1.62	
gi|124805752	glutathione peroxidase-like thioredoxin peroxidase	166	23937	15	7	5	1	33.7	8.99	0.14	50	23937	15	2	4	1	23.9	8.99	0.32	2.29	Up
gi|124810293	eukaryotic initiation factor 4A	165	45281	17	4	9	2	27.6	5.48	0.24	409	45281	23	12	9	4	28.4	5.48	0.45	1.88	
gi|7768287	formate-nitrite transporter	161	34436	12	4	4	2	17.2	8.74	0.2	176	34436	17	6	5	2	19.7	8.74	0.22	1.10	
gi|124801997	60S ribosomal protein L13, putative	160	23739	15	4	8	2	33.7	10.19	0.3	207	23739	15	5	4	2	18.3	10.19	0.32	1.07	
gi|124808442	60S ribosomal protein L10, putative	155	25200	9	2	6	1	20.5	9.97	0.29	304	25200	15	6	7	2	34.7	9.97	0.3	1.03	
gi|124800689	knob-associated histidine-rich protein	155	71259	15	5	7	2	13.9	9.17	0.1	192	71259	17	6	6	2	9.8	9.17	0.1		
gi|124804166	splicing factor, putative	153	16002	12	5	2	1	31	5.1	0.47	183	16002	9	6	2	1	20	5.1	0.84	1.79	
gi|124809606	40S ribosomal protein S5	150	29939	10	5	7	4	39.3	10.02	0.53	159	29939	8	4	6	3	26.5	10.02	0.4	0.75	
gi|23504496	Plasmodium exported protein, unknown function	147	30696	5	4	2	1	10.8	9.47	0.23	130	30696	7	4	3	1	13.1	9.47	0.24	1.04	
gi|124803848	cysteine proteinase falcipain 2b	146	55768	19	6	9	4	21.2	8.14	0.34	171	55768	23	5	7	2	17.8	8.14	0.28	0.82	
gi|124803863	cysteine proteinase falcipain 2a	146	55892	19	6	9	4	20.5	7.12	0.33	171	55892	18	5	6	2	18.8	7.12	0.28	0.85	
gi|23615388	60S ribosomal protein L6, putative	142	21588	11	7	7	4	29.5	9.88	0.79	114	21588	8	4	7	4	37.4	9.88	0.85	1.08	
gi|237640532	Chain A, HAP protein	141	37376	12	6	7	5	22.9	4.97	0.67	-	-	-	-	-	-	-	-	-	Down-Detect	Down
gi|23504595	histamine-releasing factor	140	19967	8	3	3	1	16.4	4.58	0.17	179	19967	13	4	5	1	23.4	4.58	0.39	2.29	Up
gi|46361220	pyruvate kinase	139	55625	21	5	8	4	25.4	7.5	0.26	119	55625	20	4	13	2	36.4	7.5	0.13	0.50	Down
gi|8247298	hypothetical protein, partial	135	4755	3	3	2	2	60	9.23	2.16	109	4755	2	2	1	1	32.5	9.23	0.82	0.38	Down
gi|258549116	serine esterase, putative	134	216941	50	6	21	1	18.7	8.82	0.02	66	216941	47	0	20	0	15.8	8.82	0.02		
gi|46361040	proteasome subunit alpha type-2, putative	132	26512	7	4	3	2	14.9	5.4	0.43	185	26512	9	6	3	2	14.9	5.4	0.46	1.07	
gi|75016040	RecName: Full=Acidic leucine-rich nuclear phosphoprotein 32-related protein; AltName: Full=ANP32/acidic nuclear phosphoprotein-like protein	131	32986	11	6	6	3	24.2	4.27	0.34	143	32986	10	5	4	3	13.5	4.27	0.67	1.97	
gi|124803852	cysteine proteinase falcipain 3	131	56630	32	6	8	1	17.5	6.59	0.12	75	56630	31	5	11	2	26	6.59	0.27	2.25	Up
gi|268612503	Chain A, Phosphoglycerate mutase	125	29716	17	6	7	4	36.8	8.26	0.71	180	29716	16	7	9	5	37.6	8.26	0.97	1.37	
gi|124804024	phosphoglycerate mutase, putative	125	28752	17	6	8	4	46.8	8.3	0.74	192	28752	16	8	10	6	40.8	8.3	1.26	1.70	
gi|93278503	Chain A, Falcipain 2	124	27140	15	4	6	2	32	4.98	0.42	-	-	-	-	-	-	-	-	-	Down-Detect	Down
gi|23499154	60S ribosomal protein L13-2, putative	123	25425	8	3	5	2	24.2	10.78	0.28	-	-	-	-	-	-	-	-	-	Down-Detect	Down
gi|124809152	mitochondrial acidic protein MAM33, putative	122	28854	17	5	4	2	23	4.89	0.55	119	28854	17	4	6	2	23.8	4.89	0.42	0.76	
gi|124808201	40S ribosomal protein S8e, putative	119	25035	11	5	6	3	24.3	9.98	0.46	81	25035	9	3	6	3	25.7	9.98	0.49	1.07	
gi|23504955	high molecular weight rhoptry protein 3	118	104789	19	4	11	2	21.4	6.25	0.06	65	104789	16	1	11	1	16.7	6.25	0.03	0.50	Down
gi|23615526	60S ribosomal protein L6-2, putative	116	25516	7	4	5	2	19.5	10.1	0.45	222	25516	9	7	4	3	19	10.1	0.48	1.07	
gi|23499261	1-cys peroxiredoxin	114	25148	15	5	7	2	40	6.31	0.29	145	25148	12	5	5	1	24.5	6.31	0.14	0.48	Down
gi|23615644	60S ribosomal protein L17, putative	109	23400	20	5	8	1	36.5	10.89	0.31	-	-	-	-	-	-	-	-	-	Down-Detect	Down
gi|4493906	40S ribosomal protein S12, putative	107	15387	11	4	5	3	30.5	4.9	0.82	186	15387	12	8	5	3	39	4.9	0.88	1.07	
gi|46361162	pyridoxine biosynthesis protein PDX1	107	32992	11	2	6	1	30.2	6.76	0.21	238	32992	17	7	13	4	55.1	6.76	0.67	3.19	Up
gi|124802718	adenosine deaminase	103	42438	25	7	11	4	29.4	5.41	0.7	111	42438	33	4	15	3	34.9	5.41	0.49	0.70	
gi|124809201	glucose-6-phosphate isomerase	99	67325	15	4	8	2	17.6	6.78	0.1	80	67325	20	4	9	2	20.7	6.78	0.11	1.10	
gi|124806612	conserved protein, unknown function	98	23644	13	2	6	2	25.2	9.64	0.31	-	-	-	-	-	-	-	-	-	Down-Detect	Down
gi|23504725	karyopherin beta	98	127272	6	2	6	2	7.9	4.8	0.05	-	-	-	-	-	-	-	-	-	Down-Detect	Down
gi|258597720	60S ribosomal protein L7-3, putative	97	32661	20	4	4	3	16.3	10.17	0.34	65	32661	17	3	4	2	13.4	10.17	0.23	0.68	
gi|23499195	acyl-CoA binding protein, putative	97	10768	6	3	2	1	35.6	7.68	0.75	94	10768	8	3	3	1	40	7.68	0.8	1.07	
gi|23505219	profilin, putative	97	19005	3	3	1	1	4.7	4.22	0.18	97	19005	4	3	2	1	12.9	4.22	0.19	1.06	
gi|23615603	nucleoside transporter 1	96	47600	9	3	3	2	8.1	8.36	0.14	115	47600	9	5	3	2	6.9	8.36	0.15	1.07	
gi|23498814	histone H2B variant	93	13755	21	7	6	1	51.2	10.8	0.56	173	13755	22	10	5	1	46.3	10.8	0.6	1.07	
gi|18076407	early transcribed membrane protein 14.1	93	11420	2	2	1	1	12.1	9.63	0.3	92	11420	4	2	3	1	20.6	9.63	0.32	1.07	

gi|124809402	60S ribosomal protein L1, putative	93	24790	11	2	7	1	35.9	9.85	0.29	120	24790	11	4	5	2	25.8	9.85	0.5	1.72	
gi|23615172	40S ribosomal protein S7, putative	90	22467	13	4	6	2	34	9.81	0.32	85	22467	17	4	5	1	39.7	9.81	0.16	0.50	Down
gi|124802670	60S ribosomal protein L3	89	44193	11	4	8	2	25.1	10.21	0.44	86	44193	10	3	7	2	29.3	10.21	0.17	0.39	Down
gi|23615551	60S ribosomal protein L18, putative	88	21733	12	2	7	2	45.1	10.62	0.33	22	21733	11	0	6	0	29.9	10.62	0.16	0.48	Down
gi|258597310	60S ribosomal protein L35, putative	87	14739	5	4	4	3	19.4	10.79	0.87	66	14739	7	3	5	2	25	10.79	0.55	0.63	
gi|23615654	thioredoxin-related protein, putative	86	23972	8	3	5	2	26.9	9.44	0.69	22	23972	5	0	4	0	19.7	9.44	0.15	0.22	Down
gi|124802073	single-strand telomeric DNA-binding protein GBP2, putative	85	29512	9	3	5	2	21.1	9.2	0.38	58	29512	7	2	4	1	19.5	9.2	0.26	0.68	
gi|23615715	HVA22-like protein, putative	85	18410	4	3	2	1	11.8	9.34	0.18	49	18410	2	2	1	1	6.5	9.34	0.2	1.11	
gi|258597201	40S ribosomal protein S4, putative	82	29753	7	2	5	2	28.7	10.09	0.24	104	29753	8	2	4	1	24.1	10.09	0.25	1.04	
gi|124810210	40S ribosomal protein S3	80	24652	8	3	5	2	24.9	10.2	0.29	128	24652	14	6	7	2	33.5	10.2	0.5	1.72	
gi|23498182	V-type proton ATPase subunit B	78	55753	9	2	6	2	18.6	5.46	0.12	77	55753	12	2	10	2	32.4	5.46	0.13	1.08	
gi|23498728	Plasmodium exported protein, unknown function	75	27656	6	5	2	2	10.7	8.55	0.26	61	27656	6	2	3	1	23.3	8.55	0.27	1.04	
gi|124810024	conserved Plasmodium protein, unknown function	74	40043	4	1	4	1	15	9.6	0.08	83	40043	3	2	2	1	8.5	9.6	0.09	1.13	
gi|7672213	eukaryotic translation initiation factor 3 subunit K, putative	73	28017	6	1	3	1	24.7	5.61	0.12	92	28017	2	1	1	1	7.2	5.61	0.27	2.25	Up
gi|23498233	small GTP-binding protein sar1	72	22006	8	4	3	2	16.7	6.75	0.33	60	22006	7	2	2	1	9.9	6.75	0.16	0.48	Down
gi|46576603	RecName: Full=Probable cathepsin C; Flags: Precursor	70	80361	22	3	10	2	21.7	5.82	0.08	62	80361	15	3	5	1	8.7	5.82	0.09	1.13	
gi|23498770	heat shock protein 110	68	99902	39	2	8	2	11.3	5.54	0.07	146	99902	45	4	14	3	17.4	5.54	0.11	1.57	
gi|23498142	Plasmodium exported protein (PHISTa), unknown function	67	49713	5	3	3	1	9.8	9.51	0.07	83	49713	7	2	3	1	9.3	9.51	0.07		
gi|23505019	falstatin	66	46929	11	2	4	1	16.7	6.22	0.07	33	46929	9	1	4	1	15	6.22	0.08	1.14	
gi|124802973	ADP/ATP transporter on adenylate translocase	66	33705	8	2	6	1	26.2	9.68	0.1	57	33705	5	2	3	1	11	9.68	0.1		
gi|124810483	proteasome subunit alpha type-1, putative	66	28819	3	1	2	1	10.2	5.51	0.12	93	28819	5	1	5	1	23.2	5.51	0.12		
gi|23615786	ubiquitin-60S ribosomal protein L40	66	14608	6	3	4	2	27.3	9.91	0.52	99	14608	19	6	6	3	50.8	9.91	0.94	1.81	
gi|46361167	nascent polypeptide-associated complex subunit alpha, putative	65	20610	6	2	4	1	31.5	4.77	0.16	50	20610	4	2	2	1	18.5	4.77	0.17	1.06	
gi|124804546	conserved Plasmodium protein, unknown function	64	28478	19	3	4	1	19.6	8.8	0.12	71	28478	17	2	2	1	7.8	8.8	0.12		
gi|23504696	S-adenosyl-L-homocysteine hydrolase	64	53804	8	2	6	1	13.6	5.64	0.06	68	53804	16	2	8	1	30.5	5.64	0.07	1.17	
gi|258597663	nuclear transport factor 2, putative	62	14428	4	3	3	3	31.2	5.53	0.9	26	14428	2	0	2	0	25.6	5.53	0.25	0.28	Down
gi|23498140	Plasmodium exported protein (PHISTb), unknown function	62	60233	20	1	8	1	14.3	8.76	0.05	50	60233	13	2	8	2	15.5	8.76	0.19	3.80	Up
gi|3122763	RecName: Full=60S acidic ribosomal protein P2	61	11941	5	1	3	1	60.7	4.49	0.29	78	11941	6	1	3	1	54.5	4.49	0.7	2.41	Up
gi|23504501	rhoptry-associated protein 3	60	46974	4	1	4	1	17	8.66	0.07	-	-	-	-	-	-	-	-	-	Down-Detect	Down
gi|124802223	hypoxanthine-guanine phosphoribosyltransferase	60	26346	10	4	3	2	8.7	7.59	0.27	57	26346	13	3	5	1	24.2	7.59	0.29	1.07	
gi|23615498	merozoite surface protein 7	57	41251	6	1	4	1	18.5	4.74	0.08	43	41251	9	1	5	1	15.7	4.74	0.09	1.13	
gi|23498881	conserved Plasmodium protein, unknown function	56	29231	2	1	2	1	11.1	8.76	0.11	66	29231	4	2	3	1	12.7	8.76	0.12	1.09	
gi|124801947	RNA-binding protein, putative	55	30033	10	1	6	1	19.4	10.07	0.11	29	30033	11	0	5	0	24.2	10.07	0.12	1.09	
gi|124809822	6-phosphogluconate dehydrogenase,decarboxylating, putative	55	52960	18	2	7	1	15	6.58	0.13	32	52960	15	1	7	1	17.7	6.58	0.07	0.54	
gi|124804373	60S ribosomal protein L38	54	10307	5	1	4	1	46	10.71	0.34	36	10307	3	1	3	1	28.7	10.71	0.84	2.47	Up
gi|23615340	conserved Plasmodium protein, unknown function	51	88519	16	3	8	1	13.2	9.07	0.04	44	88519	14	3	8	1	13.9	9.07	0.04		
gi|258597482	10 kDa chaperonin	51	11143	4	1	4	1	43.7	5.47	0.31	58	11143	3	1	3	1	33	5.47	0.77	2.48	Up
gi|23505090	nucleosome assembly protein	49	31807	9	2	5	1	20.8	4.17	0.22	52	31807	5	2	4	1	17.5	4.17	0.24	1.09	
gi|124803892	folate transporter 2	49	51321	5	2	4	1	10.3	8.63	0.06	36	51321	3	1	3	1	9.9	8.63	0.07	1.17	
gi|4494003	40S ribosomal protein S3A, putative	48	30028	3	1	2	1	7.3	9.8	0.24	35	30028	7	1	5	1	21.4	9.8	0.12	0.50	Down
gi|23504974	ATP-dependent protease ATPase subunit ClpY	48	106396	36	2	12	1	14.9	8.42	0.03	32	106396	40	1	13	1	16.6	8.42	0.03		
gi|46361227	protein DJ-1	48	20280	3	2	2	1	15.9	6.95	0.17	22	20280	4	0	3	0	22.8	6.95	0.18	1.06	
gi|23615478	20 kDa chaperonin	48	29045	1	1	1	1	4.7	7.63	0.12	65	29045	4	1	2	1	7	7.63	0.12		
gi|23504603	inositol-3-phosphate synthase	47	69069	20	1	12	1	23.3	7.11	0.15	60	69069	20	1	10	1	22	7.11	0.1	0.67	
gi|23499218	ras-related protein Rab-18	47	23148	2	1	1	1	5.5	8.11	0.15	26	23148	3	0	3	0	17.9	8.11	0.15		
gi|124806534	serine hydroxymethyltransferase	47	49749	11	1	9	1	26.7	8.29	0.07	79	49749	19	2	12	2	26.7	8.29	0.15	2.14	Up
gi|651207688	Chain A, Serine Hydroxymethyltransferase	47	54007	16	1	11	1	31	7.21	0.06	79	54007	21	2	14	2	31	7.21	0.13	2.17	Up
gi|23504681	40S ribosomal protein S24	46	15382	6	2	3	1	21.1	10.75	0.49	51	15382	5	3	3	1	18	10.75	0.52	1.06	
gi|8052274	elongation factor 1 (EF-1), putative	46	17695	4	1	3	1	16.7	4.5	0.19	26	17695	3	0	2	0	14.7	4.5	0.2	1.05	
gi|124803623	endoplasmic reticulum-resident calcium binding protein	45	39350	4	1	3	1	9.3	4.49	0.18	84	39350	4	2	3	1	8.5	4.49	0.09	0.50	Down
gi|23504857	Hsc70-interacting protein	45	51092	7	2	5	1	13.8	4.67	0.07	-	-	-	-	-	-	-	-	-	Down-Detect	Down
gi|124806378	ras-related protein Rab-2	45	24408	6	1	6	1	46	6.33	0.14	-	-	-	-	-	-	-	-	-	Down-Detect	Down
gi|23498727	small exported membrane protein 1	45	14186	5	2	3	1	22	9.61	0.24	38	14186	2	1	1	1	7.3	9.61	0.26	1.08	
gi|124806302	WD repeat-containing protein, putative	45	378674	51	2	25	1	9.8	9.02	0.01	37	378674	54	1	24	1	9.3	9.02	0.01		
gi|23615189	small heat shock protein, putative	44	25179	5	1	2	1	11.4	6.02	0.13	73	25179	7	2	5	1	23.7	6.02	0.14	1.08	

gi|23498791	AAA family ATPase, CDC48 subfamily	43	141991	19	1	9	1	8.3	9.07	0.02	47	141991	15	1	12	1	13.5	9.07	0.02		
gi|23615236	60S ribosomal protein L24, putative	43	19232	6	2	5	1	23.5	10.43	0.18	-	-	-	-	-	-	-	-	-	Down-Detect	Down
gi|3758867	proteasome subunit alpha type-3, putative	42	29270	7	1	5	1	21	6.38	0.11	42	29270	9	1	6	1	29	6.38	0.12	1.09	
gi|23498939	proteasome subunit alpha type-5, putative	42	28370	7	2	4	1	31.3	4.96	0.12	109	28370	11	5	3	2	19.1	4.96	0.6	5.00	Up
gi|124806724	GAS8-like protein, putative	42	54772	5	1	4	1	8.7	7.98	0.06	-	-	-	-	-	-	-	-	-	Down-Detect	Down
gi|124802168	eukaryotic translation initiation factor 2 subunit beta, putative	42	25306	12	1	5	1	24.3	9.23	0.13	-	-	-	-	-	-	-	-	-	Down-Detect	Down
gi|124804998	rifin	42	38701	18	2	8	1	28.1	8.96	0.09	62	38701	10	3	6	1	21.9	8.96	0.09		
gi|23504672	WD repeat-containing protein 26, putative	41	150608	44	3	18	1	16.8	9.24	0.02	38	150608	40	2	14	1	13.2	9.24	0.02		
gi|6851056	spermidine synthase	40	36573	10	1	6	1	23.7	6.97	0.09	32	36573	6	1	5	1	26.2	6.97	0.1	1.11	
gi|225632259	U6 snRNA-associated Sm-like protein LSm3,putative	40	10694	2	1	2	1	26.4	5.16	0.33	44	10694	5	2	4	1	61.5	5.16	0.35	1.06	
gi|258597872	signal peptide peptidase	40	47547	4	1	4	1	9	8.95	0.07	-	-	-	-	-	-	-	-	-	Down-Detect	Down
gi|124808815	basic transcription factor 3b, putative	40	19381	2	1	2	1	9.9	9.04	0.17	46	19381	4	1	4	1	28.1	9.04	0.18	1.06	
gi|7799189	thioredoxin	39	11709	16	1	4	1	60.6	4.67	0.3	-	-	-	-	-	-	-	-	-	Down-Detect	Down
gi|258597884	60S ribosomal protein L27	39	16735	8	1	5	1	40.4	10.23	0.45	51	16735	9	1	5	1	31.5	10.23	0.22	0.49	Down
gi|124802189	proteasome subunit beta type-5	39	30577	3	1	3	1	14.4	5.18	0.11	37	30577	4	1	3	1	12.9	5.18	0.12	1.09	
gi|75016029	RecName: Full=STI1-like protein	38	66015	8	1	4	1	7.8	6.63	0.05	50	66015	14	1	6	1	11.7	6.63	0.11	2.20	Up
gi|124807078	rhoptry neck protein 3	38	262988	65	2	30	1	15.6	9.23	0.01	30	262988	50	1	25	1	14	9.23	0.01		
gi|23615363	conserved Plasmodium protein, unknown function	37	170566	56	0	13	0	11.4	8.86	0.04	51	170566	64	0	10	0	8.5	8.86	0.04		
gi|23615558	40S ribosomal protein S6	37	35363	4	1	4	1	17.3	10.44	0.09	-	-	-	-	-	-	-	-	-	Down-Detect	Down
gi|124809308	thioredoxin peroxidase 1	37	21793	10	1	2	1	11.8	6.65	0.33	83	21793	14	4	4	2	20.5	6.65	0.58	1.76	
gi|23505054	6-phosphofructokinase	37	159351	27	1	17	1	18.5	6.32	0.02	52	159351	21	2	13	1	14.6	6.32	0.02		
gi|4493898	ubiquitin-conjugating enzyme E2, putative	36	16130	1	1	1	1	9.9	4.88	0.21	-	-	-	-	-	-	-	-	-	Down-Detect	Down
gi|23615263	M1-family alanyl aminopeptidase	36	125983	16	1	13	1	15.9	7.3	0.03	46	125983	14	1	12	1	13.3	7.3	0.03		
gi|59798920	RecName: Full=Serine-repeat antigen protein; AltName: Full=111 kDa antigen; AltName: Full=p126; Flags: Precursor	36	111698	23	1	14	1	17.1	5.26	0.03	28	111698	28	0	14	0	14.9	5.26	0.03		
gi|74930131	RecName: Full=40S ribosomal protein SA	35	29837	16	4	5	1	22.8	5.91	0.11	46	29837	12	3	5	1	20.5	5.91	0.12	1.09	
gi|225632158	conserved Plasmodium protein, unknown function	35	24366	2	1	2	1	9.1	5.2	0.14	26	24366	2	0	2	0	10.5	5.2	0.15	1.07	
gi|258596875	26S proteasome regulatory subunit RPN1,putative	34	108289	24	1	11	1	16.6	5.95	0.03	49	108289	38	1	17	1	26	5.95	0.03		
gi|23499155	40S ribosomal protein S16, putative	34	16275	4	1	4	1	27.1	10.25	0.21	55	16275	4	1	3	1	20.1	10.25	0.22	1.05	
gi|23615559	aconitate hydratase	34	103313	21	1	11	1	12.3	7.73	0.03	63	103313	23	1	9	1	10.1	7.73	0.03		
gi|124806145	polyadenylate-binding protein, putative	34	97169	10	1	6	1	9.8	8.96	0.03	41	97169	7	1	5	1	8.8	8.96	0.07	2.33	Up
gi|23504716	multidrug resistance protein 1	34	162150	35	1	18	1	18	8.94	0.04	43	162150	30	1	12	1	12.4	8.94	0.04		
gi|258597955	conserved Plasmodium membrane protein, unknown function	34	577659	71	3	36	1	8.8	8.86	0.01	34	577659	71	1	36	1	8.7	8.86	0.01		
gi|23615265	malonyl CoA-acyl carrier protein transacylase precursor	34	46275	5	2	3	1	10.7	8.71	0.07	-	-	-	-	-	-	-	-	-	Down-Detect	Down
gi|75009813	RecName: Full=Plasmepsin-1; AltName: Full=Aspartic hemoglobinase I; AltName: Full=PfAPG; Flags: Precursor	34	51428	3	1	3	1	8.6	6.72	0.06	34	51428	3	1	3	1	11.3	6.72	0.07	1.17	
gi|124809547	histidine--tRNA ligase, putative	34	133583	32	1	21	1	21.7	7.91	0.02	-	-	-	-	-	-	-	-	-	Down-Detect	Down
gi|23615660	Plasmodium exported protein, unknown function	33	32763	5	2	3	2	13.3	9.23	0.34	27	32763	4	0	3	0	11.6	9.23	0.23	0.68	
gi|23498992	surface-associated interspersed protein 8.2 (SURFIN 8.2)	33	248324	30	1	19	1	11.7	5.35	0.01	24	248324	37	0	25	0	14.1	5.35	0.03	3.00	Up
gi|225632239	conserved Plasmodium protein, unknown function	33	76808	32	1	11	1	14.3	5.34	0.04	33	76808	37	1	8	1	13.1	5.34	0.05	1.25	
gi|23615667	proteasome subunit alpha type-4, putative	33	27930	5	1	4	1	20.3	5.85	0.12	35	27930	6	1	3	1	13	5.85	0.13	1.08	
gi|225631627	conserved Plasmodium protein, unknown function	33	207703	19	1	14	1	9.2	9.22	0.02	32	207703	18	1	12	1	7.1	9.22	0.02		
gi|124808276	rhoptry-associated protein 1	33	89996	6	1	6	1	12.1	6.67	0.04	-	-	-	-	-	-	-	-	-	Down-Detect	Down
gi|23476993	Plasmodium exported protein (hyp8), unknown function	33	28216	5	1	2	1	6.6	9.02	0.12	35	28216	5	1	4	1	16.9	9.02	0.13	1.08	
gi|23504954	dynein heavy chain, putative	32	720134	90	1	48	1	9.7	6.18	0	-	-	-	-	-	-	-	-	-	Down-Detect	Down
gi|225632293	Plasmodium exported protein, unknown function	32	36390	6	1	6	1	22.2	5.74	0.09	-	-	-	-	-	-	-	-	-	Down-Detect	Down
gi|23498950	zinc finger, C3HC4 type, putative	32	253816	52	1	28	1	15.5	8.32	0.01	-	-	-	-	-	-	-	-	-	Down-Detect	Down
gi|13509187	putative Rab7 GTPase	32	23773	6	1	4	1	28.6	7.55	0.3	46	23773	7	2	5	2	24.3	7.55	0.32	1.07	
gi|23615267	conserved Plasmodium protein, unknown function	31	34955	13	1	6	1	19.1	9.22	0.1	-	-	-	-	-	-	-	-	-	Down-Detect	Down
gi|124808655	surface protein P113	31	112505	19	1	7	1	8.6	4.49	0.03	-	-	-	-	-	-	-	-	-	Down-Detect	Down
gi|258597176	erythrocyte membrane protein 1, PfEMP1	31	248280	62	3	18	1	10.7	5.47	0.01	31	248280	83	3	32	1	17.1	5.47	0.01		
gi|23504621	deoxyribodipyrimidine photo-lyase, putative	31	129117	21	3	9	1	9.8	9.22	0.03	31	129117	26	3	10	1	9.3	9.22	0.03		
gi|23615206	conserved Plasmodium protein, unknown function	31	56775	11	3	3	1	9.6	9.53	0.06	31	56775	12	3	3	1	6.9	9.53	0.06		
gi|258597334	26S proteasome regulatory subunit RPN7,putative	31	46595	9	1	5	1	13	6.38	0.07	-	-	-	-	-	-	-	-	-	Down-Detect	Down
gi|225632017	conserved Plasmodium protein, unknown function	30	696614	108	1	53	1	11	7.88	0	39	696614	98	1	43	1	8.4	7.88	0		

gi|23615391	60S ribosomal protein L23	30	22079	37	0	4	0	23.7	10.27	0.15	22	22079	52	0	7	0	36.3	10.27	0.16	1.07	
gi|225632182	conserved Plasmodium protein, unknown function	30	126994	9	1	6	1	6.5	5.28	0.03	-	-	-	-	-	-	-	-	-	Down-Detect	Down
gi|23498915	conserved Plasmodium protein, unknown function	30	36570	2	1	2	1	6.3	8.79	0.09	-	-	-	-	-	-	-	-	-	Down-Detect	Down
gi|23615173	conserved Plasmodium protein, unknown function	30	95550	44	0	8	0	14.8	9.45	0.03	22	95550	50	0	7	0	9.4	9.45	0.04	1.33	
gi|23498906	metallo-hydrolase/oxidoreductase, putative	30	119584	15	1	12	1	15.3	7.01	0.03	-	-	-	-	-	-	-	-	-	Down-Detect	Down
gi|23615182	conserved Plasmodium protein, unknown function	30	1111079	188	0	112	0	15.6	9.12	0	-	-	-	-	-	-	-	-	-	Down-Detect	Down
gi|225632238	conserved Plasmodium protein, unknown function	30	36234	14	1	8	1	29.2	4.11	0.09	28	36234	11	1	6	1	20.3	4.11	0.21	2.33	Up
gi|58176834	Chain A, D-ribulose-5-phosphate 3-epimerase, Putative	30	25532	3	1	3	1	30.4	6.07	0.13	41	25532	3	1	3	1	16.3	6.07	0.14	1.08	
gi|124809637	conserved Plasmodium protein, unknown function	29	442796	57	1	30	1	9.7	8.64	0.01	21	442796	63	0	30	0	9.6	8.64	0.01		
gi|23498938	DNA (cytosine-5)-methyltransferase	29	83731	7	0	4	0	7.4	8.74	0.04	-	-	-	-	-	-	-	-	-	Down-Detect	Down
gi|408535927	Chain A, Apicoplast Tic22, Putative	29	33232	51	1	25	1	37.3	9.24	0.1	41	33232	41	2	18	1	33.3	9.24	0.11	1.10	
gi|225631857	conserved Plasmodium protein, unknown function	29	138544	28	0	13	0	15.5	8.44	0.02	28	138544	22	0	9	0	10.3	8.44	0.03	1.50	
gi|23504582	asparagine--tRNA ligase	29	85195	18	1	9	1	14.4	9.01	0.04	29	85195	19	2	9	1	15	9.01	0.04		
gi|23615371	3~,5~-cyclic nucleotide phosphodiesterase,putative	29	133084	21	1	7	1	5.3	7.47	0.02	-	-	-	-	-	-	-	-	-	Down-Detect	Down
gi|3649757	conserved Plasmodium protein, unknown function	29	202018	78	0	21	0	15.7	8.3	0.02	28	202018	77	0	18	0	15.3	8.3	0.02		
gi|258597702	40S ribosomal protein S25	29	11656	7	1	6	1	55.2	10.12	0.68	33	11656	3	1	3	1	33.3	10.12	0.31	0.46	Down
gi|23615215	U3 small nucleolar RNA-associated protein 6,putative	29	120650	7	0	4	0	4.1	5.51	0.03	-	-	-	-	-	-	-	-	-	Down-Detect	Down
gi|124804079	exported protein 1	28	17285	2	1	2	1	17.3	5.64	0.2	42	17285	4	1	1	1	11.1	5.64	0.21	1.05	
gi|124801337	vacuolar protein sorting-associated protein 45,putative	27	86202	21	0	3	0	3.2	6.51	0.04	33	86202	24	1	3	1	3.3	6.51	0.04		
gi|74876421	RecName: Full=ADP-ribosylation factor 1; Short=pfARF1	26	20899	14	0	5	0	28.2	5.83	0.16	52	20899	14	1	4	1	17.7	5.83	0.17	1.06	
gi|46362284	conserved Plasmodium protein, unknown function	26	25357	14	0	3	0	10	9.82	0.13	30	25357	16	0	5	0	24.4	9.82	0.14	1.08	
gi|7264037	erythrocyte membrane protein 1, PfEMP1	26	250321	42	0	24	0	12.2	5.29	0.01	-	-	-	-	-	-	-	-	-	Down-Detect	Down
gi|124804967	Plasmodium exported protein, unknown function	25	51125	10	0	6	0	14.7	5.66	0.06	-	-	-	-	-	-	-	-	-	Down-Detect	Down
gi|124804772	60S ribosomal protein L35ae, putative	25	16255	5	0	4	0	26.4	10.55	0.47	-	-	-	-	-	-	-	-	-	Down-Detect	Down
gi|124808373	conserved Plasmodium protein, unknown function	25	390446	53	0	29	0	10.2	9.23	0.01	20	390446	55	0	31	0	11.7	9.23	0.02	2.00	Up
gi|124809878	transcription factor with AP2 domain(s)	24	161410	13	0	9	0	8.2	8.98	0.02	-	-	-	-	-	-	-	-	-	Down-Detect	Down
gi|124806892	FK506-binding protein (FKBP)-type peptidyl-prolyl isomerase	24	34805	8	0	5	0	24.3	5.36	0.1	37	34805	6	1	4	1	16.4	5.36	0.1		
gi|23504953	mitochondrial carrier protein, putative	24	141981	52	0	17	0	15.3	9.63	0.02	-	-	-	-	-	-	-	-	-	Down-Detect	Down
gi|23504600	RNA pseudouridylate synthase, putative	24	1186842	238	0	98	0	12.2	7.36	0	18	1186842	242	0	94	0	11.2	7.36	0		
gi|17148533	Ran-binding protein	24	33176	4	0	4	0	16.4	4.92	0.1	41	33176	3	2	2	1	10	4.92	0.22	2.20	Up
gi|23505262	Plasmodium exported protein (PHISTc), unknown function	24	45472	6	0	6	0	16.4	9.71	0.07	-	-	-	-	-	-	-	-	-	Down-Detect	Down
gi|6562716	conserved Plasmodium protein, unknown function	24	229981	19	0	10	0	5.4	6.44	0.01	-	-	-	-	-	-	-	-	-	Down-Detect	Down
gi|258549210	conserved Plasmodium protein, unknown function	24	30341	5	0	4	0	11.5	7.64	0.11	-	-	-	-	-	-	-	-	-	Down-Detect	Down
gi|258597832	conserved Plasmodium protein, unknown function	23	145360	26	0	10	0	9.4	8.39	0.02	-	-	-	-	-	-	-	-	-	Down-Detect	Down
gi|74862955	RecName: Full=Origin recognition complex subunit 1; Short=PfORC1	23	138653	11	0	6	0	7.2	9.52	0.02	22	138653	15	0	12	0	14.6	9.52	0.03	1.5	
gi|23615256	meiosis-specific nuclear structural protein 1,putative	23	61194	9	0	7	0	11.1	8.91	0.05	24	61194	7	0	3	0	5.3	8.91	0.06	1.2	
gi|3649767	SECIS-binding protein 2, putative	23	71419	6	0	6	0	11	9.94	0.05	24	71419	14	0	11	0	16.9	9.94	0.05		
gi|23498865	60S ribosomal protein L11a, putative	23	20215	4	0	3	0	13.3	10.1	0.17	21	20215	2	0	2	0	6.9	10.1	0.18	1.05882353	
gi|23615806	secreted ookinete protein, putative	23	135752	55	0	8	0	8.5	4.66	0.02	24	135752	77	0	11	0	10.7	4.66	0.03	1.5	
gi|1575675	rab6	23	27914	3	0	3	0	17.5	7.63	0.12	40	27914	6	1	5	1	28.3	7.63	0.13	1.08333333	
gi|23504877	conserved Plasmodium protein, unknown function	23	225390	36	0	18	0	10.5	6.01	0.01	23	225390	40	0	14	0	9.4	6.01	0.02	2	Up
gi|23499018	magnesium transporter, putative	23	109581	8	0	6	0	10	9.53	0.03	-	-	-	-	-	-	-	-	-	Down-Detect	Down
gi|23510645	conserved Plasmodium protein, unknown function	23	65626	7	0	5	0	11.4	9.65	0.05	-	-	-	-	-	-	-	-	-	Down-Detect	Down
gi|23615192	DNA-directed RNA polymerase II subunit RPB11,putative	23	14116	3	0	3	0	31	8.37	0.24	-	-	-	-	-	-	-	-	-	Down-Detect	Down
gi|258597760	conserved Plasmodium membrane protein, unknown function	22	682022	120	0	62	0	13.8	8.86	0	-	-	-	-	-	-	-	-	-	Down-Detect	Down
gi|225632153	cytoskeleton associated protein, putative	22	103523	26	0	6	0	7.9	6.43	0.03	-	-	-	-	-	-	-	-	-	Down-Detect	Down
gi|225631936	MORN repeat protein, putative	22	519893	71	0	33	0	9.9	9.15	0.01	28	519893	83	0	37	0	10.3	9.15	0.01		
gi|124808756	conserved Plasmodium protein, unknown function	22	192484	87	0	17	0	13.2	9.67	0.02	28	192484	95	0	23	0	16.8	9.67	0.02		
gi|23615179	sodium/hydrogen exchanger, Na+, H+ antiporter	22	225940	39	0	17	0	11.4	8.68	0.01	28	225940	36	0	15	0	10.4	8.68	0.02	2	Up
gi|124804234	autophagy-related protein 7, putative	22	156530	36	0	18	0	18.2	6.05	0.02	28	156530	32	0	14	0	15.3	6.05	0.02		
gi|124806636	conserved Plasmodium protein, unknown function	22	212627	24	0	15	0	11.1	4.96	0.02	28	212627	21	0	14	0	10.2	4.96	0.02		
gi|23504575	conserved Plasmodium protein, unknown function	22	369953	36	0	19	0	6.7	5.29	0.01	28	369953	44	0	24	0	8.5	5.29	0.01		
gi|124809084	conserved Plasmodium protein, unknown function	22	152167	16	0	9	0	7.9	8.42	0.02	28	152167	15	0	10	0	7.7	8.42	0.02		
gi|124805631	conserved Plasmodium protein, unknown function	22	208541	26	0	16	0	13.1	8.3	0.02	28	208541	29	0	19	0	13.7	8.3	0.02		

gi|124802600	conserved Plasmodium protein, unknown function	22	190279	24	0	14	0	10.9	8.29	0.02	28	190279	14	0	8	0	5.5	8.29	0.02		
gi|124804432	conserved Plasmodium protein, unknown function	22	133324	24	0	9	0	10.8	5.18	0.02	28	133324	27	0	11	0	13.5	5.18	0.03	1.5	
gi|124804384	conserved Plasmodium protein, unknown function	22	118257	19	0	9	0	10.7	8.84	0.03	28	118257	20	0	11	0	14.6	8.84	0.03		
gi|23504862	eukaryotic translation initiation factor 3 subunit E, putative	22	61379	13	0	2	0	3.1	7.08	0.05	28	61379	12	0	3	0	4.8	7.08	0.06	1.2	
gi|225632254	rRNA-processing protein FCF1, putative	22	23145	8	0	2	0	9.1	9.63	0.15	-	-	-	-	-	-	-	-	-	Down-Detect	Down
gi|225631798	conserved protein, unknown function	22	296598	49	0	26	0	15.4	9.02	0.01	-	-	-	-	-	-	-	-	-	Down-Detect	Down
gi|124806350	subpellicular microtubule protein 2, putative	22	30171	4	0	2	0	11.6	9.31	0.11	18	30171	5	0	3	0	16.3	9.31	0.12	1.09090909	
gi|124809790	conserved Plasmodium protein, unknown function	22	233153	20	0	13	0	7.5	7.99	0.01	-	-	-	-	-	-	-	-	-	Down-Detect	Down
gi|46362277	conserved Plasmodium protein, unknown function	22	334204	57	0	38	0	18.4	8.49	0.01	-	-	-	-	-	-	-	-	-	Down-Detect	Down
gi|225632011	heat shock protein 110, putative	21	108119	14	0	5	0	6.9	5.5	0.03	27	108119	18	0	11	0	12.7	5.5	0.03		
gi|124809582	M17 leucyl aminopeptidase	21	67778	25	0	13	0	24.3	8.78	0.05	33	67778	23	0	10	0	22.1	8.78	0.05		
gi|46362309	conserved Plasmodium protein, unknown function	21	324818	42	0	20	0	9.3	6.62	0.01	22	324818	51	0	24	0	10.5	6.62	0.02	2	Up
gi|124806590	conserved Plasmodium protein, unknown function	21	333978	48	0	29	0	12.4	9.16	0.01	-	-	-	-	-	-	-	-	-	Down-Detect	Down
gi|258597800	conserved Plasmodium protein, unknown function	21	152700	10	0	8	0	6.3	7.78	0.02	-	-	-	-	-	-	-	-	-	Down-Detect	Down
gi|14530178	Krueppel-like protein	21	151550	10	0	8	0	6.2	7.89	0.02	-	-	-	-	-	-	-	-	-	Down-Detect	Down
gi|124810028	conserved Plasmodium protein, unknown function	21	118694	22	0	7	0	10.1	8.96	0.06	-	-	-	-	-	-	-	-	-	Down-Detect	Down
gi|124805005	erythrocyte membrane protein 1, PfEMP1	21	358992	66	0	39	0	15.5	5.63	0.01	-	-	-	-	-	-	-	-	-	Down-Detect	Down
gi|296005130	Pfmc-2TM Maurer~s cleft two transmembrane protein	21	27380	13	0	7	0	24.7	9.4	0.12	22	27380	11	0	5	0	19.9	9.4	0.28	2.33333333	Up
gi|23498719	Pfmc-2TM Maurer~s cleft two transmembrane protein	21	27539	9	0	3	0	12.3	9.59	0.12	21	27539	12	0	5	0	20.9	9.59	0.13	1.08333333	
gi|23498897	conserved Plasmodium protein, unknown function	21	66258	30	0	5	0	12.3	8.46	0.05	-	-	-	-	-	-	-	-	-	Down-Detect	Down
gi|124802749	tRNA N6-adenosine threonylcarbamoyltransferase	20	69530	12	0	8	0	15.2	7.49	0.05	-	-	-	-	-	-	-	-	-	Down-Detect	Down
gi|23505246	vacuolar protein sorting-associated protein 33,putative	20	135894	52	0	7	0	6.6	4.8	0.02	-	-	-	-	-	-	-	-	-	Down-Detect	Down
gi|225631933	conserved Plasmodium protein, unknown function	20	418313	55	0	37	0	14.9	6.69	0.01	-	-	-	-	-	-	-	-	-	Down-Detect	Down
gi|225631966	conserved Plasmodium protein, unknown function	20	404851	84	0	45	0	15.7	6.56	0.01	16	404851	74	0	35	0	11.1	6.56	0.01		
gi|23498787	60S ribosomal protein L34	20	17340	1	0	1	0	6	10.77	0.2	-	-	-	-	-	-	-	-	-	Down-Detect	Down
gi|224591371	conserved Plasmodium protein, unknown function	20	109211	23	0	13	0	18.7	6.62	0.03	-	-	-	-	-	-	-	-	-	Down-Detect	Down
gi|74864044	RecName: Full=NAD-dependent protein deacetylase Sir2B; AltName: Full=Regulatory protein SIR2 homolog B; AltName: Full=SIR2-like protein B	20	154624	19	0	12	0	10.1	8.15	0.02	-	-	-	-	-	-	-	-	-	Down-Detect	Down
gi|13375179	putative GTPase	20	22872	4	0	2	0	10	6.18	0.15	-	-	-	-	-	-	-	-	-	Down-Detect	Down
gi|124808388	AAA family ATPase, putative	20	142737	19	0	11	0	9	9.27	0.02	-	-	-	-	-	-	-	-	-	Down-Detect	Down
gi|124804419	conserved Plasmodium protein, unknown function	20	330099	31	0	25	0	12.1	6.16	0.01	-	-	-	-	-	-	-	-	-	Down-Detect	Down
gi|124808513	metacaspase-like protein	20	264761	31	0	18	0	9.3	9.31	0.01	-	-	-	-	-	-	-	-	-	Down-Detect	Down
gi|23498907	conserved Plasmodium protein, unknown function	19	260606	32	0	20	0	11.6	5.49	0.01	22	260606	45	0	22	0	12.1	5.49	0.03	3	Up
gi|225631649	transcription factor with AP2 domain(s)	19	399922	46	0	21	0	8	5.68	0.01	-	-	-	-	-	-	-	-	-	Down-Detect	Down
gi|258597770	dynein-related AAA-type ATPase, putative	19	970399	116	0	76	0	12.1	8.07	0	-	-	-	-	-	-	-	-	-	Down-Detect	Down
gi|225632063	conserved Plasmodium protein, unknown function	19	346585	41	0	27	0	9.9	7.11	0.01	-	-	-	-	-	-	-	-	-	Down-Detect	Down
gi|23505158	conserved Plasmodium protein, unknown function	19	208459	17	0	10	0	7.7	7.88	0.02	-	-	-	-	-	-	-	-	-	Down-Detect	Down
gi|124802000	hypothetical protein PF3D7_1004100	19	141831	42	0	12	0	13.8	8.61	0.02	-	-	-	-	-	-	-	-	-	Down-Detect	Down
gi|124808735	nucleolar GTP-binding protein 2, putative	19	57136	10	0	6	0	9.7	9.72	0.06	23	57136	8	0	4	0	7.4	9.72	0.06		
gi|225685560	6-cysteine protein	19	114662	6	0	6	0	7.5	6.28	0.03	-	-	-	-	-	-	-	-	-	Down-Detect	Down
gi|124809716	conserved Plasmodium protein, unknown function	19	176123	11	0	9	0	7.6	5.42	0.02	-	-	-	-	-	-	-	-	-	Down-Detect	Down
gi|46361233	HECT-domain (ubiquitin-transferase), putative	19	1205255	201	0	102	0	12	8.19	0.01	25	1205255	228	0	120	0	13.9	8.19	0.01		
gi|124808084	COBW domain-containing protein 1, putative	19	67839	2	0	2	0	7.3	6.04	0.05	-	-	-	-	-	-	-	-	-	Down-Detect	Down
gi|3758839	P-type ATPase, putative	19	217882	25	0	15	0	10.2	8.09	0.02	23	217882	18	0	9	0	6.9	8.09	0.02		
gi|124801012	serine repeat antigen 7	19	109569	13	0	8	0	12.2	5.57	0.03	23	109569	20	0	12	0	17.1	5.57	0.03		
gi|4493893	conserved Plasmodium protein, unknown function	19	474832	98	0	39	0	12.7	8.71	0.01	-	-	-	-	-	-	-	-	-	Down-Detect	Down
gi|46361095	conserved protein, unknown function	19	30831	4	0	4	0	26.3	5.62	0.11	-	-	-	-	-	-	-	-	-	Down-Detect	Down
gi|124809249	conserved Plasmodium protein, unknown function	19	25471	30	0	4	0	20.8	9.37	0.13	-	-	-	-	-	-	-	-	-	Down-Detect	Down
gi|46361088	conserved Plasmodium protein, unknown function	18	123646	71	0	9	0	11.1	5.86	0.05	-	-	-	-	-	-	-	-	-	Down-Detect	Down
gi|23505265	Plasmodium exported protein, unknown function	18	31267	2	0	1	0	3.1	9.69	0.11	-	-	-	-	-	-	-	-	-	Down-Detect	Down
gi|3649758	T-complex protein 1 subunit eta	18	59540	16	0	10	0	26	5.45	0.06	17	59540	17	0	7	0	16.5	5.45	0.06		
gi|124801939	tryptophan-rich antigen 3	18	118597	12	0	8	0	9.8	5.41	0.03	22	118597	10	0	5	0	6.8	5.41	0.03		
gi|258597255	SET domain protein, putative	18	94234	22	0	9	0	12.5	4.47	0.04	-	-	-	-	-	-	-	-	-	Down-Detect	Down
gi|124806112	phospholipid-transporting ATPase, putative	18	190326	22	0	7	0	5	8.74	0.02	-	-	-	-	-	-	-	-	-	Down-Detect	Down
gi|124804153	conserved Plasmodium protein, unknown function	18	153603	33	0	10	0	10	6.77	0.02	19	153603	39	0	14	0	11.8	6.77	0.02		

gi|730436	RecName: Full=Dihydroorotate dehydrogenase (quinone), mitochondrial; Short=DHOdehase; AltName: Full=Dihydroorotate oxidase; Flags: Precursor	18	65517	13	0	8	0	13.7	9.14	0.05	-	-	-	-	-	-	-	-	-	Down-Detect	Down
gi|23498946	conserved Plasmodium membrane protein, unknown function	17	670693	56	0	34	0	8.1	9.3	0	-	-	-	-	-	-	-	-	-	Down-Detect	Down
gi|23498781	conserved Plasmodium protein, unknown function	17	407134	70	0	32	0	9.2	9.85	0.01	-	-	-	-	-	-	-	-	-	Down-Detect	Down
gi|225632037	conserved Plasmodium protein, unknown function	17	439635	33	0	22	0	7.9	7.81	0.01	26	439635	27	0	19	0	6.7	7.81	0.01		
gi|225631835	nitric oxide synthase, putative	17	92122	19	0	9	0	14.1	9.01	0.04	-	-	-	-	-	-	-	-	-	Down-Detect	Down
gi|46361075	leucine-rich repeat protein	17	220157	47	0	29	0	17.8	5.56	0.01	-	-	-	-	-	-	-	-	-	Down-Detect	Down
gi|258597622	conserved Plasmodium protein, unknown function	16	142616	28	0	14	0	14	9.55	0.02	-	-	-	-	-	-	-	-	-	Down-Detect	Down
gi|258597535	60 kDa chaperonin	16	81434	11	0	8	0	13.6	4.97	0.04	30	81434	14	1	9	1	18.9	4.97	0.09	2.25	Up
gi|258597961	cysteine repeat modular protein 4	16	700473	116	0	54	0	11.6	8.43	0	29	700473	102	0	53	0	12.2	8.43	0		
gi|23505139	RNA-binding protein, putative	16	22965	9	0	4	0	15.8	9.51	0.15	-	-	-	-	-	-	-	-	-	Down-Detect	Down
gi|7670012	rifin	16	39495	7	0	2	0	5.2	9.15	0.08	16	39495	10	0	4	0	16.9	9.15	0.09	1.125	
gi|3764022	ATP-dependent RNA helicase DHX57, putative	15	267066	54	0	24	0	12.7	8.4	0.01	-	-	-	-	-	-	-	-	-	Down-Detect	Down
gi|23498174	pre-mRNA-processing-splicing factor 8, putative	15	366166	21	0	18	0	6.2	8.79	0.01	-	-	-	-	-	-	-	-	-	Down-Detect	Down
gi|23504539	conserved Plasmodium protein, unknown function	15	89840	16	0	11	0	13.1	9.52	0.04	-	-	-	-	-	-	-	-	-	Down-Detect	Down
gi|258549051	DnaJ protein, putative	15	94626	13	0	11	0	14.6	6.66	0.03	-	-	-	-	-	-	-	-	-	Down-Detect	Down
gi|23504963	conserved Plasmodium protein, unknown function	15	69116	27	0	8	0	17.3	9.51	0.05	-	-	-	-	-	-	-	-	-	Down-Detect	Down
gi|258597286	palmitoyltransferase, putative	15	34757	4	0	3	0	19.8	8.24	0.1	-	-	-	-	-	-	-	-	-	Down-Detect	Down
gi|258597253	glycerol-3-phosphate dehydrogenase, putative	15	44846	8	0	4	0	14.7	8.81	0.07	-	-	-	-	-	-	-	-	-	Down-Detect	Down
gi|46361274	erythrocyte membrane protein 1, PfEMP1	15	456629	57	0	42	0	14.5	5.92	0.01	-	-	-	-	-	-	-	-	-	Down-Detect	Down
gi|124803766	conserved Plasmodium protein, unknown function	14	709774	62	0	36	0	8	9.02	0	-	-	-	-	-	-	-	-	-	Down-Detect	Down
gi|124802097	conserved Plasmodium protein, unknown function	14	452722	49	0	36	0	12.1	8.74	0.01	22	452722	34	0	24	0	8.9	8.74	0.01		
gi|23499052	exonuclease, putative	14	48070	21	0	9	0	20.7	8.91	0.07	13	48070	25	0	10	0	34.2	8.91	0.07		
gi|23615467	60S ribosomal protein L23, putative	14	14983	5	0	3	0	25.9	9.9	0.23	39	14983	9	1	7	1	62.6	9.9	0.24	1.04347826	
gi|124804504	heat shock protein 70	-	-	-	-	-	-	-	-	-	228	73252	29	10	17	5	30.5	6.51	0.39	Up-Detect	Up
gi|124808181	plasmepsin III	-	-	-	-	-	-	-	-	-	202	51661	20	6	13	4	35.9	8.05	0.69	Up-Detect	Up
gi|47169189	Chain A, Uridine Phosphorylase, Putative	-	-	-	-	-	-	-	-	-	134	27745	18	4	9	3	39.5	6.32	0.44	Up-Detect	Up
gi|23615408	phosphoribosylpyrophosphate synthetase	-	-	-	-	-	-	-	-	-	123	49352	13	4	7	3	19.9	9.39	0.32	Up-Detect	Up
gi|10129955	S-adenosylmethionine synthetase	-	-	-	-	-	-	-	-	-	109	44816	12	3	10	3	31.1	6.28	0.25	Up-Detect	Up
gi|23615594	DNA/RNA-binding protein Alba 2	-	-	-	-	-	-	-	-	-	99	24969	4	2	2	1	10.4	7.68	0.14	Up-Detect	Up
gi|322812543	Chain A, Glucose-6-phosphate isomerase	-	-	-	-	-	-	-	-	-	80	69517	20	4	9	2	20.9	6.9	0.1	Up-Detect	Up
gi|124809020	60S ribosomal protein L14, putative	-	-	-	-	-	-	-	-	-	65	19285	5	2	2	1	7.3	10.2	0.19	Up-Detect	Up
gi|124810100	40S ribosomal protein S28e, putative	-	-	-	-	-	-	-	-	-	65	7489	5	3	3	2	46.3	10.83	1.27	Up-Detect	Up
gi|13397937	putative Rab2 GTPase	-	-	-	-	-	-	-	-	-	60	24394	4	2	2	1	12.7	6.33	0.15	Up-Detect	Up
gi|46361134	60S ribosomal protein L27a, putative	-	-	-	-	-	-	-	-	-	59	16712	7	2	3	1	26.4	10.54	0.22	Up-Detect	Up
gi|225631680	serine/arginine-rich splicing factor 1	-	-	-	-	-	-	-	-	-	51	34540	6	2	5	1	12.8	10.61	0.1	Up-Detect	Up
gi|45478047	macrophage migration inhibitory factor-like protein	-	-	-	-	-	-	-	-	-	50	12836	6	1	4	1	44	6.15	0.28	Up-Detect	Up
gi|23504502	rhoptry-associated protein 2	-	-	-	-	-	-	-	-	-	49	46709	17	1	9	1	25.6	8.9	0.08	Up-Detect	Up
gi|4493905	40S ribosomal protein S23, putative	-	-	-	-	-	-	-	-	-	43	16120	15	1	4	1	21.4	10.83	0.22	Up-Detect	Up
gi|23615434	proteasome subunit beta type-7, putative	-	-	-	-	-	-	-	-	-	41	29942	2	1	2	1	8.5	7.98	0.12	Up-Detect	Up
gi|46362290	DNA repair protein RAD50, putative	-	-	-	-	-	-	-	-	-	39	267786	76	1	35	1	17.6	8.78	0.01	Up-Detect	Up
gi|23615274	Plasmodium exported protein (hyp12), unknown function	-	-	-	-	-	-	-	-	-	39	45997	4	1	3	1	7.3	4.62	0.08	Up-Detect	Up
gi|23510644	centrin-1	-	-	-	-	-	-	-	-	-	39	19587	14	1	2	1	11.9	4.78	0.18	Up-Detect	Up
gi|23498308	Plasmodium exported protein (PHISTb), unknown function	-	-	-	-	-	-	-	-	-	37	35939	3	1	3	1	15.5	8.75	0.1	Up-Detect	Up
gi|7340797	cytoadherence linked asexual protein 3.2	-	-	-	-	-	-	-	-	-	36	167382	23	1	15	1	13	6.74	0.02	Up-Detect	Up
gi|4493872	cytoadherence linked asexual protein 3.1	-	-	-	-	-	-	-	-	-	36	167134	23	1	14	1	13.5	6.75	0.02	Up-Detect	Up
gi|124809004	conserved Plasmodium protein, unknown function	-	-	-	-	-	-	-	-	-	36	146396	21	1	10	1	9.9	9.2	0.02	Up-Detect	Up
gi|23499061	RNA-binding protein, putative	-	-	-	-	-	-	-	-	-	36	32421	2	1	2	1	9.1	9.11	0.11	Up-Detect	Up
gi|124803451	60S acidic ribosomal protein P1, putative	-	-	-	-	-	-	-	-	-	35	13006	6	0	2	0	25.4	4.57	0.28	Up-Detect	Up
gi|258596969	conserved Plasmodium protein, unknown function	-	-	-	-	-	-	-	-	-	34	14508	8	1	4	1	33.1	6.09	0.25	Up-Detect	Up
gi|124803615	casein kinase 2, alpha subunit	-	-	-	-	-	-	-	-	-	33	39865	9	1	5	1	23	8.9	0.09	Up-Detect	Up
gi|23498804	erythrocyte membrane protein 1, PfEMP1	-	-	-	-	-	-	-	-	-	33	248785	35	1	29	1	15.3	5.3	0.01	Up-Detect	Up
gi|46361054	conserved Plasmodium protein, unknown function	-	-	-	-	-	-	-	-	-	33	244517	23	1	16	1	8	6.06	0.01	Up-Detect	Up
gi|3694805	cytoadherence linked asexual protein, partial	-	-	-	-	-	-	-	-	-	33	160994	37	1	22	1	20.1	8.98	0.02	Up-Detect	Up
gi|23505252	cytoadherence linked asexual protein 9	-	-	-	-	-	-	-	-	-	33	160313	25	1	19	1	17.1	8.88	0.02	Up-Detect	Up

gi|124805343	erythrocyte membrane protein 1, PfEMP1	-	-	-	-	-	-	-	-	-	32	333038	101	1	43	1	18.1	6.39	0.01	Up-Detect	Up
gi|124806687	erythrocyte membrane protein 1, PfEMP1	-	-	-	-	-	-	-	-	-	32	300099	62	2	31	1	12.7	5.43	0.01	Up-Detect	Up
gi|46361276	erythrocyte membrane protein 1, PfEMP1	-	-	-	-	-	-	-	-	-	32	254458	101	2	22	1	12.1	5.66	0.01	Up-Detect	Up
gi|124801366	ATP synthase F1, alpha subunit	-	-	-	-	-	-	-	-	-	32	61731	17	1	7	1	16.2	8.72	0.06	Up-Detect	Up
gi|116668029	Chain A, Spermidine Synthase	-	-	-	-	-	-	-	-	-	32	32162	6	1	5	1	29.7	6.18	0.11	Up-Detect	Up
gi|258596882	serine repeat antigen 6	-	-	-	-	-	-	-	-	-	31	118761	18	2	13	1	16.3	5.89	0.03	Up-Detect	Up
gi|8439487	hypothetical protein, partial	-	-	-	-	-	-	-	-	-	31	23455	3	1	3	1	13.6	5.87	0.15	Up-Detect	Up
gi|34305467	membrane-associated histidine-rich protein, partial	-	-	-	-	-	-	-	-	-	31	26918	3	1	3	1	10.8	6.06	0.13	Up-Detect	Up
gi|15375389	60S ribosomal protein L7, putative	-	-	-	-	-	-	-	-	-	31	30504	5	1	3	1	18.7	10.35	0.12	Up-Detect	Up
gi|124805983	clathrin heavy chain, putative	-	-	-	-	-	-	-	-	-	31	232803	37	1	18	1	12.3	6	0.01	Up-Detect	Up
gi|23499287	hypothetical protein, partial	-	-	-	-	-	-	-	-	-	30	82064	9	1	5	1	10.3	9.28	0.04	Up-Detect	Up
gi|124805775	eukaryotic translation initiation factor 3 subunit A, putative	-	-	-	-	-	-	-	-	-	30	165959	11	1	7	1	8	6.38	0.02	Up-Detect	Up
gi|23505053	conserved Plasmodium protein, unknown function	-	-	-	-	-	-	-	-	-	29	85353	8	0	5	0	6.9	9.1	0.04	Up-Detect	Up
gi|23505032	elongation factor 1-beta	-	-	-	-	-	-	-	-	-	29	32007	4	0	4	0	25.4	4.94	0.11	Up-Detect	Up
gi|124804341	parasitophorous vacuolar protein 1	-	-	-	-	-	-	-	-	-	29	51919	17	1	6	1	24.3	4.97	0.07	Up-Detect	Up
gi|23498743	tRNA m5C-methyltransferase, putative	-	-	-	-	-	-	-	-	-	28	141140	25	1	12	1	12.3	6.35	0.02	Up-Detect	Up
gi|23504571	guanidine nucleotide exchange factor	-	-	-	-	-	-	-	-	-	28	303926	34	0	15	0	7.4	8.53	0.01	Up-Detect	Up
gi|258597440	antigen 332, DBL-like protein	-	-	-	-	-	-	-	-	-	28	688870	82	0	19	0	4.2	3.86	0.01	Up-Detect	Up
gi|124808633	conserved Plasmodium protein, unknown function	-	-	-	-	-	-	-	-	-	27	170332	39	0	17	0	13.7	5.89	0.02	Up-Detect	Up
gi|124808549	NOT family protein, putative	-	-	-	-	-	-	-	-	-	27	519499	79	0	40	0	11.8	6.88	0.01	Up-Detect	Up
gi|23504536	trafficking protein particle complex subunit 8,putative	-	-	-	-	-	-	-	-	-	27	328469	52	0	24	0	10.3	7.22	0.01	Up-Detect	Up
gi|23498839	eukaryotic translation initiation factor 3 subunit I, putative	-	-	-	-	-	-	-	-	-	27	37261	6	0	4	0	17.1	6.43	0.09	Up-Detect	Up
gi|356624409	Chain A, Translationally-controlled Tumor Protein Homolog	-	-	-	-	-	-	-	-	-	26	21594	12	0	5	0	19.7	4.9	0.17	Up-Detect	Up
gi|124806152	histone chaperone ASF1, putative	-	-	-	-	-	-	-	-	-	26	31527	5	0	3	0	11.7	4.37	0.11	Up-Detect	Up
gi|59797635	RecName: Full=Glycophorin-binding protein; AltName: Full=GBP-130	-	-	-	-	-	-	-	-	-	26	95786	8	0	7	0	14.9	5.02	0.04	Up-Detect	Up
gi|402550052	Chain A, Deoxyuridine 5~-triphosphate Nucleotidohydrolase, Putative	-	-	-	-	-	-	-	-	-	25	20626	7	0	4	0	27.6	6.59	0.17	Up-Detect	Up
gi|23504945	signal peptidase complex subunit 3, putative	-	-	-	-	-	-	-	-	-	25	22194	4	1	2	1	16.2	9.34	0.16	Up-Detect	Up
gi|225631760	transcription factor with AP2 domain(s),putative	-	-	-	-	-	-	-	-	-	24	237219	27	0	16	0	10.2	9	0.01	Up-Detect	Up
gi|124808263	alpha/beta hydrolase, putative	-	-	-	-	-	-	-	-	-	24	41458	8	0	4	0	13.8	6.51	0.09	Up-Detect	Up
gi|23504898	lysine-rich membrane-associated PHISTb protein	-	-	-	-	-	-	-	-	-	24	61048	8	0	6	0	10.6	9.34	0.06	Up-Detect	Up
gi|225631686	proteasome subunit beta type-1, putative	-	-	-	-	-	-	-	-	-	24	27251	2	0	1	0	9.6	6.59	0.13	Up-Detect	Up
gi|23615433	conserved Plasmodium protein, unknown function	-	-	-	-	-	-	-	-	-	24	319935	75	0	32	0	13.9	7.32	0.01	Up-Detect	Up
gi|23499096	conserved Plasmodium protein, unknown function	-	-	-	-	-	-	-	-	-	24	170170	23	0	8	0	7	6.4	0.02	Up-Detect	Up
gi|225632226	peptidase family C50, putative	-	-	-	-	-	-	-	-	-	23	697746	100	0	59	0	11.9	8.41	0	Up-Detect	Up
gi|124806110	DNA gyrase subunit A	-	-	-	-	-	-	-	-	-	23	143055	17	0	13	0	9.8	9.39	0.02	Up-Detect	Up
gi|124805429	high mobility group protein B1	-	-	-	-	-	-	-	-	-	23	11324	4	0	3	0	25.8	9.96	0.32	Up-Detect	Up
gi|124810337	conserved Plasmodium protein, unknown function	-	-	-	-	-	-	-	-	-	23	68624	15	0	5	0	9.7	8.96	0.05	Up-Detect	Up
gi|23505130	DNA-directed RNA polymerase II subunit RPB3,putative	-	-	-	-	-	-	-	-	-	23	38322	10	0	7	0	26.3	6.01	0.09	Up-Detect	Up
gi|23504860	proteasome maturation factor UMP1, putative	-	-	-	-	-	-	-	-	-	22	14594	1	0	1	0	5.6	5.27	0.25	Up-Detect	Up
gi|74862993	RecName: Full=Uncharacterized protein PFB0765w	-	-	-	-	-	-	-	-	-	22	166903	82	0	18	0	13.8	6.19	0.04	Up-Detect	Up
gi|8248757	DNA polymerase alpha catalytic subunit A	-	-	-	-	-	-	-	-	-	22	225260	23	0	21	0	14.2	8.55	0.02	Up-Detect	Up
gi|23615691	ubiquitin-conjugating enzyme, putative	-	-	-	-	-	-	-	-	-	21	22869	1	0	1	0	7.9	5.32	0.16	Up-Detect	Up
gi|258597812	conserved Plasmodium protein, unknown function	-	-	-	-	-	-	-	-	-	21	70994	30	0	9	0	19	9.15	0.05	Up-Detect	Up
gi|74929507	RecName: Full=Actin-1; AltName: Full=Actin I	-	-	-	-	-	-	-	-	-	21	41844	13	0	4	0	14.1	5.21	0.08	Up-Detect	Up
gi|124810134	conserved Plasmodium protein, unknown function	-	-	-	-	-	-	-	-	-	21	97606	9	0	6	0	10.3	9.6	0.04	Up-Detect	Up
gi|23498914	mitochondrial import inner membrane translocase subunit TIM14, putative	-	-	-	-	-	-	-	-	-	21	13044	3	0	3	0	15.7	10.09	0.28	Up-Detect	Up
gi|23498760	RAP protein, putative	-	-	-	-	-	-	-	-	-	21	142168	9	0	8	0	7.8	8.58	0.02	Up-Detect	Up
gi|23498806	Pfmc-2TM Maurer~s cleft two transmembrane protein	-	-	-	-	-	-	-	-	-	21	27311	7	0	3	0	21.2	9.57	0.13	Up-Detect	Up
gi|23615656	P-loop containing nucleoside triphosphate hydrolase, putative	-	-	-	-	-	-	-	-	-	21	304114	38	0	20	0	9	6.24	0.01	Up-Detect	Up
gi|23498260	conserved Plasmodium protein, unknown function	-	-	-	-	-	-	-	-	-	20	296268	16	0	11	0	6.7	5.86	0.01	Up-Detect	Up
gi|225631696	conserved Plasmodium protein, unknown function	-	-	-	-	-	-	-	-	-	20	1116481	120	0	82	0	11	9.37	0	Up-Detect	Up
gi|23498206	structural maintenance of chromosomes protein 3,putative	-	-	-	-	-	-	-	-	-	20	141137	27	0	20	0	20.7	6.48	0.02	Up-Detect	Up
gi|23615774	aldehyde reductase, putative	-	-	-	-	-	-	-	-	-	20	106055	26	0	14	0	15.9	9.18	0.03	Up-Detect	Up

gi|124802266	conserved Plasmodium protein, unknown function	-	-	-	-	-	-	-	-	-	20	173168	20	0	10	0	9.1	5.76	0.02	Up-Detect	Up
gi|124802886	U2 snRNA/tRNA pseudouridine synthase, putative	-	-	-	-	-	-	-	-	-	20	116647	16	0	10	0	12.3	8.58	0.03	Up-Detect	Up
gi|124802424	uncharacterized protein PF3D7_1018800	-	-	-	-	-	-	-	-	-	20	85496	7	0	4	0	5.2	9.49	0.04	Up-Detect	Up
gi|124805367	RESA-like protein with PHIST and DnaJ domains	-	-	-	-	-	-	-	-	-	20	107558	11	0	10	0	14	7.04	0.03	Up-Detect	Up
gi|23615579	aspartate carbamoyltransferase	-	-	-	-	-	-	-	-	-	20	43224	3	0	3	0	7.2	8.55	0.08	Up-Detect	Up
gi|23615157	erythrocyte membrane protein 1, PfEMP1	-	-	-	-	-	-	-	-	-	20	385533	83	0	37	0	13.8	5.62	0.01	Up-Detect	Up
gi|124804730	conserved Plasmodium protein, unknown function	-	-	-	-	-	-	-	-	-	20	120306	20	0	11	0	12.3	9.15	0.03	Up-Detect	Up
gi|225631639	histone acetyltransferase, putative	-	-	-	-	-	-	-	-	-	20	146635	21	0	8	0	7.9	6.29	0.02	Up-Detect	Up
gi|124804435	T-complex protein 1 subunit alpha	-	-	-	-	-	-	-	-	-	19	60223	6	0	4	0	9.7	6.65	0.06	Up-Detect	Up
gi|124805672	conserved Plasmodium protein, unknown function	-	-	-	-	-	-	-	-	-	19	25749	11	0	3	0	14	6.04	0.14	Up-Detect	Up
gi|23615411	conserved Plasmodium protein, unknown function	-	-	-	-	-	-	-	-	-	19	79471	51	0	5	0	9.4	8.88	0.04	Up-Detect	Up
gi|3758855	conserved Plasmodium protein, unknown function	-	-	-	-	-	-	-	-	-	18	402704	48	0	28	0	8.8	4.68	0.01	Up-Detect	Up
gi|23498756	inositol-phosphate phosphatase, putative	-	-	-	-	-	-	-	-	-	18	330496	62	0	25	0	10.1	7.09	0.01	Up-Detect	Up
gi|124802292	conserved Plasmodium protein, unknown function	-	-	-	-	-	-	-	-	-	18	267819	31	0	24	0	11.5	7.84	0.01	Up-Detect	Up
gi|124804827	conserved Plasmodium protein, unknown function	-	-	-	-	-	-	-	-	-	18	175302	29	0	16	0	10.7	6.41	0.02	Up-Detect	Up
gi|124803597	transcription factor with AP2 domain(s)	-	-	-	-	-	-	-	-	-	18	206747	19	0	14	0	12.4	5.63	0.02	Up-Detect	Up
gi|23499166	probable protein, unknown function	-	-	-	-	-	-	-	-	-	18	187594	11	0	8	0	4.9	9.38	0.02	Up-Detect	Up
gi|124803042	rifin	-	-	-	-	-	-	-	-	-	18	40212	9	0	9	0	29.5	8.67	0.09	Up-Detect	Up
gi|224591363	Pfmc-2TM Maurer~s cleft two transmembrane protein	-	-	-	-	-	-	-	-	-	18	27543	5	0	4	0	16.2	9.69	0.13	Up-Detect	Up
gi|124805437	conserved Plasmodium protein, unknown function	-	-	-	-	-	-	-	-	-	18	20310	2	0	2	0	4.7	9.22	0.18	Up-Detect	Up
gi|124806077	transcription factor with AP2 domain(s)	-	-	-	-	-	-	-	-	-	18	301724	21	0	14	0	8.9	6.89	0.01	Up-Detect	Up
gi|7768291	40S ribosomal protein S11, putative	-	-	-	-	-	-	-	-	-	18	18748	5	0	3	0	15.5	10.18	0.19	Up-Detect	Up
gi|3758843	conserved Plasmodium protein, unknown function	-	-	-	-	-	-	-	-	-	17	592883	77	0	51	0	12.6	9.05	0.01	Up-Detect	Up
gi|23504617	conserved Plasmodium protein, unknown function	-	-	-	-	-	-	-	-	-	17	331924	86	0	26	0	12	9.2	0.01	Up-Detect	Up
gi|23504632	conserved Plasmodium protein, unknown function	-	-	-	-	-	-	-	-	-	17	190407	20	0	10	0	7.3	9.71	0.02	Up-Detect	Up
gi|225632147	conserved Plasmodium protein, unknown function	-	-	-	-	-	-	-	-	-	16	196449	33	0	18	0	11	8.24	0.02	Up-Detect	Up
gi|7264041	pre-mRNA-splicing factor PRP46, putative	-	-	-	-	-	-	-	-	-	16	69529	6	0	3	0	6.7	8.6	0.05	Up-Detect	Up
gi|23498873	ferrodoxin reductase-like protein	-	-	-	-	-	-	-	-	-	16	72675	14	0	7	0	15	8.91	0.05	Up-Detect	Up
gi|23504638	phosphatidylinositol 3-kinase	-	-	-	-	-	-	-	-	-	16	255758	44	0	20	0	11.2	9.27	0.01	Up-Detect	Up
gi|258596863	protein kinase, putative	-	-	-	-	-	-	-	-	-	16	293584	57	0	26	0	13	6.84	0.01	Up-Detect	Up
gi|258596939	DnaJ protein, putative	-	-	-	-	-	-	-	-	-	16	117215	17	0	9	0	10.1	8.77	0.03	Up-Detect	Up
gi|225685561	conserved Plasmodium protein, unknown function	-	-	-	-	-	-	-	-	-	16	83028	10	0	7	0	13.2	8.83	0.04	Up-Detect	Up
gi|46361062	transketolase	-	-	-	-	-	-	-	-	-	16	75767	6	0	5	0	9.7	6.5	0.05	Up-Detect	Up
gi|46362240	rifin	-	-	-	-	-	-	-	-	-	15	41283	5	0	4	0	17.8	8.18	0.09	Up-Detect	Up
gi|23499205	rifin	-	-	-	-	-	-	-	-	-	15	43647	3	0	3	0	12.9	8.43	0.08	Up-Detect	Up
gi|225631825	50S ribosomal protein L3, apicoplast, putative	-	-	-	-	-	-	-	-	-	15	36251	4	0	4	0	14.2	9.95	0.1	Up-Detect	Up
gi|124809291	cleavage and polyadenylation specificity factor subunit 3, putative	-	-	-	-	-	-	-	-	-	15	101120	17	0	5	0	8.6	5.29	0.03	Up-Detect	Up
gi|3758842	cleavage and polyadenylation specificity factor,putative	-	-	-	-	-	-	-	-	-	15	118775	17	0	10	0	12.1	8.49	0.03	Up-Detect	Up
gi|23504588	peptidyl-prolyl cis-trans isomerase	-	-	-	-	-	-	-	-	-	15	86985	6	0	4	0	9.4	6.98	0.04	Up-Detect	Up
gi|124809181	conserved Plasmodium protein, unknown function	-	-	-	-	-	-	-	-	-	15	30461	3	0	2	0	11	5.08	0.12	Up-Detect	Up
gi|124810013	WD repeat-containing protein, putative	-	-	-	-	-	-	-	-	-	14	113162	10	0	7	0	8.6	5.51	0.03	Up-Detect	Up
gi|225631940	conserved Plasmodium protein, unknown function	-	-	-	-	-	-	-	-	-	14	737235	90	0	55	0	11.3	9.13	0	Up-Detect	Up
