# Supplementary material for: Proteomic analysis of Plasmodium falciparum response to isocryptolepine derivative
Source: PLoS One. 2019 Aug 8;14(8):e0220871. doi: 10.1371/journal.pone.0220871 (PMC6687117; doi:10.1371/journal.pone.0220871)
Supplement: S2 File — Total proteins were identified and quantified by Mascot daemon version 2.3.2 software. The protein database was collected from an NCBInr database (24 October 2018) specific to P. falciparum 3D7. (PDF) [file pone.0220871.s002.pdf]

Supplement 2 file. Protein identification from differential proteomics experiment replication 2

Total proteins were identified and quantified by Mascot daemon version 2.3.2 software.

Protein database was collected from NCBInr database (24 October 2018) specific to *P. falciparum* 3D7

Rep.2		Rep.2 DMSO									Rep.2 ICL-M									ICL-M	ICL-M
NCBI acc_no	All prot_desc	prot_score	prot_mass	prot_matc	prot_matc	prot_sequ	prot_sequ	prot_cove	prot_pi	emPAI	prot_score	prot_mass	prot_matc	prot_matc	prot_sequ	prot_sequ	prot_cove	prot_pi	emPAI	Fold change	Up/Down
gi|23499105	heat shock protein 70	5470	73868	238	180	35	27	47.7	5.51	5.39	5748	73868	247	190	33	26	45.5	5.51	3.64	0.68	
gi|23505079	heat shock protein 70	3530	72343	201	114	24	17	43.1	5.18	1.97	4141	72343	192	123	22	18	37.7	5.18	2.06	1.05	
gi|124810131	glyceraldehyde-3-phosphate dehydrogenase	2988	36612	82	66	17	15	56.4	7.59	4.76	2920	36612	81	68	21	17	62.3	7.59	6.44	1.35	
gi|23615698	elongation factor 1-alpha	2426	48928	175	107	17	12	44.7	9.12	2.49	2125	48928	160	89	16	13	43.3	9.12	2.48	1.00	
gi|50400239	RecName: Full=Enolase; AltName: Full=2-phospho-D-glycerate hydro-lyase; AltName: Full=2-phosphoglycerate dehydratase	2406	48647	93	58	23	16	51.3	6.21	3.99	2210	48647	84	54	27	20	57.8	6.21	6.27	1.57	
gi|371941768	Hsp70-x	2038	75006	98	75	7	6	10.8	5.59	0.44	2528	75006	117	89	13	7	23.1	5.59	0.54	1.23	
gi|23498766	heat shock protein 90	1751	86112	113	54	26	12	34.5	4.94	1.05	1977	86112	117	58	31	15	40.4	4.94	1.29	1.23	
gi|225632282	protein disulfide isomerase	1568	55479	90	60	17	11	35.8	5.56	1.51	1410	55479	79	48	14	7	30.4	5.56	1.01	0.67	
gi|46361043	ornithine aminotransferase	1029	46025	48	29	16	11	33.1	6.47	1.81	1571	46025	69	46	19	10	40.3	6.47	2.05	1.13	
gi|74920225	RecName: Full=Fructose-bisphosphate aldolase	983	40080	45	34	15	12	43.4	8.33	3.2	1054	40080	42	31	15	12	42.8	8.33	3.56	1.11	
gi|23615406	L-lactate dehydrogenase	942	34086	46	28	14	10	43.4	7.12	4.37	1047	34086	42	32	13	9	34.5	7.12	3.47	0.79	
gi|124809712	elongation factor 2	878	93462	39	22	13	5	19.7	6.36	0.34	1010	93462	37	21	15	5	19.8	6.36	0.19	0.56	
gi|344189571	Chain A, Phosphoglycerate Kinase	832	46264	52	25	26	8	54.2	7.82	1.79	739	46264	53	24	29	11	54	7.82	1.64	0.92	
gi|129926	RecName: Full=Phosphoglycerate kinase	832	45398	34	25	14	8	42.5	7.63	1.85	739	45398	40	24	19	11	51	7.63	1.69	0.91	
gi|124803934	GTP-binding nuclear protein RAN/TC4	783	24860	31	21	12	8	63.1	7.72	1.92	837	24860	36	21	14	8	65.4	7.72	4.21	2.19	Up
gi|23498286	40S ribosomal protein S19	569	19710	27	16	7	5	42.4	10.17	1.72	562	19710	26	16	6	5	38.8	10.17	2.55	1.48	
gi|124806075	endoplasmin, putative	557	94959	45	19	14	6	23.4	5.28	0.24	837	94959	47	25	14	6	25	5.28	0.23	0.96	
gi|124803500	histone H2B	517	13117	67	30	11	8	59.8	10.27	13.94	681	13117	98	45	12	10	66.7	10.27	40.99	2.94	Up
gi|31505529	histone H4, partial	478	11448	24	13	5	3	35	11.23	3.03	819	11448	41	26	9	6	67	11.23	9.86	3.25	Up
gi|258597456	nucleosome assembly protein	416	40463	20	13	4	1	11.8	4.76	0.18	397	40463	15	10	3	1	13.5	4.76	0.08	0.44	Down
gi|23615568	phosphoethanolamine N-methyltransferase	381	31024	17	13	6	5	24.8	5.43	0.72	311	31024	20	10	6	4	25.9	5.43	0.51	0.71	
gi|23499152	DNA/RNA-binding protein Alba 1	381	27242	34	15	8	4	34.3	10.58	1.09	208	27242	31	9	7	3	24.6	10.58	0.59	0.54	
gi|46361058	histone H3	350	15437	52	23	10	6	44.9	11.14	3.38	404	15437	63	24	10	5	44.9	11.14	3.06	0.91	
gi|124804377	60S ribosomal protein P0	336	34945	14	8	7	4	24.1	6.28	0.62	352	34945	14	8	10	4	37.7	6.28	0.58	0.94	
gi|23498886	40S ribosomal protein S5, putative	324	21849	11	6	5	3	32.8	9.67	0.58	363	21849	16	11	6	5	32.3	9.67	1.37	2.36	Up
gi|225631678	60S ribosomal protein L12, putative	320	18101	17	9	8	3	55.8	9.54	0.72	346	18101	13	9	5	2	35.8	9.54	0.41	0.57	
gi|4493980	peptidyl-prolyl cis-trans isomerase	300	18940	16	11	6	4	45	8.29	1.38	365	18940	20	13	5	4	29.8	8.29	1.68	1.22	
gi|225631753	cell division cycle protein 48 homologue,putative	272	92329	17	8	7	2	12.6	4.95	0.12	273	92329	32	9	16	2	26.1	4.95	0.11	0.92	
gi|23504556	60S ribosomal protein L4	268	46183	12	8	9	5	22.9	10.5	0.67	94	46183	15	3	11	3	34.3	10.5	0.32	0.48	Down
gi|225632253	14-3-3 protein	267	30174	26	12	10	5	43.9	4.86	1.18	476	30174	26	13	10	5	39.7	4.86	1.32	1.12	
gi|23505200	merozoite surface protein 1	248	195605	28	8	18	4	14.1	6.11	0.11	260	195605	26	10	18	7	11.4	6.11	0.12	1.09	
gi|23498770	heat shock protein 110	242	99902	36	6	10	2	14.4	5.54	0.07	240	99902	35	8	9	3	11.1	5.54	0.14	2.00	Up
gi|23504519	actin-depolymerizing factor 1	226	13732	9	6	3	2	33.6	7.66	0.6	272	13732	14	7	6	2	59	7.66	0.56	0.93	
gi|4494010	histone H2A variant, putative	225	16443	24	14	5	3	29.7	10.63	1.22	393	16443	28	19	5	3	32.3	10.63	1.57	1.29	
gi|124802054	DNA/RNA-binding protein Alba 3	218	11969	22	7	6	3	66.4	9.3	3.96	150	11969	20	6	8	3	82.2	9.3	3.58	0.90	
gi|46361129	histone H2A	214	14114	22	14	6	2	44.7	10.29	0.58	304	14114	27	17	6	2	48.5	10.29	0.92	1.59	
gi|45478047	macrophage migration inhibitory factor-like protein	212	12836	15	7	8	4	56.9	6.15	1.72	259	12836	17	8	8	4	56	6.15	1.58	0.92	
gi|23505159	conserved Plasmodium protein, unknown function	209	24683	15	10	6	4	34.1	5.49	0.97	228	24683	16	10	6	4	28.1	5.49	0.9	0.93	
gi|124808442	60S ribosomal protein L10, putative	197	25200	11	3	5	1	24.7	9.97	0.14	104	25200	11	2	6	1	30.1	9.97	0.13	0.93	
gi|75009812	RecName: Full=Triosephosphate isomerase; Short=TIM; AltName: Full=Triose-phosphate isomerase	186	27917	11	5	6	3	17.7	6.01	0.61	218	27917	11	10	5	5	16.5	6.01	0.97	1.59	
gi|124804166	splicing factor, putative	185	16002	6	4	1	1	13.1	5.1	0.5	269	16002	11	7	3	2	27.6	5.1	0.79	1.58	
gi|124810293	eukaryotic initiation factor 4A	184	45281	21	4	6	1	16.6	5.48	0.08	171	45281	21	4	9	2	32.9	5.48	0.24	3.00	Up
gi|124802119	adenylate kinase	183	27594	11	6	8	4	33.5	8.97	1.07	351	27594	23	15	10	8	43	8.97	1.81	1.69	
gi|124803863	cysteine proteinase falcipain 2a	183	55892	16	7	8	4	18.6	7.12	0.53	186	55892	20	8	9	5	20.5	7.12	0.5	0.94	
gi|11127605	heat shock protein hsp70 homologue Pfhsp70-3	179	71546	31	7	12	4	23.5	5.9	0.27	205	71546	34	8	16	5	31.8	5.9	0.37	1.37	
gi|23499115	high mobility group protein B2	176	11527	6	5	4	3	28.3	9.97	2.02	191	11527	9	6	3	2	28.3	9.97	1.2	0.59	
gi|124808810	60S ribosomal protein L21	174	18783	16	7	4	1	26.1	10.04	0.42	221	18783	25	9	5	1	29.2	10.04	0.39	0.93	
gi|124800689	knob-associated histidine-rich protein	173	71259	12	5	5	2	8.3	9.17	0.15	240	71259	17	8	5	2	8.1	9.17	0.15		

gi|74876423	RecName: Full=Tubulin beta chain; AltName: Full=Beta-tubulin	157	49719	10	4	7	3	23.8	4.73	0.23	235	49719	17	4	11	2	44.3	4.73	0.14	0.61	
gi|10129955	S-adenosylmethionine synthetase	157	44816	8	3	6	2	23.4	6.28	0.16	250	44816	8	6	6	4	13.7	6.28	0.43	2.69	Up
gi|124810100	40S ribosomal protein S28e, putative	147	7489	7	5	2	2	40.3	10.83	2.42	117	7489	6	4	3	2	40.3	10.83	2.24	0.93	
gi|23499261	1-cys peroxiredoxin	146	25148	16	5	7	1	29.1	6.31	0.3	120	25148	15	3	6	1	23.2	6.31	0.13	0.43	Down
gi|46361130	histone H3 variant, putative	142	15432	45	13	8	2	36.8	11.15	0.88	171	15432	61	16	8	2	36.8	11.15	1.23	1.40	
gi|47168426	Chain A, Uridine Phosphorylase, Putative	141	30441	14	4	7	2	35.5	5.83	0.25	-	-	-	-	-	-	-	-	-	Down-Detect	Down
gi|47169189	Chain A, Uridine Phosphorylase, Putative	141	27745	13	4	6	2	26.5	6.32	0.27	134	27745	15	5	8	3	31.2	6.32	0.58	2.15	Up
gi|124804024	phosphoglycerate mutase, putative	141	28752	13	5	9	3	36.8	8.3	0.42	142	28752	15	2	9	2	39.2	8.3	0.74	1.76	
gi|268612503	Chain A, Phosphoglycerate mutase	141	29716	12	5	8	3	35.7	8.26	0.4	142	29716	15	2	9	2	36.8	8.26	0.71	1.78	
gi|258596854	stevor	139	34204	14	6	3	1	13.8	8.76	0.1	138	34204	11	6	4	1	15.4	8.76	0.1		
gi|23504595	histamine-releasing factor	138	19967	5	3	3	1	16.4	4.58	0.18	134	19967	6	2	4	1	18.1	4.58	0.37	2.06	Up
gi|124803860	peptidyl-prolyl cis-trans isomerase	137	21717	18	6	10	4	51.8	7.1	1.14	207	21717	18	9	7	4	39	7.1	1.06	0.93	
gi|7768287	formate-nitrite transporter	136	34436	12	4	4	2	10.7	8.74	0.22	194	34436	18	7	4	2	10.7	8.74	0.32	1.45	
gi|312208188	Chain A, Endoplasmin homolog	135	32001	15	6	5	1	25.6	5.76	0.11	-	-	-	-	-	-	-	-	-	Down-Detect	Down
gi|23615388	60S ribosomal protein L6, putative	134	21588	6	4	3	3	17.9	9.88	0.59	19	21588	2	0	2	0	12.1	9.88	0.16	0.27	Down
gi|4493906	40S ribosomal protein S12, putative	128	15387	6	5	4	3	28.4	4.9	0.88	185	15387	9	6	5	3	33.3	4.9	0.82	0.93	
gi|258597201	40S ribosomal protein S4, putative	127	29753	5	3	3	2	16.1	10.09	0.25	108	29753	12	3	8	2	41.4	10.09	0.24	0.96	
gi|237640532	Chain A, HAP protein	125	37376	14	5	8	3	32.2	4.97	0.57	68	37376	11	4	3	2	8.4	4.97	0.19	0.33	Down
gi|124809606	40S ribosomal protein S5	122	29939	9	3	7	2	32.4	10.02	0.25	107	29939	6	2	6	2	30.9	10.02	0.24	0.96	
gi|124808771	60S ribosomal protein L5, putative	117	33977	5	4	4	3	18	9.78	0.35	79	33977	4	3	4	3	15	9.78	0.33	0.94	
gi|23615478	20 kDa chaperonin	116	29045	3	2	2	1	7	7.63	0.12	73	29045	4	1	3	1	14.3	7.63	0.12		
gi|225631960	40S ribosomal protein S19	116	16753	7	2	3	1	28.3	10.27	0.22	196	16753	11	6	3	1	24.1	10.27	0.2	0.91	
gi|23615606	DNA/RNA-binding protein Alba 4	116	42133	8	2	5	1	16.4	7.14	0.08	118	42133	9	3	4	1	15.1	7.14	0.08		
gi|23498814	histone H2B variant	116	13755	15	8	5	1	37.4	10.8	0.26	144	13755	21	11	7	2	51.2	10.8	0.56	2.15	Up
gi|23499195	acyl-CoA binding protein, putative	116	10768	9	4	4	1	62.2	7.68	0.8	121	10768	7	5	2	1	31.1	7.68	0.75	0.94	
gi|124810348	exported protein 2	113	33391	10	4	5	1	21.3	5.1	0.22	105	33391	6	3	3	1	7	5.1	0.1	0.45	Down
gi|46361162	pyridoxine biosynthesis protein PDX1	112	32992	12	3	9	2	38.5	6.76	0.23	107	32992	15	3	8	2	29.2	6.76	0.21	0.91	
gi|23504494	mature parasite-infected erythrocyte surface antigen	111	168186	20	3	17	2	12.8	4.76	0.09	220	168186	31	8	21	4	15	4.76	0.1	1.11	
gi|75016040	RecName: Full=Acidic leucine-rich nuclear phosphoprotein 32-related protein; AltName: Full=ANP32/acidic nuclear phosphoprotein-like protein	105	32986	8	4	4	3	13.5	4.27	0.36	72	32986	8	2	3	2	13.5	4.27	0.47	1.31	
gi|284055700	Chain A, Pyruvate kinase	102	56744	22	4	14	2	38.7	6.98	0.13	-	-	-	-	-	-	-	-	-	Down-Detect	Down
gi|23504898	lysine-rich membrane-associated PHISTb protein	100	61048	8	2	8	2	18	9.34	0.12	57	61048	14	2	11	2	27.5	9.34	0.11	0.92	
gi|23505219	profilin, putative	98	19005	5	4	3	2	17	4.22	0.41	35	19005	2	1	2	1	8.8	4.22	0.18	0.44	Down
gi|124803852	cysteine proteinase falcipain 3	97	56630	15	3	7	1	15.2	6.59	0.13	101	56630	19	4	4	1	9.1	6.59	0.12	0.92	
gi|322812543	Chain A, Glucose-6-phosphate isomerase	93	69517	23	5	10	2	23.5	6.9	0.1	144	69517	22	6	10	3	21.8	6.9	0.15	1.50	
gi|258597720	60S ribosomal protein L7-3, putative	91	32661	19	3	5	1	17.7	10.17	0.11	88	32661	18	3	4	1	16.3	10.17	0.1	0.91	
gi|23615603	nucleoside transporter 1	89	47600	10	4	4	1	12.6	8.36	0.07	98	47600	8	5	3	2	7.3	8.36	0.14	2.00	Up
gi|124802718	adenosine deaminase	89	42438	25	5	15	3	41.7	5.41	0.38	82	42438	15	2	9	1	28.1	5.41	0.16	0.42	Down
gi|124809152	mitochondrial acidic protein MAM33, putative	88	28854	14	2	4	2	18.3	4.89	0.42	104	28854	11	4	4	2	17.5	4.89	0.39	0.93	
gi|18076407	early transcribed membrane protein 14.1	86	11420	2	2	1	1	12.1	9.63	0.32	72	11420	4	2	2	1	20.6	9.63	0.3	0.94	
gi|225632259	U6 snRNA-associated Sm-like protein LSm3,putative	84	10694	4	3	2	1	35.2	5.16	0.35	108	10694	3	3	1	1	13.2	5.16	0.33	0.94	
gi|23504696	S-adenosyl-L-homocysteine hydrolase	81	53804	6	2	5	1	10.2	5.64	0.07	-	-	-	-	-	-	-	-	-	Down-Detect	Down
gi|124806612	conserved protein, unknown function	78	23644	10	2	5	1	21	9.64	0.15	40	23644	5	1	3	1	16.2	9.64	0.14	0.93	
gi|7672213	eukaryotic translation initiation factor 3 subunit K, putative	76	28017	8	1	5	1	31.1	5.61	0.27	58	28017	5	1	4	1	20	5.61	0.12	0.44	Down
gi|46361040	proteasome subunit alpha type-2, putative	76	26512	8	3	3	2	11.9	5.4	0.29	79	26512	6	3	3	2	11.9	5.4	0.43	1.48	
gi|124808201	40S ribosomal protein S8e, putative	76	25035	8	2	5	2	25.2	9.98	0.49	42	25035	7	1	4	1	19.7	9.98	0.29	0.59	
gi|23504603	inositol-3-phosphate synthase	75	69069	13	2	6	1	13.6	7.11	0.1	114	69069	13	5	7	3	14.9	7.11	0.15	1.50	
gi|23498728	Plasmodium exported protein, unknown function	74	27656	8	2	3	1	17.4	8.55	0.27	73	27656	6	4	2	1	10.7	8.55	0.26	0.96	
gi|23498182	V-type proton ATPase subunit B	74	55753	11	3	5	1	13.2	5.46	0.13	95	55753	10	3	4	1	12.6	5.46	0.12	0.92	
gi|124809402	60S ribosomal protein L1, putative	73	24790	4	2	2	1	11.5	9.85	0.14	74	24790	6	2	4	1	24	9.85	0.47	3.36	Up
gi|23615723	40S ribosomal protein S15	73	17240	9	1	7	1	49.7	10.38	0.21	121	17240	15	2	8	1	53	10.38	1.96	9.33	Up
gi|46576603	RecName: Full=Probable cathepsin C; Flags: Precursor	72	80361	22	3	9	1	17.9	5.82	0.14	107	80361	20	5	11	2	23.1	5.82	0.08	0.57	
gi|23615786	ubiquitin-60S ribosomal protein L40	71	14608	13	4	5	1	33.6	9.91	0.94	56	14608	17	2	6	1	47.7	9.91	0.23	0.24	Down
gi|124806534	serine hydroxymethyltransferase	69	49749	10	2	7	1	16.5	8.29	0.07	69	49749	8	3	6	2	14.5	8.29	0.14	2.00	Up
gi|651207688	Chain A, Serine Hydroxymethyltransferase	69	54007	19	2	10	1	21.7	7.21	0.07	69	54007	13	3	7	2	16	7.21	0.13	1.86	

gi|23504496	Plasmodium exported protein, unknown function	68	30696	4	3	2	1	8.1	9.47	0.24	61	30696	4	1	3	1	11.9	9.47	0.11	0.46	Down
gi|124801997	60S ribosomal protein L13, putative	67	23739	14	3	5	1	12.4	10.19	0.15	48	23739	14	2	4	1	22.3	10.19	0.14	0.93	
gi|23504938	alpha tubulin 1	65	50264	7	2	6	1	14.6	4.93	0.14	84	50264	7	2	5	1	14.6	4.93	0.07	0.50	Down
gi|23615408	phosphoribosylpyrophosphate synthetase	65	49352	11	1	8	1	26.1	9.39	0.07	63	49352	11	1	7	1	22	9.39	0.14	2.00	Up
gi|23505108	inosine-5~-monophosphate dehydrogenase	63	56115	10	1	7	1	25.3	7.99	0.13	40	56115	8	1	5	1	20.2	7.99	0.06	0.46	Down
gi|23615715	HVA22-like protein, putative	62	18410	4	2	2	1	11.8	9.34	0.2	80	18410	4	3	2	1	18.3	9.34	0.18	0.90	
gi|46361062	transketolase	62	75767	5	1	3	1	5.4	6.5	0.05	33	75767	10	1	8	1	12.5	6.5	0.09	1.80	
gi|8439487	hypothetical protein, partial	61	23455	3	2	2	1	14.6	5.87	0.15	34	23455	2	1	1	1	3.5	5.87	0.14	0.93	
gi|23498308	Plasmodium exported protein (PHISTb), unknown function	61	35939	9	2	7	2	28.8	8.75	0.21	84	35939	9	3	7	2	32	8.75	0.19	0.90	
gi|124804079	exported protein 1	60	17285	3	2	2	1	17.3	5.64	0.21	39	17285	3	1	1	1	11.1	5.64	0.2	0.95	
gi|23498140	Plasmodium exported protein (PHISTb), unknown function	60	60233	15	1	9	1	22.3	8.76	0.19	85	60233	19	2	8	2	17.7	8.76	0.17	0.89	
gi|124804238	40S ribosomal protein S18, putative	60	17880	7	3	4	2	37.8	10.46	0.44	112	17880	12	6	5	2	39.7	10.46	0.42	0.95	
gi|258596910	T-complex protein 1 subunit theta	60	60920	10	1	8	1	20.3	7.79	0.06	-	-	-	-	-	-	-	-	-	Down-Detect	Down
gi|23499154	60S ribosomal protein L13-2, putative	58	25425	4	1	3	1	21.9	10.78	0.14	-	-	-	-	-	-	-	-	-	Down-Detect	Down
gi|23504502	rhoptry-associated protein 2	57	46709	11	2	3	1	10.6	8.9	0.08	56	46709	15	2	8	1	23.1	8.9	0.07	0.88	
gi|258549116	serine esterase, putative	56	216941	48	1	16	1	11.5	8.82	0.02	39	216941	39	1	22	1	15.8	8.82	0.02		
gi|6851056	spermidine synthase	55	36573	7	2	4	1	19	6.97	0.1	64	36573	8	3	4	1	23.1	6.97	0.09	0.90	
gi|116668029	Chain A, Spermidine Synthase	55	32162	7	2	4	1	21.6	6.18	0.11	64	32162	10	3	4	1	26.1	6.18	0.1	0.91	
gi|124809582	M17 leucyl aminopeptidase	55	67778	24	1	6	1	16.2	8.78	0.05	71	67778	30	1	8	1	18	8.78	0.1	2.00	Up
gi|23499061	RNA-binding protein, putative	54	32421	2	2	1	1	6.3	9.11	0.11	40	32421	1	1	1	1	6.3	9.11	0.1	0.91	
gi|17148533	Ran-binding protein	54	33176	3	1	3	1	12.1	4.92	0.11	34	33176	1	1	1	1	4.3	4.92	0.1	0.91	
gi|23615363	conserved Plasmodium protein, unknown function	54	170566	65	0	15	0	14.2	8.86	0.04	49	170566	58	0	10	0	8.3	8.86	0.04		
gi|23504716	multidrug resistance protein 1	53	162150	35	2	16	2	15.9	8.94	0.04	24	162150	26	0	15	0	15.7	8.94	0.04		
gi|124804546	conserved Plasmodium protein, unknown function	52	28478	23	2	5	1	20.4	8.8	0.12	33	28478	13	0	1	0	4.9	8.8	0.12		
gi|124803615	casein kinase 2, alpha subunit	50	39865	6	2	4	1	15.8	8.9	0.09	37	39865	4	1	3	1	14	8.9	0.08	0.89	
gi|23498939	proteasome subunit alpha type-5, putative	49	28370	10	2	6	1	27.7	4.96	0.27	57	28370	9	1	2	1	13.3	4.96	0.12	0.44	Down
gi|1297293	60 kDa heat-shock protein PfHsp60	49	62125	19	1	15	1	32.6	7.05	0.06	-	-	-	-	-	-	-	-	-	Down-Detect	Down
gi|124802320	heat shock protein 60	49	62512	18	1	14	1	29	6.71	0.06	22	62512	14	0	10	0	24.5	6.71	0.05	0.83	
gi|225632011	heat shock protein 110, putative	49	108119	15	1	8	1	11.5	5.5	0.03	67	108119	25	2	12	2	13.9	5.5	0.06	2.00	Up
gi|124802223	hypoxanthine-guanine phosphoribosyltransferase	48	26346	9	0	4	0	19	7.59	0.14	59	26346	12	3	5	1	22.9	7.59	0.13	0.93	
gi|23498233	small GTP-binding protein sar1	47	22006	5	2	2	1	9.9	6.75	0.16	32	22006	6	1	3	1	13.5	6.75	0.15	0.94	
gi|124805752	glutathione peroxidase-like thioredoxin peroxidase	45	23937	5	1	3	1	19	8.99	0.15	90	23937	12	3	4	1	24.9	8.99	0.14	0.93	
gi|124806145	polyadenylate-binding protein, putative	44	97169	11	1	8	1	12.5	8.96	0.04	-	-	-	-	-	-	-	-	-	Down-Detect	Down
gi|23504672	WD repeat-containing protein 26, putative	43	150608	46	4	16	1	18.2	9.24	0.02	40	150608	41	3	12	1	11.1	9.24	0.02		
gi|23505090	nucleosome assembly protein	43	31807	5	2	4	1	17.1	4.17	0.11	131	31807	4	2	2	1	10.8	4.17	0.22	2.00	Up
gi|23615172	40S ribosomal protein S7, putative	43	22467	11	1	3	1	24.2	9.81	0.16	38	22467	12	1	3	1	21.6	9.81	0.15	0.94	
gi|124810024	conserved Plasmodium protein, unknown function	42	40043	3	1	2	1	7.9	9.6	0.09	-	-	-	-	-	-	-	-	-	Down-Detect	Down
gi|23615526	60S ribosomal protein L6-2, putative	42	25516	5	1	4	1	19.5	10.1	0.3	-	-	-	-	-	-	-	-	-	Down-Detect	Down
gi|23615562	casein kinase II beta chain	42	45257	4	1	4	1	10.4	3.66	0.08	25	45257	2	0	2	0	7.8	3.66	0.07	0.88	
gi|23615173	conserved Plasmodium protein, unknown function	42	95550	48	0	9	0	14.1	9.45	0.04	27	95550	47	0	8	0	13.3	9.45	0.03	0.75	
gi|124804998	rifin	42	38701	14	2	5	1	17.5	8.96	0.09	42	38701	11	1	3	1	14.5	8.96	0.09		
gi|124802073	single-strand telomeric DNA-binding protein GBP2, putative	41	29512	7	1	6	1	26.4	9.2	0.12	85	29512	6	2	4	1	18.7	9.2	0.11	0.92	
gi|23504955	high molecular weight rhoptry protein 3	41	104789	17	1	12	1	17.1	6.25	0.03	135	104789	17	3	11	1	16.2	6.25	0.06	2.00	Up
gi|23504857	Hsc70-interacting protein	41	51092	5	1	4	1	13.1	4.67	0.07	-	-	-	-	-	-	-	-	-	Down-Detect	Down
gi|225632239	conserved Plasmodium protein, unknown function	40	76808	32	2	11	1	23.1	5.34	0.09	69	76808	28	4	11	2	17.5	5.34	0.09		
gi|225632017	conserved Plasmodium protein, unknown function	39	696614	103	1	48	1	10.2	7.88	0	-	-	-	-	-	-	-	-	-	Down-Detect	Down
gi|225632182	conserved Plasmodium protein, unknown function	39	126994	18	2	9	1	8.6	5.28	0.03	31	126994	15	1	9	1	7.5	5.28	0.03		
gi|402550052	Chain A, Deoxyuridine 5~-triphosphate Nucleotidohydrolase, Putative	39	20626	2	1	2	1	14.4	6.59	0.17	52	20626	3	1	2	1	14.4	6.59	0.16	0.94	
gi|225631740	60S ribosomal protein L19	39	21566	5	2	2	1	4.9	11.32	0.17	-	-	-	-	-	-	-	-	-	Down-Detect	Down
gi|23498727	small exported membrane protein 1	39	14186	3	1	3	1	28.5	9.61	0.26	38	14186	4	1	2	1	13.8	9.61	0.24	0.92	
gi|74930131	RecName: Full=40S ribosomal protein SA	38	29837	13	2	5	1	25.5	5.91	0.12	33	29837	11	1	3	1	11.4	5.91	0.11	0.92	
gi|23498142	Plasmodium exported protein (PHISTa), unknown function	38	49713	7	1	5	1	15.2	9.51	0.07	100	49713	9	2	3	1	8.2	9.51	0.07		
gi|124802989	enhancer of rudimentary homolog, putative	38	12313	4	2	2	1	29.5	8.65	0.68	31	12313	2	1	1	1	10.5	8.65	0.28	0.41	Down
gi|225632238	conserved Plasmodium protein, unknown function	38	36234	6	1	2	1	6.7	4.11	0.1	68	36234	9	2	4	1	12.7	4.11	0.09	0.90	
gi|46361167	nascent polypeptide-associated complex subunit alpha, putative	38	20610	3	1	2	1	20.1	4.77	0.17	-	-	-	-	-	-	-	-	-	Down-Detect	Down

gi|124810210	40S ribosomal protein S3	37	24652	8	2	6	1	29.4	10.2	0.14	30	24652	2	1	2	1	12.7	10.2	0.14		
gi|13397937	putative Rab2 GTPase	37	24394	2	1	2	1	13.1	6.33	0.15	38	24394	2	1	2	1	8.9	6.33	0.14	0.93	
gi|74929507	RecName: Full=Actin-1; AltName: Full=Actin I	37	41844	8	1	6	1	22.6	5.21	0.08	26	41844	7	0	4	0	14.4	5.21	0.08		
gi|258597961	cysteine repeat modular protein 4	37	700473	105	1	47	1	10.7	8.43	0	32	700473	120	0	48	0	11.2	8.43	0		
gi|23615806	secreted ookinete protein, putative	37	135752	66	0	10	0	9.3	4.66	0.03	33	135752	74	0	10	0	8	4.66	0.02	0.67	
gi|23498915	conserved Plasmodium protein, unknown function	37	36570	8	1	4	1	12.2	8.79	0.1	31	36570	5	1	2	1	8.2	8.79	0.09	0.90	
gi|23498839	eukaryotic translation initiation factor 3 subunit I, putative	36	37261	7	1	6	1	19.6	6.43	0.09	-	-	-	-	-	-	-	-	-	Down-Detect	Down
gi|23505019	falstatin	35	46929	8	1	3	1	10.4	6.22	0.08	-	-	-	-	-	-	-	-	-	Down-Detect	Down
gi|23498992	surface-associated interspersed protein 8.2 (SURFIN 8.2)	35	248324	43	1	29	1	17.7	5.35	0.01	-	-	-	-	-	-	-	-	-	Down-Detect	Down
gi|225632293	Plasmodium exported protein, unknown function	35	36390	6	1	5	1	15.2	5.74	0.1	-	-	-	-	-	-	-	-	-	Down-Detect	Down
gi|23504862	eukaryotic translation initiation factor 3 subunit E, putative	35	61379	22	1	9	1	21.1	7.08	0.12	41	61379	18	1	9	1	22.4	7.08	0.05	0.42	Down
gi|124802973	ADP/ATP transporter on adenylate translocase	35	33705	7	1	4	1	18.9	9.68	0.1	61	33705	5	2	3	1	13	9.68	0.1		
gi|225631665	conserved Plasmodium protein, unknown function	35	469585	55	1	37	1	11.8	7.47	0.01	-	-	-	-	-	-	-	-	-	Down-Detect	Down
gi|23615772	conserved Plasmodium protein, unknown function	35	386087	47	1	27	1	10.4	8.76	0.01	-	-	-	-	-	-	-	-	-	Down-Detect	Down
gi|258597310	60S ribosomal protein L35, putative	34	14739	1	1	1	1	5.6	10.79	0.25	40	14739	2	1	1	1	5.6	10.79	0.23	0.92	
gi|4494003	40S ribosomal protein S3A, putative	33	30028	9	1	9	1	33.2	9.8	0.12	-	-	-	-	-	-	-	-	-	Down-Detect	Down
gi|124805983	clathrin heavy chain, putative	33	232803	40	1	16	1	9.5	6	0.01	-	-	-	-	-	-	-	-	-	Down-Detect	Down
gi|23615267	conserved Plasmodium protein, unknown function	32	34955	19	1	5	1	15.4	9.22	0.1	31	34955	19	1	6	1	19.8	9.22	0.1		
gi|23615340	conserved Plasmodium protein, unknown function	32	88519	20	2	9	1	14.6	9.07	0.04	-	-	-	-	-	-	-	-	-	Down-Detect	Down
gi|258596983	conserved Plasmodium protein, unknown function	32	45596	10	1	6	1	23	7.12	0.08	-	-	-	-	-	-	-	-	-	Down-Detect	Down
gi|124803451	60S acidic ribosomal protein P1, putative	32	13006	3	0	1	0	13.6	4.57	0.28	26	13006	8	0	3	0	35.6	4.57	0.27	0.96	
gi|23504660	FACT complex subunit SPT16, putative	31	132600	9	1	9	1	9.1	4.86	0.03	-	-	-	-	-	-	-	-	-	Down-Detect	Down
gi|23504543	small ubiquitin-related modifier	31	11053	3	1	3	1	38	4.74	0.33	-	-	-	-	-	-	-	-	-	Down-Detect	Down
gi|23498881	conserved Plasmodium protein, unknown function	31	29231	1	1	1	1	7.4	8.76	0.12	33	29231	2	1	2	1	9	8.76	0.11	0.92	
gi|75016029	RecName: Full=STI1-like protein	30	66015	7	1	6	1	10.3	6.63	0.05	44	66015	10	2	6	1	10.3	6.63	0.05		
gi|46361188	hexokinase	30	55226	26	0	9	0	31.2	6.72	0.13	-	-	-	-	-	-	-	-	-	Down-Detect	Down
gi|23504582	asparagine--tRNA ligase	30	85195	22	3	8	1	13.3	9.01	0.04	32	85195	27	3	8	1	12.2	9.01	0.04		
gi|23498950	zinc finger, C3HC4 type, putative	29	253816	45	1	17	1	8.6	8.32	0.01	-	-	-	-	-	-	-	-	-	Down-Detect	Down
gi|23504648	40S ribosomal protein S11	29	16067	15	0	7	0	51.7	10.49	0.5	142	16067	15	4	7	3	43	10.49	1.16	2.32	Up
gi|225631797	AP-4 complex subunit epsilon, putative	29	161304	33	1	17	1	14.5	5.44	0.02	-	-	-	-	-	-	-	-	-	Down-Detect	Down
gi|258597176	erythrocyte membrane protein 1, PfEMP1	29	248280	73	1	23	1	13.3	5.47	0.01	30	248280	79	4	26	1	12.3	5.47	0.01		
gi|23504621	deoxyribodipyrimidine photo-lyase, putative	29	129117	22	1	8	1	8.1	9.22	0.03	30	129117	26	4	11	1	9.2	9.22	0.03		
gi|23615206	conserved Plasmodium protein, unknown function	29	56775	16	1	4	1	10.3	9.53	0.06	30	56775	18	4	6	1	11.9	9.53	0.06		
gi|46361276	erythrocyte membrane protein 1, PfEMP1	29	254458	105	1	33	1	18.5	5.66	0.01	-	-	-	-	-	-	-	-	-	Down-Detect	Down
gi|124806687	erythrocyte membrane protein 1, PfEMP1	29	300099	34	1	25	1	12.4	5.43	0.01	-	-	-	-	-	-	-	-	-	Down-Detect	Down
gi|46362284	conserved Plasmodium protein, unknown function	29	25357	12	0	3	0	10	9.82	0.14	-	-	-	-	-	-	-	-	-	Down-Detect	Down
gi|124802670	60S ribosomal protein L3	28	44193	10	0	7	0	23.8	10.21	0.08	-	-	-	-	-	-	-	-	-	Down-Detect	Down
gi|152968490	cyclic nucleotide-specific phosphodiesterase	28	97106	8	1	6	1	9.6	6.54	0.04	31	97106	6	1	6	1	8.6	6.54	0.03	0.75	
gi|23505053	conserved Plasmodium protein, unknown function	28	85353	9	0	6	0	11.2	9.1	0.04	32	85353	9	0	7	0	11.7	9.1	0.04		
gi|124800762	conserved Plasmodium protein, unknown function	28	83770	12	0	10	0	14.8	8.54	0.04	-	-	-	-	-	-	-	-	-	Down-Detect	Down
gi|23499287	hypothetical protein, partial	28	82064	10	0	7	0	12.6	9.28	0.04	-	-	-	-	-	-	-	-	-	Down-Detect	Down
gi|23615441	conserved Plasmodium protein, unknown function	28	205350	50	0	22	0	12.9	9.22	0.02	22	205350	51	0	16	0	8.2	9.22	0.02		
gi|46362258	conserved Plasmodium protein, unknown function	27	153622	26	0	21	0	17.5	8.47	0.02	-	-	-	-	-	-	-	-	-	Down-Detect	Down
gi|124801366	ATP synthase F1, alpha subunit	27	61731	13	0	4	0	12	8.72	0.06	-	-	-	-	-	-	-	-	-	Down-Detect	Down
gi|23505253	ring-exported protein 1	27	82991	8	0	5	0	7.4	5.46	0.04	-	-	-	-	-	-	-	-	-	Down-Detect	Down
gi|23498873	ferrodoxin reductase-like protein	26	72675	23	1	8	1	15.3	8.91	0.05	-	-	-	-	-	-	-	-	-	Down-Detect	Down
gi|225631857	conserved Plasmodium protein, unknown function	26	138544	23	0	9	0	10.1	8.44	0.03	29	138544	23	0	9	0	11.9	8.44	0.02	0.67	
gi|75009813	RecName: Full=Plasmepsin-1; AltName: Full=Aspartic hemoglobinase I; AltName: Full=PfAPG; Flags: Precursor	26	51428	3	1	2	1	8.8	6.72	0.07	20	51428	4	0	3	0	12.8	6.72	0.06	0.86	
gi|23504536	trafficking protein particle complex subunit 8,putative	26	328469	56	0	26	0	11	7.22	0.01	29	328469	57	0	26	0	11.5	7.22	0.01		
gi|23615433	conserved Plasmodium protein, unknown function	25	319935	65	0	32	0	14.1	7.32	0.01	-	-	-	-	-	-	-	-	-	Down-Detect	Down
gi|23615272	conserved Plasmodium protein, unknown function	25	277526	36	0	19	0	9	7.79	0.01	-	-	-	-	-	-	-	-	-	Down-Detect	Down
gi|258596969	conserved Plasmodium protein, unknown function	25	14508	3	0	2	0	15	6.09	0.25	22	14508	4	0	2	0	15	6.09	0.24	0.96	
gi|74862993	RecName: Full=Uncharacterized protein PFB0765w	25	166903	88	0	19	0	16.5	6.19	0.04	-	-	-	-	-	-	-	-	-	Down-Detect	Down
gi|15375389	60S ribosomal protein L7, putative	25	30504	6	0	4	0	16	10.35	0.12	-	-	-	-	-	-	-	-	-	Down-Detect	Down
gi|124810495	conserved protein, unknown function	25	115321	19	0	12	0	14.9	8.18	0.03	-	-	-	-	-	-	-	-	-	Down-Detect	Down
gi|124803955	heat shock protein 90, putative	25	108397	5	0	4	0	5.9	6.22	0.03	34	108397	5	1	5	1	9.1	6.22	0.03		

gi|23505054	6-phosphofructokinase	25	159351	25	0	17	0	19.2	6.32	0.02	-	-	-	-	-	-	-	-	-	Down-Detect	Down
gi|258596939	DnaJ protein, putative	25	117215	18	0	10	0	10.7	8.77	0.03	-	-	-	-	-	-	-	-	-	Down-Detect	Down
gi|124809222	cGMP-dependent protein kinase	24	97632	14	0	11	0	15.4	5.4	0.04	-	-	-	-	-	-	-	-	-	Down-Detect	Down
gi|7799189	thioredoxin	24	11709	15	0	2	0	14.4	4.67	0.31	54	11709	19	2	4	1	48.1	4.67	0.3	0.97	
gi|74862728	RecName: Full=Pentatricopeptide repeat-containing protein PFL1605w	24	169454	26	0	11	0	10.1	7.95	0.02	-	-	-	-	-	-	-	-	-	Down-Detect	Down
gi|124810493	conserved Plasmodium protein, unknown function	24	185792	25	1	15	1	15.5	9.12	0.02	-	-	-	-	-	-	-	-	-	Down-Detect	Down
gi|258597955	conserved Plasmodium membrane protein, unknown function	24	577659	68	1	36	1	8.8	8.86	0.01	36	577659	77	3	45	1	11.7	8.86	0.01		
gi|23615467	60S ribosomal protein L23, putative	23	14983	5	0	4	0	33.8	9.9	0.24	-	-	-	-	-	-	-	-	-	Down-Detect	Down
gi|23498211	conserved protein, unknown function	23	27337	6	0	5	0	19.1	6.78	0.13	-	-	-	-	-	-	-	-	-	Down-Detect	Down
gi|124809822	6-phosphogluconate dehydrogenase,decarboxylating, putative	23	52960	14	0	7	0	19	6.58	0.14	38	52960	11	1	6	1	12.4	6.58	0.13	0.93	
gi|46361227	protein DJ-1	23	20280	5	0	5	0	33.9	6.95	0.18	-	-	-	-	-	-	-	-	-	Down-Detect	Down
gi|23615559	aconitate hydratase	23	103313	14	0	9	0	10.3	7.73	0.03	66	103313	13	2	8	1	11.3	7.73	0.03		
gi|23498752	conserved Plasmodium protein, unknown function	23	118270	5	0	4	0	5.6	8.1	0.03	-	-	-	-	-	-	-	-	-	Down-Detect	Down
gi|258597760	conserved Plasmodium membrane protein, unknown function	23	682022	106	0	52	0	10.8	8.86	0.01	24	682022	112	0	52	0	11.6	8.86	0		
gi|23504632	conserved Plasmodium protein, unknown function	23	190407	21	0	10	0	7.2	9.71	0.02	-	-	-	-	-	-	-	-	-	Down-Detect	Down
gi|124801947	RNA-binding protein, putative	22	30033	12	0	7	0	26.6	10.07	0.12	-	-	-	-	-	-	-	-	-	Down-Detect	Down
gi|225631936	MORN repeat protein, putative	21	519893	80	0	36	0	8.9	9.15	0.01	-	-	-	-	-	-	-	-	-	Down-Detect	Down
gi|23504575	conserved Plasmodium protein, unknown function	21	369953	38	0	23	0	9	5.29	0.01	-	-	-	-	-	-	-	-	-	Down-Detect	Down
gi|3649757	conserved Plasmodium protein, unknown function	21	202018	65	0	19	0	16.5	8.3	0.02	-	-	-	-	-	-	-	-	-	Down-Detect	Down
gi|124808756	conserved Plasmodium protein, unknown function	21	192484	86	0	17	0	12.4	9.67	0.02	-	-	-	-	-	-	-	-	-	Down-Detect	Down
gi|124805631	conserved Plasmodium protein, unknown function	21	208541	28	0	17	0	12.6	8.3	0.02	-	-	-	-	-	-	-	-	-	Down-Detect	Down
gi|124804234	autophagy-related protein 7, putative	21	156530	34	0	14	0	12.5	6.05	0.02	-	-	-	-	-	-	-	-	-	Down-Detect	Down
gi|124809084	conserved Plasmodium protein, unknown function	21	152167	24	0	13	0	12.9	8.42	0.02	-	-	-	-	-	-	-	-	-	Down-Detect	Down
gi|23615179	sodium/hydrogen exchanger, Na+, H+ antiporter	21	225940	33	0	18	0	10.8	8.68	0.02	-	-	-	-	-	-	-	-	-	Down-Detect	Down
gi|124802600	conserved Plasmodium protein, unknown function	21	190279	15	0	8	0	5.8	8.29	0.02	-	-	-	-	-	-	-	-	-	Down-Detect	Down
gi|124806636	conserved Plasmodium protein, unknown function	21	212627	28	0	17	0	12.8	4.96	0.02	-	-	-	-	-	-	-	-	-	Down-Detect	Down
gi|124804384	conserved Plasmodium protein, unknown function	21	118257	20	0	9	0	12.6	8.84	0.03	-	-	-	-	-	-	-	-	-	Down-Detect	Down
gi|124804432	conserved Plasmodium protein, unknown function	21	133324	27	0	12	0	13.8	5.18	0.03	-	-	-	-	-	-	-	-	-	Down-Detect	Down
gi|225632254	rRNA-processing protein FCF1, putative	21	23145	9	0	3	0	15.7	9.63	0.15	-	-	-	-	-	-	-	-	-	Down-Detect	Down
gi|23504673	mRNA-binding protein PUF1	21	223948	41	0	18	0	12.6	8.2	0.02	-	-	-	-	-	-	-	-	-	Down-Detect	Down
gi|23615263	M1-family alanyl aminopeptidase	21	125983	10	0	9	0	10.7	7.3	0.03	24	125983	13	0	10	0	11.2	7.3	0.03		
gi|225631658	conserved Plasmodium protein, unknown function	21	46993	9	0	6	0	20.5	8.92	0.08	-	-	-	-	-	-	-	-	-	Down-Detect	Down
gi|23615579	aspartate carbamoyltransferase	21	43224	8	0	5	0	17.1	8.55	0.08	-	-	-	-	-	-	-	-	-	Down-Detect	Down
gi|3649758	T-complex protein 1 subunit eta	21	59540	20	0	6	0	17.4	5.45	0.06	18	59540	22	0	8	0	23	5.45	0.06		
gi|258597535	60 kDa chaperonin	21	81434	15	0	10	0	21	4.97	0.04	28	81434	18	1	12	1	22.8	4.97	0.08	2.00	Up
gi|74862955	RecName: Full=Origin recognition complex subunit 1; Short=PfORC1	21	138653	7	0	5	0	5.8	9.52	0.03	22	138653	10	0	10	0	12.8	9.52	0.02	0.67	
gi|23504953	mitochondrial carrier protein, putative	20	141981	56	0	22	0	19.8	9.63	0.02	21	141981	52	0	15	0	13.7	9.63	0.02		
gi|23498891	rhoptry-associated leucine zipper-like protein 1	20	87825	27	0	9	0	13	5.31	0.04	-	-	-	-	-	-	-	-	-	Down-Detect	Down
gi|23498854	mitochondrial ribosomal protein S8 precursor,putative	20	14793	3	0	1	0	13.2	9.98	0.25	-	-	-	-	-	-	-	-	-	Down-Detect	Down
gi|23505129	3-oxoacyl-[acyl-carrier-protein] reductase	20	33326	6	0	4	0	15	9.33	0.11	-	-	-	-	-	-	-	-	-	Down-Detect	Down
gi|124809066	conserved Plasmodium protein, unknown function	20	143906	16	0	12	0	11.2	8.94	0.02	-	-	-	-	-	-	-	-	-	Down-Detect	Down
gi|225631940	conserved Plasmodium protein, unknown function	20	737235	114	0	55	0	11.3	9.13	0	-	-	-	-	-	-	-	-	-	Down-Detect	Down
gi|23615541	ABC transporter B family member 5, putative	20	108443	23	0	10	0	11.2	8.47	0.03	-	-	-	-	-	-	-	-	-	Down-Detect	Down
gi|225632201	inositol 5-phosphatase, putative	20	172950	48	0	18	0	16.4	9.68	0.02	-	-	-	-	-	-	-	-	-	Down-Detect	Down
gi|23615413	conserved Plasmodium protein, unknown function	20	185578	24	0	17	0	12.3	7.09	0.02	-	-	-	-	-	-	-	-	-	Down-Detect	Down
gi|23499167	RNA-binding protein (U1 snRNP-like), putative	20	27861	5	0	5	0	28.9	8.34	0.13	-	-	-	-	-	-	-	-	-	Down-Detect	Down
gi|124805668	porphobilinogen deaminase	20	50315	95	0	7	0	24.4	9.54	0.07	-	-	-	-	-	-	-	-	-	Down-Detect	Down
gi|46361176	leucine--tRNA ligase, putative	20	170133	11	0	9	0	8.5	6.12	0.02	14	170133	16	0	8	0	6.6	6.12	0.02		
gi|124802394	conserved Plasmodium protein, unknown function	20	137321	16	0	10	0	11.2	9.36	0.03	-	-	-	-	-	-	-	-	-	Down-Detect	Down
gi|124802282	transcriptional coactivator ADA2	20	300067	43	0	22	0	9.9	8.79	0.01	-	-	-	-	-	-	-	-	-	Down-Detect	Down
gi|124805693	conserved Plasmodium protein, unknown function	19	24532	3	0	2	0	11.5	5.4	0.15	-	-	-	-	-	-	-	-	-	Down-Detect	Down

gi|124801201	conserved Plasmodium membrane protein, unknown function	19	493899	58	0	29	0	9.1	8.46	0.01	-	-	-	-	-	-	-	-	-	Down-Detect	Down
gi|23498892	ubiquitin carboxyl-terminal hydrolase, putative	19	145399	26	0	12	0	13.5	8.9	0.02	-	-	-	-	-	-	-	-	-	Down-Detect	Down
gi|1575675	rab6	19	27914	2	0	2	0	7.9	7.63	0.13	31	27914	4	1	3	1	10.8	7.63	0.12	0.92	
gi|225632022	phosphatidylinositol transfer protein, putative	19	220966	38	0	16	0	8.3	5.88	0.02	-	-	-	-	-	-	-	-	-	Down-Detect	Down
gi|124806350	subpellicular microtubule protein 2, putative	19	30171	3	0	1	0	5	9.31	0.12	18	30171	4	0	2	0	11.6	9.31	0.11	0.92	
gi|23504954	dynein heavy chain, putative	18	720134	98	0	48	0	10.6	6.18	0	-	-	-	-	-	-	-	-	-	Down-Detect	Down
gi|124808290	conserved Plasmodium protein, unknown function	18	39806	4	0	4	0	16.2	8.57	0.09	-	-	-	-	-	-	-	-	-	Down-Detect	Down
gi|23510631	asparagine-rich antigen Pfa35-2	18	363848	48	0	24	0	9.5	6.47	0.01	25	363848	53	0	28	0	11.7	6.47	0.01		
gi|124804142	leucine-rich repeat protein	18	93275	24	0	10	0	13.6	8.97	0.04	-	-	-	-	-	-	-	-	-	Down-Detect	Down
gi|225631849	conserved Plasmodium protein, unknown function	18	102515	21	0	10	0	11.7	8.83	0.03	-	-	-	-	-	-	-	-	-	Down-Detect	Down
gi|225632037	conserved Plasmodium protein, unknown function	18	439635	32	0	22	0	7.3	7.81	0.01	-	-	-	-	-	-	-	-	-	Down-Detect	Down
gi|124804153	conserved Plasmodium protein, unknown function	18	153603	34	0	10	0	9.6	6.77	0.02	17	153603	40	0	15	0	13.5	6.77	0.02		
gi|124803042	rifin	18	40212	8	0	6	0	25.3	8.67	0.09	-	-	-	-	-	-	-	-	-	Down-Detect	Down
gi|258597363	3-oxo-5-alpha-steroid 4-dehydrogenase, putative	18	35445	3	0	2	0	5.4	9.53	0.1	-	-	-	-	-	-	-	-	-	Down-Detect	Down
gi|23504877	conserved Plasmodium protein, unknown function	17	225390	25	0	12	0	9.2	6.01	0.02	21	225390	45	0	22	0	14.4	6.01	0.01	0.50	Down
gi|225631798	conserved protein, unknown function	17	296598	30	0	16	0	10	9.02	0.01	-	-	-	-	-	-	-	-	-	Down-Detect	Down
gi|23615204	conserved Plasmodium protein, unknown function	17	160471	15	0	10	0	8.8	9.3	0.02	-	-	-	-	-	-	-	-	-	Down-Detect	Down
gi|258597491	triose or hexose phosphate/phosphate translocator, putative	17	54687	4	0	3	0	13.2	9.13	0.06	-	-	-	-	-	-	-	-	-	Down-Detect	Down
gi|124804419	conserved Plasmodium protein, unknown function	17	330099	28	0	19	0	8.6	6.16	0.02	21	330099	27	0	20	0	8.4	6.16	0.01	0.50	Down
gi|225631826	conserved Plasmodium protein, unknown function	17	205540	23	0	17	0	11.3	8.72	0.02	-	-	-	-	-	-	-	-	-	Down-Detect	Down
gi|23615660	Plasmodium exported protein, unknown function	17	32763	5	0	2	0	11.9	9.23	0.11	19	32763	7	0	6	0	27	9.23	0.1	0.91	
gi|7670012	rifin	16	39495	14	0	3	0	6.1	9.15	0.09	16	39495	14	0	5	0	22.7	9.15	0.08	0.89	
gi|124808373	conserved Plasmodium protein, unknown function	16	390446	46	0	28	0	10.6	9.23	0.01	-	-	-	-	-	-	-	-	-	Down-Detect	Down
gi|225631966	conserved Plasmodium protein, unknown function	16	404851	73	0	33	0	11.5	6.56	0.01	-	-	-	-	-	-	-	-	-	Down-Detect	Down
gi|124804710	conserved Plasmodium protein, unknown function	16	219717	14	0	13	0	8.6	9.63	0.02	-	-	-	-	-	-	-	-	-	Down-Detect	Down
gi|124804373	60S ribosomal protein L38	16	10307	1	0	1	0	8	10.71	0.36	38	10307	8	1	3	1	20.7	10.71	0.79	2.19	Up
gi|124802905	merozoite surface protein 6	16	42250	18	0	3	0	10.8	4.32	0.08	18	42250	21	0	4	0	13.2	4.32	0.08		
gi|124806291	RNA pseudouridylate synthase, putative	16	48335	10	0	6	0	15.9	9.57	0.07	-	-	-	-	-	-	-	-	-	Down-Detect	Down
gi|124804435	T-complex protein 1 subunit alpha	15	60223	4	0	2	0	2.6	6.65	0.06	16	60223	6	0	2	0	3.3	6.65	0.05	0.83	
gi|23498776	Cg7 protein	15	153379	25	0	14	0	13.9	6.98	0.02	-	-	-	-	-	-	-	-	-	Down-Detect	Down
gi|124808195	DNA repair helicase, putative	15	135460	49	0	20	0	22.2	8.96	0.03	14	135460	37	0	13	0	10.4	8.96	0.02	0.67	
gi|46361233	HECT-domain (ubiquitin-transferase), putative	15	1205255	194	0	99	0	12	8.19	0	16	1205255	190	0	106	0	12.5	8.19	0		
gi|3758839	P-type ATPase, putative	15	217882	19	0	14	0	9.7	8.09	0.02	16	217882	21	0	13	0	7	8.09	0.02		
gi|124801012	serine repeat antigen 7	15	109569	13	0	9	0	11.5	5.57	0.03	16	109569	17	0	10	0	12.6	5.57	0.03		
gi|124807008	osmiophilic body protein	14	377151	62	0	24	0	8.5	5.71	0.01	-	-	-	-	-	-	-	-	-	Down-Detect	Down
gi|303324901	Chain A, Heat Shock Protein 86	-	-	-	-	-	-	-	-	-	447	25195	23	15	7	2	28.3	4.85	0.46	Up-Detect	Up
gi|124803848	cysteine proteinase falcipain 2b	-	-	-	-	-	-	-	-	-	186	55768	21	8	9	5	21.6	8.14	0.5	Up-Detect	Up
gi|23504618	purine nucleoside phosphorylase	-	-	-	-	-	-	-	-	-	134	26841	13	5	6	3	22.4	6.07	0.6	Up-Detect	Up
gi|124810483	proteasome subunit alpha type-1, putative	-	-	-	-	-	-	-	-	-	127	28819	5	2	3	1	14.2	5.51	0.12	Up-Detect	Up
gi|124805478	eukaryotic translation initiation factor 5A	-	-	-	-	-	-	-	-	-	74	17620	5	2	2	1	24.8	5.42	0.19	Up-Detect	Up
gi|23504725	karyopherin beta	-	-	-	-	-	-	-	-	-	73	127272	7	1	6	1	7.1	4.8	0.03	Up-Detect	Up
gi|23499027	receptor for activated c kinase	-	-	-	-	-	-	-	-	-	62	35664	3	2	3	2	15.2	6.24	0.2	Up-Detect	Up
gi|23505091	DnaJ protein, putative	-	-	-	-	-	-	-	-	-	59	43233	11	2	8	1	28.1	8.81	0.16	Up-Detect	Up
gi|258596875	26S proteasome regulatory subunit RPN1,putative	-	-	-	-	-	-	-	-	-	59	108289	44	2	14	1	21	5.95	0.03	Up-Detect	Up
gi|23504687	40S ribosomal protein S9, putative	-	-	-	-	-	-	-	-	-	57	22109	7	2	3	2	23.3	10.45	0.33	Up-Detect	Up
gi|23499155	40S ribosomal protein S16, putative	-	-	-	-	-	-	-	-	-	50	16275	5	2	2	1	12.5	10.25	0.21	Up-Detect	Up
gi|23505032	elongation factor 1-beta	-	-	-	-	-	-	-	-	-	50	32007	3	1	3	1	17	4.94	0.1	Up-Detect	Up
gi|124801080	ATP-dependent RNA helicase UAP56	-	-	-	-	-	-	-	-	-	49	52191	7	1	5	1	12	5.68	0.06	Up-Detect	Up
gi|225632223	coatomer subunit epsilon, putative	-	-	-	-	-	-	-	-	-	44	33071	3	1	3	1	14.7	4.59	0.1	Up-Detect	Up
gi|124806302	WD repeat-containing protein, putative	-	-	-	-	-	-	-	-	-	41	378674	49	2	24	1	10.2	9.02	0.01	Up-Detect	Up
gi|124809308	thioredoxin peroxidase 1	-	-	-	-	-	-	-	-	-	40	21793	11	1	4	1	22.1	6.65	0.16	Up-Detect	Up
gi|124803892	folate transporter 2	-	-	-	-	-	-	-	-	-	40	51321	4	1	3	1	6.6	8.63	0.06	Up-Detect	Up
gi|225631696	conserved Plasmodium protein, unknown function	-	-	-	-	-	-	-	-	-	39	1116481	113	1	73	1	9.3	9.37	0	Up-Detect	Up
gi|23615654	thioredoxin-related protein, putative	-	-	-	-	-	-	-	-	-	38	23972	2	1	2	1	10.1	9.44	0.3	Up-Detect	Up
gi|8052274	elongation factor 1 (EF-1), putative	-	-	-	-	-	-	-	-	-	38	17695	3	0	2	0	12.8	4.5	0.19	Up-Detect	Up

gi|23498906	metallo-hydrolase/oxidoreductase, putative	-	-	-	-	-	-	-	-	-	38	119584	16	2	13	1	16.9	7.01	0.03	Up-Detect	Up
gi|258597872	signal peptide peptidase	-	-	-	-	-	-	-	-	-	37	47547	4	1	4	1	7	8.95	0.07	Up-Detect	Up
gi|23504974	ATP-dependent protease ATPase subunit ClpY	-	-	-	-	-	-	-	-	-	36	106396	35	3	10	1	11.3	8.42	0.03	Up-Detect	Up
gi|23499022	conserved Plasmodium protein, unknown function	-	-	-	-	-	-	-	-	-	35	100876	8	1	6	1	7.7	5.92	0.03	Up-Detect	Up
gi|124803903	heat shock protein 101	-	-	-	-	-	-	-	-	-	35	102810	22	1	15	1	21.2	9.17	0.03	Up-Detect	Up
gi|408535927	Chain A, Apicoplast Tic22, Putative	-	-	-	-	-	-	-	-	-	34	33232	53	1	26	1	38.4	9.24	0.1	Up-Detect	Up
gi|46361219	conserved protein, unknown function	-	-	-	-	-	-	-	-	-	33	43622	3	1	3	1	11.3	7.19	0.08	Up-Detect	Up
gi|46361134	60S ribosomal protein L27a, putative	-	-	-	-	-	-	-	-	-	33	16712	3	1	2	1	15.5	10.54	0.2	Up-Detect	Up
gi|3694805	cytoadherence linked asexual protein, partial	-	-	-	-	-	-	-	-	-	33	160994	33	1	14	1	15.8	8.98	0.02	Up-Detect	Up
gi|23505252	cytoadherence linked asexual protein 9	-	-	-	-	-	-	-	-	-	33	160313	20	1	14	1	14.2	8.88	0.02	Up-Detect	Up
gi|124802168	eukaryotic translation initiation factor 2 subunit beta, putative	-	-	-	-	-	-	-	-	-	33	25306	11	1	8	1	43.2	9.23	0.13	Up-Detect	Up
gi|124806724	GAS8-like protein, putative	-	-	-	-	-	-	-	-	-	33	54772	4	1	2	1	3.8	7.98	0.06	Up-Detect	Up
gi|124800772	hexose transporter	-	-	-	-	-	-	-	-	-	33	56380	5	1	3	1	12.7	8.8	0.06	Up-Detect	Up
gi|23505020	conserved Plasmodium protein, unknown function	-	-	-	-	-	-	-	-	-	32	188566	43	1	20	1	14.4	9.15	0.02	Up-Detect	Up
gi|124802200	PRE-binding protein	-	-	-	-	-	-	-	-	-	30	131545	18	1	13	1	14.3	9.19	0.03	Up-Detect	Up
gi|124801337	vacuolar protein sorting-associated protein 45,putative	-	-	-	-	-	-	-	-	-	30	86202	27	1	5	1	5.7	6.51	0.04	Up-Detect	Up
gi|124801981	40S ribosomal protein S20e, putative	-	-	-	-	-	-	-	-	-	30	13504	1	1	1	1	11	9.67	0.25	Up-Detect	Up
gi|23498743	tRNA m5C-methyltransferase, putative	-	-	-	-	-	-	-	-	-	30	141140	19	2	12	1	12.5	6.35	0.02	Up-Detect	Up
gi|23499165	conserved Plasmodium protein, unknown function	-	-	-	-	-	-	-	-	-	29	70662	3	1	2	1	3.3	9.11	0.05	Up-Detect	Up
gi|23504681	40S ribosomal protein S24	-	-	-	-	-	-	-	-	-	29	15382	2	1	2	1	14.3	10.75	0.22	Up-Detect	Up
gi|258549210	conserved Plasmodium protein, unknown function	-	-	-	-	-	-	-	-	-	29	30341	6	0	4	0	18.6	7.64	0.11	Up-Detect	Up
gi|124808563	H/ACA ribonucleoprotein complex subunit 4,putative	-	-	-	-	-	-	-	-	-	27	52908	19	0	5	0	13.9	9.06	0.06	Up-Detect	Up
gi|46362290	DNA repair protein RAD50, putative	-	-	-	-	-	-	-	-	-	27	267786	76	0	33	0	16.6	8.78	0.01	Up-Detect	Up
gi|23499218	ras-related protein Rab-18	-	-	-	-	-	-	-	-	-	27	23148	3	0	2	0	15.4	8.11	0.15	Up-Detect	Up

gi|6562716	conserved Plasmodium protein, unknown function	-	-	-	-	-	-	-	-	-	27	229981	21	0	11	0	5.4	6.44	0.01	Up-Detect	Up
gi|225632241	ATPase, putative	-	-	-	-	-	-	-	-	-	27	139581	72	0	12	0	9.8	8.66	0.02	Up-Detect	Up
gi|258597683	conserved Plasmodium protein, unknown function	-	-	-	-	-	-	-	-	-	27	77169	40	0	12	0	22.6	8.98	0.04	Up-Detect	Up
gi|23615185	DNA ligase I	-	-	-	-	-	-	-	-	-	27	104440	11	0	7	0	10.3	7.66	0.03	Up-Detect	Up
gi|23615667	proteasome subunit alpha type-4, putative	-	-	-	-	-	-	-	-	-	26	27930	3	0	2	0	8.1	5.85	0.12	Up-Detect	Up
gi|124809637	conserved Plasmodium protein, unknown function	-	-	-	-	-	-	-	-	-	26	442796	51	0	35	0	10.6	8.64	0.01	Up-Detect	Up
gi|46361220	pyruvate kinase	-	-	-	-	-	-	-	-	-	26	55625	14	0	10	0	27	7.5	0.06	Up-Detect	Up
gi|23615256	meiosis-specific nuclear structural protein 1,putative	-	-	-	-	-	-	-	-	-	26	61194	11	0	6	0	8.9	8.91	0.05	Up-Detect	Up
gi|3649767	SECIS-binding protein 2, putative	-	-	-	-	-	-	-	-	-	26	71419	3	0	2	0	2.1	9.94	0.05	Up-Detect	Up
gi|258597895	conserved Plasmodium membrane protein, unknown function	-	-	-	-	-	-	-	-	-	25	388575	96	0	29	0	9.4	8.84	0.01	Up-Detect	Up
gi|225632177	conserved Plasmodium protein, unknown function	-	-	-	-	-	-	-	-	-	25	420410	90	0	31	0	9.5	8.7	0.01	Up-Detect	Up
gi|296005130	Pfmc-2TM Maurer~s cleft two transmembrane protein	-	-	-	-	-	-	-	-	-	25	27380	14	0	8	0	31.2	9.4	0.12	Up-Detect	Up
gi|258597178	Pfmc-2TM Maurer~s cleft two transmembrane protein	-	-	-	-	-	-	-	-	-	25	27162	12	0	6	0	22.5	9.36	0.13	Up-Detect	Up
gi|23498719	Pfmc-2TM Maurer~s cleft two transmembrane protein	-	-	-	-	-	-	-	-	-	25	27539	10	0	5	0	21.3	9.59	0.13	Up-Detect	Up
gi|46361088	conserved Plasmodium protein, unknown function	-	-	-	-	-	-	-	-	-	25	123646	60	0	6	0	9.5	5.86	0.05	Up-Detect	Up
gi|124808549	NOT family protein, putative	-	-	-	-	-	-	-	-	-	24	519499	72	0	30	0	9.1	6.88	0.01	Up-Detect	Up
gi|160736	vacuolar ATPase	-	-	-	-	-	-	-	-	-	24	68532	19	0	8	0	20.6	5.51	0.05	Up-Detect	Up
gi|124809489	poly(A)-specific ribonuclease PARN, putative	-	-	-	-	-	-	-	-	-	24	102192	14	0	11	0	15.5	7.72	0.03	Up-Detect	Up
gi|6562724	sporozoite invasion-associated protein 1	-	-	-	-	-	-	-	-	-	24	112862	14	0	10	0	13.7	6.27	0.03	Up-Detect	Up
gi|46361184	transcription or splicing factor-like protein,putative	-	-	-	-	-	-	-	-	-	24	70382	4	0	4	0	8.8	5.75	0.05	Up-Detect	Up
gi|23505262	Plasmodium exported protein (PHISTc), unknown function	-	-	-	-	-	-	-	-	-	24	45472	3	0	2	0	6.5	9.71	0.07	Up-Detect	Up
gi|1373027	apical membrane antigen-1, partial	-	-	-	-	-	-	-	-	-	24	68829	17	0	9	0	15.6	5.23	0.05	Up-Detect	Up
gi|74864044	RecName: Full=NAD-dependent protein deacetylase Sir2B; AltName: Full=Regulatory protein SIR2 homolog B; AltName: Full=SIR2-like protein B	-	-	-	-	-	-	-	-	-	23	154624	12	0	7	0	7.4	8.15	0.02	Up-Detect	Up
gi|356624409	Chain A, Translationally-controlled Tumor Protein Homolog	-	-	-	-	-	-	-	-	-	23	21594	4	0	4	0	14.8	4.9	0.16	Up-Detect	Up
gi|124810469	splicing factor 3A subunit 1, putative	-	-	-	-	-	-	-	-	-	23	83698	5	0	2	0	3.1	5.27	0.04	Up-Detect	Up
gi|23505236	TFIIH basal transcription factor complex helicase XPD subunit	-	-	-	-	-	-	-	-	-	22	122760	16	0	13	0	15.8	5.94	0.03	Up-Detect	Up
gi|124808162	conserved Plasmodium protein, unknown function	-	-	-	-	-	-	-	-	-	22	240981	41	0	23	0	12.2	9.51	0.01	Up-Detect	Up
gi|23505235	histidine--tRNA ligase, putative	-	-	-	-	-	-	-	-	-	22	78134	8	0	8	0	16.7	9.51	0.04	Up-Detect	Up
gi|23499096	conserved Plasmodium protein, unknown function	-	-	-	-	-	-	-	-	-	22	170170	27	0	10	0	8.6	6.4	0.04	Up-Detect	Up
gi|258597547	aspartyl protease, putative	-	-	-	-	-	-	-	-	-	21	77044	38	0	4	0	12	7.79	0.04	Up-Detect	Up
gi|23505153	proline--tRNA ligase, putative	-	-	-	-	-	-	-	-	-	21	68423	8	0	6	0	10.4	9.33	0.05	Up-Detect	Up
gi|258597812	conserved Plasmodium protein, unknown function	-	-	-	-	-	-	-	-	-	21	70994	25	0	10	0	18.3	9.15	0.05	Up-Detect	Up
gi|23615369	conserved Plasmodium membrane protein, unknown function	-	-	-	-	-	-	-	-	-	21	404731	76	0	38	0	12.5	8.76	0.01	Up-Detect	Up
gi|23498252	regulator of chromosome condensation, putative	-	-	-	-	-	-	-	-	-	21	236211	39	0	16	0	8.7	9.23	0.01	Up-Detect	Up
gi|23615188	conserved Plasmodium protein, unknown function	-	-	-	-	-	-	-	-	-	20	72454	5	0	3	0	5.1	6.55	0.05	Up-Detect	Up
gi|225631931	condensin complex subunit 2, putative	-	-	-	-	-	-	-	-	-	20	119143	17	0	11	0	15.3	4.87	0.06	Up-Detect	Up
gi|23499100	perforin-like protein 4	-	-	-	-	-	-	-	-	-	20	76298	9	0	9	0	18.7	8.61	0.04	Up-Detect	Up
gi|23505203	peptidyl-prolyl cis-trans isomerase	-	-	-	-	-	-	-	-	-	20	72507	8	0	4	0	5.4	9.11	0.05	Up-Detect	Up
gi|23615551	60S ribosomal protein L18, putative	-	-	-	-	-	-	-	-	-	19	21733	13	0	5	0	28.3	10.62	0.16	Up-Detect	Up
gi|46362277	conserved Plasmodium protein, unknown function	-	-	-	-	-	-	-	-	-	19	334204	44	0	26	0	11	8.49	0.01	Up-Detect	Up
gi|124806649	protein kinase 2	-	-	-	-	-	-	-	-	-	18	58876	18	0	13	0	35.6	8.77	0.06	Up-Detect	Up
gi|23505265	Plasmodium exported protein, unknown function	-	-	-	-	-	-	-	-	-	18	31267	6	0	3	0	8.9	9.69	0.11	Up-Detect	Up
gi|124809797	RAP protein, putative	-	-	-	-	-	-	-	-	-	18	208094	15	0	12	0	7.4	9.38	0.02	Up-Detect	Up
gi|124808735	nucleolar GTP-binding protein 2, putative	-	-	-	-	-	-	-	-	-	18	57136	8	0	5	0	11.7	9.72	0.06	Up-Detect	Up
gi|225632270	histone acetyltransferase GCN5	-	-	-	-	-	-	-	-	-	18	170810	23	0	11	0	10.3	6.25	0.02	Up-Detect	Up
gi|23504560	subtilisin-like protease 1	-	-	-	-	-	-	-	-	-	18	77598	14	0	8	0	12.6	5.68	0.04	Up-Detect	Up
gi|23615425	myosin C	-	-	-	-	-	-	-	-	-	18	250131	53	0	19	0	11	8.84	0.01	Up-Detect	Up
gi|124804772	60S ribosomal protein L35ae, putative	-	-	-	-	-	-	-	-	-	18	16255	2	0	2	0	11.4	10.55	0.21	Up-Detect	Up
gi|15375380	P-loop containing nucleoside triphosphate hydrolase, putative	-	-	-	-	-	-	-	-	-	17	132116	39	0	12	0	13.2	5.07	0.02	Up-Detect	Up
gi|258597726	conserved Plasmodium protein, unknown function	-	-	-	-	-	-	-	-	-	17	248348	46	0	17	0	9.2	9.01	0.01	Up-Detect	Up
gi|124806112	phospholipid-transporting ATPase, putative	-	-	-	-	-	-	-	-	-	17	190326	22	0	11	0	11.2	8.74	0.02	Up-Detect	Up

gi|23505002	conserved Plasmodium protein, unknown function	-	-	-	-	-	-	-	-	-	16	368118	31	0	21	0	9.5	5.27	0.01	Up-Detect	Up
gi|23498863	conserved Plasmodium protein, unknown function	-	-	-	-	-	-	-	-	-	16	205848	19	0	12	0	7.5	9.17	0.02	Up-Detect	Up
gi|23498942	conserved Plasmodium protein, unknown function	-	-	-	-	-	-	-	-	-	16	114757	4	0	3	0	3.6	9.19	0.03	Up-Detect	Up
gi|23504638	phosphatidylinositol 3-kinase	-	-	-	-	-	-	-	-	-	15	255758	30	0	15	0	8.2	9.27	0.01	Up-Detect	Up
gi|124806958	conserved Plasmodium membrane protein, unknown function	-	-	-	-	-	-	-	-	-	14	81491	24	0	5	0	10.6	9.45	0.04	Up-Detect	Up
