# Supplementary material for: Proteomic analysis of Plasmodium falciparum response to isocryptolepine derivative
Source: PLoS One. 2019 Aug 8;14(8):e0220871. doi: 10.1371/journal.pone.0220871 (PMC6687117; doi:10.1371/journal.pone.0220871)
Supplement: S3 File — Total proteins were identified and quantified by Mascot daemon version 2.3.2 software. The protein database was collected from an NCBInr database (24 October 2018) specific to P. falciparum 3D7. (PDF) [file pone.0220871.s003.pdf]

Supplement 3 file. Protein identification from differential proteomics experiment replication 3

Total proteins were identified and quantified by Mascot daemon version 2.3.2 software.

Protein database was collected from NCBInr database (24 October 2018) specific to *P. falciparum* 3D7

Rep.3		Rep.3 DMSO									Rep.3 ICL-M									ICL-M	ICL-M
NCBI acc_no	All prot_desc	prot_score	prot_mass	prot_matc	prot_matc	prot_sequ	prot_sequ	prot_cove	prot_pi	emPAI	prot_score	prot_mass	prot_matc	prot_matc	prot_sequ	prot_sequ	prot_cove	prot_pi	emPAI	Fold change	Up/Down
gi|23499105	heat shock protein 70	6134	73868	251	193	35	30	49.3	5.51	5.7	6476	73868	267	209	32	29	43.7	5.51	6.01	1.05	
gi|23505079	heat shock protein 70	4154	72343	208	127	28	19	51.4	5.18	2.58	4848	72343	221	137	25	19	43.9	5.18	2.76	1.07	
gi|124810131	glyceraldehyde-3-phosphate dehydrogenase	3333	36612	100	80	21	19	74.5	7.59	9.99	3876	36612	108	92	23	19	76.3	7.59	9.99		
gi|50400239	RecName: Full=Enolase; AltName: Full=2-phospho-D-glycerate hydro-lyase; AltName: Full=2-phosphoglycerate dehydratase	2872	48647	97	61	29	21	63.5	6.21	5.16	2553	48647	87	57	26	20	65	6.21	6.08	1.18	
gi|23615698	elongation factor 1-alpha	2611	48928	195	117	23	15	56.7	9.12	4.3	2796	48928	200	115	18	14	48.3	9.12	3.94	0.92	
gi|371941768	Hsp70-x	2194	75006	97	75	13	6	19.6	5.59	0.51	2182	75006	111	77	15	6	24.4	5.59	0.44	0.86	
gi|225632282	protein disulfide isomerase	1965	55479	92	64	17	10	39.5	5.56	1.84	1497	55479	70	48	13	8	32.5	5.56	1.67	0.91	
gi|23498766	heat shock protein 90	1813	86112	105	58	24	13	35	4.94	1.22	1360	86112	92	47	27	13	32.8	4.94	0.97	0.80	
gi|124803934	GTP-binding nuclear protein RAN/TC4	1240	24860	40	30	12	8	63.1	7.72	4.71	1197	24860	46	31	13	9	61.7	7.72	4.71		
gi|124803500	histone H2B	1233	13117	108	56	14	13	74.4	10.27	284.27	1304	13117	128	72	13	11	73.5	10.27	173.48	0.61	
gi|124809712	elongation factor 2	1221	93462	39	29	14	5	23.6	6.36	0.2	1337	93462	40	25	12	6	18.9	6.36	0.29	1.45	
gi|23615406	L-lactate dehydrogenase	1125	34086	56	33	16	11	48.1	7.12	4.93	1290	34086	51	37	13	12	40.8	7.12	4.93		
gi|46361043	ornithine aminotransferase	1059	46025	42	28	15	11	34.8	6.47	2.02	837	46025	39	22	16	8	30.7	6.47	1.61	0.80	
gi|31505529	histone H4, partial	1039	11448	47	31	8	6	66	11.23	20.44	1401	11448	59	40	8	6	66	11.23	27.33	1.34	
gi|344189571	Chain A, Phosphoglycerate Kinase	979	46264	63	32	31	11	60.1	7.82	2.01	1246	46264	68	39	33	13	67	7.82	2.48	1.23	
gi|129926	RecName: Full=Phosphoglycerate kinase	979	45398	46	32	19	11	54.6	7.63	2.07	1246	45398	50	39	21	13	64.7	7.63	2.56	1.24	
gi|23505200	merozoite surface protein 1	910	195605	60	24	28	12	20.6	6.11	0.3	779	195605	47	27	24	10	17.8	6.11	0.26	0.87	
gi|23498286	40S ribosomal protein S19	876	19710	25	20	7	7	38.8	10.17	3.5	553	19710	23	15	6	5	38.8	10.17	2.8	0.80	
gi|74920225	RecName: Full=Fructose-bisphosphate aldolase	842	40080	39	26	15	11	49.6	8.33	2.86	1084	40080	53	33	22	15	64	8.33	5.98	2.09	Up
gi|124806075	endoplasmin, putative	738	94959	48	21	16	7	26.1	5.28	0.29	928	94959	48	21	20	9	29.8	5.28	0.44	1.52	
gi|23615568	phosphoethanolamine N-methyltransferase	632	31024	21	16	8	6	39.1	5.43	0.92	531	31024	23	14	9	6	39.5	5.43	1.14	1.24	
gi|23504519	actin-depolymerizing factor 1	569	13732	14	12	2	2	18	7.66	0.6	649	13732	16	12	4	2	32.8	7.66	0.6		
gi|46361058	histone H3	544	15437	81	30	12	6	49.3	11.14	5.68	691	15437	103	43	13	7	51.5	11.14	5.68		
gi|23498886	40S ribosomal protein S5, putative	505	21849	16	12	7	6	37.9	9.67	2.36	254	21849	11	6	5	3	33.3	9.67	1.13	0.48	Down
gi|124804377	60S ribosomal protein P0	445	34945	24	15	12	8	42.7	6.28	2.18	479	34945	26	14	13	7	50.9	6.28	1.38	0.63	
gi|4494010	histone H2A variant, putative	402	16443	27	16	3	3	23.4	10.63	3.02	486	16443	36	21	4	3	29.7	10.63	1.22	0.40	Down
gi|74876423	RecName: Full=Tubulin beta chain; AltName: Full=Beta-tubulin	389	49719	19	9	13	5	38.9	4.73	0.41	264	49719	19	8	10	3	36.4	4.73	0.23	0.56	
gi|225632253	14-3-3 protein	383	30174	31	16	13	7	48.5	4.86	2.8	580	30174	34	18	14	6	52.7	4.86	1.43	0.51	
gi|303324901	Chain A, Heat Shock Protein 86	364	25195	24	12	5	1	22.9	4.85	0.49	373	25195	25	14	7	3	28.7	4.85	0.49		
gi|225631960	40S ribosomal protein S19	351	16753	8	6	2	2	20.7	10.27	0.48	215	16753	5	3	2	1	20.7	10.27	0.22	0.46	Down
gi|46361129	histone H2A	346	14114	24	14	5	2	43.9	10.29	0.58	397	14114	33	19	4	2	34.8	10.29	0.99	1.71	
gi|4493980	peptidyl-prolyl cis-trans isomerase	345	18940	15	11	7	4	50.3	8.29	1.83	430	18940	19	15	5	5	31	8.29	3.01	1.64	
gi|124808810	60S ribosomal protein L21	324	18783	24	11	5	1	37.3	10.04	0.42	477	18783	34	17	3	1	19.3	10.04	0.42		
gi|75009812	RecName: Full=Triosephosphate isomerase; Short=TIM; AltName: Full=Triose-phosphate isomerase	317	27917	20	10	12	5	36.3	6.01	1.61	322	27917	17	10	9	6	31.9	6.01	1.61		
gi|225631678	60S ribosomal protein L12, putative	313	18101	17	10	6	2	36.4	9.54	0.44	356	18101	19	12	4	2	30.3	9.54	0.44		
gi|46361162	pyridoxine biosynthesis protein PDX1	312	32992	17	8	12	6	46.2	6.76	1.04	276	32992	13	5	8	2	29.9	6.76	0.5	0.48	Down
gi|124803860	peptidyl-prolyl cis-trans isomerase	308	21717	23	10	14	5	69.2	7.1	1.91	343	21717	21	11	9	6	54.4	7.1	2.38	1.25	
gi|46361220	pyruvate kinase	302	55625	21	8	10	2	32.9	7.5	0.13	309	55625	19	9	10	2	26.4	7.5	0.28	2.15	Up
gi|23505159	conserved Plasmodium protein, unknown function	293	24683	13	11	4	3	27.2	5.49	0.72	298	24683	16	11	5	2	33.2	5.49	0.5	0.69	
gi|225631753	cell division cycle protein 48 homologue,putative	289	92329	26	10	13	4	20.7	4.95	0.16	163	92329	19	3	11	1	18	4.95	0.04	0.25	Down
gi|258597456	nucleosome assembly protein	284	40463	11	7	3	1	11.2	4.76	0.09	335	40463	12	8	3	1	14.7	4.76	0.09		
gi|23499152	DNA/RNA-binding protein Alba 1	269	27242	38	16	11	6	42.3	10.58	2.03	240	27242	25	11	8	3	28.2	10.58	0.64	0.32	Down
gi|124800689	knob-associated histidine-rich protein	269	71259	16	7	6	2	11.2	9.17	0.15	309	71259	19	10	7	3	12.4	9.17	0.21	1.40	
gi|23615172	40S ribosomal protein S7, putative	266	22467	15	7	5	2	30.4	9.81	0.56	205	22467	14	8	2	1	16.5	9.81	0.16	0.29	Down
gi|124802054	DNA/RNA-binding protein Alba 3	261	11969	21	9	7	3	72	9.3	1.91	336	11969	16	10	8	5	74.8	9.3	7.45	3.90	Up
gi|124805752	glutathione peroxidase-like thioredoxin peroxidase	254	23937	14	7	3	1	16.6	8.99	0.15	302	23937	15	7	4	1	16.6	8.99	0.15		
gi|124808442	60S ribosomal protein L10, putative	253	25200	16	5	8	1	40.6	9.97	0.3	320	25200	16	6	7	1	26	9.97	0.14	0.47	Down

gi|124804166	splicing factor, putative	241	16002	7	5	2	1	20	5.1	0.5	301	16002	7	6	1	1	13.1	5.1	0.5		
gi|45478047	macrophage migration inhibitory factor-like protein	238	12836	16	8	7	4	54.3	6.15	1.72	396	12836	19	13	5	5	49.1	6.15	2.49	1.45	
gi|46361130	histone H3 variant, putative	236	15432	74	20	10	4	47.1	11.15	2.55	235	15432	90	27	9	3	39	11.15	1.33	0.52	
gi|23499115	high mobility group protein B2	235	11527	9	7	4	3	42.4	9.97	2.02	290	11527	8	7	4	3	42.4	9.97	2.02		
gi|124802119	adenylate kinase	221	27594	16	10	10	6	40.5	8.97	1.97	306	27594	14	12	7	7	27.7	8.97	1.63	0.83	
gi|23504618	purine nucleoside phosphorylase	205	26841	16	5	7	3	31	6.07	0.65	261	26841	13	8	7	5	29.8	6.07	0.86	1.32	
gi|282403624	Chain A, Uridine Phosphorylase, Putative	205	30453	17	5	8	3	36.2	5.83	0.56	-	-	-	-	-	-	-	-	-	Down-Detect	Down
gi|23498814	histone H2B variant	198	13755	26	14	11	4	74.8	10.8	1.56	209	13755	28	12	7	2	56.9	10.8	1.02	0.65	
gi|23504938	alpha tubulin 1	196	50264	6	4	2	1	7.1	4.93	0.14	124	50264	4	2	3	1	11.5	4.93	0.07	0.50	Down
gi|23499195	acyl-CoA binding protein, putative	193	10768	8	6	3	1	48.9	7.68	0.8	142	10768	7	6	2	1	38.9	7.68	0.8		
gi|124804238	40S ribosomal protein S18, putative	186	17880	16	8	6	3	45.5	10.46	1.09	143	17880	14	8	6	3	49.4	10.46	0.74	0.68	
gi|258597201	40S ribosomal protein S4, putative	185	29753	7	5	3	2	17.6	10.09	0.25	193	29753	10	4	6	2	26.4	10.09	0.25		
gi|23498770	heat shock protein 110	181	99902	38	5	10	2	13.4	5.54	0.11	95	99902	39	3	14	3	16	5.54	0.11		
gi|4493906	40S ribosomal protein S12, putative	181	15387	11	6	6	3	46.1	4.9	0.88	154	15387	8	4	5	3	39	4.9	1.33	1.51	
gi|124803848	cysteine proteinase falcipain 2b	181	55768	16	8	8	3	17.2	8.14	0.44	222	55768	13	8	8	3	22	8.14	0.28	0.64	
gi|124803863	cysteine proteinase falcipain 2a	181	55892	15	8	8	3	22.7	7.12	0.44	222	55892	12	8	7	3	20.2	7.12	0.28	0.64	
gi|23504494	mature parasite-infected erythrocyte surface antigen	179	168186	31	6	23	5	17.6	4.76	0.15	239	168186	31	6	23	5	16.3	4.76	0.11	0.73	
gi|75016040	RecName: Full=Acidic leucine-rich nuclear phosphoprotein 32-related protein; AltName: Full=ANP32/acidic nuclear phosphoprotein-like protein	177	32986	9	6	4	3	18.9	4.27	0.5	106	32986	7	6	2	2	7.8	4.27	0.36	0.72	
gi|124809152	mitochondrial acidic protein MAM33, putative	172	28854	22	6	7	3	29.4	4.89	0.42	130	28854	17	5	5	2	20.2	4.89	0.42		
gi|124810348	exported protein 2	161	33391	12	5	6	2	22.3	5.1	0.35	107	33391	6	4	3	2	12.5	5.1	0.35		
gi|23504595	histamine-releasing factor	158	19967	7	4	3	2	16.4	4.58	0.64	173	19967	7	4	3	1	16.4	4.58	0.39	0.61	
gi|23615594	DNA/RNA-binding protein Alba 2	158	24969	12	4	7	2	37	7.68	1.23	103	24969	10	3	7	2	39.3	7.68	0.71	0.58	
gi|23504648	40S ribosomal protein S11	152	16067	19	4	5	2	35.8	10.49	0.84	95	16067	18	3	9	2	58.3	10.49	0.84		
gi|124804024	phosphoglycerate mutase, putative	152	28752	19	9	10	5	48.8	8.3	0.79	151	28752	15	6	7	3	40.8	8.3	0.79		
gi|258596854	stevor	151	34204	12	6	3	1	11.8	8.76	0.1	155	34204	13	7	2	1	6.6	8.76	0.1		
gi|23504955	high molecular weight rhoptry protein 3	150	104789	16	4	8	4	11.8	6.25	0.14	149	104789	10	2	6	1	8.7	6.25	0.03	0.21	Down
gi|23504687	40S ribosomal protein S9, putative	149	22109	10	4	5	2	34.4	10.45	0.35	188	22109	13	6	5	2	25.4	10.45	0.35		
gi|124810293	eukaryotic initiation factor 4A	147	45281	15	3	6	1	20.6	5.48	0.16	135	45281	19	3	9	1	30.4	5.48	0.08	0.50	Down
gi|124810210	40S ribosomal protein S3	144	24652	12	5	7	2	31.7	10.2	0.5	48	24652	9	1	7	1	31.2	10.2	0.14	0.28	Down
gi|11127605	heat shock protein hsp70 homologue Pfhsp70-3	142	71546	26	4	16	4	32.6	5.9	0.27	95	71546	22	4	12	2	27.5	5.9	0.15	0.56	
gi|6851056	spermidine synthase	139	36573	13	4	8	2	30.5	6.97	0.2	-	-	-	-	-	-	-	-	-	Down-Detect	Down
gi|116668029	Chain A, Spermidine Synthase	139	32162	13	4	8	2	34.6	6.18	0.23	110	32162	13	5	4	2	24.7	6.18	0.23		
gi|23615654	thioredoxin-related protein, putative	134	23972	7	4	4	3	20.7	9.44	1	29	23972	3	1	2	1	13.5	9.44	0.15	0.15	Down
gi|124810100	40S ribosomal protein S28e, putative	131	7489	6	5	3	2	50.7	10.83	2.42	329	7489	12	11	3	3	40.3	10.83	4.16	1.72	
gi|23504496	Plasmodium exported protein, unknown function	128	30696	4	3	2	1	9.6	9.47	0.24	85	30696	3	2	1	1	4.2	9.47	0.24		
gi|124801997	60S ribosomal protein L13, putative	127	23739	16	4	8	2	37.1	10.19	0.32	35	23739	12	1	4	1	18.3	10.19	0.15	0.47	Down
gi|23504725	karyopherin beta	122	127272	10	3	8	2	10.5	4.8	0.06	61	127272	14	1	10	1	14.1	4.8	0.06		
gi|237640532	Chain A, HAP protein	122	37376	15	4	9	3	28.9	4.97	0.44	-	-	-	-	-	-	-	-	-	Down-Detect	Down
gi|124808181	plasmepsin III	122	51661	17	4	10	3	25.1	8.05	0.3	76	51661	8	2	5	1	12.9	8.05	0.07	0.23	Down
gi|23504556	60S ribosomal protein L4	119	46183	12	3	8	3	25.3	10.5	0.34	-	-	-	-	-	-	-	-	-	Down-Detect	Down
gi|23499154	60S ribosomal protein L13-2, putative	116	25425	6	3	4	2	22.3	10.78	0.3	-	-	-	-	-	-	-	-	-	Down-Detect	Down
gi|225632259	U6 snRNA-associated Sm-like protein LSm3,putative	115	10694	4	3	2	1	26.4	5.16	0.35	108	10694	6	3	4	1	58.2	5.16	0.35		
gi|23615723	40S ribosomal protein S15	115	17240	14	6	6	4	46.4	10.38	1.59	86	17240	15	2	9	2	58.9	10.38	1.59		
gi|46361040	proteasome subunit alpha type-2, putative	111	26512	3	3	2	2	11.1	5.4	0.46	107	26512	8	4	3	2	11.9	5.4	0.46		
gi|124808201	40S ribosomal protein S8e, putative	110	25035	10	3	4	2	20.2	9.98	0.7	42	25035	2	1	2	1	11.5	9.98	0.14	0.20	Down
gi|124806612	conserved protein, unknown function	109	23644	12	4	4	1	25.2	9.64	0.15	20	23644	10	0	5	0	23.8	9.64	0.15		
gi|1575675	rab6	108	27914	5	4	3	3	12.9	7.63	0.43	39	27914	3	1	2	1	7.5	7.63	0.13	0.30	Down
gi|124802073	single-strand telomeric DNA-binding protein GBP2, putative	106	29512	8	3	4	1	19.5	9.2	0.26	89	29512	7	2	4	1	18.7	9.2	0.26		
gi|124804079	exported protein 1	102	17285	7	2	3	1	35.8	5.64	0.21	70	17285	2	2	1	1	11.1	5.64	0.21		
gi|10129955	S-adenosylmethionine synthetase	100	44816	10	2	7	2	22.1	6.28	0.16	172	44816	5	3	3	2	9.7	6.28	0.16		
gi|124809201	glucose-6-phosphate isomerase	100	67325	19	4	9	2	17.6	6.78	0.11	-	-	-	-	-	-	-	-	-	Down-Detect	Down
gi|7768287	formate-nitrite transporter	98	34436	18	3	5	1	7.4	8.74	0.1	149	34436	16	5	3	2	8.7	8.74	0.22	2.20	Up
gi|124805478	eukaryotic translation initiation factor 5A	98	17620	9	3	3	2	32.3	5.42	0.45	63	17620	4	2	4	2	36	5.42	0.45		
gi|258549116	serine esterase, putative	96	216941	63	1	22	1	18.1	8.82	0.02	90	216941	57	2	16	1	13.9	8.82	0.02		
gi|23498728	Plasmodium exported protein, unknown function	95	27656	5	2	3	2	17.4	8.55	0.27	77	27656	7	3	4	1	28.9	8.55	0.13	0.48	Down

gi|23615603	nucleoside transporter 1	94	47600	7	5	2	2	2.8	8.36	0.15	135	47600	7	6	2	1	2.8	8.36	0.07	0.47	Down
gi|23615478	20 kDa chaperonin	94	29045	2	2	1	1	4.7	7.63	0.12	67	29045	1	1	1	1	4.7	7.63	0.12		
gi|23615498	merozoite surface protein 7	90	41251	8	2	3	1	11.1	4.74	0.09	146	41251	12	3	4	1	16.8	4.74	0.28	3.11	Up
gi|23505090	nucleosome assembly protein	90	31807	4	3	2	1	9.7	4.17	0.11	37	31807	6	1	4	1	16.7	4.17	0.24	2.18	Up
gi|23505054	6-phosphofructokinase	90	159351	24	4	11	3	10.6	6.32	0.07	56	159351	23	2	13	2	11.4	6.32	0.07		
gi|23498140	Plasmodium exported protein (PHISTb), unknown function	89	60233	17	3	7	3	12.7	8.76	0.25	88	60233	17	2	7	1	13.6	8.76	0.12	0.48	Down
gi|23615606	DNA/RNA-binding protein Alba 4	88	42133	9	2	5	1	14.2	7.14	0.08	70	42133	6	2	3	1	7	7.14	0.08		
gi|124809606	40S ribosomal protein S5	88	29939	3	2	3	2	21.3	10.02	0.25	37	29939	8	1	5	1	15.1	10.02	0.25		
gi|258596875	26S proteasome regulatory subunit RPN1,putative	87	108289	37	2	14	1	21.4	5.95	0.03	35	108289	35	1	14	1	20.1	5.95	0.03		
gi|124809402	60S ribosomal protein L1, putative	87	24790	9	4	5	2	24.4	9.85	0.5	121	24790	9	3	5	1	24.9	9.85	0.31	0.62	
gi|124806145	polyadenylate-binding protein, putative	85	97169	14	3	11	2	15.7	8.96	0.07	39	97169	7	1	7	1	8.8	8.96	0.04	0.57	
gi|23499261	1-cys peroxiredoxin	83	25148	12	4	6	2	29.1	6.31	0.49	86	25148	10	3	5	1	24.5	6.31	0.14	0.29	Down
gi|124802718	adenosine deaminase	83	42438	28	3	10	2	29.2	5.41	0.38	231	42438	30	10	14	8	35.4	5.41	1.22	3.21	Up
gi|23504603	inositol-3-phosphate synthase	83	69069	18	2	9	2	14.9	7.11	0.16	30	69069	15	0	8	0	13.1	7.11	0.1	0.63	
gi|23615408	phosphoribosylpyrophosphate synthetase	82	49352	11	3	6	3	18.1	9.39	0.23	151	49352	14	5	7	4	18.1	9.39	0.32	1.39	
gi|23615786	ubiquitin-60S ribosomal protein L40	81	14608	20	2	6	2	43	9.91	2.03	172	14608	18	6	8	4	52.3	9.91	2.03		
gi|23615715	HVA22-like protein, putative	81	18410	4	3	2	1	12.4	9.34	0.2	46	18410	5	2	2	1	11.8	9.34	0.2		
gi|47169189	Chain A, Uridine Phosphorylase, Putative	78	27745	16	2	9	2	44.3	6.32	0.44	188	27745	13	5	8	4	30	6.32	0.62	1.41	
gi|74929507	RecName: Full=Actin-1; AltName: Full=Actin I	78	41844	11	3	5	1	18.9	5.21	0.08	75	41844	13	2	6	1	24.7	5.21	0.18	2.25	Up
gi|124808771	60S ribosomal protein L5, putative	77	33977	3	2	3	2	11.9	9.78	0.22	94	33977	4	2	3	1	15	9.78	0.1	0.45	Down
gi|124808276	rhoptry-associated protein 1	77	89996	5	2	4	1	6.1	6.67	0.04	148	89996	12	4	10	2	15.3	6.67	0.12	3.00	Up
gi|23615526	60S ribosomal protein L6-2, putative	75	25516	5	3	3	2	14.9	10.1	0.48	76	25516	6	3	4	1	24.9	10.1	0.14	0.29	Down
gi|23505219	profilin, putative	75	19005	2	2	1	1	4.7	4.22	0.19	97	19005	3	3	1	1	4.7	4.22	0.19		
gi|23615363	conserved Plasmodium protein, unknown function	72	170566	68	0	16	0	14.8	8.86	0.04	55	170566	63	0	12	0	9.8	8.86	0.04		
gi|23498939	proteasome subunit alpha type-5, putative	71	28370	8	3	6	1	38.3	4.96	0.27	147	28370	14	8	4	1	26.6	4.96	0.42	1.56	
gi|651207688	Chain A, Serine Hydroxymethyltransferase	70	54007	16	2	11	1	24.2	7.21	0.07	39	54007	11	1	9	1	20.4	7.21	0.07		
gi|124806534	serine hydroxymethyltransferase	70	49749	11	2	9	1	21.3	8.29	0.07	39	49749	10	1	8	1	18.1	8.29	0.07		
gi|124803852	cysteine proteinase falcipain 3	66	56630	24	3	7	1	15.2	6.59	0.13	65	56630	22	2	7	1	15	6.59	0.2	1.54	
gi|23498142	Plasmodium exported protein (PHISTa), unknown function	65	49713	9	1	4	1	10.5	9.51	0.07	73	49713	9	2	6	1	14.3	9.51	0.07		
gi|8247298	hypothetical protein, partial	64	4755	3	2	2	2	60	9.23	2.31	71	4755	1	1	1	1	60	9.23	0.82	0.35	Down
gi|124809582	M17 leucyl aminopeptidase	64	67778	23	3	7	2	18.3	8.78	0.11	70	67778	26	2	8	1	22.5	8.78	0.11		
gi|23504898	lysine-rich membrane-associated PHISTb protein	63	61048	11	2	7	1	17	9.34	0.06	61	61048	10	2	8	1	15.7	9.34	0.06		
gi|124809914	V-type H(+)-translocating pyrophosphatase,putative	62	76367	16	2	6	1	11.9	6.14	0.09	83	76367	12	2	4	2	7.1	6.14	0.09		
gi|124802670	60S ribosomal protein L3	62	44193	6	2	6	2	23.3	10.21	0.17	50	44193	2	1	2	1	8.3	10.21	0.08	0.47	Down
gi|46361167	nascent polypeptide-associated complex subunit alpha, putative	61	20610	2	1	2	1	12	4.77	0.17	58	20610	4	1	4	1	30.4	4.77	0.17		
gi|3122763	RecName: Full=60S acidic ribosomal protein P2	61	11941	6	2	4	2	64.3	4.49	0.7	67	11941	5	1	4	1	60.7	4.49	0.7		
gi|46361227	protein DJ-1	60	20280	4	2	2	2	15.3	6.95	0.38	90	20280	6	3	2	2	15.3	6.95	0.38		
gi|124802223	hypoxanthine-guanine phosphoribosyltransferase	59	26346	9	0	4	0	16	7.59	0.14	49	26346	12	1	6	1	30.3	7.59	0.14		
gi|124804821	40S ribosomal protein S21	57	9140	1	1	1	1	11	8.66	0.41	73	9140	5	3	3	2	43.9	8.66	0.99	2.41	Up
gi|124803451	60S acidic ribosomal protein P1, putative	57	13006	6	2	1	1	13.6	4.57	0.64	37	13006	6	1	3	1	39	4.57	0.64		
gi|23498182	V-type proton ATPase subunit B	57	55753	6	2	5	1	16.2	5.46	0.06	78	55753	6	3	4	2	11.5	5.46	0.13	2.17	Up
gi|23504502	rhoptry-associated protein 2	56	46709	12	2	3	1	6.5	8.9	0.08	46	46709	17	1	6	1	19.6	8.9	0.08		
gi|8052274	elongation factor 1 (EF-1), putative	55	17695	3	1	1	1	5.8	4.5	0.2	27	17695	1	0	1	0	5.8	4.5	0.2		
gi|225632186	signal recognition particle subunit SRP9	54	12127	1	1	1	1	13.6	9.45	0.3	65	12127	4	1	4	1	59.2	9.45	0.3		
gi|124802973	ADP/ATP transporter on adenylate translocase	54	33705	4	2	3	2	10	9.68	0.22	35	33705	5	1	4	1	18.9	9.68	0.1	0.45	Down
gi|124804998	rifin	52	38701	12	3	5	1	18.9	8.96	0.09	62	38701	20	3	6	1	21.9	8.96	0.09		
gi|23504543	small ubiquitin-related modifier	52	11053	3	1	3	1	20	4.74	0.77	24	11053	2	0	2	0	20	4.74	0.33	0.43	Down
gi|7799189	thioredoxin	52	11709	21	3	3	2	34.6	4.67	1.27	201	11709	20	6	5	3	35.6	4.67	1.99	1.57	
gi|258597310	60S ribosomal protein L35, putative	50	14739	4	2	3	1	18.5	10.79	0.25	58	14739	4	2	2	1	12.1	10.79	0.25		
gi|124804373	60S ribosomal protein L38	50	10307	7	1	5	1	44.8	10.71	0.36	100	10307	8	4	3	2	28.7	10.71	1.5	4.17	Up
gi|124804546	conserved Plasmodium protein, unknown function	48	28478	17	0	3	0	15.1	8.8	0.12	35	28478	17	0	3	0	14.3	8.8	0.12		
gi|23499155	40S ribosomal protein S16, putative	48	16275	6	2	5	1	39.6	10.25	0.22	44	16275	4	2	3	1	20.8	10.25	0.22		
gi|23504672	WD repeat-containing protein 26, putative	48	150608	41	3	13	1	12.2	9.24	0.02	39	150608	34	3	11	1	11.1	9.24	0.02		
gi|3758867	proteasome subunit alpha type-3, putative	48	29270	11	2	7	1	35.7	6.38	0.12	44	29270	8	1	3	1	15.5	6.38	0.12		
gi|23498308	Plasmodium exported protein (PHISTb), unknown function	48	35939	4	2	3	1	13.6	8.75	0.1	88	35939	5	3	5	3	24.6	8.75	0.33	3.30	Up
gi|23498791	AAA family ATPase, CDC48 subfamily	47	141991	23	2	18	1	17.9	9.07	0.02	-	-	-	-	-	-	-	-	-	Down-Detect	Down

gi|124808815	basic transcription factor 3b, putative	47	19381	4	1	4	1	17	9.04	0.18	-	-	-	-	-	-	-	-	-	Down-Detect	Down
gi|23504624	ras-related protein Rab-1A	47	23844	17	1	6	1	33.3	6.89	0.15	-	-	-	-	-	-	-	-	-	Down-Detect	Down
gi|13375179	putative GTPase	47	22872	4	1	3	1	16	6.18	0.16	-	-	-	-	-	-	-	-	-	Down-Detect	Down
gi|124803892	folate transporter 2	47	51321	4	1	4	1	12.3	8.63	0.07	-	-	-	-	-	-	-	-	-	Down-Detect	Down
gi|59798920	RecName: Full=Serine-repeat antigen protein; AltName: Full=111 kDa antigen; AltName: Full=p126; Flags: Precursor	46	111698	24	1	16	1	18.8	5.26	0.03	41	111698	20	1	10	1	13.1	5.26	0.03		
gi|225632238	conserved Plasmodium protein, unknown function	46	36234	13	1	3	1	7.9	4.11	0.1	45	36234	9	1	3	1	7.9	4.11	0.1		
gi|124805527	conserved Plasmodium protein, unknown function	45	38994	17	1	3	1	12	4.84	0.09	-	-	-	-	-	-	-	-	-	Down-Detect	Down
gi|67463796	Chain A, Deoxyuridine 5~-triphosphate Nucleotidohydrolase	44	19561	2	1	2	1	17.3	6.53	0.18	42	19561	2	1	2	1	17.3	6.53	0.18		
gi|258597702	40S ribosomal protein S25	44	11656	5	1	4	1	49.5	10.12	0.31	-	-	-	-	-	-	-	-	-	Down-Detect	Down
gi|23615388	60S ribosomal protein L6, putative	43	21588	6	2	5	1	23.7	9.88	0.17	34	21588	3	1	3	1	15.8	9.88	0.17		
gi|124809308	thioredoxin peroxidase 1	43	21793	7	1	3	1	20.5	6.65	0.36	90	21793	13	2	4	1	21.5	6.65	0.36		
gi|23504681	40S ribosomal protein S24	42	15382	2	1	2	1	14.3	10.75	0.23	43	15382	2	1	1	1	6.8	10.75	0.23		
gi|124802189	proteasome subunit beta type-5	41	30577	4	1	3	1	13.7	5.18	0.12	46	30577	5	2	4	2	15.5	5.18	0.25	2.08	Up
gi|23615644	60S ribosomal protein L17, putative	41	23400	17	1	5	1	28.1	10.89	0.15	34	23400	16	1	5	1	21.2	10.89	0.15		
gi|46576603	RecName: Full=Probable cathepsin C; Flags: Precursor	40	80361	15	2	7	1	15	5.82	0.04	31	80361	20	1	8	1	16.6	5.82	0.04		
gi|225632239	conserved Plasmodium protein, unknown function	40	76808	16	1	7	1	13.2	5.34	0.05	48	76808	13	2	8	1	13.5	5.34	0.05		
gi|23498233	small GTP-binding protein sar1	39	22006	4	1	3	1	19.8	6.75	0.16	-	-	-	-	-	-	-	-	-	Down-Detect	Down
gi|225632182	conserved Plasmodium protein, unknown function	39	126994	18	2	13	1	12	5.28	0.03	-	-	-	-	-	-	-	-	-	Down-Detect	Down
gi|13397937	putative Rab2 GTPase	38	24394	4	2	3	1	23.9	6.33	0.15	-	-	-	-	-	-	-	-	-	Down-Detect	Down
gi|23476987	glycogen synthase kinase 3	38	51583	9	1	7	1	21.1	5.4	0.07	-	-	-	-	-	-	-	-	-	Down-Detect	Down
gi|23615340	conserved Plasmodium protein, unknown function	38	88519	16	3	9	1	15.4	9.07	0.04	29	88519	18	1	10	1	16.6	9.07	0.04		
gi|23504696	S-adenosyl-L-homocysteine hydrolase	37	53804	3	1	3	1	9	5.64	0.07	69	53804	11	2	8	1	21.3	5.64	0.07		
gi|124804435	T-complex protein 1 subunit alpha	36	60223	6	1	6	1	14.9	6.65	0.12	25	60223	7	0	5	0	10.3	6.65	0.12		
gi|23504857	Hsc70-interacting protein	36	51092	2	1	1	1	1.7	4.67	0.07	-	-	-	-	-	-	-	-	-	Down-Detect	Down
gi|225631740	60S ribosomal protein L19	35	21566	2	1	1	1	4.4	11.32	0.17	-	-	-	-	-	-	-	-	-	Down-Detect	Down
gi|124809822	6-phosphogluconate dehydrogenase,decarboxylating, putative	35	52960	12	1	6	1	14.7	6.58	0.14	29	52960	10	1	5	1	8.5	6.58	0.14		
gi|225631696	conserved Plasmodium protein, unknown function	34	1116481	122	1	84	1	11.1	9.37	0	-	-	-	-	-	-	-	-	-	Down-Detect	Down
gi|23505020	conserved Plasmodium protein, unknown function	34	188566	37	1	19	1	15.6	9.15	0.02	-	-	-	-	-	-	-	-	-	Down-Detect	Down
gi|23498915	conserved Plasmodium protein, unknown function	34	36570	4	2	3	1	9.9	8.79	0.1	-	-	-	-	-	-	-	-	-	Down-Detect	Down
gi|258597173	Pfmc-2TM Maurer~s cleft two transmembrane protein	34	27356	3	1	3	1	26	9.63	0.13	-	-	-	-	-	-	-	-	-	Down-Detect	Down
gi|258597663	nuclear transport factor 2, putative	34	14428	6	1	3	1	31.2	5.53	0.25	32	14428	6	1	4	1	48	5.53	0.25		
gi|124807078	rhoptry neck protein 3	34	262988	75	1	27	1	12.8	9.23	0.01	-	-	-	-	-	-	-	-	-	Down-Detect	Down
gi|124801981	40S ribosomal protein S20e, putative	34	13504	2	1	2	1	17.8	9.67	0.27	21	13504	2	0	2	0	16.9	9.67	0.27		
gi|224591381	Pfmc-2TM Maurer~s cleft two transmembrane protein	34	26882	3	2	3	2	28.4	9.71	0.28	-	-	-	-	-	-	-	-	-	Down-Detect	Down
gi|23499027	receptor for activated c kinase	34	35664	2	1	2	1	9.6	6.24	0.1	-	-	-	-	-	-	-	-	-	Down-Detect	Down
gi|23504959	conserved Plasmodium protein, unknown function	34	139901	22	1	10	1	11.7	8.75	0.02	-	-	-	-	-	-	-	-	-	Down-Detect	Down
gi|258597261	pyridoxine biosynthesis protein PDX2	34	24547	5	1	3	1	18.7	6.43	0.15	-	-	-	-	-	-	-	-	-	Down-Detect	Down
gi|258597176	erythrocyte membrane protein 1, PfEMP1	34	248280	77	8	21	1	10.6	5.47	0.01	33	248280	77	6	16	1	8	5.47	0.01		
gi|23504621	deoxyribodipyrimidine photo-lyase, putative	34	129117	24	8	9	1	8.8	9.22	0.03	33	129117	30	6	7	1	6	9.22	0.03		
gi|23615206	conserved Plasmodium protein, unknown function	34	56775	16	8	2	1	3.1	9.53	0.06	33	56775	20	6	2	1	3.3	9.53	0.06		
gi|225632293	Plasmodium exported protein, unknown function	33	36390	2	1	2	1	7.3	5.74	0.1	-	-	-	-	-	-	-	-	-	Down-Detect	Down
gi|23505080	glideosome-associated protein 50	33	44576	4	1	3	1	9.3	8.69	0.08	42	44576	8	2	6	2	16.9	8.69	0.26	3.25	Up
gi|14530178	Krueppel-like protein	33	151550	20	1	11	1	11.5	7.89	0.02	-	-	-	-	-	-	-	-	-	Down-Detect	Down
gi|74930131	RecName: Full=40S ribosomal protein SA	33	29837	10	1	6	1	22.8	5.91	0.12	43	29837	6	4	3	1	14.1	5.91	0.25	2.08	Up
gi|258597872	signal peptide peptidase	33	47547	6	1	4	1	13.3	8.95	0.07	42	47547	3	1	2	1	5.1	8.95	0.07		
gi|23505011	conserved Plasmodium protein, unknown function	33	137031	13	1	10	1	12.2	9	0.03	-	-	-	-	-	-	-	-	-	Down-Detect	Down
gi|74873111	RecName: Full=Uncharacterized protein PFB0145c	33	237599	82	1	27	1	16.4	5.77	0.01	-	-	-	-	-	-	-	-	-	Down-Detect	Down
gi|23504862	eukaryotic translation initiation factor 3 subunit E, putative	32	61379	15	1	5	1	9.7	7.08	0.12	49	61379	15	2	8	1	15.3	7.08	0.06	0.50	Down
gi|23498881	conserved Plasmodium protein, unknown function	32	29231	2	1	2	1	10.2	8.76	0.12	-	-	-	-	-	-	-	-	-	Down-Detect	Down
gi|225632017	conserved Plasmodium protein, unknown function	32	696614	105	1	45	1	9.5	7.88	0	29	696614	105	1	50	1	10.6	7.88	0		
gi|23476993	Plasmodium exported protein (hyp8), unknown function	31	28216	5	1	3	1	12.3	9.02	0.13	-	-	-	-	-	-	-	-	-	Down-Detect	Down
gi|258597955	conserved Plasmodium membrane protein, unknown function	31	577659	68	2	40	1	10.7	8.86	0.01	44	577659	74	2	35	1	8.1	8.86	0.01		
gi|17148533	Ran-binding protein	30	33176	6	1	2	1	10	4.92	0.11	44	33176	2	1	1	1	4.3	4.92	0.22	2.00	Up
gi|258597233	60S ribosomal protein L36	30	12779	2	1	2	1	17	11.07	0.29	-	-	-	-	-	-	-	-	-	Down-Detect	Down

gi|3694805	cytoadherence linked asexual protein, partial	30	160994	30	1	16	1	17	8.98	0.02	42	160994	33	2	11	1	8.8	8.98	0.02		
gi|23615192	DNA-directed RNA polymerase II subunit RPB11,putative	30	14116	3	1	2	1	23.8	8.37	0.26	27	14116	1	1	1	1	17.5	8.37	0.26		
gi|124802320	heat shock protein 60	30	62512	17	1	11	1	22.2	6.71	0.06	23	62512	19	0	12	0	26.9	6.71	0.06		
gi|23504716	multidrug resistance protein 1	30	162150	34	1	19	1	18.7	8.94	0.02	37	162150	33	0	15	0	12.3	8.94	0.04	2.00	Up
gi|225631857	conserved Plasmodium protein, unknown function	30	138544	27	0	10	0	10	8.44	0.03	45	138544	21	0	5	0	5.7	8.44	0.03		
gi|23504582	asparagine--tRNA ligase	30	85195	21	3	10	1	13.3	9.01	0.04	31	85195	23	3	10	1	11.8	9.01	0.08	2.00	Up
gi|18076407	early transcribed membrane protein 14.1	30	11420	1	1	1	1	12.1	9.63	0.32	66	11420	2	2	1	1	12.1	9.63	0.32		
gi|225631936	MORN repeat protein, putative	29	519893	86	0	29	0	7.3	9.15	0.01	-	-	-	-	-	-	-	-	-	Down-Detect	Down
gi|3649757	conserved Plasmodium protein, unknown function	29	202018	72	0	22	0	15.6	8.3	0.02	-	-	-	-	-	-	-	-	-	Down-Detect	Down
gi|124808756	conserved Plasmodium protein, unknown function	29	192484	89	0	17	0	10.8	9.67	0.02	-	-	-	-	-	-	-	-	-	Down-Detect	Down
gi|124806636	conserved Plasmodium protein, unknown function	29	212627	29	0	16	0	10.3	4.96	0.02	-	-	-	-	-	-	-	-	-	Down-Detect	Down
gi|23615179	sodium/hydrogen exchanger, Na+, H+ antiporter	29	225940	34	0	17	0	10.9	8.68	0.02	-	-	-	-	-	-	-	-	-	Down-Detect	Down
gi|124804384	conserved Plasmodium protein, unknown function	29	118257	23	0	12	0	11.9	8.84	0.03	-	-	-	-	-	-	-	-	-	Down-Detect	Down
gi|124805631	conserved Plasmodium protein, unknown function	29	208541	29	0	18	0	15.2	8.3	0.02	-	-	-	-	-	-	-	-	-	Down-Detect	Down
gi|23504575	conserved Plasmodium protein, unknown function	29	369953	40	0	24	0	8.4	5.29	0.01	-	-	-	-	-	-	-	-	-	Down-Detect	Down
gi|124804234	autophagy-related protein 7, putative	29	156530	21	0	11	0	10.7	6.05	0.02	-	-	-	-	-	-	-	-	-	Down-Detect	Down
gi|124809084	conserved Plasmodium protein, unknown function	29	152167	18	0	10	0	8.4	8.42	0.02	-	-	-	-	-	-	-	-	-	Down-Detect	Down
gi|124802600	conserved Plasmodium protein, unknown function	29	190279	19	0	11	0	9.7	8.29	0.02	-	-	-	-	-	-	-	-	-	Down-Detect	Down
gi|124804432	conserved Plasmodium protein, unknown function	29	133324	24	0	11	0	17.4	5.18	0.03	-	-	-	-	-	-	-	-	-	Down-Detect	Down
gi|225632254	rRNA-processing protein FCF1, putative	29	23145	9	0	2	0	12.6	9.63	0.15	-	-	-	-	-	-	-	-	-	Down-Detect	Down
gi|23498865	60S ribosomal protein L11a, putative	29	20215	4	1	3	1	17.9	10.1	0.39	35	20215	4	1	3	1	17.9	10.1	0.39		
gi|23615434	proteasome subunit beta type-7, putative	28	29942	4	1	3	1	13	7.98	0.12	33	29942	6	1	5	1	27	7.98	0.12		
gi|23498743	tRNA m5C-methyltransferase, putative	28	141140	29	1	15	1	17	6.35	0.02	-	-	-	-	-	-	-	-	-	Down-Detect	Down
gi|46362284	conserved Plasmodium protein, unknown function	28	25357	21	0	4	0	10.5	9.82	0.14	26	25357	18	0	3	0	10	9.82	0.14		
gi|4493905	40S ribosomal protein S23, putative	27	16120	13	1	3	1	29.7	10.83	0.22	47	16120	14	1	4	1	31.7	10.83	0.5	2.27	Up
gi|23505139	RNA-binding protein, putative	27	22965	8	0	4	0	20.3	9.51	0.16	-	-	-	-	-	-	-	-	-	Down-Detect	Down
gi|3649767	SECIS-binding protein 2, putative	27	71419	10	0	7	0	12.2	9.94	0.05	27	71419	10	0	7	0	12	9.94	0.05		
gi|23615256	meiosis-specific nuclear structural protein 1,putative	27	61194	10	0	6	0	9.9	8.91	0.06	27	61194	10	0	6	0	9.9	8.91	0.06		
gi|124805363	Plasmodium exported protein (PHISTb), unknown function	27	77069	10	0	4	0	6.3	4.4	0.05	-	-	-	-	-	-	-	-	-	Down-Detect	Down
gi|23498201	phosphoglucomutase-2	27	35210	3	1	2	1	16.3	9.52	0.1	-	-	-	-	-	-	-	-	-	Down-Detect	Down
gi|23615263	M1-family alanyl aminopeptidase	27	125983	9	0	8	0	10	7.3	0.03	52	125983	20	1	16	1	15.1	7.3	0.06	2.00	Up
gi|124803477	uncharacterized protein PF3D7_1104400	27	49235	7	0	6	0	21	9.18	0.07	37	49235	6	1	5	1	14.2	9.18	0.07		
gi|6562716	conserved Plasmodium protein, unknown function	26	229981	28	0	13	0	9.2	6.44	0.02	20	229981	29	0	16	0	10.1	6.44	0.02		
gi|124806350	subpellicular microtubule protein 2, putative	26	30171	8	0	4	0	17.8	9.31	0.12	25	30171	7	0	1	0	5	9.31	0.12		
gi|23615806	secreted ookinete protein, putative	26	135752	63	0	11	0	10.6	4.66	0.03	21	135752	63	0	7	0	8.5	4.66	0.03		
gi|124801947	RNA-binding protein, putative	26	30033	6	0	4	0	16.9	10.07	0.12	-	-	-	-	-	-	-	-	-	Down-Detect	Down
gi|23615660	Plasmodium exported protein, unknown function	26	32763	6	0	4	0	18.6	9.23	0.23	16	32763	6	0	4	0	20.7	9.23	0.11	0.48	Down
gi|124810028	conserved Plasmodium protein, unknown function	25	118694	24	0	7	0	9.8	8.96	0.03	27	118694	30	0	7	0	9.1	8.96	0.03		
gi|23615683	serine/threonine protein phosphatase 5	25	76840	10	0	7	0	13.5	5.06	0.05	-	-	-	-	-	-	-	-	-	Down-Detect	Down
gi|7672216	phosphatidylinositol 3-and 4-kinase, putative	25	110490	18	0	10	0	15.2	6.16	0.03	-	-	-	-	-	-	-	-	-	Down-Detect	Down
gi|23498891	rhoptry-associated leucine zipper-like protein 1	25	87825	27	0	8	0	13	5.31	0.04	23	87825	27	0	9	0	12.4	5.31	0.04		
gi|46361062	transketolase	25	75767	3	0	3	0	4.6	6.5	0.05	39	75767	6	1	5	1	9.8	6.5	0.05		
gi|23615173	conserved Plasmodium protein, unknown function	24	95550	45	0	9	0	15.6	9.45	0.04	32	95550	40	0	5	0	7.5	9.45	0.04		
gi|23504632	conserved Plasmodium protein, unknown function	24	190407	30	0	12	0	8.5	9.71	0.02	16	190407	26	0	12	0	9	9.71	0.02		
gi|23498787	60S ribosomal protein L34	24	17340	2	0	2	0	10.7	10.77	0.21	-	-	-	-	-	-	-	-	-	Down-Detect	Down
gi|225631797	AP-4 complex subunit epsilon, putative	24	161304	27	0	15	0	13.6	5.44	0.02	28	161304	24	0	13	0	11.2	5.44	0.02		
gi|258596969	conserved Plasmodium protein, unknown function	24	14508	6	0	4	0	20.5	6.09	0.25	35	14508	4	1	2	1	15	6.09	0.25		
gi|23504580	RNA polymerase I	24	340462	46	0	30	0	12.1	5.75	0.01	30	340462	41	1	22	1	8.1	5.75	0.01		
gi|124806008	cell cycle associated protein, putative	23	323873	35	0	24	0	11.1	9.26	0.01	-	-	-	-	-	-	-	-	-	Down-Detect	Down
gi|124810087	conserved Plasmodium protein, unknown function	23	59322	10	0	7	0	23	5.28	0.06	-	-	-	-	-	-	-	-	-	Down-Detect	Down
gi|225631639	histone acetyltransferase, putative	23	146635	25	0	10	0	9.1	6.29	0.02	-	-	-	-	-	-	-	-	-	Down-Detect	Down
gi|75009813	RecName: Full=Plasmepsin-1; AltName: Full=Aspartic hemoglobinase I; AltName: Full=PfAPG; Flags: Precursor	23	51428	4	0	3	0	15.7	6.72	0.07	-	-	-	-	-	-	-	-	-	Down-Detect	Down
gi|23615547	RNA lariat debranching enzyme, putative	23	67513	37	0	5	0	9.9	5.21	0.05	-	-	-	-	-	-	-	-	-	Down-Detect	Down
gi|124802645	conserved Plasmodium protein, unknown function	23	196369	32	0	15	0	9.6	9.26	0.02	-	-	-	-	-	-	-	-	-	Down-Detect	Down
gi|46361188	hexokinase	22	55226	21	0	7	0	19.7	6.72	0.06	-	-	-	-	-	-	-	-	-	Down-Detect	Down
gi|124805429	high mobility group protein B1	22	11324	2	0	2	0	15.5	9.96	0.32	-	-	-	-	-	-	-	-	-	Down-Detect	Down

gi|23499218	ras-related protein Rab-18	22	23148	1	0	1	0	5.5	8.11	0.15	-	-	-	-	-	-	-	-	-	Down-Detect	Down
gi|23504877	conserved Plasmodium protein, unknown function	22	225390	37	0	19	0	9.8	6.01	0.02	-	-	-	-	-	-	-	-	-	Down-Detect	Down
gi|75016029	RecName: Full=STI1-like protein	22	66015	10	0	5	0	12.2	6.63	0.05	-	-	-	-	-	-	-	-	-	Down-Detect	Down
gi|23498929	DNA mismatch repair protein PMS1, putative	22	156741	9	0	6	0	6.4	5.48	0.02	-	-	-	-	-	-	-	-	-	Down-Detect	Down
gi|258597681	conserved protein, unknown function	21	49106	17	0	2	0	10.1	9.61	0.07	-	-	-	-	-	-	-	-	-	Down-Detect	Down
gi|4493872	cytoadherence linked asexual protein 3.1	21	167134	23	0	14	0	13.2	6.75	0.02	-	-	-	-	-	-	-	-	-	Down-Detect	Down
gi|7340797	cytoadherence linked asexual protein 3.2	21	167382	22	0	13	0	11.6	6.74	0.02	-	-	-	-	-	-	-	-	-	Down-Detect	Down
gi|23615502	conserved Plasmodium protein, unknown function	21	48840	2	0	2	0	4.9	9	0.07	-	-	-	-	-	-	-	-	-	Down-Detect	Down
gi|23499061	RNA-binding protein, putative	21	32421	3	0	3	0	11.5	9.11	0.11	81	32421	8	2	4	1	15	9.11	0.37	3.36	Up
gi|74864044	RecName: Full=NAD-dependent protein deacetylase Sir2B; AltName: Full=Regulatory protein SIR2 homolog B; AltName: Full=SIR2-like protein B	21	154624	24	0	16	0	15.4	8.15	0.02	23	154624	19	0	11	0	11.2	8.15	0.02		
gi|23498839	eukaryotic translation initiation factor 3 subunit I, putative	21	37261	6	0	5	0	18.3	6.43	0.09	-	-	-	-	-	-	-	-	-	Down-Detect	Down
gi|23498907	conserved Plasmodium protein, unknown function	21	260606	34	0	13	0	7.3	5.49	0.01	19	260606	43	0	21	0	10.8	5.49	0.01		
gi|124809814	serine/threonine protein kinase KIN	20	90798	22	0	8	0	8.6	9.7	0.04	-	-	-	-	-	-	-	-	-	Down-Detect	Down
gi|7672215	pre-mRNA splicing factor, putative	20	71621	3	0	2	0	2.2	10.01	0.05	-	-	-	-	-	-	-	-	-	Down-Detect	Down
gi|124804419	conserved Plasmodium protein, unknown function	20	330099	21	0	15	0	6.4	6.16	0.01	20	330099	29	0	21	0	9.3	6.16	0.01		
gi|33413784	normocyte binding protein 2b	20	382646	57	0	34	0	11.7	5.18	0.01	-	-	-	-	-	-	-	-	-	Down-Detect	Down
gi|23615500	reticulocyte binding protein 2 homologue a	20	370208	51	0	34	0	12.2	5.44	0.01	-	-	-	-	-	-	-	-	-	Down-Detect	Down
gi|124806068	uncharacterized protein PF3D7_1222000	19	65650	7	0	7	0	10.5	9.23	0.05	-	-	-	-	-	-	-	-	-	Down-Detect	Down
gi|124808735	nucleolar GTP-binding protein 2, putative	19	57136	6	0	4	0	8.4	9.72	0.06	19	57136	10	0	8	0	16.2	9.72	0.06		
gi|258597770	dynein-related AAA-type ATPase, putative	19	970399	117	0	73	0	11.7	8.07	0	20	970399	132	0	74	0	11.9	8.07	0		
gi|225632063	conserved Plasmodium protein, unknown function	19	346585	49	0	30	0	12	7.11	0.01	20	346585	46	0	31	0	12.6	7.11	0.01		
gi|124802000	hypothetical protein PF3D7_1004100	19	141831	34	0	11	0	11.5	8.61	0.02	20	141831	41	0	13	0	15.4	8.61	0.02		
gi|225685560	6-cysteine protein	19	114662	3	0	3	0	3.3	6.28	0.03	20	114662	5	0	5	0	6.3	6.28	0.03		
gi|23615182	conserved Plasmodium protein, unknown function	19	1111079	153	0	82	0	11.6	9.12	0	-	-	-	-	-	-	-	-	-	Down-Detect	Down
gi|23510631	asparagine-rich antigen Pfa35-2	19	363848	52	0	21	0	8.9	6.47	0.01	-	-	-	-	-	-	-	-	-	Down-Detect	Down
gi|124808373	conserved Plasmodium protein, unknown function	18	390446	71	0	30	0	10.4	9.23	0.01	-	-	-	-	-	-	-	-	-	Down-Detect	Down
gi|258597555	clustered-asparagine-rich protein	18	51501	4	0	4	0	8.1	8.71	0.07	-	-	-	-	-	-	-	-	-	Down-Detect	Down
gi|124806250	conserved Plasmodium protein, unknown function	18	140933	17	0	9	0	9.6	9.27	0.02	-	-	-	-	-	-	-	-	-	Down-Detect	Down
gi|3894094	mitogen-activated protein kinase 2	18	59173	7	0	4	0	9.8	8.63	0.06	-	-	-	-	-	-	-	-	-	Down-Detect	Down
gi|23504897	Plasmodium exported protein (PHISTb), unknown function	18	60150	15	0	9	0	18.3	4.93	0.06	-	-	-	-	-	-	-	-	-	Down-Detect	Down
gi|3764023	parasite-infected erythrocyte surface protein	18	153993	13	0	8	0	7.6	5.14	0.02	-	-	-	-	-	-	-	-	-	Down-Detect	Down
gi|124804142	leucine-rich repeat protein	18	93275	25	0	10	0	13.9	8.97	0.04	-	-	-	-	-	-	-	-	-	Down-Detect	Down
gi|225631849	conserved Plasmodium protein, unknown function	18	102515	14	0	7	0	7.8	8.83	0.03	-	-	-	-	-	-	-	-	-	Down-Detect	Down
gi|124809929	conserved Plasmodium protein, unknown function	18	88742	16	0	13	0	19.1	6.56	0.04	-	-	-	-	-	-	-	-	-	Down-Detect	Down
gi|23498873	ferrodoxin reductase-like protein	18	72675	14	0	8	0	17.6	8.91	0.05	15	72675	24	0	12	0	24.9	8.91	0.05		
gi|124801397	40S ribosomal protein S26	18	12501	2	0	2	0	15	10.98	0.29	-	-	-	-	-	-	-	-	-	Down-Detect	Down
gi|23498197	erythrocyte membrane protein 1, PfEMP1	18	263576	32	0	22	0	10.4	5.25	0.01	-	-	-	-	-	-	-	-	-	Down-Detect	Down
gi|23498196	erythrocyte membrane protein 1, PfEMP1	18	261684	31	0	22	0	10.3	5.28	0.01	-	-	-	-	-	-	-	-	-	Down-Detect	Down
gi|46361233	HECT-domain (ubiquitin-transferase), putative	18	1205255	209	0	113	0	13.2	8.19	0.01	17	1205255	182	0	91	0	11.3	8.19	0		
gi|23504939	LCCL domain-containing protein	18	178224	27	0	14	0	11.4	8.81	0.02	-	-	-	-	-	-	-	-	-	Down-Detect	Down
gi|225631940	conserved Plasmodium protein, unknown function	17	737235	102	0	48	0	10	9.13	0	-	-	-	-	-	-	-	-	-	Down-Detect	Down
gi|74930170	RecName: Full=Actin-2; AltName: Full=Actin II	17	42578	4	0	4	0	18.6	5.21	0.08	-	-	-	-	-	-	-	-	-	Down-Detect	Down
gi|124801175	tyrosine kinase-like protein, putative	17	148133	65	0	11	0	10	9.57	0.02	-	-	-	-	-	-	-	-	-	Down-Detect	Down
gi|124808338	conserved protein, unknown function	17	81048	9	0	4	0	6.9	8.6	0.04	-	-	-	-	-	-	-	-	-	Down-Detect	Down
gi|124804772	60S ribosomal protein L35ae, putative	17	16255	1	0	1	0	8.6	10.55	0.22	-	-	-	-	-	-	-	-	-	Down-Detect	Down
gi|124804153	conserved Plasmodium protein, unknown function	17	153603	33	0	8	0	7.3	6.77	0.02	15	153603	30	0	11	0	12.1	6.77	0.02		
gi|23498172	ag-1 blood stage membrane protein homologue	17	69060	9	0	6	0	9.8	5.47	0.05	-	-	-	-	-	-	-	-	-	Down-Detect	Down
gi|23615433	conserved Plasmodium protein, unknown function	17	319935	85	0	38	0	14.7	7.32	0.01	-	-	-	-	-	-	-	-	-	Down-Detect	Down
gi|124803597	transcription factor with AP2 domain(s)	17	206747	14	0	12	0	10.8	5.63	0.02	-	-	-	-	-	-	-	-	-	Down-Detect	Down
gi|23615614	conserved Plasmodium membrane protein, unknown function	17	460569	44	0	30	0	10.8	7.48	0.01	-	-	-	-	-	-	-	-	-	Down-Detect	Down
gi|4493884	inner membrane complex protein 1e, putative	16	61726	8	0	7	0	18.1	6.63	0.06	-	-	-	-	-	-	-	-	-	Down-Detect	Down
gi|23505002	conserved Plasmodium protein, unknown function	16	368118	39	0	23	0	10.9	5.27	0.01	-	-	-	-	-	-	-	-	-	Down-Detect	Down
gi|74862993	RecName: Full=Uncharacterized protein PFB0765w	16	166903	83	0	21	0	14.4	6.19	0.02	23	166903	82	0	21	0	17.1	6.19	0.04	2.00	Up
gi|23498752	conserved Plasmodium protein, unknown function	16	118270	4	0	2	0	3.8	8.1	0.03	-	-	-	-	-	-	-	-	-	Down-Detect	Down

gi|7670012	rifin	16	39495	10	0	6	0	27.3	9.15	0.09	-	-	-	-	-	-	-	-	-	Down-Detect	Down
gi|23499096	conserved Plasmodium protein, unknown function	15	170170	28	0	9	0	8.1	6.4	0.02	27	170170	30	0	14	0	12.1	6.4	0.02		
gi|124804913	tubulin--tyrosine ligase, putative	15	325121	72	0	30	0	13.9	9.25	0.01	-	-	-	-	-	-	-	-	-	Down-Detect	Down
gi|23615797	conserved Plasmodium protein, unknown function	15	78525	14	0	6	0	9.9	9.38	0.04	-	-	-	-	-	-	-	-	-	Down-Detect	Down
gi|124806691	erythrocyte membrane protein 1, PfEMP1	15	261440	34	0	28	0	12.8	5.15	0.01	-	-	-	-	-	-	-	-	-	Down-Detect	Down
gi|124801837	erythrocyte membrane protein 1, PfEMP1	15	252173	42	0	27	0	15.6	5.26	0.01	-	-	-	-	-	-	-	-	-	Down-Detect	Down
gi|124805350	erythrocyte membrane protein 1, PfEMP1	15	355019	60	0	35	0	13.3	7.25	0.01	-	-	-	-	-	-	-	-	-	Down-Detect	Down
gi|256032266	Chain A, Erythrocyte Membrane Protein 1 (pfemp1)	15	35553	22	0	9	0	31.1	8.3	0.1	-	-	-	-	-	-	-	-	-	Down-Detect	Down
gi|124808195	DNA repair helicase, putative	15	135460	41	0	16	0	12.8	8.96	0.03	18	135460	42	0	16	0	12.8	8.96	0.03		
gi|23615267	conserved Plasmodium protein, unknown function	13	34955	13	0	7	0	24.2	9.22	0.1	31	34955	16	1	5	1	16	9.22	0.1		
gi|6138833	Polyubiquitin	-	-	-	-	-	-	-	-	-	172	42803	19	6	9	4	21	7	0.49	Up-Detect	Up
gi|322812543	Chain A, Glucose-6-phosphate isomerase	-	-	-	-	-	-	-	-	-	102	69517	25	4	11	2	24.3	6.9	0.16	Up-Detect	Up
gi|7672213	eukaryotic translation initiation factor 3 subunit K, putative	-	-	-	-	-	-	-	-	-	79	28017	4	1	4	1	23	5.61	0.13	Up-Detect	Up
gi|124810483	proteasome subunit alpha type-1, putative	-	-	-	-	-	-	-	-	-	65	28819	4	1	4	1	24	5.51	0.12	Up-Detect	Up
gi|15375385	glutaredoxin 1	-	-	-	-	-	-	-	-	-	62	12410	7	3	4	3	31.5	7.68	1.17	Up-Detect	Up
gi|285803543	Chain A, M17 Leucyl Aminopeptidase	-	-	-	-	-	-	-	-	-	57	58604	18	2	7	1	23.3	6.38	0.06	Up-Detect	Up
gi|124803623	endoplasmic reticulum-resident calcium binding protein	-	-	-	-	-	-	-	-	-	55	39350	4	1	4	1	12.5	4.49	0.19	Up-Detect	Up
gi|8439487	hypothetical protein, partial	-	-	-	-	-	-	-	-	-	48	23455	4	2	2	1	4	5.87	0.15	Up-Detect	Up
gi|23615667	proteasome subunit alpha type-4, putative	-	-	-	-	-	-	-	-	-	47	27930	4	2	3	1	11.8	5.85	0.13	Up-Detect	Up
gi|23504501	rhoptry-associated protein 3	-	-	-	-	-	-	-	-	-	46	46974	1	1	1	1	4.3	8.66	0.08	Up-Detect	Up
gi|258597440	antigen 332, DBL-like protein	-	-	-	-	-	-	-	-	-	45	688870	84	1	20	1	4.2	3.86	0.01	Up-Detect	Up
gi|23504499	skeleton-binding protein 1	-	-	-	-	-	-	-	-	-	45	36277	2	1	1	1	7.4	4.33	0.1	Up-Detect	Up
gi|23505252	cytoadherence linked asexual protein 9	-	-	-	-	-	-	-	-	-	42	160313	24	2	11	1	8.5	8.88	0.02	Up-Detect	Up
gi|23615691	ubiquitin-conjugating enzyme, putative	-	-	-	-	-	-	-	-	-	40	22869	2	1	2	1	13.9	5.32	0.16	Up-Detect	Up
gi|23615551	60S ribosomal protein L18, putative	-	-	-	-	-	-	-	-	-	39	21733	13	1	5	1	27.7	10.62	0.16	Up-Detect	Up
gi|23505019	falstatin	-	-	-	-	-	-	-	-	-	39	46929	4	1	3	1	10.4	6.22	0.08	Up-Detect	Up
gi|23498992	surface-associated interspersed protein 8.2 (SURFIN 8.2)	-	-	-	-	-	-	-	-	-	38	248324	37	2	26	1	13.3	5.35	0.01	Up-Detect	Up
gi|124809878	transcription factor with AP2 domain(s)	-	-	-	-	-	-	-	-	-	36	161410	13	1	9	1	7.4	8.98	0.02	Up-Detect	Up
gi|124808162	conserved Plasmodium protein, unknown function	-	-	-	-	-	-	-	-	-	35	240981	43	1	24	1	13	9.51	0.01	Up-Detect	Up
gi|258597720	60S ribosomal protein L7-3, putative	-	-	-	-	-	-	-	-	-	34	32661	9	1	3	1	11.7	10.17	0.11	Up-Detect	Up
gi|23498727	small exported membrane protein 1	-	-	-	-	-	-	-	-	-	33	14186	2	1	1	1	7.3	9.61	0.26	Up-Detect	Up
gi|124801366	ATP synthase F1, alpha subunit	-	-	-	-	-	-	-	-	-	33	61731	16	1	7	1	16.9	8.72	0.06	Up-Detect	Up
gi|3649758	T-complex protein 1 subunit eta	-	-	-	-	-	-	-	-	-	33	59540	19	1	6	1	12.6	5.45	0.06	Up-Detect	Up
gi|124805983	clathrin heavy chain, putative	-	-	-	-	-	-	-	-	-	32	232803	38	1	13	1	7.8	6	0.01	Up-Detect	Up
gi|258597961	cysteine repeat modular protein 4	-	-	-	-	-	-	-	-	-	32	700473	99	2	41	1	9.8	8.43	0	Up-Detect	Up
gi|23505053	conserved Plasmodium protein, unknown function	-	-	-	-	-	-	-	-	-	32	85353	10	2	9	1	12.3	9.1	0.04	Up-Detect	Up
gi|296004752	erythrocyte membrane protein 1, PfEMP1	-	-	-	-	-	-	-	-	-	32	255615	49	1	28	1	14.5	5.41	0.01	Up-Detect	Up
gi|124806724	GAS8-like protein, putative	-	-	-	-	-	-	-	-	-	31	54772	4	1	4	1	8.9	7.98	0.06	Up-Detect	Up
gi|124802168	eukaryotic translation initiation factor 2 subunit beta, putative	-	-	-	-	-	-	-	-	-	31	25306	9	1	5	1	22.5	9.23	0.14	Up-Detect	Up
gi|124802833	haloacid dehalogenase-like hydrolase	-	-	-	-	-	-	-	-	-	31	32821	2	1	2	1	13.2	5.62	0.11	Up-Detect	Up
gi|6562734	replication protein A1, large subunit	-	-	-	-	-	-	-	-	-	30	134118	19	1	14	1	14.2	6.58	0.03	Up-Detect	Up
gi|258597947	conserved Plasmodium membrane protein, unknown function	-	-	-	-	-	-	-	-	-	30	64379	2	1	2	1	3	8.88	0.05	Up-Detect	Up
gi|258597726	conserved Plasmodium protein, unknown function	-	-	-	-	-	-	-	-	-	30	248348	51	1	18	1	10.2	9.01	0.01	Up-Detect	Up
gi|124806302	WD repeat-containing protein, putative	-	-	-	-	-	-	-	-	-	30	378674	54	1	26	1	9.9	9.02	0.02	Up-Detect	Up
gi|23504974	ATP-dependent protease ATPase subunit ClpY	-	-	-	-	-	-	-	-	-	30	106396	38	1	15	1	17.4	8.42	0.03	Up-Detect	Up
gi|23505108	inosine-5~-monophosphate dehydrogenase	-	-	-	-	-	-	-	-	-	30	56115	13	1	9	1	34.1	7.99	0.06	Up-Detect	Up
gi|124810280	conserved Plasmodium protein, unknown function	-	-	-	-	-	-	-	-	-	30	295648	21	1	16	1	8.1	5.19	0.01	Up-Detect	Up
gi|23615562	casein kinase II beta chain	-	-	-	-	-	-	-	-	-	30	45257	2	1	2	1	7.5	3.66	0.08	Up-Detect	Up
gi|74873273	RecName: Full=GRIP and coiled-coil domain-containing protein PFC0235w	-	-	-	-	-	-	-	-	-	30	135640	26	0	17	0	14	4.87	0.03	Up-Detect	Up
gi|225632280	conserved Plasmodium protein, unknown function	-	-	-	-	-	-	-	-	-	30	88381	21	0	9	0	12.4	9.76	0.04	Up-Detect	Up
gi|23504941	GTPase-activating protein, putative	-	-	-	-	-	-	-	-	-	30	50269	4	0	4	0	8.5	4.84	0.07	Up-Detect	Up
gi|225631662	conserved Plasmodium membrane protein, unknown function	-	-	-	-	-	-	-	-	-	29	354955	29	1	23	1	9	5.17	0.01	Up-Detect	Up
gi|225631719	conserved Plasmodium protein, unknown function	-	-	-	-	-	-	-	-	-	29	280186	41	1	27	1	12.5	5.04	0.01	Up-Detect	Up

gi|258549210	conserved Plasmodium protein, unknown function	-	-	-	-	-	-	-	-	-	29	30341	4	0	3	0	12.6	7.64	0.13	Up-Detect	Up
gi|23615215	U3 small nucleolar RNA-associated protein 6,putative	-	-	-	-	-	-	-	-	-	29	120650	13	0	6	0	5.1	5.51	0.03	Up-Detect	Up
gi|23615769	6-cysteine protein	-	-	-	-	-	-	-	-	-	29	92669	9	1	6	1	10.2	6.49	0.04	Up-Detect	Up
gi|15383897	conserved protein, unknown function	-	-	-	-	-	-	-	-	-	29	12885	8	1	1	1	12	10.19	0.28	Up-Detect	Up
gi|124802200	PRE-binding protein	-	-	-	-	-	-	-	-	-	28	131545	15	1	11	1	11.2	9.19	0.03	Up-Detect	Up
gi|124804380	26S protease regulatory subunit 6A, putative	-	-	-	-	-	-	-	-	-	27	49510	4	1	4	1	13.4	5.07	0.07	Up-Detect	Up
gi|23615467	60S ribosomal protein L23, putative	-	-	-	-	-	-	-	-	-	27	14983	7	0	4	0	37.4	9.9	0.24	Up-Detect	Up
gi|74862955	RecName: Full=Origin recognition complex subunit 1; Short=PfORC1	-	-	-	-	-	-	-	-	-	27	138653	11	0	8	0	8.7	9.52	0.03	Up-Detect	Up
gi|258597800	conserved Plasmodium protein, unknown function	-	-	-	-	-	-	-	-	-	26	152700	28	0	16	0	16	7.78	0.02	Up-Detect	Up
gi|258597165	antigen UB05	-	-	-	-	-	-	-	-	-	25	13703	3	0	3	0	20.2	9.44	0.27	Up-Detect	Up
gi|23505203	peptidyl-prolyl cis-trans isomerase	-	-	-	-	-	-	-	-	-	25	72507	10	0	7	0	15.8	9.11	0.05	Up-Detect	Up
gi|23504953	mitochondrial carrier protein, putative	-	-	-	-	-	-	-	-	-	24	141981	62	0	18	0	18.7	9.63	0.02	Up-Detect	Up
gi|23615559	aconitate hydratase	-	-	-	-	-	-	-	-	-	24	103313	9	0	4	0	3.7	7.73	0.03	Up-Detect	Up
gi|46362309	conserved Plasmodium protein, unknown function	-	-	-	-	-	-	-	-	-	24	324818	46	0	21	0	10	6.62	0.01	Up-Detect	Up
gi|124805657	U6 snRNA-associated Sm-like protein LSm7,putative	-	-	-	-	-	-	-	-	-	22	11988	3	0	3	0	38	5.8	0.31	Up-Detect	Up
gi|225631642	conserved Plasmodium protein, unknown function	-	-	-	-	-	-	-	-	-	22	821866	120	0	66	0	11.4	9.5	0	Up-Detect	Up
gi|124809457	conserved Plasmodium protein, unknown function	-	-	-	-	-	-	-	-	-	22	300791	33	0	28	0	13.7	9.23	0.01	Up-Detect	Up
gi|23615625	conserved Plasmodium protein, unknown function	-	-	-	-	-	-	-	-	-	22	405156	55	0	23	0	8.6	8.06	0.01	Up-Detect	Up
gi|258597814	conserved Plasmodium protein, unknown function	-	-	-	-	-	-	-	-	-	21	864485	122	0	55	0	8.4	8.74	0	Up-Detect	Up
gi|258597897	serine/threonine protein phosphatase UIS2,putative	-	-	-	-	-	-	-	-	-	21	170374	46	0	18	0	14.8	5.51	0.02	Up-Detect	Up
gi|23505262	Plasmodium exported protein (PHISTc), unknown function	-	-	-	-	-	-	-	-	-	21	45472	6	0	5	0	13.8	9.71	0.08	Up-Detect	Up
gi|23504667	RAP protein, putative	-	-	-	-	-	-	-	-	-	21	162718	7	0	6	0	7	7.96	0.02	Up-Detect	Up
gi|124804341	parasitophorous vacuolar protein 1	-	-	-	-	-	-	-	-	-	20	51919	13	0	5	0	19.5	4.97	0.07	Up-Detect	Up
gi|23615369	conserved Plasmodium membrane protein, unknown function	-	-	-	-	-	-	-	-	-	20	404731	84	0	40	0	13	8.76	0.01	Up-Detect	Up
gi|23498252	regulator of chromosome condensation, putative	-	-	-	-	-	-	-	-	-	20	236211	38	0	16	0	9.2	9.23	0.01	Up-Detect	Up
gi|23498938	DNA (cytosine-5)-methyltransferase	-	-	-	-	-	-	-	-	-	20	83731	6	0	5	0	9.6	8.74	0.04	Up-Detect	Up
gi|124804507	protein disulfide isomerase	-	-	-	-	-	-	-	-	-	19	49164	16	0	5	0	11.1	8.94	0.07	Up-Detect	Up
gi|46362277	conserved Plasmodium protein, unknown function	-	-	-	-	-	-	-	-	-	19	334204	60	0	32	0	14.4	8.49	0.01	Up-Detect	Up
gi|124806715	conserved Plasmodium protein, unknown function	-	-	-	-	-	-	-	-	-	18	63686	4	0	3	0	6.2	6.73	0.06	Up-Detect	Up
gi|23505265	Plasmodium exported protein, unknown function	-	-	-	-	-	-	-	-	-	18	31267	5	0	3	0	8.9	9.69	0.11	Up-Detect	Up
gi|23505270	erythrocyte membrane protein 1, PfEMP1	-	-	-	-	-	-	-	-	-	18	151890	21	0	14	0	13.2	5.66	0.02	Up-Detect	Up
gi|124807182	rifin	-	-	-	-	-	-	-	-	-	18	34492	23	0	4	0	12.7	8.89	0.1	Up-Detect	Up
gi|124805668	porphobilinogen deaminase	-	-	-	-	-	-	-	-	-	18	50315	103	0	7	0	23.9	9.54	0.07	Up-Detect	Up
gi|124802905	merozoite surface protein 6	-	-	-	-	-	-	-	-	-	17	42250	18	0	2	0	6.7	4.32	0.08	Up-Detect	Up
gi|225632049	conserved Plasmodium protein, unknown function	-	-	-	-	-	-	-	-	-	17	144043	16	0	9	0	8.1	9.66	0.02	Up-Detect	Up
gi|3758839	P-type ATPase, putative	-	-	-	-	-	-	-	-	-	17	217882	21	0	14	0	7.8	8.09	0.02	Up-Detect	Up
gi|124801012	serine repeat antigen 7	-	-	-	-	-	-	-	-	-	17	109569	12	0	7	0	9.9	5.57	0.03	Up-Detect	Up
gi|23498914	mitochondrial import inner membrane translocase subunit TIM14, putative	-	-	-	-	-	-	-	-	-	17	13044	5	0	3	0	22.6	10.09	0.28	Up-Detect	Up
gi|23615584	rhomboid protease ROM6, putative	-	-	-	-	-	-	-	-	-	17	67822	3	0	3	0	4	9.73	0.05	Up-Detect	Up
gi|124806291	RNA pseudouridylate synthase, putative	-	-	-	-	-	-	-	-	-	17	48335	7	0	7	0	19.3	9.57	0.07	Up-Detect	Up
gi|46362292	conserved Plasmodium protein, unknown function	-	-	-	-	-	-	-	-	-	17	123256	15	0	10	0	12.6	5.59	0.03	Up-Detect	Up
gi|23615696	serine/threonine protein kinase, putative	-	-	-	-	-	-	-	-	-	17	475422	65	0	47	0	13.6	8.99	0.01	Up-Detect	Up
gi|124806489	DNA polymerase epsilon subunit B, putative	-	-	-	-	-	-	-	-	-	17	73584	11	0	4	0	8.2	6.02	0.05	Up-Detect	Up
gi|225632011	heat shock protein 110, putative	-	-	-	-	-	-	-	-	-	16	108119	21	0	11	0	13.5	5.5	0.03	Up-Detect	Up
gi|46361213	oxidoreductase, short-chain dehydrogenase family, putative	-	-	-	-	-	-	-	-	-	16	44730	11	0	8	0	23.5	9.39	0.08	Up-Detect	Up
gi|23498967	transcription factor with AP2 domain(s)	-	-	-	-	-	-	-	-	-	16	155036	16	0	14	0	12.3	4.95	0.02	Up-Detect	Up
gi|23615716	CUGBP Elav-like family member 1	-	-	-	-	-	-	-	-	-	15	57079	5	0	3	0	6.1	5.43	0.06	Up-Detect	Up
gi|23504871	thioredoxin-like protein, putative	-	-	-	-	-	-	-	-	-	15	22041	3	0	2	0	9.8	9.16	0.16	Up-Detect	Up
gi|124802989	enhancer of rudimentary homolog, putative	-	-	-	-	-	-	-	-	-	15	12313	2	0	2	0	29.5	8.65	0.3	Up-Detect	Up
gi|23615391	60S ribosomal protein L23	-	-	-	-	-	-	-	-	-	15	22079	41	0	4	0	20.5	10.27	0.16	Up-Detect	Up
