# Supplementary material for: Proteomic analysis of Plasmodium falciparum response to isocryptolepine derivative
Source: PLoS One. 2019 Aug 8;14(8):e0220871. doi: 10.1371/journal.pone.0220871 (PMC6687117; doi:10.1371/journal.pone.0220871)
Supplement: S4 File — The experiment was performed in three biological replicates. Proteins with altered expression levels were selected based on different expression levels in at least two biological replicates. (PDF) [file pone.0220871.s004.pdf]

Supplement 4 file. Selection of differential proteins from three biological replicates

The experiment was performed in three biological replicates.

Protein alteration was selected by the different expression at least in two biological replicates.

									ICL-M			
									**Rep.1**	**Rep.2**	**Rep.3**	**Select**
**NCBI acc_no**	**All prot_desc**	**Mass**	**PI**	**Score**	***Coverage***	**UniProtKB**	**Biological Process**	**Localization**	**Up/Down**	**Up/Down**	**Up/Down**	**Up/Down**
gi|23499105	heat shock protein 70	73868	5.51	7020	53.8	Q8IB24_PLAF7	Unknown	Cytoplasm	-	-	-	-
gi|23505079	heat shock protein 70	72343	5.18	4848	51.4	Q8I2X4_PLAF7	Unknown	Endoplasmic Reticulum	-	-	-	-
gi|124810131	glyceraldehyde-3-phosphate dehydrogenase	36612	7.59	3876	76.6	Q8IKK7_PLAF7	Metabolism	Cytoplasm	-	-	-	-
gi|371941768	Hsp70-x	75006	5.59	2586	24.4	K7NTP5_PLAF7	Unknown	Unknown	-	-	-	-
gi|50400239	RecName: Full=Enolase; AltName: Full=2-phospho-D-glycerate hydro-lyase; AltName: Full=2-phosphoglycerate dehydratase	48647	6.21	2933	65	ENO_PLAF7	Metabolism	Cytoplasm	-	-	-	-
gi|23615698	elongation factor 1-alpha	48928	9.12	2796	59.6	Q8I0P6_PLAF7	Translation	Cytoplasm	-	-	-	-
gi|23498766	heat shock protein 90	86112	4.94	1977	40.4	Q8IC05_PLAF7	Protein fate	Unknown	-	-	-	-
gi|46361043	ornithine aminotransferase	46025	6.47	1642	40.3	OAT_PLAF7	Metabolism	Cytoplasm	-	-	-	-
gi|124803934	GTP-binding nuclear protein RAN/TC4	24860	7.72	1358	67.8	Q7KQK6_PLAF7	Transport	Cytoplasm	-	Up	-	-
gi|23615406	L-lactate dehydrogenase	34086	7.12	1413	48.1	Q76NM3_PLAF7	Metabolism	Unknown	-	-	-	-
gi|225632282	protein disulfide isomerase	55479	5.56	1965	39.5	C0H4Y6_PLAF7	Cell Rescue Defense	Endoplasmic Reticulum	-	-	-	-
gi|344189571	Chain A, Phosphoglycerate Kinase	46264	7.82	1460	70.5	PGK_PLAF7	Metabolism	Unknown	Up	-	-	-
gi|129926	RecName: Full=Phosphoglycerate kinase	45398	7.63	1460	64.7	PGK_PLAF7	Metabolism	Unknown	-	-	-	-
gi|74920225	RecName: Full=Fructose-bisphosphate aldolase	40080	8.33	1103	64	ALF_PLAF7	Metabolism	Cytoplasm	-	-	Up	-
gi|124803500	histone H2B	13117	10.27	1304	74.4	Q8IIV1_PLAF7	Nucleosome Assembly	Nucleus	Down	Up	-	-
gi|31505529	histone H4, partial	11448	11.23	1401	67	Q8IIV2_PLAF7	Nucleosome Assembly	Nucleus	-	Up	-	-
gi|124809712	elongation factor 2	93462	6.36	1337	31.3	Q8IKW5_PLAF7	Unknown	Unknown	-	-	-	-
gi|23498286	40S ribosomal protein S19	19710	10.17	876	62.9	Q8IFP2_PLAF7	Translation	Cytoplasm	Down	-	-	-
gi|23505200	merozoite surface protein 1	195605	6.11	910	20.6	Q8I0U8_PLAF7	Pathogenesis	Membrane	-	-	-	-
gi|225632253	14-3-3 protein	30174	4.86	622	52.7	C0H4V6_PLAF7	Unknown	Cytoplasm	-	-	-	-
gi|124806075	endoplasmin, putative	94959	5.28	1054	37.1	Q8I0V4_PLAF7	Protein fate	Endoplasmic Reticulum	-	-	-	-
gi|46361058	histone H3	15437	11.14	691	51.5	C6KSV0_PLAF7	Nucleosome Assembly	Nucleus	-	-	-	-
gi|124804377	60S ribosomal protein P0	34945	6.28	514	50.9	Q8II61_PLAF7	Translation	Cytoplasm	-	-	-	-
gi|124802054	DNA/RNA-binding protein Alba 3	11969	9.3	415	92.5	Q8IJX8_PLAF7	Unknown	Nucleus	-	-	Up	-
gi|23498886	40S ribosomal protein S5, putative	21849	9.67	505	45.1	Q8IBN5_PLAF7	Translation	Cytoplasm	-	Up	Down	-
gi|303324901	Chain A, Heat Shock Protein 86	25195	4.85	470	32.7	Q8IC05_PLAF7	Protein fate	Unknown	Down	Up	-	-
gi|23615568	phosphoethanolamine N-methyltransferase	31024	5.43	637	50.4	Q8IDQ9_PLAF7	Metabolism	Golgi Apparatus	-	-	-	-
gi|23499152	DNA/RNA-binding protein Alba 1	27242	10.58	409	42.3	Q8IAX8_PLAF7	Unknown	Cytoplasm	-	-	Down	-
gi|4493980	peptidyl-prolyl cis-trans isomerase	18940	8.29	444	50.3	Q76NN7_PLAF7	Protein fate	Cytoplasm	-	-	-	-
gi|225631678	60S ribosomal protein L12, putative	18101	9.54	373	58.8	Q8I3T8_PLAF7	Translation	Cytoplasm	-	-	-	-
gi|23504519	actin-depolymerizing factor 1	13732	7.66	649	59	CADF1_PLAF7	Cytoskeleton	Cytoplasm	Up	-	-	-
gi|4494010	histone H2A variant, putative	16443	10.63	486	32.3	O97320_PLAF7	Nucleosome Assembly	Nucleus	-	-	Down	-
gi|225631753	cell division cycle protein 48 homologue,putative	92329	4.95	420	28.4	C6KT34_PLAF7	Cell Cycle	Unknown	-	-	Down	-
gi|124803860	peptidyl-prolyl cis-trans isomerase	21717	7.1	343	69.2	Q8IIK8_PLAF7	Protein fate	Unknown	-	-	-	-
gi|124802119	adenylate kinase	27594	8.97	351	46.3	Q8IJV6_PLAF7	Metabolism	Cytoplasm	-	-	-	-
gi|46361129	histone H2A	14114	10.29	397	48.5	C6KT18_PLAF7	Nucleosome Assembly	Nucleus	-	-	-	-
gi|23499115	high mobility group protein B2	11527	9.97	290	42.4	Q8IB14_PLAF7	Transcription	Nucleus	-	-	-	-
gi|75009812	RecName: Full=Triosephosphate isomerase; Short=TIM; AltName: Full=Triose-phosphate isomerase	27917	6.01	409	36.3	TPIS_PLAF7	Metabolism	Cytoplasm	-	-	-	-
gi|23505159	conserved Plasmodium protein, unknown function	24683	5.49	298	47.9	Q8I2Q0_PLAF7	Unknown	Unknown	-	-	-	-
gi|258597456	nucleosome assembly protein	40463	4.76	416	17	Q8I608_PLAF7	Nucleosome Assembly	Cytoplasm	-	Down	-	-
gi|23504687	40S ribosomal protein S9, putative	22109	10.45	237	45.5	Q8I3R0_PLAF7	Translation	Cytoplasm	Down	Up	-	-
gi|23504938	alpha tubulin 1	50264	4.93	234	23.8	Q6ZLZ9_PLAF7	Cytoskeleton	Cytoplasm	Down	Down	Down	**Down**
gi|23504556	60S ribosomal protein L4	46183	10.5	268	34.3	Q8I431_PLAF7	Translation	Cytoplasm	-	Down	Down	**Down**
gi|124810348	exported protein 2	33391	5.1	229	24.4	Q8IKC8_PLAF7	Unknown	Membrane	-	Down	-	-
gi|124808771	60S ribosomal protein L5, putative	33977	9.78	223	21.1	Q8ILL3_PLAF7	Translation	Cytoplasm	-	-	Down	-
gi|74876423	RecName: Full=Tubulin beta chain; AltName: Full=Beta-tubulin	49719	4.73	389	44.3	TBB_PLAF7	Cytoskeleton	Cytoplasm	-	-	-	-
gi|11127605	heat shock protein hsp70 homologue Pfhsp70-3	71546	5.9	410	33.2	Q9GUX1_PLAFA	Protein fate	Unknown	Down	-	-	-
gi|124805478	eukaryotic translation initiation factor 5A	17620	5.42	222	66.5	Q8I603_PLAF7	Translation	Unknown	-	Up	-	-
gi|225631960	40S ribosomal protein S19	16753	10.27	351	38.6	C0H5C2_PLAF7	Translation	Cytoplasm	-	-	Down	-
gi|124808810	60S ribosomal protein L21	18783	10.04	477	37.3	Q8ILK3_PLAF7	Translation	Cytoplasm	-	-	-	-
gi|23504648	40S ribosomal protein S11	16067	10.49	236	58.3	Q8I3U6_PLAF7	Translation	Cytoplasm	-	Up	-	-
gi|23615723	40S ribosomal protein S15	17240	10.38	195	59.6	Q8IDB0_PLAF7	Translation	Cytoplasm	-	Up	-	-
gi|124804238	40S ribosomal protein S18, putative	17880	10.46	261	49.4	Q8IIA2_PLAF7	Translation	Cytoplasm	-	-	-	-
gi|258596854	stevor	34204	8.76	180	15.4	O96118_PLAF7	Cell Surface	Membrane	-	-	-	-
gi|23615606	DNA/RNA-binding protein Alba 4	42133	7.14	174	25.8	Q8IDM3_PLAF7	Unknown	Cytoplasm	-	-	-	-
gi|23504494	mature parasite-infected erythrocyte surface antigen	168186	4.76	275	17.6	Q8I492_PLAF7	Unknown	Membrane	-	-	-	-
gi|23504618	purine nucleoside phosphorylase	26841	6.07	261	42.9	Q8I3X4_PLAF7	Metabolism	Unknown	Down	Up	-	-
gi|282403624	Chain A, Uridine Phosphorylase, Putative	30453	5.83	205	36.2	Q8I3X4_PLAF7	Metabolism	Unknown	-	-	Down	-
gi|225631740	60S ribosomal protein L19	21566	11.32	168	14.3	C6KSY6_PLAF7	Translation	Cytoplasm	-	Down	Down	**Down**
gi|46361130	histone H3 variant, putative	15432	11.15	236	47.1	C6KT19_PLAF7	Nucleosome Assembly	Nucleus	-	-	-	-
gi|124805752	glutathione peroxidase-like thioredoxin peroxidase	23937	8.99	305	35.6	Q8I5T2_PLAF7	Protein fate	Apicoplast	Up	-	-	-
gi|124810293	eukaryotic initiation factor 4A	45281	5.48	409	36.2	Q8IKF0_PLAF7	Translation	Cytoplasm	-	Up	Down	-
gi|7768287	formate-nitrite transporter	34436	8.74	194	19.7	O77389_PLAF7	Transport	Membrane	-	-	Up	-
gi|124801997	60S ribosomal protein L13, putative	23739	10.19	207	37.1	Q8IJZ7_PLAF7	Translation	Cytoplasm	-	-	Down	-
gi|124808442	60S ribosomal protein L10, putative	25200	9.97	320	40.6	Q8ILV2_PLAF7	Translation	Cytoplasm	-	-	Down	-
gi|124800689	knob-associated histidine-rich protein	71259	9.17	309	15	Q9TY99_PLAF7	Unknown	Cytoplasm	-	-	-	-
gi|124804166	splicing factor, putative	16002	5.1	365	37.9	Q8IIC4_PLAF7	Transcription	Nucleus	-	-	-	-
gi|124809606	40S ribosomal protein S5	29939	10.02	270	42.3	Q8IL02_PLAF7	Translation	Cytoplasm	-	-	-	-
gi|23504496	Plasmodium exported protein, unknown function	30696	9.47	147	13.1	Q8I490_PLAF7	Unknown	Membrane	-	Down	-	-
gi|124803848	cysteine proteinase falcipain 2b	55768	8.14	246	26.6	Q8I6U5_PLAF7	Metabolism	Food Vacuole	-	Up	-	-
gi|124803863	cysteine proteinase falcipain 2a	55892	7.12	246	22.7	Q8I6U4_PLAF7	Metabolism	Food Vacuole	-	-	-	-
gi|23615388	60S ribosomal protein L6, putative	21588	9.88	142	37.4	Q8IE85_PLAF7	Translation	Cytoplasm	-	Down	-	-
gi|237640532	Chain A, HAP protein	37376	4.97	141	32.2	Q8IM15_PLAF7	Metabolism	Food Vacuole	Down	Down	Down	**Down**
gi|23504595	histamine-releasing factor	19967	4.58	210	23.4	TCTP_PLAF7	Cell Cycle	Cytoplasm	Up	Up	-	**Up**
gi|46361220	pyruvate kinase	55625	7.5	405	44.8	C6KTA4_PLAF7	Metabolism	Cytoplasm	Down	Up	Up	**Up**
gi|8247298	hypothetical protein, partial	4755	9.23	159	60	Q9NFH5_PLAFA	Unknown	Unknown	Down	-	Down	**Down**
gi|258549116	serine esterase, putative	216941	8.82	134	18.7	C6S3F2_PLAF7	Metabolism	Unknown	-	-	-	-
gi|46361040	proteasome subunit alpha type-2, putative	26512	5.4	185	14.9	C6KST3_PLAF7	Protein fate	Cytoplasm	-	-	-	-

gi|75016040	RecName: Full=Acidic leucine-rich nuclear phosphoprotein 32-related protein; AltName: Full=ANP32/acidic nuclear phosphoprotein-like protein	32986	4.27	177	24.2	AN32_PLAF7	Nucleosome Assembly	Nucleus	-	-	-	**-**
gi|124803852	cysteine proteinase falcipain 3	56630	6.59	131	26	Q8IIL0_PLAF7	Metabolism	Food Vacuole	Up	-	-	**-**
gi|268612503	Chain A, Phosphoglycerate mutase	29716	8.26	247	41.9	Q8IIG6_PLAF7	Metabolism	Cytoplasm	-	-	-	**-**
gi|124804024	phosphoglycerate mutase, putative	28752	8.3	247	57.6	Q8IIG6_PLAF7	Metabolism	Cytoplasm	-	-	-	**-**
gi|93278503	Chain A, Falcipain 2	27140	4.98	124	32	Q9N6S8_PLAFA	Unknown	Membrane	Down	-	-	**-**
gi|23499154	60S ribosomal protein L13-2, putative	25425	10.78	123	30.2	Q8IAX6_PLAF7	Translation	Cytoplasm	Down	Down	Down	**Down**
gi|124809152	mitochondrial acidic protein MAM33, putative	28854	4.89	172	29.4	Q8ILB6_PLAF7	Unknown	Mitochondria	-	-	-	**-**
gi|124808201	40S ribosomal protein S8e, putative	25035	9.98	262	28	Q8IM10_PLAF7	Translation	Cytoplasm	-	-	Down	**-**
gi|23504955	high molecular weight rhoptry protein 3	104789	6.25	150	21.4	Q8I395_PLAF7	Invasion	Rhoptry	Down	Up	Down	**Down**
gi|23615526	60S ribosomal protein L6-2, putative	25516	10.1	222	26.7	Q8IDV1_PLAF7	Translation	Cytoplasm	-	Down	Down	**Down**
gi|23499261	1-cys peroxiredoxin	25148	6.31	155	47.3	Q8IAM2_PLAF7	Cell Rescue Defense	Cytoplasm	Down	Down	Down	**Down**
gi|23615644	60S ribosomal protein L17, putative	23400	10.89	109	36.5	Q8IDI5_PLAF7	Translation	Cytoplasm	Down	-	-	**-**
gi|4493906	40S ribosomal protein S12, putative	15387	4.9	192	60.3	RS12_PLAF7	Translation	Cytoplasm	-	-	-	**-**
gi|46361162	pyridoxine biosynthesis protein PDX1	32992	6.76	314	55.1	PDX1_PLAF7	Metabolism	Cytoplasm	Up	-	Down	**-**
gi|124802718	adenosine deaminase	42438	5.41	231	41.7	Q8IJA9_PLAF7	Metabolism	Cytoplasm	-	Down	Up	**-**
gi|124809201	glucose-6-phosphate isomerase	67325	6.78	172	23.1	Q8ILA4_PLAF7	Metabolism	Unknown	-	-	Down	**-**
gi|124806612	conserved protein, unknown function	23644	9.64	139	25.2	Q8I546_PLAF7	Unknown	Unknown	Down	-	-	**-**
gi|23504725	karyopherin beta	127272	4.8	141	14.1	Q8I3M5_PLAF7	Transport	Cytoplasm	Down	Up	-	**-**
gi|258597720	60S ribosomal protein L7-3, putative	32661	10.17	97	20.5	Q8ILL2_PLAF7	Translation	Cytoplasm	-	-	Up	**-**
gi|23499195	acyl-CoA binding protein, putative	10768	7.68	193	62.2	Q8IAT5_PLAF7	Unknown	Unknown	-	-	-	**-**
gi|23505219	profilin, putative	19005	4.22	98	18.7	PROF_PLAF7	Cytoskeleton	Cytoplasm	-	Down	-	**-**
gi|23615603	nucleoside transporter 1	47600	8.36	135	12.6	Q8IDM6_PLAF7	Transport	Membrane	-	Up	Down	**-**
gi|23498814	histone H2B variant	13755	10.8	209	74.8	Q8IBV7_PLAF7	Nucleosome Assembly	Nucleus	-	Up	-	**-**
gi|18076407	early transcribed membrane protein 14.1	11420	9.63	93	20.6	Q7KQM5_PLAF7	Unknown	Membrane	-	-	-	**-**
gi|124809402	60S ribosomal protein L1, putative	24790	9.85	127	43.8	Q8IL58_PLAF7	Translation	Cytoplasm	-	Up	-	**-**
gi|23615172	40S ribosomal protein S7, putative	22467	9.81	266	41.8	Q8IET7_PLAF7	Translation	Cytoplasm	Down	-	Down	**Down**
gi|124802670	60S ribosomal protein L3	44193	10.21	89	29.3	Q8IJC6_PLAF7	Translation	Cytoplasm	Down	Down	Down	**Down**
gi|23615551	60S ribosomal protein L18, putative	21733	10.62	88	45.1	Q8IDS6_PLAF7	Translation	Cytoplasm	Down	Up	Up	**Up**
gi|258597310	60S ribosomal protein L35, putative	14739	10.79	87	32.3	Q8IIB4_PLAF7	Translation	Cytoplasm	-	-	-	**-**
gi|23615654	thioredoxin-related protein, putative	23972	9.44	134	26.9	Q8IDH5_PLAF7	Cell Rescue Defense	Membrane	Down	Up	Down	**Down**
gi|124802073	single-strand telomeric DNA-binding protein GBP2, putative	29512	9.2	136	26.4	Q8IJX3_PLAF7	Unknown	Unknown	-	-	-	**-**
gi|23615715	HVA22-like protein, putative	18410	9.34	91	37.3	Q8IDB8_PLAF7	Unknown	Membrane	-	-	-	**-**
gi|258597201	40S ribosomal protein S4, putative	29753	10.09	193	41.4	Q8IIU8_PLAF7	Translation	Cytoplasm	-	-	-	**-**
gi|124810210	40S ribosomal protein S3	24652	10.2	144	33.5	Q8IKH8_PLAF7	Translation	Nucleus	-	-	Down	**-**
gi|23498182	V-type proton ATPase subunit B	55753	5.46	106	32.4	Q6ZMA8_PLAF7	Metabolism	Cytoplasm	-	-	Up	**-**
gi|23498728	Plasmodium exported protein, unknown function	27656	8.55	95	28.9	Q8IC42_PLAF7	Unknown	Cytoplasm	-	-	Down	**-**
gi|124810024	conserved Plasmodium protein, unknown function	40043	9.6	83	15	Q8IKN7_PLAF7	Unknown	Unknown	-	Down	-	**-**
gi|7672213	eukaryotic translation initiation factor 3 subunit K, putative	28017	5.61	92	31.1	Q9NFE6_PLAF7	Translation	Cytoplasm	Up	Down	Up	**Up**
gi|23498233	small GTP-binding protein sar1	22006	6.75	72	31.3	Q8I1S0_PLAF7	Transport	Endoplasmic Reticulum	Down	-	Down	**Down**
gi|46576603	RecName: Full=Probable cathepsin C; Flags: Precursor	80361	5.82	107	23.1	CATC_PLAF7	Metabolism	Food Vacuole	-	-	-	**-**
gi|23498770	heat shock protein 110	99902	5.54	297	24.1	Q8IC01_PLAF7	Unknown	Cytoplasm	-	Up	-	**-**
gi|23498142	Plasmodium exported protein (PHISTa), unknown function	49713	9.51	126	15.2	Q8I206_PLAF7	Unknown	Membrane	-	-	-	**-**
gi|23505019	falstatin	46929	6.22	66	16.7	Q8I333_PLAF7	Invasion	Cytoplasm	-	Down	Up	**-**
gi|124802973	ADP/ATP transporter on adenylate translocase	33705	9.68	66	26.2	Q8IJ34_PLAF7	Transport	Membrane	-	-	Down	**-**
gi|124810483	proteasome subunit alpha type-1, putative	28819	5.51	192	24	Q8IK90_PLAF7	Protein fate	Cytoplasm	-	Up	Up	**Up**
gi|23615786	ubiquitin-60S ribosomal protein L40	14608	9.91	172	52.3	Q8ID50_PLAF7	Translation	Cytoplasm	-	Down	-	**-**
gi|46361167	nascent polypeptide-associated complex subunit alpha, putative	20610	4.77	95	31.5	C6KT55_PLAF7	Translation	Cytoplasm	-	Down	-	**-**
gi|124804546	conserved Plasmodium protein, unknown function	28478	8.8	71	22	Q8II11_PLAF7	Unknown	Unknown	-	-	-	**-**
gi|23504696	S-adenosyl-L-homocysteine hydrolase	53804	5.64	81	30.5	SAHH_PLAF7	Metabolism	Cytoplasm	-	Down	-	**-**
gi|258597663	nuclear transport factor 2, putative	14428	5.53	62	48	Q8ILX1_PLAF7	Transport	Cytoplasm	Down	-	-	**-**
gi|23498140	Plasmodium exported protein (PHISTb), unknown function	60233	8.76	142	22.3	Q8I207_PLAF7	Unknown	Membrane	Up	-	Down	**-**
gi|3122763	RecName: Full=60S acidic ribosomal protein P2	11941	4.49	115	88.4	RLA2_PLAF7	Translation	Cytoplasm	Up	-	-	**-**
gi|23504501	rhoptry-associated protein 3	46974	8.66	60	17	Q8I485_PLAF7	Unknown	Rhoptry	Down	-	Up	**-**
gi|124802223	hypoxanthine-guanine phosphoribosyltransferase	26346	7.59	73	30.3	Q8IJS1_PLAF7	Metabolism	Cytoplasm	-	-	-	**-**
gi|23615498	merozoite surface protein 7	41251	4.74	146	18.5	Q8IDX8_PLAF7	Invasion	Membrane	-	-	Up	**-**
gi|23498881	conserved Plasmodium protein, unknown function	29231	8.76	80	19.3	Q8IBP0_PLAF7	Unknown	Unknown	-	-	Down	**-**
gi|124801947	RNA-binding protein, putative	30033	10.07	55	35.1	Q8IK12_PLAF7	Unknown	Unknown	-	Down	Down	**Down**
gi|124809822	6-phosphogluconate dehydrogenase,decarboxylating, putative	52960	6.58	55	21.2	Q8IKT2_PLAF7	Metabolism	Unknown	-	-	-	**-**
gi|124804373	60S ribosomal protein L38	10307	10.71	100	58.6	Q8II62_PLAF7	Translation	Cytoplasm	Up	Up	Up	**Up**
gi|23615340	conserved Plasmodium protein, unknown function	88519	9.07	51	16.6	Q8IEC9_PLAF7	Unknown	Unknown	-	Down	-	**-**
gi|258597482	10 kDa chaperonin	11143	5.47	100	43.7	Q8I5Q3_PLAF7	Protein fate	Mitochondria	Up	-	-	**-**
gi|23505090	nucleosome assembly protein	31807	4.17	131	24.2	Q8I2W3_PLAF7	Nucleosome Assembly	Cytoplasm	-	Up	Up	**Up**
gi|124803892	folate transporter 2	51321	8.63	49	12.3	Q8IIK1_PLAF7	Unknown	Membrane	-	Up	Down	**-**
gi|4494003	40S ribosomal protein S3A, putative	30028	9.8	55	33.2	RS3A_PLAF7	Translation	Cytoplasm	Down	Down	-	**Down**
gi|23504974	ATP-dependent protease ATPase subunit ClpY	106396	8.42	48	17.4	Q8I377_PLAF7	Unknown	Cytoplasm	-	Up	Up	**Up**
gi|46361227	protein DJ-1	20280	6.95	182	36.5	C6KTB1_PLAF7	Metabolism	Cytoplasm	-	Down	-	**-**
gi|23615478	20 kDa chaperonin	29045	7.63	116	28.7	Q8IDZ8_PLAF7	Protein fate	Apicoplast	-	-	-	**-**
gi|23504603	inositol-3-phosphate synthase	69069	7.11	114	27.6	Q8I3Y8_PLAF7	Metabolism	Cytoplasm	-	-	-	**-**
gi|23499218	ras-related protein Rab-18	23148	8.11	63	17.9	Q7K6B0_PLAF7	Transport	Golgi Apparatus	-	Up	Down	**-**
gi|124806534	serine hydroxymethyltransferase	49749	8.29	103	31	Q8I566_PLAF7	Metabolism	Cytoplasm	Up	Up	-	**Up**
gi|651207688	Chain A, Serine Hydroxymethyltransferase	54007	7.21	103	33.1	Q8I566_PLAF7	Metabolism	Cytoplasm	Up	-	-	**-**
gi|23504681	40S ribosomal protein S24	15382	10.75	54	21.1	Q8I3R6_PLAF7	Translation	Cytoplasm	-	Up	-	**-**
gi|8052274	elongation factor 1 (EF-1), putative	17695	4.5	63	16.7	O97319_PLAF7	Translation	Cytoplasm	-	Up	-	**-**
gi|124803623	endoplasmic reticulum-resident calcium binding protein	39350	4.49	84	12.5	Q8IIR7_PLAF7	Transport	Endoplasmic Reticulum	Down	-	Up	**-**
gi|23504857	Hsc70-interacting protein	51092	4.67	45	14.6	Q8I3J0_PLAF7	Unknown	Unknown	Down	Down	Down	**Down**
gi|124806378	ras-related protein Rab-2	24408	6.33	45	46	Q8I5A9_PLAF7	Transport	Membrane	Down	-	-	**-**
gi|23498727	small exported membrane protein 1	14186	9.61	59	43.1	Q8IC43_PLAF7	Unknown	Cytoplasm	-	-	Up	**-**
gi|124806302	WD repeat-containing protein, putative	378674	9.02	45	12.8	Q8I5D0_PLAF7	Nucleosome Assembly	Nucleus	-	Up	Up	**Up**
gi|23615189	small heat shock protein, putative	25179	6.02	73	25.1	Q8IES0_PLAF7	Protein fate	Unknown	-	-	-	**-**

gi|23498791	AAA family ATPase, CDC48 subfamily	141991	9.07	47	17.9	CDAT_PLAF7	Cell Cycle	Apicoplast	-	-	Down	-
gi|23615236	60S ribosomal protein L24, putative	19232	10.43	43	23.5	Q8IEM3_PLAF7	Translation	Cytoplasm	Down	-	-	-
gi|3758867	proteasome subunit alpha type-3, putative	29270	6.38	48	35.7	O77396_PLAF7	Protein fate	Nucleus	-	-	-	-
gi|23498939	proteasome subunit alpha type-5, putative	28370	4.96	147	38.3	Q8IBI3_PLAF7	Protein fate	Cytoplasm	Up	Down	-	-
gi|124806724	GAS8-like protein, putative	54772	7.98	42	8.9	Q8I505_PLAF7	Cytoskeleton	Golgi Apparatus	Down	Up	Up	**Up**
gi|124802168	eukaryotic translation initiation factor 2 subunit beta, putative	25306	9.23	42	43.2	Q8IJT9_PLAF7	Translation	Cytoplasm	Down	Up	Up	**Up**
gi|124804998	rifin	38701	8.96	62	28.1	Q8IHM2_PLAF7	Cell Surface	Membrane	-	-	-	-
gi|23504672	WD repeat-containing protein 26, putative	150608	9.24	52	18.2	Q8I3S4_PLAF7	Unknown	Unknown	-	-	-	-
gi|6851056	spermidine synthase	36573	6.97	139	30.5	Q9NFS5_PLAFA	Metabolism	Unknown	-	-	Down	-
gi|225632259	U6 snRNA-associated Sm-like protein LSm3,putative	10694	5.16	115	61.5	C0H4W2_PLAF7	Transcription	Nucleus	-	-	-	-
gi|258597872	signal peptide peptidase	47547	8.95	42	13.3	Q8IKQ9_PLAF7	Protein Signaling	Cytoplasm	Down	Up	-	-
gi|124808815	basic transcription factor 3b, putative	19381	9.04	55	29.2	Q8ILK2_PLAF7	Transcription	Cytoplasm	-	-	Down	-
gi|7799189	thioredoxin	11709	4.67	201	60.6	A0A144A4E0_PLAF7	Cell Rescue Defense	Cytoplasm	Down	-	-	-
gi|258597884	60S ribosomal protein L27	16735	10.23	51	40.4	Q8IKM5_PLAF7	Translation	Cytoplasm	Down	-	-	-
gi|124802189	proteasome subunit beta type-5	30577	5.18	46	16.6	Q8IJT1_PLAF7	Protein fate	Cytoplasm	-	-	Up	-
gi|75016029	RecName: Full=STI1-like protein	66015	6.63	50	15.8	STI1L_PLAF7	Unknown	Cytoplasm	Up	-	Down	-
gi|124807078	rhoptry neck protein 3	262988	9.23	38	15.6	Q8I4R5_PLAF7	Invasion	Rhoptry	-	-	Down	-
gi|23615363	conserved Plasmodium protein, unknown function	170566	8.86	72	14.8	Q8IEA7_PLAF7	Unknown	Unknown	-	-	-	-
gi|23615558	40S ribosomal protein S6	35363	10.44	37	17.3	Q8IDR9_PLAF7	Translation	Cytoplasm	Down	-	-	-
gi|124809308	thioredoxin peroxidase 1	21793	6.65	90	31.3	Q8IL80_PLAF7	Cell Rescue Defense	Cytoplasm	-	Up	-	-
gi|23505054	6-phosphofructokinase	159351	6.32	90	19.2	Q8I2Z8_PLAF7	Metabolism	Cytoplasm	-	Down	-	-
gi|4493898	ubiquitin-conjugating enzyme E2, putative	16130	4.88	36	9.9	O97241_PLAF7	Protein fate	Cytoplasm	Down	-	-	-
gi|23615263	M1-family alanyl aminopeptidase	125983	7.3	66	15.9	Q8IEK1_PLAF7	Metabolism	Cytoplasm	-	-	Up	-
gi|59798920	RecName: Full=Serine-repeat antigen protein; AltName: Full=111 kDa antigen; AltName: Full=p126; Flags: Precursor	111698	5.26	46	18.8	SERA_PLAF7	Cell Surface	Lysosome	-	-	-	-
gi|74930131	RecName: Full=40S ribosomal protein SA	29837	5.91	46	25.5	RSSA_PLAF7	Translation	Cytoplasm	-	-	Up	-
gi|225632158	conserved Plasmodium protein, unknown function	24366	5.2	35	10.5	C0H4M7_PLAF7	Unknown	Membrane	-	-	-	-
gi|258596875	26S proteasome regulatory subunit RPN1,putative	108289	5.95	87	26	O96153_PLAF7	Protein fate	Nucleus	-	Up	-	-
gi|23499155	40S ribosomal protein S16, putative	16275	10.25	55	39.6	Q8IAX5_PLAF7	Translation	Cytoplasm	-	Up	-	-
gi|23615559	aconitate hydratase	103313	7.73	66	12.3	Q8IDR8_PLAF7	Translation	Cytoplasm	-	-	Up	-
gi|124806145	polyadenylate-binding protein, putative	97169	8.96	85	15.7	Q8I5H4_PLAF7	Transcription	Cytoplasm	Up	Down	-	-
gi|23504716	multidrug resistance protein 1	162150	8.94	118	18.7	Q7K6A5_PLAF7	Response to drug	Membrane	-	-	Up	-
gi|258597955	conserved Plasmodium membrane protein, unknown function	577659	8.86	44	11.7	Q8IK96_PLAF7	Unknown	Unknown	-	-	-	-
gi|23615265	malonyl CoA-acyl carrier protein transacylase precursor	46275	8.71	34	10.7	Q8I6Z9_PLAF7	Metabolism	Apicoplast	Down	-	-	-
gi|75009813	RecName: Full=Plasmepsin-1; AltName: Full=Aspartic hemoglobinase I; AltName: Full=PfAPG; Flags: Precursor	51428	6.72	65	15.7	PLM1_PLAF7	Metabolism	Food Vacuole	-	-	Down	-
gi|124809547	histidine--tRNA ligase, putative	133583	7.91	34	21.7	Q8IL22_PLAF7	Translation	Mitochondria	Down	-	-	-
gi|23615660	Plasmodium exported protein, unknown function	32763	9.23	59	27	Q8IDG9_PLAF7	Unknown	Membrane	-	-	Down	-
gi|23498992	surface-associated interspersed protein 8.2 (SURFIN 8.2)	248324	5.35	38	17.7	Q8IBD4_PLAF7	Unknown	Membrane	Up	Down	Up	**Up**
gi|225632239	conserved Plasmodium protein, unknown function	76808	5.34	69	23.1	C0H4U5_PLAF7	Unknown	Unknown	-	-	-	-
gi|23615667	proteasome subunit alpha type-4, putative	27930	5.85	47	20.3	Q8IDG3_PLAF7	Protein fate	Cytoplasm	-	Up	Up	**Up**
gi|225631627	conserved Plasmodium protein, unknown function	207703	9.22	34	9.8	C0H493_PLAF7	Unknown	Unknown	-	-	-	-
gi|124808276	rhoptry-associated protein 1	89996	6.67	148	15.3	Q8ILZ1_PLAF7	Unknown	Rhoptry	Down	-	Up	-
gi|23476993	Plasmodium exported protein (hyp8), unknown function	28216	9.02	35	16.9	Q8I2D9_PLAF7	Unknown	Cytoplasm	-	-	Down	-
gi|23504954	dynein heavy chain, putative	720134	6.18	32	10.6	Q8I396_PLAF7	Cytoskeleton	Cytoplasm	Down	Down	-	**Down**
gi|225632293	Plasmodium exported protein, unknown function	36390	5.74	35	22.2	C0H4Z7_PLAF7	Unknown	Unknown	Down	Down	Down	**Down**
gi|23498950	zinc finger, C3HC4 type, putative	253816	8.32	32	15.5	Q8IBH2_PLAF7	Unknown	Unknown	Down	Down	-	**Down**
gi|13509187	putative Rab7 GTPase	23773	7.55	55	28.6	C0H516_PLAF7	Unknown	Membrane	-	-	-	-
gi|23615267	conserved Plasmodium protein, unknown function	34955	9.22	32	26.6	Q8IEJ9_PLAF7	Unknown	Unknown	Down	-	-	-
gi|124808655	surface protein P113	112505	4.49	31	8.6	Q8ILP3_PLAF7	Unknown	Membrane	Down	-	-	-
gi|258597176	erythrocyte membrane protein 1, PfEMP1	248280	5.47	34	17.1	Q8IIZ6_PLAF7	Pathogenesis	Membrane	-	-	-	-
gi|23504621	deoxyribodipyrimidine photo-lyase, putative	129117	9.22	34	11.6	Q8I0W8_PLAF7	DNA Replication	Unknown	-	-	-	-
gi|23615206	conserved Plasmodium protein, unknown function	56775	9.53	34	11.9	Q8IEQ3_PLAF7	Unknown	Unknown	-	-	-	-
gi|258597334	26S proteasome regulatory subunit RPN7,putative	46595	6.38	31	13	Q8II71_PLAF7	Protein fate	Cytoplasm	Down	-	-	-
gi|225632017	conserved Plasmodium protein, unknown function	696614	7.88	39	11	C0H5H6_PLAF7	Unknown	Unknown	-	Down	-	-
gi|23615391	60S ribosomal protein L23	22079	10.27	30	36.3	Q8IE82_PLAF7	Translation	Cytoplasm	-	-	Up	-
gi|225632182	conserved Plasmodium protein, unknown function	126994	5.28	39	12	C0H4P6_PLAF7	Unknown	Unknown	Down	-	Down	**Down**
gi|23498915	conserved Plasmodium protein, unknown function	36570	8.79	39	22	Q8IBK7_PLAF7	Unknown	Unknown	Down	-	Down	**Down**
gi|23615173	conserved Plasmodium protein, unknown function	95550	9.45	42	15.6	Q8IET6_PLAF7	Unknown	Unknown	-	-	-	-
gi|23498906	metallo-hydrolase/oxidoreductase, putative	119584	7.01	47	16.9	Q8IBL6_PLAF7	Unknown	Membrane	Down	Up	-	-
gi|23615182	conserved Plasmodium protein, unknown function	1111079	9.12	30	15.6	Q8IES7_PLAF7	Unknown	Membrane	Down	-	Down	**Down**
gi|225632238	conserved Plasmodium protein, unknown function	36234	4.11	68	29.2	C0H4U4_PLAF7	Unknown	Unknown	Up	-	-	-
gi|58176834	Chain A, D-ribulose-5-phosphate 3-epimerase, Putative	25532	6.07	41	30.4	Q8I5L3_PLAF7	Metabolism	Cytoplasm	-	-	-	-
gi|124809637	conserved Plasmodium protein, unknown function	442796	8.64	29	10.6	Q8IKY8_PLAF7	Unknown	Unknown	-	Up	-	-
gi|23498938	DNA (cytosine-5)-methyltransferase	83731	8.74	29	9.6	Q8IBI4_PLAF7	DNA Replication	Nucleus	Down	-	Up	-
gi|408535927	Chain A, Apicoplast Tic22, Putative	33232	9.24	41	38.4	Q8I3H4_PLAF7	Transport	Apicoplast	-	Up	-	-
gi|225631857	conserved Plasmodium protein, unknown function	138544	8.44	45	15.7	C0H575_PLAF7	Unknown	Membrane	-	-	-	-
gi|23504582	asparagine--tRNA ligase	85195	9.01	32	22.3	Q8I408_PLAF7	Translation	Cytoplasm	-	-	Up	-
gi|23615371	3~,5~-cyclic nucleotide phosphodiesterase,putative	133084	7.47	30	6.8	Q8I6Z7_PLAF7	Protein Signaling	Membrane	Down	-	-	-
gi|3649757	conserved Plasmodium protein, unknown function	202018	8.3	29	16.5	O77322_PLAF7	Unknown	Cytoplasm	-	Down	Down	**Down**

gi|258597702	40S ribosomal protein S25	11656	10.12	44	55.2	Q8ILN8_PLAF7	Translation	Cytoplasm	Down	-	Down	**Down**
gi|23615215	U3 small nucleolar RNA-associated protein 6,putative	120650	5.51	29	5.6	Q8IEP4_PLAF7	Transcription	Membrane	Down	-	Up	-
gi|124804079	exported protein 1	17285	5.64	102	35.8	Q8IIF0_PLAF7	Metabolism	Membrane	-	-	-	-
gi|124801337	vacuolar protein sorting-associated protein 45,putative	86202	6.51	33	5.7	O96243_PLAF7	Transport	Cytoplasm	-	Up	-	-
gi|74876421	RecName: Full=ADP-ribosylation factor 1; Short=pfARF1	20899	5.83	52	33.1	ARF1_PLAF7	Transport	Golgi Apparatus	-	-	-	-
gi|46362284	conserved Plasmodium protein, unknown function	25357	9.82	33	30.1	C6KSQ0_PLAF7	Unknown	Unknown	-	Down	-	-
gi|7264037	erythrocyte membrane protein 1, PfEMP1	250321	5.29	26	17	O97324_PLAF7	Pathogenesis	Membrane	Down	-	-	-
gi|124804967	Plasmodium exported protein, unknown function	51125	5.66	25	14.7	Q8IHN2_PLAF7	Unknown	Unknown	Down	-	-	-
gi|124804772	60S ribosomal protein L35ae, putative	16255	10.55	25	26.4	Q8IHT9_PLAF7	Translation	Cytoplasm	Down	Up	Down	**Down**
gi|124808373	conserved Plasmodium protein, unknown function	390446	9.23	25	11.7	Q8ILX0_PLAF7	Unknown	Unknown	Up	Down	Down	**Down**
gi|124809878	transcription factor with AP2 domain(s)	161410	8.98	36	8.2	Q8IKR9_PLAF7	Unknown	Unknown	Down	-	Up	-
gi|124806892	FK506-binding protein (FKBP)-type peptidyl-prolyl isomerase	34805	5.36	37	24.3	Q8I4V8_PLAF7	Protein fate	Cytoplasm	-	-	-	-
gi|23504953	mitochondrial carrier protein, putative	141981	9.63	28	20.9	Q8I397_PLAF7	Transport	Membrane	Down	-	Up	-
gi|23504600	RNA pseudouridylate synthase, putative	1186842	7.36	24	12.8	MLRR1_PLAF7	Metabolism	Membrane	-	-	-	-
gi|17148533	Ran-binding protein	33176	4.92	57	16.4	Q76NN6_PLAF7	DNA Replication	Cytoplasm	Up	-	Up	**Up**
gi|23505262	Plasmodium exported protein (PHISTc), unknown function	45472	9.71	33	16.4	Q8I2F2_PLAF7	Unknown	Membrane	Down	Up	Up	**Up**
gi|6562716	conserved Plasmodium protein, unknown function	229981	6.44	27	10.8	Q9U0K8_PLAF7	Unknown	Unknown	Down	Up	-	-
gi|258549210	conserved Plasmodium protein, unknown function	30341	7.64	29	18.6	C6S3I8_PLAF7	Unknown	Unknown	Down	Up	Up	**Up**
gi|258597832	conserved Plasmodium protein, unknown function	145360	8.39	23	9.4	Q8IKZ3_PLAF7	Unknown	Nucleus	Down	-	-	-
gi|74862955	RecName: Full=Origin recognition complex subunit 1; Short=PfORC1	138653	9.52	27	14.6	ORC1_PLAF7	DNA Replication	Cytoplasm	-	-	Up	-
gi|23615256	meiosis-specific nuclear structural protein 1,putative	61194	8.91	30	18.8	Q8IEK6_PLAF7	Unknown	Unknown	-	Up	-	-
gi|3649767	SECIS-binding protein 2, putative	71419	9.94	30	16.9	O77331_PLAF7	Translation	Unknown	-	Up	-	-
gi|23498865	60S ribosomal protein L11a, putative	20215	10.1	36	25.4	Q8IBQ6_PLAF7	Translation	Cytoplasm	-	-	-	-
gi|23615806	secreted ookinete protein, putative	135752	4.66	37	16.1	Q8ID30_PLAF7	Unknown	Unknown	-	-	-	-
gi|1575675	rab6	27914	7.63	108	28.3	Q94663_PLAFA	Unknown	Membrane	-	-	Down	-
gi|23504877	conserved Plasmodium protein, unknown function	225390	6.01	30	16	Q8I3H0_PLAF7	Unknown	Unknown	Up	Down	Down	**Down**
gi|23499018	magnesium transporter, putative	109581	9.53	23	10	Q8IBA9_PLAF7	Transport	Membrane	Down	-	-	-
gi|23510645	conserved Plasmodium protein, unknown function	65626	9.65	23	11.4	Q8I271_PLAF7	Unknown	Unknown	Down	-	-	-
gi|23615192	DNA-directed RNA polymerase II subunit RPB11,putative	14116	8.37	30	31	RPB11_PLAF7	Transcription	Cytoplasm	Down	-	-	-
gi|258597760	conserved Plasmodium membrane protein, unknown function	682022	8.86	26	13.8	Q8ILC9_PLAF7	Unknown	Unknown	Down	-	-	-
gi|225632153	cytoskeleton associated protein, putative	103523	6.43	22	7.9	C0H4M2_PLAF7	Unknown	Unknown	Down	-	-	-
gi|225631936	MORN repeat protein, putative	519893	9.15	29	10.3	C0H5A3_PLAF7	Unknown	Membrane	-	Down	Down	**Down**
gi|124808756	conserved Plasmodium protein, unknown function	192484	9.67	29	16.8	Q8ILL7_PLAF7	Unknown	Membrane	-	Down	Down	**Down**
gi|23615179	sodium/hydrogen exchanger, Na+, H+ antiporter	225940	8.68	29	11.4	Q8IET0_PLAF7	Transport	Membrane	Up	Down	Down	**Down**
gi|124804234	autophagy-related protein 7, putative	156530	6.05	29	18.2	Q8IIA3_PLAF7	Protein fate	Cytoplasm	-	Down	Down	**Down**
gi|124806636	conserved Plasmodium protein, unknown function	212627	4.96	29	12.8	Q8I538_PLAF7	Unknown	Unknown	-	Down	Down	**Down**
gi|23504575	conserved Plasmodium protein, unknown function	369953	5.29	29	9	Q8I414_PLAF7	Unknown	Unknown	-	Down	Down	**Down**
gi|124809084	conserved Plasmodium protein, unknown function	152167	8.42	29	12.9	Q8ILD1_PLAF7	Unknown	Unknown	-	Down	Down	**Down**
gi|124805631	conserved Plasmodium protein, unknown function	208541	8.3	29	15.2	Q8I5W3_PLAF7	Unknown	Unknown	-	Down	Down	**Down**
gi|124802600	conserved Plasmodium protein, unknown function	190279	8.29	29	10.9	Q8IJF5_PLAF7	Unknown	Unknown	-	Down	Down	**Down**
gi|124804432	conserved Plasmodium protein, unknown function	133324	5.18	29	17.4	Q8II44_PLAF7	Unknown	Unknown	-	Down	Down	**Down**
gi|124804384	conserved Plasmodium protein, unknown function	118257	8.84	29	14.6	Q8II59_PLAF7	Unknown	Unknown	-	Down	Down	**Down**
gi|23504862	eukaryotic translation initiation factor 3 subunit E, putative	61379	7.08	49	23.4	Q8I3I5_PLAF7	Translation	Cytoplasm	-	Down	Down	**Down**
gi|225632254	rRNA-processing protein FCF1, putative	23145	9.63	29	23.2	C0H4V7_PLAF7	Unknown	Nucleus	Down	Down	Down	**Down**
gi|225631798	conserved protein, unknown function	296598	9.02	22	15.4	C0H518_PLAF7	Unknown	Membrane	Down	Down	-	**Down**
gi|124806350	subpellicular microtubule protein 2, putative	30171	9.31	31	21.7	Q8I5B7_PLAF7	Unknown	Unknown	-	-	-	-
gi|124809790	conserved Plasmodium protein, unknown function	233153	7.99	22	7.5	Q8IKU4_PLAF7	Unknown	Cytoplasm	Down	-	-	-
gi|46362277	conserved Plasmodium protein, unknown function	334204	8.49	22	18.4	C6KSP3_PLAF7	Unknown	Unknown	Down	Up	Up	**Up**
gi|225632011	heat shock protein 110, putative	108119	5.5	95	16.8	C0H5H0_PLAF7	Unknown	Apicoplast	-	Up	Up	**Up**
gi|124809582	M17 leucyl aminopeptidase	67778	8.78	83	24.3	Q8IL11_PLAF7	Unknown	Cytoplasm	-	Up	-	-
gi|46362309	conserved Plasmodium protein, unknown function	324818	6.62	25	10.5	LRR2_PLAF7	Unknown	Unknown	Up	-	Up	**Up**
gi|124806590	conserved Plasmodium protein, unknown function	333978	9.16	21	12.4	Q8I552_PLAF7	Unknown	Unknown	Down	-	-	-
gi|258597800	conserved Plasmodium protein, unknown function	152700	7.78	26	16	Q8IL66_PLAF7	Unknown	Cytoplasm	Down	-	Up	-
gi|14530178	Krueppel-like protein	151550	7.89	33	11.5	Q95Z58_PLAFA	Unknown	Membrane	Down	-	Down	**Down**
gi|124810028	conserved Plasmodium protein, unknown function	118694	8.96	27	10.1	Q8IKN6_PLAF7	Unknown	Unknown	Down	-	-	-
gi|124805005	erythrocyte membrane protein 1, PfEMP1	358992	5.63	21	15.5	Q8IHM0_PLAF7	Pathogenesis	Nucleus	Down	-	-	-
gi|296005130	Pfmc-2TM Maurer~s cleft two transmembrane protein	27380	9.4	25	31.2	A0A143ZWD4_PLAF7	Unknown	Membrane	Up	Up	-	**Up**
gi|23498719	Pfmc-2TM Maurer~s cleft two transmembrane protein	27539	9.59	25	21.3	Q8IC51_PLAF7	Transport	Membrane	-	Up	-	-
gi|23498897	conserved Plasmodium protein, unknown function	66258	8.46	21	12.3	Q8IBM5_PLAF7	Unknown	Unknown	Down	-	-	-
gi|124802749	tRNA N6-adenosine threonylcarbamoyltransferase	69530	7.49	20	15.2	Q8IJ99_PLAF7	Translation	Cytoplasm	Down	-	-	-

gi|23505246	vacuolar protein sorting-associated protein 33,putative	135894	4.8	20	6.6	Q8I2G8_PLAF7	Transport	Unknown	Down	-	-	-
gi|225631933	conserved Plasmodium protein, unknown function	418313	6.69	20	14.9	C0H5A0_PLAF7	Unknown	Unknown	Down	-	-	-
gi|225631966	conserved Plasmodium protein, unknown function	404851	6.56	20	15.7	C0H5C8_PLAF7	Unknown	Membrane	-	Down	-	-
gi|23498787	60S ribosomal protein L34	17340	10.77	24	10.7	Q8IBY4_PLAF7	Translation	Cytoplasm	Down	-	Down	**Down**
gi|224591371	conserved Plasmodium protein, unknown function	109211	6.62	20	18.7	B9ZSI3_PLAF7	Unknown	Unknown	Down	-	-	-
gi|74864044	RecName: Full=NAD-dependent protein deacetylase Sir2B; AltName: Full=Regulatory protein SIR2 homolog B; AltName: Full=SIR2-like protein B	154624	8.15	23	15.4	SIR2B_PLAF7	Transcription	Unknown	Down	Up	-	-
gi|13375179	putative GTPase	22872	6.18	47	16	Q7K6A8_PLAF7	Transport	Membrane	Down	-	Down	**Down**
gi|124808388	AAA family ATPase, putative	142737	9.27	20	16.8	Q8ILW7_PLAF7	Unknown	Unknown	Down	-	-	-
gi|124804419	conserved Plasmodium protein, unknown function	330099	6.16	21	12.1	Q8II48_PLAF7	Unknown	Unknown	Down	Down	-	**Down**
gi|124808513	metacaspase-like protein	264761	9.31	20	9.3	Q8ILT4_PLAF7	Unknown	Unknown	Down	-	-	-
gi|23498907	conserved Plasmodium protein, unknown function	260606	5.49	22	12.1	Q8IBL5_PLAF7	Unknown	Unknown	Up	-	-	-
gi|225631649	transcription factor with AP2 domain(s)	399922	5.68	19	8	AP2A_PLAF7	Transcription	Nucleus	Down	-	-	-
gi|258597770	dynein-related AAA-type ATPase, putative	970399	8.07	21	12.6	Q8ILB9_PLAF7	Translation	Cytoplasm	Down	-	-	-
gi|225632063	conserved Plasmodium protein, unknown function	346585	7.11	21	12.6	C0H5L6_PLAF7	Unknown	Membrane	Down	-	-	-
gi|23505158	conserved Plasmodium protein, unknown function	208459	7.88	19	7.7	Q8I2Q1_PLAF7	Unknown	Cytoplasm	Down	-	-	-
gi|124802000	hypothetical protein PF3D7_1004100	141831	8.61	20	15.4	Q8IJZ6_PLAF7	Unknown	Unknown	Down	-	-	-
gi|124808735	nucleolar GTP-binding protein 2, putative	57136	9.72	23	16.2	Q8ILM2_PLAF7	Metabolism	Nucleus	-	Up	-	-
gi|225685560	6-cysteine protein	114662	6.28	20	7.5	C0H478_PLAF7	Unknown	Membrane	Down	-	-	-
gi|124809716	conserved Plasmodium protein, unknown function	176123	5.42	23	10.2	Q8IKW4_PLAF7	Unknown	Unknown	Down	-	-	-
gi|46361233	HECT-domain (ubiquitin-transferase), putative	1205255	8.19	25	13.9	ALTH1_PLAF7	Unknown	Cytoplasm	-	-	-	-
gi|124808084	COBW domain-containing protein 1, putative	67839	6.04	19	7.3	Q8IM39_PLAF7	Unknown	Unknown	Down	-	-	-
gi|3758839	P-type ATPase, putative	217882	8.09	23	10.2	O77368_PLAF7	Transport	Membrane	-	-	Up	-
gi|124801012	serine repeat antigen 7	109569	5.57	23	17.1	O96163_PLAF7	Cell Surface	Lysosome	-	-	Up	-
gi|4493893	conserved Plasmodium protein, unknown function	474832	8.71	30	12.7	O97236_PLAF7	Unknown	Membrane	Down	-	-	-
gi|46361095	conserved protein, unknown function	30831	5.62	19	26.3	C6KSY5_PLAF7	Unknown	Nucleus	Down	-	-	-
gi|124809249	conserved Plasmodium protein, unknown function	25471	9.37	19	20.8	Q8IL93_PLAF7	Unknown	Unknown	Down	-	-	-
gi|46361088	conserved Plasmodium protein, unknown function	123646	5.86	25	11.1	C6KSX8_PLAF7	Unknown	Membrane	Down	Up	-	-
gi|23505265	Plasmodium exported protein, unknown function	31267	9.69	20	8.9	Q8I2E9_PLAF7	Unknown	Membrane	Down	Up	Up	**Up**
gi|3649758	T-complex protein 1 subunit eta	59540	5.45	52	26	TCPH_PLAF7	Protein fate	Cytoplasm	-	-	Up	-
gi|124801939	tryptophan-rich antigen 3	118597	5.41	23	9.8	Q8IK14_PLAF7	Unknown	Membrane	-	-	-	-
gi|258597255	SET domain protein, putative	94234	4.47	18	12.5	Q8IIL1_PLAF7	Unknown	Unknown	Down	-	-	-
gi|124806112	phospholipid-transporting ATPase, putative	190326	8.74	18	11.2	Q8I5I3_PLAF7	Transport	Membrane	Down	Up	-	-
gi|124804153	conserved Plasmodium protein, unknown function	153603	6.77	24	13.5	Q8IIC8_PLAF7	Unknown	Unknown	-	-	-	-
gi|730436	RecName: Full=Dihydroorotate dehydrogenase (quinone), mitochondrial; Short=DHOdehase; AltName: Full=Dihydroorotate oxidase; Flags: Precursor	65517	9.14	32	13.7	PYRD_PLAF7	Metabolism	Membrane	Down	-	-	-
gi|23498946	conserved Plasmodium membrane protein, unknown function	670693	9.3	17	8.1	Q8IBH6_PLAF7	Unknown	Membrane	Down	-	-	-
gi|23498781	conserved Plasmodium protein, unknown function	407134	9.85	17	9.2	Q8IBZ0_PLAF7	Unknown	Cytoplasm	Down	-	-	-
gi|225632037	conserved Plasmodium protein, unknown function	439635	7.81	27	7.9	C0H5J3_PLAF7	Unknown	Unknown	-	Down	-	-
gi|225631835	nitric oxide synthase, putative	92122	9.01	17	14.1	C0H553_PLAF7	Unknown	Unknown	Down	-	-	-
gi|46361075	leucine-rich repeat protein	220157	5.56	17	17.8	C6KSW6_PLAF7	Unknown	Unknown	Down	-	-	-
gi|258597622	conserved Plasmodium protein, unknown function	142616	9.55	16	14	Q8IM60_PLAF7	Unknown	Unknown	Down	-	-	-
gi|258597535	60 kDa chaperonin	81434	4.97	30	22.8	Q8I0V3_PLAF7	Protein fate	Apicoplast	Up	Up	-	**Up**
gi|258597961	cysteine repeat modular protein 4	700473	8.43	37	12.9	Q8IK84_PLAF7	Transport	Membrane	-	-	Up	-
gi|23505139	RNA-binding protein, putative	22965	9.51	27	20.3	Q8I2R8_PLAF7	Unknown	Unknown	Down	-	Down	**Down**
gi|7670012	rifin	39495	9.15	18	27.3	Q9NFB0_PLAF7	Cell Surface	Membrane	-	-	Down	-
gi|3764022	ATP-dependent RNA helicase DHX57, putative	267066	8.4	15	12.7	O77360_PLAF7	Transcription	Cytoplasm	Down	-	-	-
gi|23498174	pre-mRNA-processing-splicing factor 8, putative	366166	8.79	15	6.2	Q8I1X5_PLAF7	Transcription	Nucleus	Down	-	-	-
gi|23504539	conserved Plasmodium protein, unknown function	89840	9.52	15	13.1	Q8I448_PLAF7	Unknown	Unknown	Down	-	-	-
gi|258549051	DnaJ protein, putative	94626	6.66	15	14.6	C6S3C2_PLAF7	Unknown	Unknown	Down	-	-	-
gi|23504963	conserved Plasmodium protein, unknown function	69116	9.51	15	17.3	Q8I387_PLAF7	Unknown	Membrane	Down	-	-	-
gi|258597286	palmitoyltransferase, putative	34757	8.24	15	19.8	Q8IIF7_PLAF7	Unknown	Membrane	Down	-	-	-
gi|258597253	glycerol-3-phosphate dehydrogenase, putative	44846	8.81	15	14.7	Q8IIL4_PLAF7	Metabolism	Cytoplasm	Down	-	-	-
gi|46361274	erythrocyte membrane protein 1, PfEMP1	456629	5.92	26	14.5	C6KTF7_PLAF7	Pathogenesis	Membrane	Down	-	-	-
gi|124803766	conserved Plasmodium protein, unknown function	709774	9.02	14	8	Q8IIN2_PLAF7	Unknown	Unknown	Down	-	-	-
gi|124802097	conserved Plasmodium protein, unknown function	452722	8.74	22	12.1	Q8IJW2_PLAF7	Unknown	Unknown	-	-	-	-
gi|23499052	exonuclease, putative	48070	8.91	14	34.2	Q8IB75_PLAF7	Unknown	Unknown	-	-	-	-
gi|23615467	60S ribosomal protein L23, putative	14983	9.9	39	62.6	Q8IE09_PLAF7	Translation	Cytoplasm	-	Down	Up	-
gi|124804504	heat shock protein 70	73252	6.51	228	30.5	Q8II24_PLAF7	Protein fate	Mitochondria	Up	-	-	-
gi|124808181	plasmepsin III	51661	8.05	202	35.9	Q8IM15_PLAF7	Metabolism	Food Vacuole	Up	-	Down	-
gi|47169189	Chain A, Uridine Phosphorylase, Putative	27745	6.32	188	44.3	Q8I3X4_PLAF7	Metabolism	Unknown	Up	Up	-	**Up**
gi|23615408	phosphoribosylpyrophosphate synthetase	49352	9.39	151	26.3	Q8IE67_PLAF7	Metabolism	Unknown	Up	Up	-	**Up**
gi|10129955	S-adenosylmethionine synthetase	44816	6.28	250	31.1	Q9GN14_PLAFA	Metabolism	Unknown	Up	Up	-	**Up**
gi|23615594	DNA/RNA-binding protein Alba 2	24969	7.68	233	39.3	Q8IDN4_PLAF7	Unknown	Cytoplasm	Up	-	-	-
gi|322812543	Chain A, Glucose-6-phosphate isomerase	69517	6.9	144	24.3	Q8ILA4_PLAF7	Metabolism	Unknown	Up	-	Up	**Up**
gi|124809020	60S ribosomal protein L14, putative	19285	10.2	65	14.5	Q8ILE8_PLAF7	Translation	Cytoplasm	Up	-	-	-
gi|124810100	40S ribosomal protein S28e, putative	7489	10.83	329	50.7	Q8IKL9_PLAF7	Translation	Cytoplasm	Up	-	-	-
gi|13397937	putative Rab2 GTPase	24394	6.33	60	23.9	Q9BHT6_PLAFA	Unknown	Unknown	Up	-	Down	-
gi|46361134	60S ribosomal protein L27a, putative	16712	10.54	59	31.8	C6KT23_PLAF7	Translation	Cytoplasm	Up	Up	-	**Up**
gi|225631680	serine/arginine-rich splicing factor 1	34540	10.61	51	12.8	Q8I3T5_PLAF7	Transcription	Nucleus	Up	-	-	-

gi|45478047	macrophage migration inhibitory factor-like protein	12836	6.15	396	56.9	Q6Q3H7_PLAFA	Unknown	Unknown	Up	-	-	-
gi|23504502	rhoptry-associated protein 2	46709	8.9	57	25.6	Q8I484_PLAF7	Invasion	Rhoptry	Up	-	-	-
gi|4493905	40S ribosomal protein S23, putative	16120	10.83	66	33.8	O97248_PLAF7	Translation	Cytoplasm	Up	-	Up	**Up**
gi|23615434	proteasome subunit beta type-7, putative	29942	7.98	62	27.4	Q8I6T3_PLAF7	Protein fate	Cytoplasm	Up	-	-	-
gi|46362290	DNA repair protein RAD50, putative	267786	8.78	39	17.6	RAD50_PLAF7	Cell Cycle	Nucleus	Up	Up	-	**Up**
gi|23615274	Plasmodium exported protein (hyp12), unknown function	45997	4.62	39	7.3	Q8IEJ2_PLAF7	Unknown	Unknown	Up	-	-	-
gi|23510644	centrin-1	19587	4.78	39	11.9	Q8I272_PLAF7	Unknown	Unknown	Up	-	-	-
gi|23498308	Plasmodium exported protein (PHISTb), unknown function	35939	8.75	88	32	Q8IFM0_PLAF7	Unknown	Unknown	Up	-	Up	**Up**
gi|7340797	cytoadherence linked asexual protein 3.2	167382	6.74	36	13	O77309_PLAF7	Cell Surface	Membrane	Up	-	Down	-
gi|4493872	cytoadherence linked asexual protein 3.1	167134	6.75	36	13.5	O77310_PLAF7	Cell Surface	Membrane	Up	-	Down	-
gi|124809004	conserved Plasmodium protein, unknown function	146396	9.2	36	9.9	Q8ILF3_PLAF7	Unknown	Unknown	Up	-	-	-
gi|23499061	RNA-binding protein, putative	32421	9.11	81	15.4	Q8IB66_PLAF7	Translation	Unknown	Up	-	Up	**Up**
gi|124803451	60S acidic ribosomal protein P1, putative	13006	4.57	82	39	Q8IIX0_PLAF7	Translation	Cytoplasm	Up	-	-	-
gi|258596969	conserved Plasmodium protein, unknown function	14508	6.09	35	33.9	Q8IK07_PLAF7	Unknown	Unknown	Up	-	-	-
gi|124803615	casein kinase 2, alpha subunit	39865	8.9	50	23	Q8IIR9_PLAF7	Regulation	Cytoplasm	Up	-	-	-
gi|23498804	erythrocyte membrane protein 1, PfEMP1	248785	5.3	33	15.3	Q8IBW7_PLAF7	Pathogenesis	Membrane	Up	-	-	-
gi|46361054	conserved Plasmodium protein, unknown function	244517	6.06	33	8	C6KSU6_PLAF7	Unknown	Unknown	Up	-	-	-
gi|3694805	cytoadherence linked asexual protein, partial	160994	8.98	42	20.1	O77090_PLAFA	Unknown	Membrane	Up	Up	-	**Up**
gi|23505252	cytoadherence linked asexual protein 9	160313	8.88	42	17.1	Q8I2G2_PLAF7	Cell Surface	Membrane	Up	Up	Up	**Up**
gi|124805343	erythrocyte membrane protein 1, PfEMP1	333038	6.39	32	18.1	Q8I640_PLAF7	Pathogenesis	Membrane	Up	-	-	-
gi|124806687	erythrocyte membrane protein 1, PfEMP1	300099	5.43	36	13.2	Q8I521_PLAF7	Pathogenesis	Membrane	Up	Down	-	-
gi|46361276	erythrocyte membrane protein 1, PfEMP1	254458	5.66	36	18.5	C6KTF9_PLAF7	Pathogenesis	Membrane	Up	Down	-	-
gi|124801366	ATP synthase F1, alpha subunit	61731	8.72	33	19.6	O96252_PLAF7	Metabolism	Mitochondria	Up	Down	Up	**Up**
gi|116668029	Chain A, Spermidine Synthase	32162	6.18	139	34.6	Q8II73_PLAF7	Metabolism	Cytoplasm	Up	-	-	-
gi|258596882	serine repeat antigen 6	118761	5.89	43	16.3	Q9TY96_PLAF7	Cell Surface	Lysosome	Up	-	-	-
gi|8439487	hypothetical protein, partial	23455	5.87	61	14.6	Q9NNV9_PLAFA	Unknown	Membrane	Up	-	Up	**Up**
gi|34305467	membrane-associated histidine-rich protein, partial	26918	6.06	31	10.8	Q6V0W8_PLAFA	Unknown	Membrane	Up	-	-	-
gi|15375389	60S ribosomal protein L7, putative	30504	10.35	55	18.7	O97250_PLAF7	Translation	Cytoplasm	Up	Down	-	-
gi|124805983	clathrin heavy chain, putative	232803	6	38	12.3	Q8I5L6_PLAF7	Transport	Cytoplasm	Up	Down	Up	**Up**
gi|23499287	hypothetical protein, partial	82064	9.28	30	12.6	C0H4L7_PLAF7	Unknown	Membrane	Up	Down	-	-
gi|124805775	eukaryotic translation initiation factor 3 subunit A, putative	165959	6.38	30	9.4	Q8I5S6_PLAF7	Translation	Cytoplasm	Up	-	-	-
gi|23505053	conserved Plasmodium protein, unknown function	85353	9.1	32	12.3	Q8I2Z9_PLAF7	Unknown	Unknown	Up	-	Up	**Up**
gi|23505032	elongation factor 1-beta	32007	4.94	50	25.4	Q8I320_PLAF7	Unknown	Unknown	Up	Up	-	**Up**
gi|124804341	parasitophorous vacuolar protein 1	51919	4.97	29	24.3	Q8II72_PLAF7	Unknown	Cytoplasm	Up	-	Up	**Up**
gi|23498743	tRNA m5C-methyltransferase, putative	141140	6.35	30	17	Q8IC28_PLAF7	Regulation	Unknown	Up	Up	Down	**Up**
gi|23504571	guanidine nucleotide exchange factor	303926	8.53	28	7.4	Q8I418_PLAF7	DNA Replication	Nucleus	Up	-	-	-
gi|258597440	antigen 332, DBL-like protein	688870	3.86	45	5.2	Q8IHN4_PLAF7	Pathogenesis	Membrane	Up	-	Up	**Up**
gi|124808633	conserved Plasmodium protein, unknown function	170332	5.89	27	13.7	Q8ILP9_PLAF7	Unknown	Unknown	Up	-	-	-
gi|124808549	NOT family protein, putative	519499	6.88	27	11.8	Q8ILS4_PLAF7	Translation	Cytoplasm	Up	Up	-	**Up**
gi|23504536	trafficking protein particle complex subunit 8,putative	328469	7.22	30	12.6	Q8I451_PLAF7	Unknown	Unknown	Up	-	-	-
gi|23498839	eukaryotic translation initiation factor 3 subunit I, putative	37261	6.43	36	19.6	Q8IBT2_PLAF7	Translation	Cytoplasm	Up	Down	Down	**Down**
gi|356624409	Chain A, Translationally-controlled Tumor Protein Homolog	21594	4.9	26	19.7	TCTP_PLAF7	Cell Cycle	Cytoplasm	Up	Up	-	**Up**
gi|124806152	histone chaperone ASF1, putative	31527	4.37	26	11.7	Q8I5H2_PLAF7	Nucleosome Assembly	Nucleus	Up	-	-	-
gi|59797635	RecName: Full=Glycophorin-binding protein; AltName: Full=GBP-130	95786	5.02	26	14.9	GBP_PLAF7	Unknown	Cytoplasm	Up	-	-	-
gi|402550052	Chain A, Deoxyuridine 5~-triphosphate Nucleotidohydrolase, Putative	20626	6.59	80	27.6	Q8II92_PLAF7	DNA Replication	Unknown	Up	-	-	-
gi|23504945	signal peptidase complex subunit 3, putative	22194	9.34	25	16.2	Q8I3A5_PLAF7	Protein Signaling	Membrane	Up	-	-	-
gi|225631760	transcription factor with AP2 domain(s),putative	237219	9	24	10.2	C6KT65_PLAF7	Transcription	Unknown	Up	-	-	-
gi|124808263	alpha/beta hydrolase, putative	41458	6.51	25	13.8	Q8ILZ4_PLAF7	Unknown	Unknown	Up	-	-	-
gi|23504898	lysine-rich membrane-associated PHISTb protein	61048	9.34	100	27.5	Q8I3F0_PLAF7	Unknown	Cytoplasm	Up	-	-	-
gi|225631686	proteasome subunit beta type-1, putative	27251	6.59	24	9.6	C0H4E8_PLAF7	Protein fate	Cytoplasm	Up	-	-	-
gi|23615433	conserved Plasmodium protein, unknown function	319935	7.32	25	15.5	Q8IE42_PLAF7	Unknown	Unknown	Up	Down	Down	**Down**
gi|23499096	conserved Plasmodium protein, unknown function	170170	6.4	27	12.1	Q8IB33_PLAF7	Unknown	Unknown	Up	Up	-	**Up**
gi|225632226	peptidase family C50, putative	697746	8.41	30	11.9	C0H4T3_PLAF7	Unknown	Nucleus	Up	-	-	-
gi|124806110	DNA gyrase subunit A	143055	9.39	23	9.8	Q8I0X3_PLAF7	DNA Replication	Apicoplast	Up	-	-	-
gi|124805429	high mobility group protein B1	11324	9.96	51	25.8	Q8I616_PLAF7	Transcription	Nucleus	Up	-	Down	-
gi|124810337	conserved Plasmodium protein, unknown function	68624	8.96	23	9.7	Q8IKD1_PLAF7	Unknown	Unknown	Up	-	-	-
gi|23505130	DNA-directed RNA polymerase II subunit RPB3,putative	38322	6.01	23	26.3	Q8I2S6_PLAF7	Transcription	Cytoplasm	Up	-	-	-
gi|23504860	proteasome maturation factor UMP1, putative	14594	5.27	22	5.6	Q8I3I7_PLAF7	Unknown	Cytoplasm	Up	-	-	-
gi|74862993	RecName: Full=Uncharacterized protein PFB0765w	166903	6.19	25	17.1	YPF11_PLAF7	Unknown	Unknown	Up	Down	Up	**Up**
gi|8248757	DNA polymerase alpha catalytic subunit A	225260	8.55	22	14.2	Q9U0H1_PLAF7	DNA Replication	Nucleus	Up	-	-	-
gi|23615691	ubiquitin-conjugating enzyme, putative	22869	5.32	40	17.3	Q8IDD9_PLAF7	Protein fate	Cytoplasm	Up	-	Up	**Up**
gi|258597812	conserved Plasmodium protein, unknown function	70994	9.15	24	19	Q8IL39_PLAF7	Unknown	Unknown	Up	Up	-	**Up**
gi|74929507	RecName: Full=Actin-1; AltName: Full=Actin I	41844	5.21	78	24.7	ACT1_PLAF7	Cytoskeleton	Cytoplasm	Up	-	Up	**Up**
gi|124810134	conserved Plasmodium protein, unknown function	97606	9.6	21	10.3	Q8IKK6_PLAF7	Unknown	Unknown	Up	-	-	-
gi|23498914	mitochondrial import inner membrane translocase subunit TIM14, putative	13044	10.09	21	22.6	Q8IBK8_PLAF7	Unknown	Unknown	Up	-	Up	**Up**
gi|23498760	RAP protein, putative	142168	8.58	21	7.8	Q8IC11_PLAF7	Unknown	Membrane	Up	-	-	-
gi|23498806	Pfmc-2TM Maurer~s cleft two transmembrane protein	27311	9.57	21	21.2	Q8IBW5_PLAF7	Transport	Membrane	Up	-	-	-
gi|23615656	P-loop containing nucleoside triphosphate hydrolase, putative	304114	6.24	21	10	Q8IDH3_PLAF7	Transcription	Nucleus	Up	-	-	-
gi|23498260	conserved Plasmodium protein, unknown function	296268	5.86	20	6.7	Q8I1P3_PLAF7	Unknown	Membrane	Up	-	-	-

gi|225631696	conserved Plasmodium protein, unknown function	1116481	9.37	39	11.1	C0H4F8_PLAF7	Unknown	Membrane	Up	Up	Down	**Up**
gi|23498206	structural maintenance of chromosomes protein 3,putative	141137	6.48	20	20.7	SMC3_PLAF7	Cell Cycle	Nucleus	Up	-	-	-
gi|23615774	aldehyde reductase, putative	106055	9.18	20	15.9	Q8ID61_PLAF7	Unknown	Apicoplast	Up	-	-	-
gi|124802266	conserved Plasmodium protein, unknown function	173168	5.76	20	9.1	Q8IJQ4_PLAF7	Unknown	Unknown	Up	-	-	-
gi|124802886	U2 snRNA/tRNA pseudouridine synthase, putative	116647	8.58	20	12.3	Q8IJ59_PLAF7	Metabolism	Nucleus	Up	-	-	-
gi|124802424	uncharacterized protein PF3D7_1018800	85496	9.49	20	5.2	Q8IJL4_PLAF7	Unknown	Unknown	Up	-	-	-
gi|124805367	RESA-like protein with PHIST and DnaJ domains	107558	7.04	20	14	Q8I634_PLAF7	Unknown	Unknown	Up	-	-	-
gi|23615579	aspartate carbamoyltransferase	43224	8.55	21	17.1	Q8IDP8_PLAF7	Metabolism	Cytoplasm	Up	Down	-	-
gi|23615157	erythrocyte membrane protein 1, PfEMP1	385533	5.62	20	13.8	Q8IEU9_PLAF7	Pathogenesis	Membrane	Up	-	-	-
gi|124804730	conserved Plasmodium protein, unknown function	120306	9.15	20	12.3	Q8IHV2_PLAF7	Unknown	Unknown	Up	-	-	-
gi|225631639	histone acetyltransferase, putative	146635	6.29	23	9.1	C0H4A7_PLAF7	Nucleosome Assembly	Nucleus	Up	-	Down	-
gi|124804435	T-complex protein 1 subunit alpha	60223	6.65	47	18.2	Q8II43_PLAF7	Protein fate	Cytoplasm	Up	-	-	-
gi|124805672	conserved Plasmodium protein, unknown function	25749	6.04	19	14	Q8I5V2_PLAF7	Unknown	Unknown	Up	-	-	-
gi|23615411	conserved Plasmodium protein, unknown function	79471	8.88	24	9.4	Q8IE64_PLAF7	Unknown	Unknown	Up	-	-	-
gi|3758855	conserved Plasmodium protein, unknown function	402704	4.68	18	8.8	LRR4_PLAF7	Unknown	Cytoplasm	Up	-	-	-
gi|23498756	inositol-phosphate phosphatase, putative	330496	7.09	18	10.1	Q8IC15_PLAF7	Regulation	Unknown	Up	-	-	-
gi|124802292	conserved Plasmodium protein, unknown function	267819	7.84	18	11.5	Q8IJP6_PLAF7	Unknown	Unknown	Up	-	-	-
gi|124804827	conserved Plasmodium protein, unknown function	175302	6.41	18	10.7	Q8IHS3_PLAF7	Unknown	Unknown	Up	-	-	-
gi|124803597	transcription factor with AP2 domain(s)	206747	5.63	18	12.4	Q8IIS4_PLAF7	Unknown	Unknown	Up	-	Down	-
gi|23499166	probable protein, unknown function	187594	9.38	18	4.9	Q8IAW4_PLAF7	Unknown	Membrane	Up	-	-	-
gi|124803042	rifin	40212	8.67	18	29.5	Q8IJ09_PLAF7	Cell Surface	Membrane	Up	Down	-	-
gi|224591363	Pfmc-2TM Maurer~s cleft two transmembrane protein	27543	9.69	18	16.2	B9ZSH7_PLAF7	Transport	Membrane	Up	-	-	-
gi|124805437	conserved Plasmodium protein, unknown function	20310	9.22	18	4.7	Q8I614_PLAF7	Unknown	Unknown	Up	-	-	-
gi|124806077	transcription factor with AP2 domain(s)	301724	6.89	18	8.9	Q8I5J1_PLAF7	Unknown	Unknown	Up	-	-	-
gi|7768291	40S ribosomal protein S11, putative	18748	10.18	18	15.5	O77381_PLAF7	Translation	Cytoplasm	Up	-	-	-
gi|3758843	conserved Plasmodium protein, unknown function	592883	9.05	17	12.6	O77372_PLAF7	Unknown	Membrane	Up	-	-	-
gi|23504617	conserved Plasmodium protein, unknown function	331924	9.2	17	12	Q8I3X5_PLAF7	Unknown	Nucleus	Up	-	-	-
gi|23504632	conserved Plasmodium protein, unknown function	190407	9.71	24	9	Q8I3W1_PLAF7	Unknown	Membrane	Up	Down	-	-
gi|225632147	conserved Plasmodium protein, unknown function	196449	8.24	16	11	C0H4L6_PLAF7	Unknown	Unknown	Up	-	-	-
gi|7264041	pre-mRNA-splicing factor PRP46, putative	69529	8.6	16	6.7	O97334_PLAF7	Transcription	Nucleus	Up	-	-	-
gi|23498873	ferrodoxin reductase-like protein	72675	8.91	26	24.9	Q8IBP8_PLAF7	Cell Rescue Defense	Unknown	Up	Down	-	-
gi|23504638	phosphatidylinositol 3-kinase	255758	9.27	16	11.2	Q8I3V5_PLAF7	Regulation	Food Vacuole	Up	Up	-	**Up**
gi|258596863	protein kinase, putative	293584	6.84	16	13	O96134_PLAF7	Regulation	Cytoplasm	Up	-	-	-
gi|258596939	DnaJ protein, putative	117215	8.77	25	18.7	O96276_PLAF7	Unknown	Unknown	Up	Down	-	-
gi|225685561	conserved Plasmodium protein, unknown function	83028	8.83	16	13.2	C0H479_PLAF7	Unknown	Unknown	Up	-	-	-
gi|46361062	transketolase	75767	6.5	62	15.6	C6KSV3_PLAF7	Metabolism	Cytoplasm	Up	-	-	-
gi|46362240	rifin	41283	8.18	15	17.8	C6KSK9_PLAF7	Cell Surface	Membrane	Up	-	-	-
gi|23499205	rifin	43647	8.43	15	12.9	Q8IAS5_PLAF7	Cell Surface	Membrane	Up	-	-	-
gi|225631825	50S ribosomal protein L3, apicoplast, putative	36251	9.95	15	14.2	C0H543_PLAF7	Translation	Cytoplasm	Up	-	-	-
gi|124809291	cleavage and polyadenylation specificity factor subunit 3, putative	101120	5.29	15	8.6	Q8IL83_PLAF7	Transcription	Nucleus	Up	-	-	-
gi|3758842	cleavage and polyadenylation specificity factor,putative	118775	8.49	15	12.1	O77371_PLAF7	Transcription	Nucleus	Up	-	-	-
gi|23504588	peptidyl-prolyl cis-trans isomerase	86985	6.98	15	9.4	Q8I402_PLAF7	Protein fate	Unknown	Up	-	-	-
gi|124809181	conserved Plasmodium protein, unknown function	30461	5.08	15	11	Q8ILA9_PLAF7	Unknown	Cytoplasm	Up	-	-	-
gi|124810013	WD repeat-containing protein, putative	113162	5.51	14	8.6	Q8IKN9_PLAF7	Unknown	Unknown	Up	-	-	-
gi|225631940	conserved Plasmodium protein, unknown function	737235	9.13	21	11.3	C0H5A6_PLAF7	Unknown	Membrane	Up	Down	Down	**Down**
gi|124802320	heat shock protein 60	62512	6.71	65	33.1	Q8IJN9_PLAF7	Protein fate	Cytoplasm	-	-	-	-
gi|258596910	T-complex protein 1 subunit theta	60920	7.79	60	27.1	O96220_PLAF7	Regulation	Cytoplasm	-	Down	-	-
gi|23504543	small ubiquitin-related modifier	11053	4.74	52	38	Q8I444_PLAF7	Metabolism	Nucleus	-	Down	Down	**Down**
gi|124803903	heat shock protein 101	102810	9.17	35	21.2	Q8IIJ8_PLAF7	Metabolism	Membrane	-	Up	-	-
gi|258597233	60S ribosomal protein L36	12779	11.07	33	17	Q8I713_PLAF7	Translation	Cytoplasm	-	-	Down	-
gi|124803477	uncharacterized protein PF3D7_1104400	49235	9.18	37	21	Q8IIV8_PLAF7	Unknown	Endoplasmic Reticulum	-	-	-	-
gi|23505091	DnaJ protein, putative	43233	8.81	59	28.1	Q8I2W2_PLAF7	Unknown	Membrane	-	Up	-	-
gi|6562734	replication protein A1, large subunit	134118	6.58	30	14.2	Q9U0J0_PLAF7	DNA Replication	Unknown	-	-	Up	-
gi|124802200	PRE-binding protein	131545	9.19	30	14.3	Q8IJS7_PLAF7	Transcription	Nucleus	-	Up	Up	**Up**
gi|124806691	erythrocyte membrane protein 1, PfEMP1	261440	5.15	22	12.8	Q8I519_PLAF7	Pathogenesis	Membrane	-	-	Down	-
gi|6562724	sporozoite invasion-associated protein 1	112862	6.27	24	13.7	Q9U0K0_PLAF7	Unknown	Unknown	-	Up	-	-
gi|258597681	conserved protein, unknown function	49106	9.61	21	10.1	Q8ILT3_PLAF7	Unknown	Unknown	-	-	Down	-
gi|225632022	phosphatidylinositol transfer protein, putative	220966	5.88	20	14.1	Q8IDI9_PLAF7	Unknown	Cytoplasm	-	Down	-	-
gi|225631642	conserved Plasmodium protein, unknown function	821866	9.5	22	11.9	C0H4B0_PLAF7	Unknown	Membrane	-	-	Up	-
gi|23510631	asparagine-rich antigen Pfa35-2	363848	6.47	25	11.7	Q8I284_PLAF7	Nucleosome Assembly	Cytoplasm	-	-	Down	-
gi|74862728	RecName: Full=Pentatricopeptide repeat-containing protein PFL1605w	169454	7.95	24	12.3	PPR1_PLAF7	Unknown	Cytoplasm	-	Down	-	-
gi|47168426	Chain A, Uridine Phosphorylase, Putative	30441	5.83	141	35.5	Q8I3X4_PLAF7	Metabolism	Unknown	-	Down	-	-
gi|312208188	Chain A, Endoplasmin homolog	32001	5.76	135	25.6	Q8I0V4_PLAF7	Protein fate	Endoplasmic Reticulum	-	Down	-	-
gi|284055700	Chain A, Pyruvate kinase	56744	6.98	102	38.7	C6KTA4_PLAF7	Metabolism	Cytoplasm	-	Down	-	-
gi|23505108	inosine-5~-monophosphate dehydrogenase	56115	7.99	63	34.1	Q8I2U5_PLAF7	Metabolism	Cytoplasm	-	Down	Up	-
gi|1297293	60 kDa heat-shock protein PfHsp60	62125	7.05	49	32.6	Q27723_PLAFA	Protein fate	Cytoplasm	-	Down	-	-
gi|23615562	casein kinase II beta chain	45257	3.66	42	15.6	Q8IDR5_PLAF7	Unknown	Cytoplasm	-	-	Up	-
gi|124802989	enhancer of rudimentary homolog, putative	12313	8.65	38	29.5	Q8IJ30_PLAF7	Cell Cycle	Unknown	-	Down	Up	-
gi|225631665	conserved Plasmodium protein, unknown function	469585	7.47	35	11.8	C0H4C9_PLAF7	Unknown	Unknown	-	Down	-	-
gi|23615772	conserved Plasmodium protein, unknown function	386087	8.76	35	10.4	Q8ID63_PLAF7	Unknown	Membrane	-	Down	-	-

gi|258596983	conserved Plasmodium protein, unknown function	45596	7.12	32	23	Q8IJX4_PLAF7	Unknown	Unknown	-	Down	-	-
gi|23504660	FACT complex subunit SPT16, putative	132600	4.86	31	9.1	Q8I3T4_PLAF7	Trascription	Nucleus	-	Down	-	-
gi|46361188	hexokinase	55226	6.72	53	31.2	C6KT76_PLAF7	Metabolism	Cytoplasm	-	Down	Down	**Down**
gi|225631797	AP-4 complex subunit epsilon, putative	161304	5.44	29	14.5	Q8I3A8_PLAF7	Transport	Cytoplasm	-	Down	-	-
gi|152968490	cyclic nucleotide-specific phosphodiesterase	97106	6.54	31	9.6	Q8IKD3_PLAF7	Metabolism	Membrane	-	-	-	-
gi|124800762	conserved Plasmodium protein, unknown function	83770	8.54	28	14.8	O96141_PLAF7	Unknown	Unknown	-	Down	-	-
gi|23615441	conserved Plasmodium protein, unknown function	205350	9.22	28	12.9	Q8IE35_PLAF7	Unknown	Unknown	-	-	-	-
gi|46362258	conserved Plasmodium protein, unknown function	153622	8.47	27	17.5	C6KSM6_PLAF7	Unknown	Unknown	-	Down	-	-
gi|23505253	ring-exported protein 1	82991	5.46	27	7.4	Q8I2G1_PLAF7	Unknown	Cytoplasm	-	Down	-	-
gi|23615272	conserved Plasmodium protein, unknown function	277526	7.79	25	10.3	Q8IEJ4_PLAF7	Unknown	Unknown	-	Down	-	-
gi|124810495	conserved protein, unknown function	115321	8.18	25	14.9	Q8IK86_PLAF7	Unknown	Cytoplasm	-	Down	-	-
gi|124803955	heat shock protein 90, putative	108397	6.22	34	9.1	Q8III6_PLAF7	Protein fate	Mitochondria	-	-	-	-
gi|124809222	cGMP-dependent protein kinase	97632	5.4	24	15.4	Q8I719_PLAF7	Regulation	Cytoplasm	-	Down	-	-
gi|124810493	conserved Plasmodium protein, unknown function	185792	9.12	24	15.5	Q8IK87_PLAF7	Unknown	Unknown	-	Down	-	-
gi|23498211	conserved protein, unknown function	27337	6.78	23	19.1	Q8I1U2_PLAF7	Unknown	Unknown	-	Down	-	-
gi|23498752	conserved Plasmodium protein, unknown function	118270	8.1	23	9	YPF13_PLAF7	Unknown	Unknown	-	Down	Down	**Down**
gi|23504673	mRNA-binding protein PUF1	223948	8.2	21	12.6	Q8I3S3_PLAF7	Translation	Unknown	-	Down	-	-
gi|225631658	conserved Plasmodium protein, unknown function	46993	8.92	21	20.5	C0H4C2_PLAF7	Unknown	Unknown	-	Down	-	-
gi|23498891	rhoptry-associated leucine zipper-like protein 1	87825	5.31	27	19.4	Q8IBN1_PLAF7	Unknown	Rhoptry	-	Down	-	-
gi|23498854	mitochondrial ribosomal protein S8 precursor,putative	14793	9.98	20	13.2	Q8IBR7_PLAF7	Translation	Cytoplasm	-	Down	-	-
gi|23505129	3-oxoacyl-[acyl-carrier-protein] reductase	33326	9.33	20	15	Q8I2S7_PLAF7	Metabolism	Unknown	-	Down	-	-
gi|124809066	conserved Plasmodium protein, unknown function	143906	8.94	20	11.2	Q8ILD6_PLAF7	Unknown	Nucleus	-	Down	-	-
gi|23615541	ABC transporter B family member 5, putative	108443	8.47	20	11.2	Q8IDT6_PLAF7	Unknown	Membrane	-	Down	-	-
gi|225632201	inositol 5-phosphatase, putative	172950	9.68	27	19.4	C0H4Q9_PLAF7	Unknown	Membrane	-	Down	-	-
gi|23615413	conserved Plasmodium protein, unknown function	185578	7.09	20	12.3	Q8IE62_PLAF7	Unknown	Unknown	-	Down	-	-
gi|23499167	RNA-binding protein (U1 snRNP-like), putative	27861	8.34	20	28.9	RU1C_PLAF7	Transcription	Nucleus	-	Down	-	-
gi|124805668	porphobilinogen deaminase	50315	9.54	20	24.4	Q8I5V3_PLAF7	Metabolism	Apicoplast	-	Down	Up	-
gi|46361176	leucine--tRNA ligase, putative	170133	6.12	20	8.5	C6KT64_PLAF7	Translation	Cytoplasm	-	-	-	-
gi|124802394	conserved Plasmodium protein, unknown function	137321	9.36	20	11.2	Q8IJM1_PLAF7	Unknown	Unknown	-	Down	-	-
gi|124802282	transcriptional coactivator ADA2	300067	8.79	20	9.9	Q8IJP9_PLAF7	Transcription	Nucleus	-	Down	-	-
gi|124805693	conserved Plasmodium protein, unknown function	24532	5.4	19	11.5	Q8I5U6_PLAF7	Unknown	Unknown	-	Down	-	-
gi|124801201	conserved Plasmodium membrane protein, unknown function	493899	8.46	19	9.1	O96204_PLAF7	Unknown	Unknown	-	Down	-	-
gi|23498892	ubiquitin carboxyl-terminal hydrolase, putative	145399	8.9	19	13.5	Q8IBN0_PLAF7	Protein fate	Cytoplasm	-	Down	-	-
gi|124808290	conserved Plasmodium protein, unknown function	39806	8.57	18	16.2	Q8ILY8_PLAF7	Unknown	Unknown	-	Down	-	-
gi|124804142	leucine-rich repeat protein	93275	8.97	18	13.9	Q8IID1_PLAF7	Unknown	Unknown	-	Down	Down	**Down**
gi|225631849	conserved Plasmodium protein, unknown function	102515	8.83	18	11.7	C0H567_PLAF7	Unknown	Unknown	-	Down	Down	**Down**
gi|258597363	3-oxo-5-alpha-steroid 4-dehydrogenase, putative	35445	9.53	18	5.4	Q8II05_PLAF7	Metabolism	Cytoplasm	-	Down	-	-
gi|23615204	conserved Plasmodium protein, unknown function	160471	9.3	17	8.8	Q8IEQ5_PLAF7	Unknown	Membrane	-	Down	-	-
gi|258597491	triose or hexose phosphate/phosphate translocator, putative	54687	9.13	17	13.2	Q8I5M4_PLAF7	Transport	Unknown	-	Down	-	-
gi|225631826	conserved Plasmodium protein, unknown function	205540	8.72	17	11.3	C0H544_PLAF7	Unknown	Membrane	-	Down	-	-
gi|124804710	conserved Plasmodium protein, unknown function	219717	9.63	16	8.6	Q8IHV9_PLAF7	Unknown	Unknown	-	Down	-	-
gi|124802905	merozoite surface protein 6	42250	4.32	18	13.2	Q8IJ54_PLAF7	Invasion	Unknown	-	-	Up	-
gi|124806291	RNA pseudouridylate synthase, putative	48335	9.57	17	19.3	Q8I5D3_PLAF7	Metabolism	Unknown	-	Down	Up	-
gi|23498776	Cg7 protein	153379	6.98	15	13.9	Q8IBZ5_PLAF7	Unknown	Unknown	-	Down	-	-
gi|124808195	DNA repair helicase, putative	135460	8.96	18	22.2	Q8IM12_PLAF7	Metabolism	Nucleus	-	-	-	-
gi|124807008	osmiophilic body protein	377151	5.71	14	8.5	Q8I4T3_PLAF7	Unknown	Cytoplasm	-	Down	-	-
gi|23499027	receptor for activated c kinase	35664	6.24	62	15.2	Q8IBA0_PLAF7	Unknown	Cytoplasm	-	Up	Down	-
gi|124801080	ATP-dependent RNA helicase UAP56	52191	5.68	49	12	Q9TY94_PLAF7	Transcription	Nucleus	-	Up	-	-
gi|225632223	coatomer subunit epsilon, putative	33071	4.59	44	14.7	C0H4T1_PLAF7	Transport	Cytoplasm	-	Up	-	-
gi|23499022	conserved Plasmodium protein, unknown function	100876	5.92	35	7.7	Q8IBA5_PLAF7	Unknown	Unknown	-	Up	-	-
gi|46361219	conserved protein, unknown function	43622	7.19	33	19.9	C6KTA3_PLAF7	Unknown	Unknown	-	Up	-	-
gi|124800772	hexose transporter	56380	8.8	33	12.7	Q7KWJ5_PLAF7	Unknown	Membrane	-	Up	-	-
gi|23505020	conserved Plasmodium protein, unknown function	188566	9.15	34	15.6	Q8I332_PLAF7	Unknown	Apicoplast	-	Up	Down	-
gi|124801981	40S ribosomal protein S20e, putative	13504	9.67	34	17.8	Q8IK02_PLAF7	Translation	Cytoplasm	-	Up	-	-
gi|23499165	conserved Plasmodium protein, unknown function	70662	9.11	30	4.8	Q8IAW5_PLAF7	Unknown	Cytoplasm	-	Up	-	-
gi|124808563	H/ACA ribonucleoprotein complex subunit 4,putative	52908	9.06	27	14.6	Q8ILS0_PLAF7	Metabolism	Cytoplasm	-	Up	-	-
gi|225632241	ATPase, putative	139581	8.66	27	9.8	Q8IAX9_PLAF7	Unknown	Unknown	-	Up	-	-
gi|258597683	conserved Plasmodium protein, unknown function	77169	8.98	27	22.6	Q8ILT2_PLAF7	Unknown	Unknown	-	Up	-	-
gi|23615185	DNA ligase I	104440	7.66	27	10.3	Q8IES4_PLAF7	DNA Replication	Nucleus	-	Up	-	-
gi|258597895	conserved Plasmodium membrane protein, unknown function	388575	8.84	25	12.3	Q8IKJ2_PLAF7	Unknown	Membrane	-	Up	-	-
gi|225632177	conserved Plasmodium protein, unknown function	420410	8.7	25	12	C0H4P2_PLAF7	Unknown	Unknown	-	Up	-	-
gi|258597178	Pfmc-2TM Maurer~s cleft two transmembrane protein	27162	9.36	25	22.5	Q8IIY8_PLAF7	Transport	Membrane	-	Up	-	-
gi|160736	vacuolar ATPase	68532	5.51	24	20.6	VATA_PLAFA	Metabolism	Cytoplasm	-	Up	-	-
gi|124809489	poly(A)-specific ribonuclease PARN, putative	102192	7.72	24	15.5	Q8IL36_PLAF7	Unknown	Unknown	-	Up	-	-
gi|46361184	transcription or splicing factor-like protein,putative	70382	5.75	24	8.8	C6KT72_PLAF7	Transcription	Unknown	-	Up	-	-
gi|1373027	apical membrane antigen-1, partial	68829	5.23	24	15.6	Q7JPC6_PLAFA	Pathogenesis	Membrane	-	Up	-	-

gi|124810469	splicing factor 3A subunit 1, putative	83698	5.27	23	3.1	Q8IK93_PLAF7	Transcription	Nucleus	-	Up	-	-
gi|23505236	TFIIH basal transcription factor complex helicase XPD subunit	122760	5.94	22	15.8	Q8I2H7_PLAF7	DNA Replication	Cytoplasm	-	Up	-	-
gi|124808162	conserved Plasmodium protein, unknown function	240981	9.51	36	13	Q8IM18_PLAF7	Unknown	Unknown	-	Up	Up	**Up**
gi|23505235	histidine--tRNA ligase, putative	78134	9.51	22	16.7	Q8I2H8_PLAF7	Unknown	Cytoplasm	-	Up	-	-
gi|258597547	aspartyl protease, putative	77044	7.79	21	12	Q8I578_PLAF7	Metabolism	Food Vacuole	-	Up	-	-
gi|23505153	proline--tRNA ligase, putative	68423	9.33	21	10.4	Q8I2Q5_PLAF7	Translation	Unknown	-	Up	-	-
gi|23615369	conserved Plasmodium membrane protein, unknown function	404731	8.76	21	14.6	Q8IEA1_PLAF7	Unknown	Membrane	-	Up	Up	**Up**
gi|23498252	regulator of chromosome condensation, putative	236211	9.23	21	9.2	Q8I1Q1_PLAF7	Unknown	Unknown	-	Up	Up	**Up**
gi|23615188	conserved Plasmodium protein, unknown function	72454	6.55	20	5.1	Q8IES1_PLAF7	Unknown	Unknown	-	Up	-	-
gi|225631931	condensin complex subunit 2, putative	119143	4.87	20	15.3	C0H598_PLAF7	Cell Cycle	Nucleus	-	Up	-	-
gi|23499100	perforin-like protein 4	76298	8.61	20	18.7	Q8IB29_PLAF7	Unknown	Membrane	-	Up	-	-
gi|23505203	peptidyl-prolyl cis-trans isomerase	72507	9.11	25	15.8	Q8I2K8_PLAF7	Protein fate	Nucleus	-	Up	Up	**Up**
gi|124806649	protein kinase 2	58876	8.77	18	35.6	Q8I534_PLAF7	Protein Signaling	Cytoplasm	-	Up	-	-
gi|124809797	RAP protein, putative	208094	9.38	18	7.4	Q8IKU2_PLAF7	Unknown	Unknown	-	Up	-	-
gi|225632270	histone acetyltransferase GCN5	170810	6.25	18	10.3	Q8IB67_PLAF7	Nucleosome Assembly	Nucleus	-	Up	-	-
gi|23504560	subtilisin-like protease 1	77598	5.68	18	12.6	Q8I0V0_PLAF7	Protein fate	Cytoplasm	-	Up	-	-
gi|23615425	myosin C	250131	8.84	18	11	Q8IE50_PLAF7	Cytoskeleton	Cytoplasm	-	Up	-	-
gi|15375380	P-loop containing nucleoside triphosphate hydrolase, putative	132116	5.07	17	13.2	O97242_PLAF7	Metabolism	Unknown	-	Up	-	-
gi|258597726	conserved Plasmodium protein, unknown function	248348	9.01	30	10.2	Q8ILJ6_PLAF7	Unknown	Unknown	-	Up	Up	**Up**
gi|23505002	conserved Plasmodium protein, unknown function	368118	5.27	16	10.9	Q8I350_PLAF7	Unknown	Unknown	-	Up	Down	-
gi|23498863	conserved Plasmodium protein, unknown function	205848	9.17	16	7.5	Q8IBQ8_PLAF7	Unknown	Membrane	-	Up	-	-
gi|23498942	conserved Plasmodium protein, unknown function	114757	9.19	16	3.6	Q8IBI0_PLAF7	Unknown	Unknown	-	Up	-	-
gi|124806958	conserved Plasmodium membrane protein, unknown function	81491	9.45	14	10.6	Q8I4U4_PLAF7	Unknown	Unknown	-	Up	-	-
gi|124801397	40S ribosomal protein S26	12501	10.98	37	29	O96258_PLAF7	Translation	Cytoplasm	-	-	Down	-
gi|15375385	glutaredoxin 1	12410	7.68	62	42.3	Q9NLB2_PLAF7	Cell Rescue Defense	Cytoplasm	-	-	Up	-
gi|258597165	antigen UB05	13703	9.44	28	20.2	Q8IJ28_PLAF7	Unknown	Unknown	-	-	Up	-
gi|23504580	RNA polymerase I	340462	5.75	30	12.1	Q8I410_PLAF7	Transcription	Nucleus	-	-	-	-
gi|296004752	erythrocyte membrane protein 1, PfEMP1	255615	5.41	32	15.4	A0A143ZZY8_PLAF7	Pathogenesis	Membrane	-	-	Up	-
gi|124801175	tyrosine kinase-like protein, putative	148133	9.57	18	10	O96197_PLAF7	Protein Signaling	Cytoplasm	-	-	Down	-
gi|124809914	V-type H(+)-translocating pyrophosphatase,putative	76367	6.14	100	12.3	Q8IKR1_PLAF7	Regulation	Membrane	-	-	-	-
gi|124804821	40S ribosomal protein S21	9140	8.66	73	43.9	Q8IHS5_PLAF7	Translation	Cytoplasm	-	-	Up	-
gi|225632186	signal recognition particle subunit SRP9	12127	9.45	65	59.2	C0H4Q0_PLAF7	Translation	Cytoplasm	-	-	-	-
gi|23504624	ras-related protein Rab-1A	23844	6.89	47	33.3	Q8I3W9_PLAF7	Unknown	Endoplasmic Reticulum	-	-	Down	-
gi|124805527	conserved Plasmodium protein, unknown function	38994	4.84	45	12	Q8I5Y9_PLAF7	Unknown	Unknown	-	-	Down	-
gi|67463796	Chain A, Deoxyuridine 5~-triphosphate Nucleotidohydrolase	19561	6.53	44	17.3	Q8II92_PLAF7	DNA Replication	Unknown	-	-	-	-
gi|23476987	glycogen synthase kinase 3	51583	5.4	38	21.1	O77344_PLAF7	Unknown	Membrane	-	-	Down	-
gi|258597173	Pfmc-2TM Maurer~s cleft two transmembrane protein	27356	9.63	34	26	Q8IJ11_PLAF7	Transport	Membrane	-	-	Down	-
gi|224591381	Pfmc-2TM Maurer~s cleft two transmembrane protein	26882	9.71	34	28.4	B9ZSJ3_PLAF7	Transport	Membrane	-	-	Down	-
gi|23504959	conserved Plasmodium protein, unknown function	139901	8.75	34	11.7	Q8I391_PLAF7	Unknown	Nucleus	-	-	Down	-
gi|258597261	pyridoxine biosynthesis protein PDX2	24547	6.43	34	18.7	A0A144A0A4_PLAF7	Unknown	Unknown	-	-	Down	-
gi|23505080	glideosome-associated protein 50	44576	8.69	42	16.9	Q8I2X3_PLAF7	Unknown	Membrane	-	-	Up	-
gi|23505011	conserved Plasmodium protein, unknown function	137031	9	33	12.2	Q8I341_PLAF7	Unknown	Unknown	-	-	Down	-
gi|74873111	RecName: Full=Uncharacterized protein PFB0145c	237599	5.77	33	16.4	YB145_PLAF7	Unknown	Cytoplasm	-	-	Down	-
gi|124805363	Plasmodium exported protein (PHISTb), unknown function	77069	4.4	27	6.3	Q8I635_PLAF7	Unknown	Unknown	-	-	Down	-
gi|23498201	phosphoglucomutase-2	35210	9.52	27	16.3	Q8I1V2_PLAF7	Metabolism	Membrane	-	-	Down	-
gi|23615683	serine/threonine protein phosphatase 5	76840	5.06	25	13.5	Q8IDE7_PLAF7	Unknown	Unknown	-	-	Down	-
gi|7672216	phosphatidylinositol 3-and 4-kinase, putative	110490	6.16	25	15.2	O77353_PLAF7	Unknown	Unknown	-	-	Down	-
gi|124806008	cell cycle associated protein, putative	323873	9.26	23	11.1	Q8I5L0_PLAF7	Unknown	Unknown	-	-	Down	-
gi|124810087	conserved Plasmodium protein, unknown function	59322	5.28	23	23	Q8IKM2_PLAF7	Unknown	Unknown	-	-	Down	-
gi|23615547	RNA lariat debranching enzyme, putative	67513	5.21	23	9.9	Q8IDT0_PLAF7	Transcription	Nucleus	-	-	Down	-
gi|124802645	conserved Plasmodium protein, unknown function	196369	9.26	23	9.6	Q8IJD6_PLAF7	Unknown	Unknown	-	-	Down	-
gi|23498929	DNA mismatch repair protein PMS1, putative	156741	5.48	22	6.4	Q8IBJ3_PLAF7	DNA Replication	Cytoplasm	-	-	Down	-
gi|23615502	conserved Plasmodium protein, unknown function	48840	9	21	4.9	Q8IDX4_PLAF7	Unknown	Unknown	-	-	Down	-
gi|124809814	serine/threonine protein kinase KIN	90798	9.7	20	8.6	Q8IKT6_PLAF7	Protein Signaling	Cytoplasm	-	-	Down	-
gi|7672215	pre-mRNA splicing factor, putative	71621	10.01	20	2.2	O77355_PLAF7	Transcription	Unknown	-	-	Down	-
gi|33413784	normocyte binding protein 2b	382646	5.18	20	11.7	Q9BK45_PLAFA	Unknown	Membrane	-	-	Down	-
gi|23615500	reticulocyte binding protein 2 homologue a	370208	5.44	20	12.2	RBP2A_PLAF7	Cell Surface	Membrane	-	-	Down	-
gi|124806068	uncharacterized protein PF3D7_1222000	65650	9.23	19	10.5	Q8I5J3_PLAF7	Unknown	Unknown	-	-	Down	-
gi|258597555	clustered-asparagine-rich protein	51501	8.71	18	8.1	Q8I562_PLAF7	Unknown	Unknown	-	-	Down	-
gi|124806250	conserved Plasmodium protein, unknown function	140933	9.27	18	9.6	Q8I5E5_PLAF7	Unknown	Unknown	-	-	Down	-
gi|3894094	mitogen-activated protein kinase 2	59173	8.63	18	9.8	Q7KQK7_PLAF7	Protein Signaling	Cytoplasm	-	-	Down	-
gi|23504897	Plasmodium exported protein (PHISTb), unknown function	60150	4.93	18	18.3	Q8I3F1_PLAF7	Unknown	Unknown	-	-	Down	-
gi|3764023	parasite-infected erythrocyte surface protein	153993	5.14	18	7.6	O77361_PLAF7	Unknown	Membrane	-	-	Down	-
gi|124809929	conserved Plasmodium protein, unknown function	88742	6.56	18	19.1	Q8IKQ8_PLAF7	Unknown	Unknown	-	-	Down	-
gi|23498197	erythrocyte membrane protein 1, PfEMP1	263576	5.25	18	10.4	Q8I1V6_PLAF7	Pathogenesis	Membrane	-	-	Down	-
gi|23498196	erythrocyte membrane protein 1, PfEMP1	261684	5.28	18	10.3	Q8I1V7_PLAF7	Pathogenesis	Membrane	-	-	Down	-
gi|23504939	LCCL domain-containing protein	178224	8.81	18	11.4	Q8I3B1_PLAF7	Unknown	Cytoplasm	-	-	Down	-
gi|74930170	RecName: Full=Actin-2; AltName: Full=Actin II	42578	5.21	17	18.6	ACT2_PLAF7	Cytoskeleton	Cytoplasm	-	-	Down	-
gi|124808338	conserved protein, unknown function	81048	8.6	17	6.9	Q8ILX8_PLAF7	Unknown	Nucleus	-	-	Down	-
gi|23498172	ag-1 blood stage membrane protein homologue	69060	5.47	17	9.8	Q8I1X7_PLAF7	Unknown	Unknown	-	-	Down	-

gi|23615614	conserved Plasmodium membrane protein, unknown function	460569	7.48	17	10.8	Q8IDL5_PLAF7	Unknown	Membrane	-	-	Down	-
gi|4493884	inner membrane complex protein 1e, putative	61726	6.63	16	18.1	O97229_PLAF7	Unknown	Membrane	-	-	Down	-
gi|124804913	tubulin--tyrosine ligase, putative	325121	9.25	15	13.9	Q8IHP8_PLAF7	Metabolism	Unknown	-	-	Down	-
gi|23615797	conserved Plasmodium protein, unknown function	78525	9.38	15	9.9	Y13P2_PLAF7	Unknown	Unknown	-	-	Down	-
gi|124801837	erythrocyte membrane protein 1, PfEMP1	252173	5.26	15	15.6	Q8IK37_PLAF7	Pathogenesis	Membrane	-	-	Down	-
gi|124805350	erythrocyte membrane protein 1, PfEMP1	355019	7.25	15	13.3	Q8I639_PLAF7	Pathogenesis	Membrane	-	-	Down	-
gi|256032266	Chain A, Erythrocyte Membrane Protein 1 (pfemp1)	35553	8.3	15	31.1	Q8I639_PLAF7	Pathogenesis	Membrane	-	-	Down	-
gi|6138833	Polyubiquitin	42803	7	172	21	Q7KQK2_PLAF7	Protein fate	Cytoplasm	-	-	Up	-
gi|285803543	Chain A, M17 Leucyl Aminopeptidase	58604	6.38	57	23.3	Q8IL11_PLAF7	Unknown	Cytoplasm	-	-	Up	-
gi|23504499	skeleton-binding protein 1	36277	4.33	45	7.4	Q8I487_PLAF7	Translation	Membrane	-	-	Up	-
gi|124802833	haloacid dehalogenase-like hydrolase	32821	5.62	31	13.2	Q8IJ74_PLAF7	Regulation	Cytoplasm	-	-	Up	-
gi|258597947	conserved Plasmodium membrane protein, unknown function	64379	8.88	30	3	Q8IKB5_PLAF7	Unknown	Unknown	-	-	Up	-
gi|124810280	conserved Plasmodium protein, unknown function	295648	5.19	30	8.1	Q8IKF6_PLAF7	Unknown	Unknown	-	-	Up	-
gi|74873273	RecName: Full=GRIP and coiled-coil domain-containing protein PFC0235w	135640	4.87	30	14	YGCC1_PLAF7	Unknown	Unknown	-	-	Up	-
gi|225632280	conserved Plasmodium protein, unknown function	88381	9.76	30	12.4	C0H4Y1_PLAF7	Unknown	Unknown	-	-	Up	-
gi|23504941	GTPase-activating protein, putative	50269	4.84	30	8.5	Q8I3A9_PLAF7	Unknown	Unknown	-	-	Up	-
gi|225631662	conserved Plasmodium membrane protein, unknown function	354955	5.17	29	9	C0H4C6_PLAF7	Unknown	Membrane	-	-	Up	-
gi|225631719	conserved Plasmodium protein, unknown function	280186	5.04	29	12.5	C6KSR4_PLAF7	Unknown	Cytoplasm	-	-	Up	-
gi|23615769	6-cysteine protein	92669	6.49	41	14.3	PF92_PLAF7	Unknown	Membrane	-	-	Up	-
gi|15383897	conserved protein, unknown function	12885	10.19	29	12	Q9NLA9_PLAF7	Unknown	Unknown	-	-	Up	-
gi|124804380	26S protease regulatory subunit 6A, putative	49510	5.07	27	13.4	Q8II60_PLAF7	Transcription	Cytoplasm	-	-	Up	-
gi|124805657	U6 snRNA-associated Sm-like protein LSm7,putative	11988	5.8	28	43.5	Q8I5V6_PLAF7	Transcription	Nucleus	-	-	Up	-
gi|124809457	conserved Plasmodium protein, unknown function	300791	9.23	22	13.7	Q8IL44_PLAF7	Unknown	Unknown	-	-	Up	-
gi|23615625	conserved Plasmodium protein, unknown function	405156	8.06	22	8.6	Q8IDK4_PLAF7	Unknown	Membrane	-	-	Up	-
gi|258597814	conserved Plasmodium protein, unknown function	864485	8.74	21	8.4	Q8IL30_PLAF7	Unknown	Unknown	-	-	Up	-
gi|258597897	serine/threonine protein phosphatase UIS2,putative	170374	5.51	21	14.8	Q8IKJ1_PLAF7	Unknown	Unknown	-	-	Up	-
gi|23504667	RAP protein, putative	162718	7.96	21	7	Q8I3S7_PLAF7	Unknown	Membrane	-	-	Up	-
gi|124804507	protein disulfide isomerase	49164	8.94	19	11.1	Q8II23_PLAF7	Cell Rescue Defense	Endoplasmic Reticulum	-	-	Up	-
gi|124806715	conserved Plasmodium protein, unknown function	63686	6.73	18	6.2	Q8I509_PLAF7	Unknown	Membrane	-	-	Up	-
gi|23505270	erythrocyte membrane protein 1, PfEMP1	151890	5.66	18	13.2	Q8I2E4_PLAF7	Pathogenesis	Membrane	-	-	Up	-
gi|124807182	rifin	34492	8.89	18	12.7	Q8I4P2_PLAF7	Cell Surface	Membrane	-	-	Up	-
gi|225632049	conserved Plasmodium protein, unknown function	144043	9.66	17	8.1	C0H5K4_PLAF7	Unknown	Unknown	-	-	Up	-
gi|23615584	rhomboid protease ROM6, putative	67822	9.73	17	4	Q8IDP3_PLAF7	Unknown	Membrane	-	-	Up	-
gi|46362292	conserved Plasmodium protein, unknown function	123256	5.59	17	12.6	C6KSQ8_PLAF7	Unknown	Unknown	-	-	Up	-
gi|23615696	serine/threonine protein kinase, putative	475422	8.99	17	13.6	Q8IDD4_PLAF7	Regulation	Cytoplasm	-	-	Up	-
gi|124806489	DNA polymerase epsilon subunit B, putative	73584	6.02	17	8.2	Q8I579_PLAF7	DNA Replication	Nucleus	-	-	Up	-
gi|46361213	oxidoreductase, short-chain dehydrogenase family, putative	44730	9.39	16	23.5	C6KT97_PLAF7	Unknown	Membrane	-	-	Up	-
gi|23498967	transcription factor with AP2 domain(s)	155036	4.95	16	12.3	Q8IBF6_PLAF7	Unknown	Unknown	-	-	Up	-
gi|23615716	CUGBP Elav-like family member 1	57079	5.43	15	6.1	Q8IDB7_PLAF7	Unknown	Cytoplasm	-	-	Up	-
gi|23504871	thioredoxin-like protein, putative	22041	9.16	15	9.8	Q8I3H6_PLAF7	Unknown	Membrane	-	-	Up	-
